# Search for improved triplet-state quenchers for fluorescence imaging: a computational framework incorporating excited-state Baird-aromaticity†

**DOI:** 10.1039/d5sc01131k

**Published:** 2025-03-26

**Authors:** Ouissam El Bakouri, Matthew A. Johnson, Joshua R. Smith, Avik K. Pati, Maxwell I. Martin, Scott C. Blanchard, Henrik Ottosson

**Affiliations:** a Department of Chemistry –Ångström, Uppsala University Uppsala Sweden Henrik.Ottosson@kemi.uu.se; b Institut de Química Computacional i Catàlisi (IQCC), Departament de Química, Universitat de Girona C/Maria Aurèlia Capmany 6 17003 Girona Catalonia Spain; c Department of Chemistry & Biochemistry, Cal Poly Humboldt Arcata CA 95501 USA; d Department of Structural Biology, St. Jude Children's Research Hospital Memphis USA Scott.Blanchard@stjude.org; e Department of Chemistry, Birla Institute of Technology and Science Pilani Rajasthan 333031 India

## Abstract

Fluorescence imaging is crucial for studying biology. Triplet state quenchers (TSQs), especially cyclooctatetraene (COT), can dramatically improve fluorophore performance, particularly when linked intramolecularly so as to enable “self-healing”. Leveraging knowledge revealed through investigations of the self-healing mechanism enabled by COT, we computationally screened for cyclic 8π-electron species, and their annulated derivatives, with efficient triplet–triplet energy transfer potential, high photostability, and strong spin–orbit coupling (SOC) between the lowest triplet state to the singlet ground state. Here, we report theory-based analyses of a broad array of candidates that demonstrate various extents of triplet state Baird-aromaticity, indicating self-healing potential. We identify specific candidates with 7-membered ring structures predicted to exhibit favorable enhancements in fluorophore performance spanning the visible spectrum, with several possessing estimated intersystem crossing (ISC) rates up to 4 × 10^6^ times faster than that of COT, the current benchmark for the self-healing strategy.

## Abstract 2

Towards the goal of advancing self-healing organic fluorophore technologies, we computationally identify specific 7-membered ring molecules that exhibit much faster triplet relaxation rates than cyclooctatetraene, the current standard in the field.

## Graphical abstract

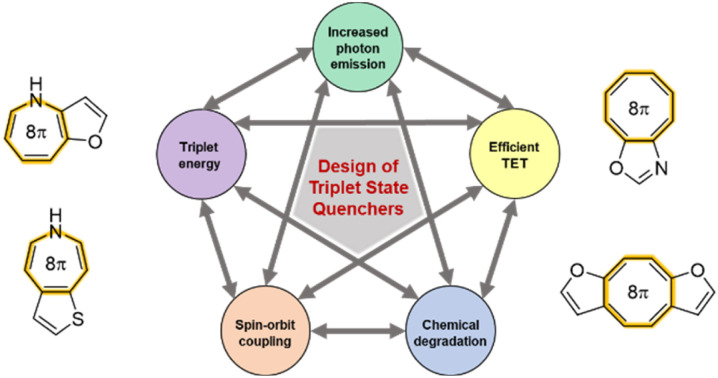

## Introduction

Single-molecule and super-resolution fluorescence microscopy techniques have become powerful tools for the improved understanding of biological processes.^1–3^ These techniques allow high specificity that can be achieved by site-specifically labelling fluorescent molecules (fluorophores) to specific proteins in cells and tissues. Organic fluorophores,^4^ in particular cyanine-^5^ and rhodamine-classes of dyes,^6^ are essential extrinsic contrast agents^7^ and they are widely used in biological and medical research. The main advantages of utilizing such organic fluorophores over other fluorescent systems such as fluorescent proteins are their small sizes, easy chemical tunability, better photostability, brightness, and the variety of organic fluorophores available that span the visible and infrared spectrum.^8^

Despite remarkable progress,^9,10^ the photostability, brightness and phototoxicity of fluorophores remain limiting factors to fluorescence imaging experiments. The most widely employed fluorophores undergo stochastic transitions to non-fluorescent and long-lived triplet excited states. Because triplet state (T_1_) lifetimes of these imaging fluorophores are nearly 100 000-fold longer than their singlet excited state lifetimes (1–10 ns), T_1_ states compromise fluorophore brightness, particularly at elevated illumination intensities and in continuous illumination settings. T_1_ states are also prone to reactivity with molecular oxygen, which quench the T_1_ state to yield reactive oxygen species (ROS).^4,8–12^ The ROS generated contributes to fluorophore photobleaching and damages neighboring biomolecules, causing phototoxicity in the biological system of interest. While oxygen can be removed from experimental settings to improve fluorophore performance, oxygen removal is often incomplete and reduced oxygen prolongs the duration of fluorophore in triplet states, giving rise to downstream reactions that result in extended-duration non-fluorescent periods that compromise data quality and lead to the generation of reactive species.

**Fig. 1:**
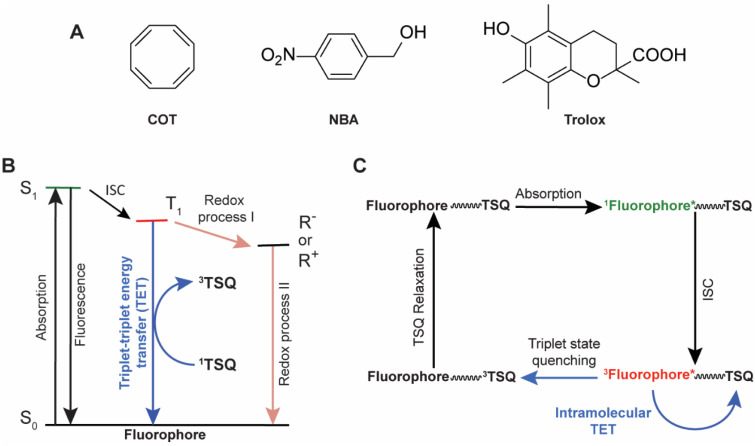
(A) Structures of commonly used TSQs: cyclooctatetraene (COT), nitrobenzyl alcohol (NBA), and 6-hydroxy-2,5,7,8-tetramethylchroman-2-carboxylic acid (Trolox). (B) A simplified energy diagram showing absorption, fluorescence, intersystem-crossing (ISC), and triplet-state quenching processes of a fluorophore by small-molecule photoprotective agents. R^+^ and R^−^ represent the radical cation and radical anion of the fluorophore, respectively. (C) A schematic diagram of the self-healing mechanism of a fluorophore–TSQ conjugate, where the TSQ quenches the triplet state of the fluorophore *via* the triplet–triplet energy transfer mechanism.

These critical drawbacks can be mitigated by the addition of exogenous solution-based triplet state quenchers (TSQs) – also referred to more generally as photoprotective agents (PAs). The most commonly employed TSQs are cyclooctatetraene (COT), 4-nitrobenzyl alcohol (NBA), and 6-hydroxy-2,5,7,8-tetramethylchroman-2-carboxylic acid (Trolox) (Fig. 1A). Alternatively, a combination of a reducing (*e.g.*, Trolox) and an oxidizing (*e.g.*, quinone derivative of Trolox) system (ROXS) may be used, as well as a “cocktail” of PAs (*e.g.*, COT, NBA and Trolox together) in imaging buffers.^8,13–15^ While solution-based TSQs can enhance the brightness and photostability of a variety of organic fluorophores in the absence of oxygen, their performances are strongly dependent on fluorophore type and the mechanism of action of the TSQs or PAs by which they quench the fluorophore triplet states (Fig. 1B).^16^ Solution-based TSQs also strongly perturb biological systems, cause varied excited state reactivities, and induce lethal toxicities in live-cell investigations.^8^ Solution-based TSQs are also ineffective in the presence of oxygen due to their limited solubilities in aqueous buffer environments.^17^ To bypass these disadvantages of solution-based TSQs, Blanchard and co-workers established an intramolecular photostabilization or “self-healing” strategy in which a single or multiple TSQs were intramolecularly linked to chemically and spectrally diverse organic fluorophores to dramatically increase the brightness and photostability by reducing the T_1_ state lifetime of the fluorophores (Fig. 1C).^17–21^

NBA, Trolox and ROXS reduce the T_1_ state lifetimes of fluorophores *via* redox-based mechanisms and generate charged intermediates (R^+^ and R^−^, Fig. 1B) that potentially reduce the fluorescent lifetime and brightness of the fluorophores (Fig. 1B).^16^ By contrast, COT decreases T_1_ state lifetimes by a triplet–triplet energy transfer (TTET or TET) mechanism in which COT as a TSQ acts as an energy acceptor (Fig. 1B).^17^ As COT-mediated triplet quenching of fluorophores is a charge–neutral mechanism, it can improve overall fluorophore performance in a manner that is substantially more independent of environmental setting. When COT is tethered directly to fluorophores across the visible spectrum it significantly outperforms “self-healing” fluorophores containing redox-active NBA or Trolox.^18^

COT is an electronically tunable, 8π-electron species.^20^ Formally, COT should be Hückel-antiaromatic in the singlet ground state (S_0_), but it distorts to a puckered geometry that is non-aromatic. On the other hand, COT in its T_1_ state adopts a planar geometry and is Baird-aromatic,^22–27^ a general feature for many cyclic 4*n*π-electron molecules, which is particularly advantageous as it leads to high photochemical stability.^28,29^ Here, we refer to photochemical stability as the resistance of triplet-state quenchers (TSQs) to chemical reactions, particularly radical-mediated degradation processes, which are common in the triplet excited state, particularly under bioimaging settings. Such resistance enables TSQs to maintain functionality under prolonged fluorophore illumination, where repetitive TET-relaxation cycles take place. Similar to Hückel-aromaticity providing stability in the S_0_ state, Baird-aromaticity affords photochemical stability by achieving aromatic stabilization in the T_1_ state (for COT estimated experimentally to be 88 kJ mol^−1^, and computationally 71 kJ mol^−1^).^30,31^ A practical aspect of aromatic stability in the T_1_ state is the reluctance of Baird-aromatic molecules to undergo radical reactions,^32–34^ and COT derivatives have been found to resist photodegradation under sensitized irradiation for up to 100 hours.^34^ As will be seen below, the reaction energies for the addition of either a methyl radical or a hydroxyl radical to a T_1_ state benzene (Baird-antiaromatic) and to a T_1_ state COT (Baird-aromatic) are markedly different, suggesting a substantially higher photostability of the latter species.

Tuning the triplet energy of COT through chemical derivatization has the potential to enhance TET rates for specific fluorophore species thereby enhancing their performance.^20^ Electronic tuning investigations have led to two important conclusions. The first is that the T_1_ dark state of the fluorophore can be efficiently quenched through TET from the fluorophore to the TSQ if the triplet energies of the fluorophore–TSQ pair are closely matched. The second is that the TET rate can be limited by the conformational processes within COT. In principle, rate-limiting processes may govern transitions between COT's distorted ground state structures to its Baird-aromatic planar octagonal form or limit transitions from its Baird-aromatic planar form back to ground state.

**Fig. 2:**
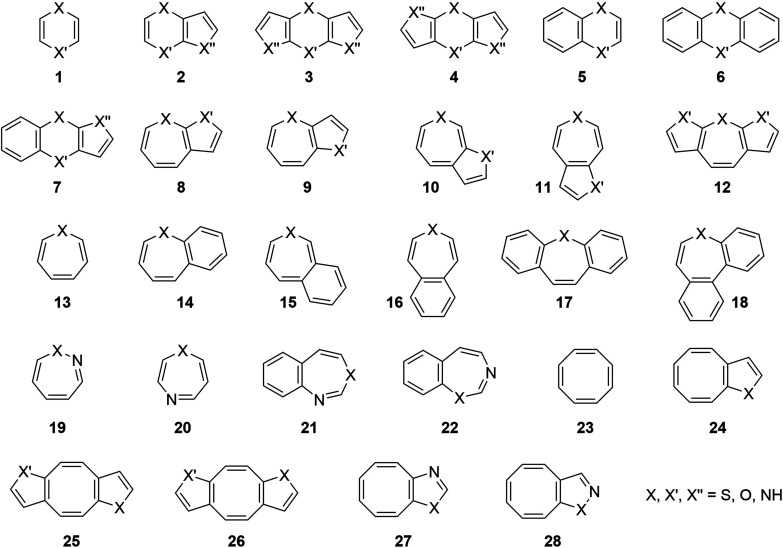
Candidate TSQ species investigated in this report, *i.e*., (hetero)cyclic 8π-electron compounds and their mono- and di(hetero)-annulated derivatives.

To further advance the self-healing approach, there is a need for the development of novel TSQ cores that can improve TET efficiency in multi-turnover settings to enhance fluorophore brightness and total photon budget.^9^ To address this need, we establish a computational framework for designing new TSQs by exploring heteroatom 8π-electron, 6–8 member-ring (MR) cycles as well as derivatives with annulated benzene and heteroarene rings (Fig. 2). These constitute a portfolio of potential TSQs (in total 214 compounds) with a large span of triplet energies (5–370 kJ mol^−1^). The photophysics of a number of the TSQ candidate compounds identified have earlier been explored experimentally.^35–49^ Many of these are influenced by (anti)aromaticity in both ground and excited states, resulting in conformations (planar or puckered) that vary with the electronic state.^50^

We further refined this set of lead compounds to identify those that possess specific characteristics deemed essential for pursuits aimed at enhancing fluorophore performance. Due to the superior performance of COT as a TSQ, incorporation of 8π-electron cycles which can be T_1_ state Baird-aromatic into our candidates was a prerequisite in our design philosophy.^24,27,28^ Based on our previous findings,^51^ we further stipulate that a fusion of Baird-aromatic 8π- and Hückel-aromatic 6π-electron cycles provides a means to vary the triplet energies (*E*_T_1__) of the TSQ candidates. The desired TSQ attributes were also stipulated to include: (i) triplet energies in the range of 80 to 200 kJ mol^−1^ so that they become suitable for common imaging probes across the visible spectrum, (ii) high photochemical stability in the T_1_ state as a result of extensive Baird-aromatic character, (iii) strong spin–orbit coupling facilitating efficient relaxation from the T_1_ to the S_0_ state to shorten T_1_ lifetimes, (iv) minimal energy requirement to transition from the S_0_ to the T_1_ state, specifically in terms of planarity, as this factor impacts TET efficiency, as observed in earlier studies involving COT,^20^ (v) a high number of heteroatoms to improve the hydrophilicity, and (vi) reduced vulnerability to chemical degradation, particularly from reactions with molecular oxygen. These properties, except hydrophilicity (v), were explored theoretically and computationally in a combined analysis that allows us to predict TSQ cores expected to exhibit dramatically higher performance than COT. The ambition is to identify compounds that are experimentally realistic candidates to function as improved TSQs in fluorescence microscopy. TSQs of this kind have the potential to maximize the efficiency and robustness of the self-healing mechanism to, and thereby, enhance fluorescence imaging pursuits.^52–57^

## Results and discussion

Herein, we provide motivations for our TSQ candidate compound selection followed by a brief discussion of the change in (anti)aromatic character upon excitation from the S_0_ state to the T_1_ state and their extents of Baird-aromatic character in the T_1_ state, as well as its impact on reaction energies for methyl and hydroxyl radical additions. We then analyze relevant computed photophysical properties followed by a combined evaluation of the various TSQ candidates based on the desired attributes (i)–(vi). Our analysis is based on results from Kohn–Sham DFT computations at the (U)M06-2X/6-311G(d,p) level using the polarization continuum model with water as solvent,^58–60^ which we previously found to provide good agreements with our experimental findings.^20^ For an assessment of the performance of various DFT functionals against DLPNO-UCCSD(T)//UDFT results, see Table S1.†

### Compound selection

Among the vast number of bi- and tricyclic compounds that can be drawn by fusing an 8π-electron heteroannulene ring with one or two 6π-electron heteroarenes, we primarily focused on those that have been generated experimentally (see ESI† for the list) or that are related to these through heteroatom replacements. Examples are the dibenz[*b*,*f*]oxepin (17, X = O) explored photophysically by Wan and Shukla,^35^ a compound whose derivatives have medicinal and biological relevance,^61^ and the recently synthesized dithienoazepine derivatives (12, X = NH, X′ = S), which are suitable as near-infrared fluorescent dyes.^62^ Structural moieties composed of other di(hetero)areno fused 8π-electron cycles are also found in pharmaceutical compounds used as antipsychotics.^50^ In our set we additionally included two compound types (10 and 15) for which one cannot draw closed-shell or non-zwitterionic resonance structures with Hückel-aromatic 6π-electron cycles in S_0_ (see Fig. S1†). The compounds in our portfolio have one, two, three or four heteroatoms being either N, O or S atoms.

As previously mentioned, it is essential to select compounds that meet our criteria for *E*_T_1__, SOC, a low inversion barrier in the S_0_ state (Δ*G*^‡^) and planarity in the T_1_ state, features which are each crucial for enhancing fluorophore performance and multi-turnover triplet energy transfer (TET) processes in which repetitive TET processes occur between donor and acceptor molecules. These properties are studied alongside their degree of Baird-aromaticity in the T_1_ state, which we utilize as an indicator of photostability.

### Baird-aromatic character in the T_1_ state and *E*_T_1__

An array of computational tools have been recently advanced to assess potential Baird-aromatic character of compounds in electronically excited states.^63^ Given the sheer abundance of TSQ compounds and the tendency of some to deviate from planarity, we employed a single electronic aromaticity index – the multicenter index (MCI)^64^ – alongside a geometric index known as the harmonic oscillator model of aromaticity (HOMA, see Table S2†).^65,66^ MCI measures electron delocalization, with higher values indicating greater aromaticity. Normalized MCI (MCI^1/*n*^, where *n* is the number of atoms in the *n*-membered ring) has been used to allow consistent comparisons between rings of different sizes by accounting for the influence of the ring size on the delocalization measure. HOMA, on the other hand, evaluates bond length equalization, with values near 1 signifying aromaticity and negative values or values near 0 suggesting non- or antiaromaticity. Further details on the MCI and HOMA indices are provided in the Computational methods section of the ESI.† The choice of MCI as our primary electronic index is grounded in its proven efficacy in providing a comprehensive assessment of aromatic character, not only in annulenes but also in heterocycles.^67^ It should be stressed that the unambiguous and unique assessment of aromatic character in polycyclic molecules is not possible as such molecules exhibit several different cyclic paths that all fulfil the aromaticity criteria.^68–71^ For polycyclic molecules which are Baird-aromatic in their T_1_ states this means cyclic 8π-, 12π- and 16π-electron paths.^51^ In our analysis we estimate the Baird-aromatic character of a polycyclic compound through the aromatic character of its 8π-electron cycle since the smallest cycle often has the strongest aromatic character.^51^

**Fig. 3:**
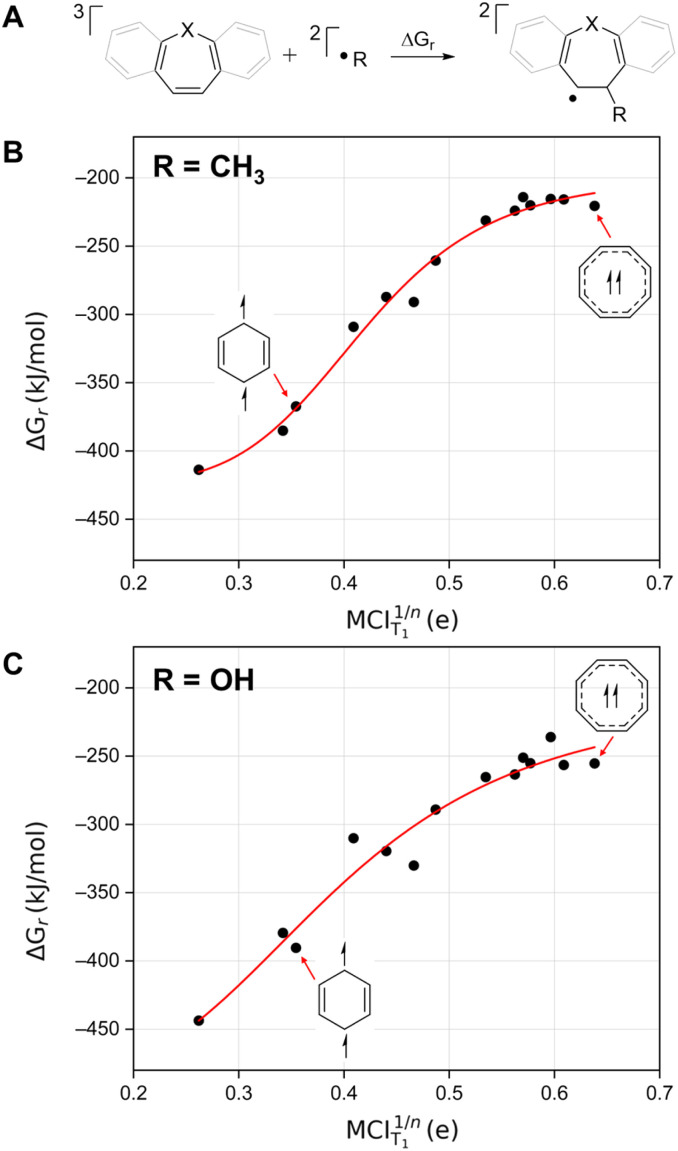
(A) Schematic depiction for the reaction of radicals with various species. Reaction energies as a function of the T_1_ state (anti)aromatic character of the (6π)8π-electron cycle as assessed through the MCI_T_1__ values for (B) methyl radical and (C) hydroxyl radical. For simplicity, only benzene and COT were shown, see Fig. S2† for the labelled plot. For unsymmetric structures containing heteroatoms, the methyl group was added to the furthest carbon from the heteroatom.

We first approached the effect of T_1_ state Baird-aromatic character on the photostability, and for this purpose explored the reaction energies for the addition of a methyl or a hydroxyl radical to a selection of species in the T_1_ state as a function of the Baird-aromatic character of the 8π-electron cycle (Fig. 3A). We also selected four 6π species (benzene, pyridinium, phosphinium, and silabenzene) in order to show the full extent of the effects of Baird (anti)aromaticity *versus* reaction energies with radicals. As seen in Fig. 3B, T_1_ state benzene has a markedly more exergonic reaction energy for methyl radical addition than T_1_ state COT, and there is a sigmoidal relationship (*R*^2^ = 0.98) between the reaction energy and the (anti)aromatic character in the T_1_ state. The non-linear nature of the data suggests that the effects of Baird-(anti)aromaticity reach a threshold where the effect on the reaction energy is diminished. Fig. 3C illustrates the same concept with the biologically more problematic hydroxyl radical and once again a non-linear trend can be observed. Utilizing the same sigmoidal function as with the methyl radical gives an acceptable fitting (*R*^2^ = 0.95). Based on the Bell–Evans–Polanyi principle we argue that the activation energies will scale with the reaction energies. Here, it is noteworthy that we earlier found that the activation energies for the H atom abstraction by T_1_ state COT and benzene were much higher for the first of these two species (150.2 *vs.* 10.0 kJ mol^−1^),^32^ and a similar trend has also been found for the cyclopropyl ring-opening by a triplet diradical benzene ring as compared to a triplet diradical COT ring.^33^ Processes that disrupt the Baird-aromatic character of a COT ring in its T_1_ state are unfavorable.

**Fig. 4:**
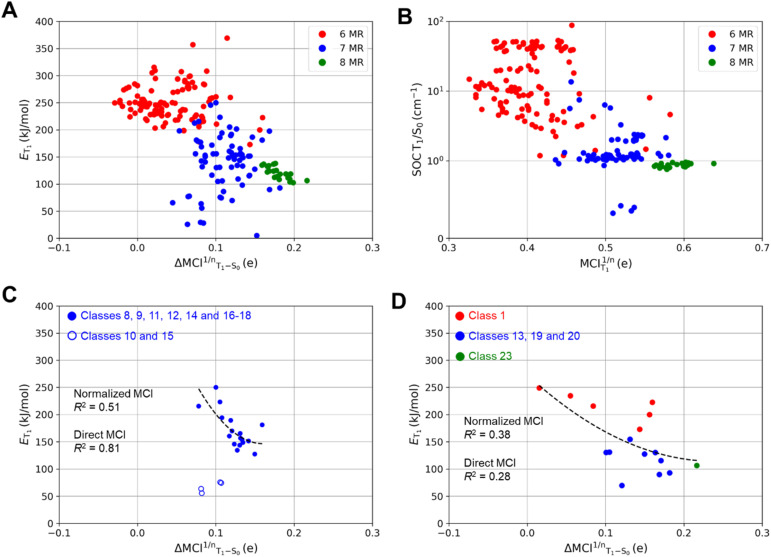
Variations of (A) the triplet energies as a function of aromaticity changes based on normalized MCI (ΔMCI^1/*n*^_T_1_–S_0__), and (B) the spin–orbit coupling as a function of aromaticity in T_1_ based on normalized MCI (MCI_T_1__^1/*n*^) of different 6-(red), 7-(blue), and 8-MR (green) compounds. Two compounds belonging to compound classes 3 and 15 were omitted. Compound 3 (X = NH, X′ = S, X″ = O) was not included as a thiol moiety is formed as the global minimum structure in the T_1_ state. Compound 15 (X = O) was excluded as an epoxide is formed instead of the oxepine ring in the S_0_ state. Variations of the triplet energies as a function of aromaticity changes based on ΔMCI^1/*n*^_T_1_–S_0__ of (C) monocyclic compounds numbers 1, 13, 19, 20 and 23, and (D) thiepines except for 10 and 15. Triplet energies, SOCs and MCI values are computed at M06-2X/6-311G(d,p) level.

According to our computations, the T_1_ states of all compounds have ππ* character but not all adopt planar T_1_ structures. In previous reports on specific monocyclic compound classes (either substituted cyclopentadienes, siloles, or pentafulvenes) there were often good correlations between the *E*_T_1__ and the change in (anti)aromaticity when going from S_0_ to T_1_ assessed by various aromaticity indices.^72,73^ However, no strict correlation between *E*_T_1__ and the (anti)aromaticity change described by ΔMCI^1/*n*^_T_1_–S_0__ (the difference in normalized MCI values between S_0_ and T_1_) is observed if one considers the complete set of compounds investigated herein (Fig. 4A). This is presumably due to the large differences between the molecules. A lack of strong correlation is also found for the strength of the spin–orbit coupling between the T_1_ and S_0_ states at the optimal T_1_ geometries and the Baird-aromatic character of the T_1_ state as given by MCI^1/*n*^ (Fig. 4B). For the SOC values one can note a levelling towards values of ∼1 cm^−1^ as the MCI^1/*n*^ exceeds a value of ∼0.5. The corresponding HOMA-based plots resemble the MCI-based ones, as seen in Fig. S3.†

Fig. 4A shows that *E*_T_1__ generally decreases when the difference in (anti)aromatic character between S_0_ and T_1_ states of a compound increases, with the 6-MR compounds distributed towards the upper left corner (high *E*_T_1__ and small (anti)aromaticity differences) while the 8-MR species cluster at the lower right corner (lower *E*_T_1__ and large (anti)aromaticity differences). However, the dispersion is very large, and four 7-MR species have lower *E*_T_1__ than any of the 8-MR ones. A closer look reveals that these 7-MR species with especially low *E*_T_1__ belong to the compound classes 10 and 15 (Fig. 2), *i.e.*, bicyclic species composed of one 8π-electron 7-MR and one 6π-electron 5-MR fused so that the latter ring due to topology cannot adopt strong Hückel-aromatic character in S_0_ (see Tables S5 and S6†).

Despite the overall lack of correlation between *E*_T_1__ and the changes in (anti)aromaticity upon excitation from S_0_ to T_1_, modest correlations are found for a few subsets of the molecules (Fig. S4 and S5†). For instance, the set of molecules with a thiepine ring (compound classes 8–18, X = S) exhibit some correlation when described mathematically through a power fit (*R*^2^ = 0.51 with normalized MCI (see Fig. 4C) and *R*^2^ = 0.81 with direct MCI (see Fig. S5E and F†)). As noted above, four thiepine derivatives were excluded from the plots as they cannot have 6π-electron Hückel-aromatic rings in S_0_, whereby their S_0_ states are destabilized relative to the other isomers. This leads to the very low *E*_T_1__ for these four compounds (Fig. S1†). In contrast to the thiepine ring, the subset of all monocyclic compounds (compound classes 1, 13, 19, 20 and 23) lack correlation between *E*_T_1__ and ΔMCI^1/*n*^(T_1_ – S_0_) (Fig. 4D). For the corresponding plots for azepines, oxepins and 8-MR compounds, see Fig. S4C, D and S5A–D.†

Combined, it is apparent that additional factors other than the (anti)antiaromaticity difference between S_0_ and T_1_ also impact *E*_T_1__. These factors include: (i) the ring size of the 8π-electron ring, (ii) the π-conjugation strength of the heteroatom(s) in the 8π-electron cycle, (iii) the number and position of the heteroatoms in both 8π- and 6π-electron rings, and (iv) the number of fused cycles leading to 12π- and 16π-electron cycles which also can adopt Baird-aromaticity in T_1_. Here, it should once more be emphasized that the assessment of the (anti)aromatic character in T_1_ by their 8π-electron cycles is an approximation for the bi- and tricyclic species. To determine the full extent of which Baird aromaticity impacts *E*_T_1__, comparative examples with Baird anti-aromatic and non-aromatic molecules should also be considered. However, in this study we only focus on (hetero)annelated 8π-electron species.

As for the strength of the SOC between the T_1_ and S_0_ states at the optimal T_1_ geometries, it decreases as the Baird-aromatic character of the 8π-electron ring increases (Fig. 4B). Thus, the 6-MR compounds display the highest and the 8-MRs the lowest SOC, except for four 7-MR species with exceptionally low SOC values (see Tables S7 and S8†). In contrast to the *E*_T_1__ values, we are unable to identify any subset of the compounds for which a high degree of Baird-aromaticity correlates with a low SOC value. The cause of the very low SOC values for the four 7-MR species is unclear, yet, we note that it is the same four species with exceptionally low *E*_T_1__ (compounds 10 and 15 with X = S).

**Fig. 5:**
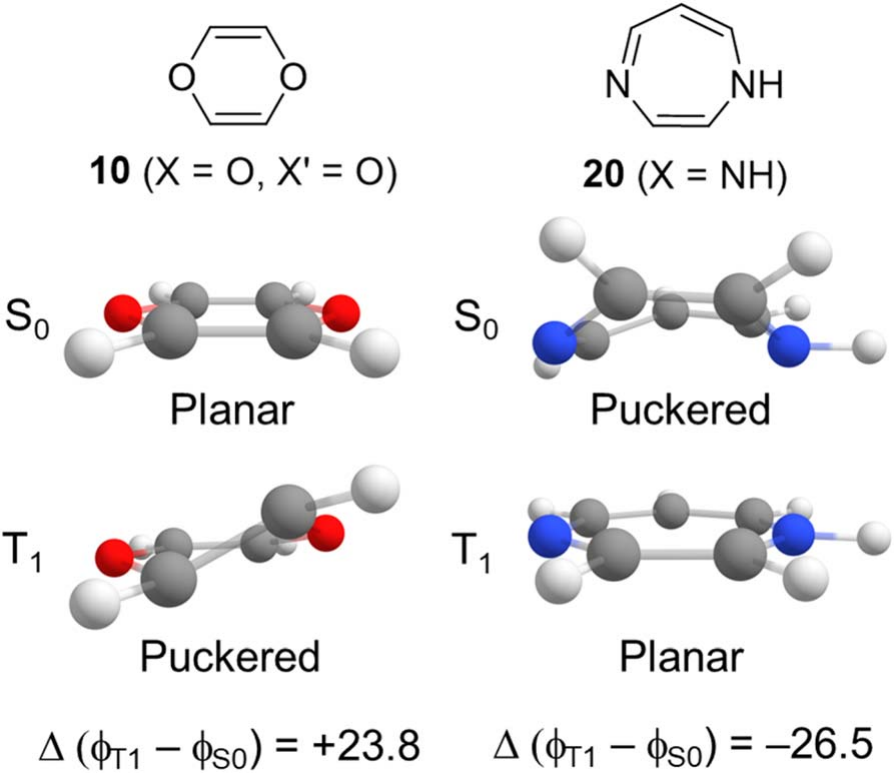
Examples of the difference in average dihedral angles from S_0_ to T_1_ for compounds 10 and 20.

**Fig. 6:**
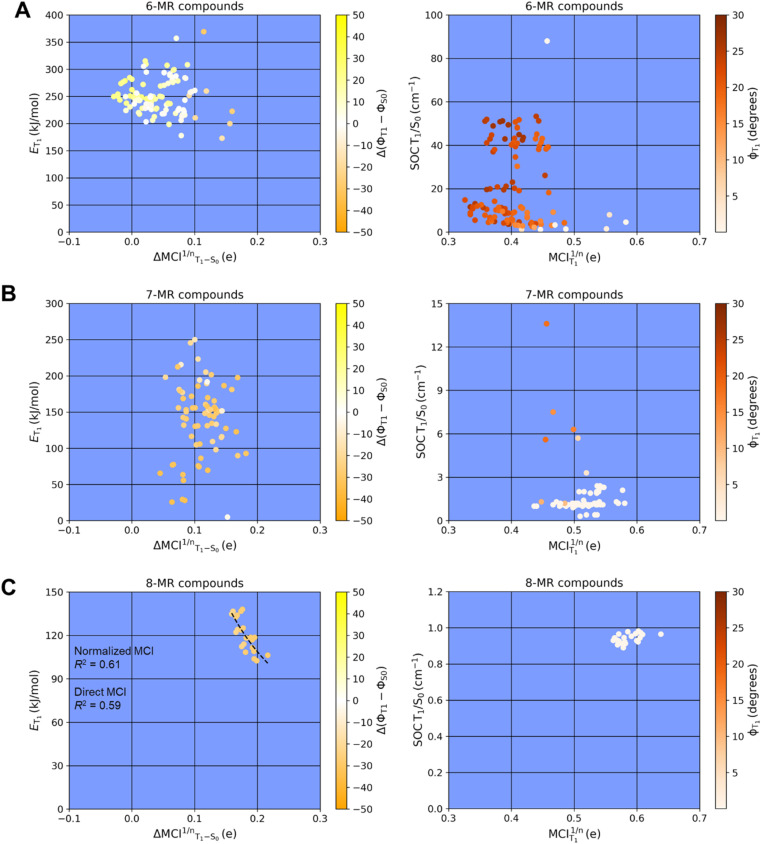
Variations of the triplet energies as a function of aromaticity changes based on normalized MCI (ΔMCI^1/*n*^_T_1_–S_0__) (left panels) and the spin–orbit coupling in dependence of aromaticity in T_1_ based on normalized MCI (MCI_T_1__^1/*n*^) (right panels) at different geometric parameters, *i.e.*, dihedral angles (*ϕ*, color scale), for the (A) 6-, (B) 7-, and (C) 8-MR compounds. Compound 3 (X = NH, X′ = S, X″ = O) was not included as a thiol moiety is formed at the minimum structure in the T_1_ state. Four compounds were not included; 3, 15, 19 and 20. Compound 15 (X = O) was excluded as an epoxide is formed instead of the oxepine ring in the S_0_ state. Triplet energies, SOCs and MCI values are computed at M06-2X/6-311G(d,p) level. Note the different scales in *E*_T_1__ and SOC values between 6-, 7- and 8-MR compounds. Legends next to each plot show color scales for Δ(*ϕ*_T_1__ − *ϕ*_S_0__) values, with scales from yellow (positive) to orange (negative) for left panels and from white (zero) to dark red (positive) for right panels.

Important parameters in the analysis are the differences in ring dihedral angles between the S_0_ and T_1_ states (Fig. 5) and the T_1_ state dihedral angles which reflect, respectively, the change towards planarity when going from S_0_ to T_1_ and the planarity in T_1_. To quantify the geometric changes from S_0_ to T_1_, we utilize the average absolute values in the dihedral angle differences between T_1_ and S_0_, Δ(*ϕ*_T_1__ − *ϕ*_S_0__). For further information on how these are calculated see Table S3† and Computational methods in the ESI.† Positive values of Δ(*ϕ*_T_1__ − *ϕ*_S_0__) indicate structural changes from a planar S_0_ state to a puckered T_1_ state (Fig. 5). Negative Δ(*ϕ*_T_1__ − *ϕ*_S_0__) values indicate structural changes from a puckered S_0_ state to a planar T_1_ state. It is worth noting that if the value of Δ(*ϕ*_T_1__ − *ϕ*_S_0__) is close to zero, the structural changes from S_0_ to T_1_ are minimal. We saw in Fig. 4A, and we see for each specific *n*-membered ring type (*n* = 6, 7 or 8) in Fig. 6 (left panels), that the 8π-electron 6-MR compounds have, in general, the highest *E*_T_1__, while the 8-MR ones have the lowest, and the 7-MR TSQ candidates exhibit a large spread. The left panels of Fig. 6 also shed light on geometric changes in the transition from S_0_ to T_1_ within the 8π-electron ring by utilizing the Δ(*ϕ*_T_1__ − *ϕ*_S_0__) values. The values of Δ(*ϕ*_T_1__ − *ϕ*_S_0__) are reflected by the color of the data points. Near-zero values (white data points) imply small differences between the S_0_ and T_1_ geometries, whereas large positive or negative values imply the opposite. Negative values for certain TSQ candidates indicate that their T_1_ state structures are less puckered than those in S_0_, and positive values the opposite. Nearly all 7- and 8-MR TSQ candidates have negative Δ(*ϕ*_T_1__ − *ϕ*_S_0__), which reveals planarization upon excitation to T_1_.

As noted above, the change in (anti)aromatic character upon excitation from S_0_ to T_1_ is generally larger for the 8-MR ones, lower for the 6-MR compounds (nonaromatic in both states), while the changes in (anti)aromaticity for the 7-MR compounds vary. The extent of Baird-aromatic character in the T_1_ state is also related to the extent of planarity in T_1_, given by the color of the data points in the right panels of Fig. 6, because all 8-MR and most 7-MR species have planar 8π-electron rings in T_1_. Similar plots with HOMA as an aromaticity descriptor, instead of MCI, are available in ESI (Fig. S6†), from which analogous conclusions can be drawn as those presented in Fig. 6.

Non-planar T_1_ state structures were particularly notable among compounds with 8π-electron 6-MRs. This puckering behavior may be attributed to both the avoidance of steric congestion in their planar conformations and the repulsion between the two unpaired same-spin π-electrons, *i.e.*, Pauli repulsions, within the small 8π-electron cycle. Here it is noteworthy that the larger 7- and 8-MRs of bi- and tricyclic compounds exhibit stronger Baird-aromaticity, despite the increase in both angle strain and steric congestion between H atoms at adjacent rings at planar structures. Lastly, despite the absence of a correlation with aromaticity, it is worth noticing that the (anti)aromaticity changes when going from S_0_ to T_1_, assessed by ΔMCI^1/*n*^_T_1_–S_0__ become slightly more pronounced as the number of atoms in the 8π-electron cycle increases (Fig. 6, left panels).

### Suitable photophysical properties for optimal TSQ performance

Matched triplet energies of the fluorophore and a TSQ is considered an important factor for efficient TET.^20^ While triplet energies determine the compatibility of the TSQ agents with common fluorophores, SOC values, and the inversion barriers in S_0_, represent pivotal factors in the identification of optimal TSQs to ensure efficient relaxation to the singlet ground state. Therefore, a key question is whether these properties are in any way linked to the degree of T_1_ state Baird-aromaticity estimated by the normalized MCI_T_1__ of the 8π-electron cycle.

Given that common imaging fluorophores emit in the spectral range of 450–850 nm (141–265 kJ mol^−1^), promising TSQ candidates should exhibit triplet energies that range from 80 to 200 kJ mol^−1^ as this energy range overlaps with the triplet energies of common imaging probes across the visible spectrum. It is, nonetheless, noteworthy that the *E*_T_1__ of all of the 8-MR compounds fall within the optimal range (80 ≤ *E*_T_1__ ≤ 200 kJ mol^−1^), and for most of the 7-MR derivatives, as well as for four 6-MR compounds (1 (X = X′ = NH), 1 (X = NH, X′ = S), 2 (X = S, X′ = X″ = NH) and 3 (X = S, X′ = X″ = NH)).

To ensure that transitions from S_0_ to T_1_ are energetically feasible, structural changes during the TET process should be minimized.^20^ As shown above, all 7- or 8-MRs compounds exhibited negative Δ(*ϕ*_T_1__ − *ϕ*_S_0__) values, revealing that the T_1_ states are more planar compared to the S_0_ states. In contrast, the 6-MR compounds mostly showed positive values, indicating that their T_1_ states are more puckered. Only two of the 6-MR compounds (1 (X = X′ = NH) and 2 (X = X′ = NH, X″ = S)) had *E*_T_1__ within the desired range and negligible structural changes in terms of planarity when going from S_0_ to T_1_.

Another important factor is the rate of intersystem crossing (ISC) of the TSQ from T_1_ to S_0_ (*k*_ISC_), particularly in continuous illumination settings, where multi-turnover TET processes must occur. To explore this aspect of the self-healing mechanism, one can derive the *k*_ISC_ through Marcus theory,^74^ where the *k*_ISC_ values depend on the adiabatic singlet-triplet energy gap, the reorganization energies (*λ*), and the SOCs. Here, a small reorganization energy is desirable as it enhances the T_1_ → S_0_*k*_ISC_ by improving the overlap of the nuclear wavefunctions, and thereby reducing the energetic barrier in the normal region within the Marcus theory framework. To justify if our candidates lie in the normal region, we plot the values of *E*_T_1__ (−Δ*E* for T_1_ → S_0_) *versus λ* (Fig. S7A†). The molecules which fall below the reference line (where *E*_T_1__ = *λ*) lie in the normal region in a Marcus theory framework, and molecules above the reference line lie in the inverted region. Here, we find that all 8-MR candidates are in the normal region as are all but ten of the 7-MR candidates (see Fig. S7B† for the ten structures). Additionally, for this approximation to be applied successfully, SOC values must be weak. Hence, in the present work, we calculate the *k*_ISC_ for compounds with SOC values between 0 and 10 cm^−1^, as is the case for all 8-MR and for all but one of the 7-MR TSQ candidates (Fig. 6, right panels).

A potential TSQ will exhibit short T_1_ state lifetimes if the ISC process from T_1_ to S_0_ is rapid, which generally require high SOC values (strong coupling). However, the ISC process can be sufficiently fast if other factors than SOC, such as the reorganization energy and the singlet-triplet energy gap, can compensate. At this point, it should be noted that SOC values below 10 cm^−1^ are labelled as weak, values in the range 10–100 cm^−1^ as medium, and those above 100 cm^−1^ as strong.^75^ As a reference species, we use COT, which according to our calculations has a weak SOC value of 1.0 cm^−1^. Thus, while a SOC value of 2.0 cm^−1^ for a given TSQ candidate compound remains weak, this would represent a doubling compared to COT and may lead to increased ISC. While the difference of 1 cm^−1^ in computed SOC values is within the expected error of the method,^54^ the observed trends in SOC values remain informative for understanding how structural modifications influence intersystem crossing efficiency. These results should be interpreted as indicative of general trends rather than precise absolute values, given factors such as ring size, and the presence of heteroatoms. Additionally, molecular planarity, singlet-triplet energy gaps, and orbital symmetry effects can further influence SOC values. Solvent effects may also introduce variations, making direct quantitative comparisons challenging.

Although there is no correlation between the SOC and normalized MCI values of the T_1_ state, *i.e.*, MCI_T_1__^1/*n*^ (Fig. 6, right panels), one can note that for both 7- and 8-MR TSQ candidates, the lower SOC values appear to be associated with structural features such as increased planarity in the T_1_ state and, to some extent, Baird-aromatic character. However, these trends should not be interpreted as a direct causal relationship, as other factors including the number of heteroatoms and conformational flexibility also contribute significantly to SOC values. This is reflected in the magnitude of the SOC values (*y*-axis of Fig. 6) where a pronounced drop in SOC values is observed across the 6-, 7-, and 8-MR classes, accompanied by slight increases in aromaticity (measured by MCI and HOMA). For the 7-MR TSQ candidates several exhibit SOC values in the range 2–14 cm^−1^, while all 8-MR TSQ candidates, which are markedly Baird-aromatic in T_1_, have SOC values slightly below 1.0 cm^−1^. In contrast, the 6-MR compounds exhibit the highest SOC values (up to 126 cm^−1^). Yet, there are also two 6-MR compounds with more modest SOC values (7.2 and 9.2 cm^−1^), *E*_T_1__ in the desired energy range, and minimal changes in geometry between the S_0_ and T_1_ states, requiring near planarity in S_0_.

**Fig. 7:**
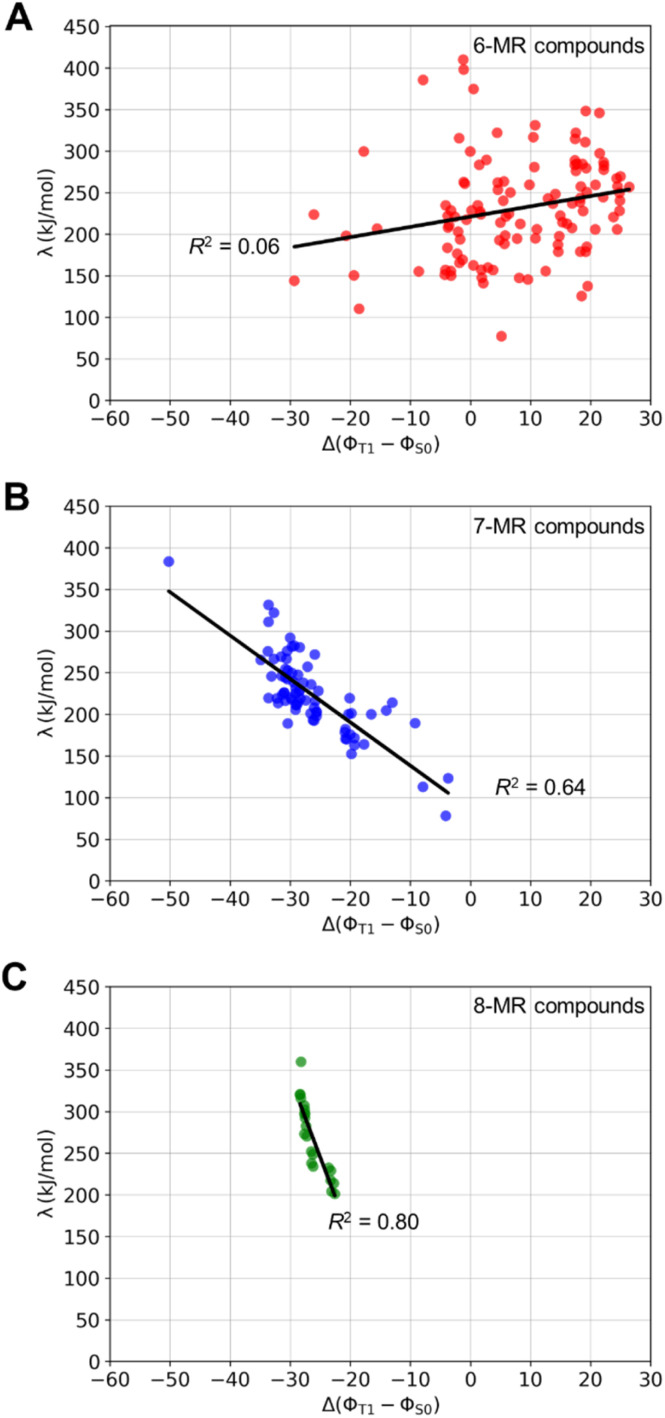
Reorganization energies (*λ*) plotted against the differences in average dihedral angles between the S_0_ and T_1_ states (Δ(*ϕ*_T_1__ − *ϕ*_S_0__)) as a measure of the change in puckering for (A) 6-, (B) 7-, and (C) 8-MR compounds. *λ* and Δ(*ϕ*_T_1__ − *ϕ*_S_0__) are computed at the M06-2X/6-311G(d,p) level.

With regard to reorganization energies, we examined the relationship between *λ* and differences in puckering between the S_0_ and T_1_ states (Δ(*ϕ*_T_1__ − *ϕ*_S_0__)), where we find that the impact of planarity on *λ* varies among different compound classes (Fig. 7). For 6-MR TSQ candidates, a lack of correlation (*R*^2^ = 0.06, Fig. 7A) indicates that the structural reorganization, as given by *λ*, primarily involves changes in bond lengths rather than dihedral angles. Furthermore, the Δ(*ϕ*_T_1__ − *ϕ*_S_0__) values are positive for many of those compounds, which indicate that the T_1_ structures are more puckered than those in the S_0_ state. Stronger correlations between *λ* and Δ(*ϕ*_T_1__ − *ϕ*_S_0__) were found for 7- and 8-MR TSQ candidates (*R*^2^ = 0.64 and 0.80, respectively, Fig. 7B and C), revealing that the energy required for S_0_ to T_1_ structural transitions is mostly due to changes in the degree of puckering. This observation illustrates the importance of low inversion barriers of the 8π-electron ring in TSQ compounds.

The computed *k*_ISC_ for COT is 2 × 10^9^ s^−1^. The computed *k*_ISC_ for 7- and 8-MR TSQ candidates were of a similar or higher order (10^8^–10^36^ s^−1^), and substantially greater than for 6-MR species (10^−23^–10^6^ s^−1^). The low *k*_ISC_ for 6-MR species are likely due to their higher triplet energies (see Table S4†). The compound with the highest *k*_ISC_, 15 (X = NH), *k*_ISC_ = 1.7 × 10^32^ s^−1^, is not suitable as a TSQ due to its minute *E*_T_1__ of 4.9 kJ mol^−1^. The highest computed *k*_ISC_ for species with 80 ≤ *E*_T_1__ ≤ 200 kJ mol^−1^ were generally found for bicyclic 7-MRs. A strong linear correlation was observed when plotting the natural logarithm of the *k*_ISC_ (ln(*k*_ISC_)) *vs. E*_T_1__ for the 7-MRs, where the higher the triplet energy, the slower the ISC process, in agreement with Fermi's golden rule (Fig. S8A†).^74^ This correlation was poor for the 8-MRs as the *E*_T_1__ for these species fall within a much smaller range of 100–140 kJ mol^−1^ (Fig. S8B†).

**Fig. 8:**
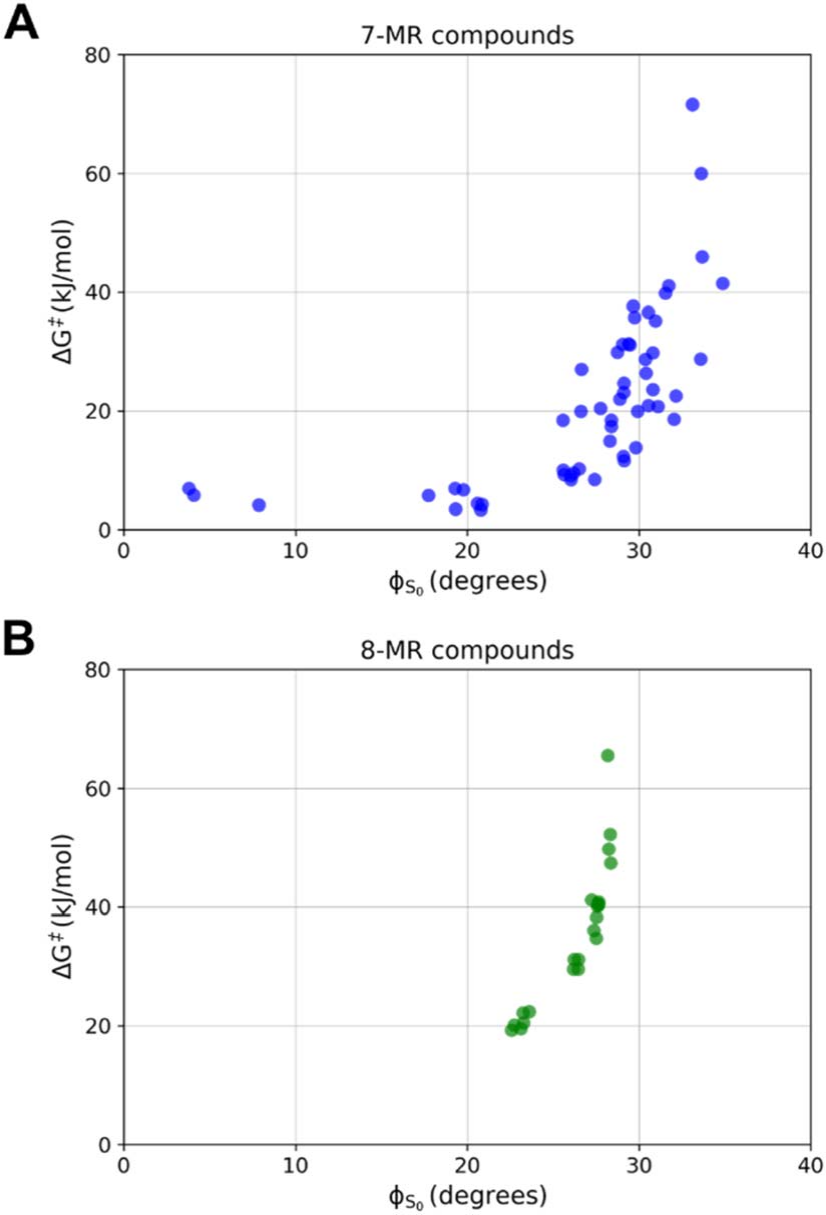
Variations of the dihedral angle average in the S_0_ state (*ϕ*) and the inversion barrier (Δ*G*^‡^) for (A) 7- and (B) 8-MR compounds. The Δ*G*^‡^ and *ϕ* are computed at the PCM-M06-2X/6-311G(d,p) level.

For Baird-aromaticity to contribute to the photochemical stability of a TSQ it is crucial that the compound can achieve planarity in its T_1_ state. This links to the conformational rigidity of the planar T_1_ state, which is closely related to the planarity discussed earlier, particularly for the 7-MR and 8-MR compounds. Rigidity is also pivotal for ensuring efficient triplet–triplet energy transfer (TET), as it reduces reorganization energy during transitions between singlet and triplet states (Fig. 7). While the T_1_ energy itself is determined by electronic and structural factors, conformational rigidity indirectly supports efficient TET processes by minimizing energy losses due to geometric distortions. To achieve their relatively planar and Baird-aromatic T_1_ state structures, 7-MRs and 8-MRs primarily undergo puckering, while 6-MRs, as described above, undergo bond length changes. These variations are significantly reduced if one considers that a planarization process in the S_0_ state can easily occur for molecules with inversion in the range 0–35 kJ mol^−1^ (Fig. 8), whereby Δ(*ϕ*_T_1__ − *ϕ*_S_0__) should be close to 0. These findings suggest that many 7- and 8-MR compounds are promising candidates. Interestingly, several 7-MR TSQ candidates exhibit low inversion barriers (≤20 kJ mol^−1^) despite being rather puckered in S_0_ with *ϕ*_S_0__ values up to 30° (Fig. 8A).

### Combined considerations for TSQ candidate selection

**Fig. 9:**
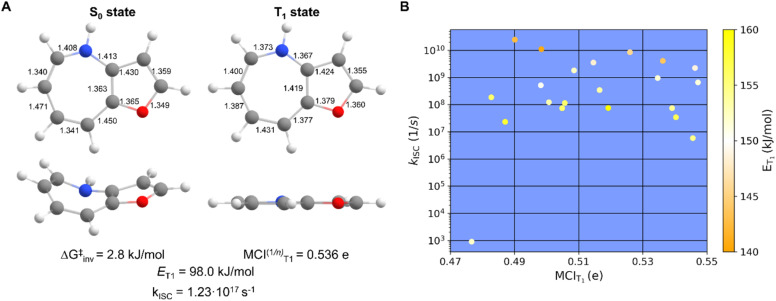
(A) One of the most suitable TSQ candidate compound 9 (X = NH, X′ = O) with the computed inversion barrier in S_0_, bond lengths (Å), normalized MCI value of 7-MR in T_1_, the *E*_T_1__ and rate constant for ISC (*k*_ISC_), and (B) a plot of the *k*_ISC_*versus* MCI_T_1__ for TSQ candidates with *E*_T_1__ in the range 140.1–160.0 kJ mol^−1^ and with the *E*_T_1__ values color coded.

When considering the attributes (i)–(vi) given in the Introduction for suitable TSQ candidates, 54 out of 75 7-MRs and 21 out of 22 8-MRs fall within the desirable ranges. An example is compound 9 with X = NH and X′ = O (Fig. 9A). When plotting the *k*_ISC_ of TSQ candidates with *E*_T_1__ in the range 140.1–160.0 kJ mol^−1^ against their degrees of T_1_-state aromaticity assessed by MCI_T_1__ (Fig. 9B), one can see that there is no obvious connection between the two properties and that *E*_T_1__, given by the color of the data point, is only weakly correlated to *k*_ISC_. Nonetheless, Baird-aromaticity, while not quantitatively correlated with *E*_T_1__ or *k*_ISC_, serves as a qualitative indicator of structural and electronic stability in the T_1_ state, a key factor to ensure photochemical stability (Fig. 3).

A further criterion addresses singlet oxygen formation, which can occur through the photophysical process: ^3^TSQ + ^3^O_2_ → TSQ + ^1^O_2_. The reaction energy of this process should be endergonic (Table S4† and footnote *a* with a discussion), and all 8-MR compounds satisfy this criterion, while the number of viable 7-MR species reduces from 54 to 38. Furthermore, one should consider the undesired chemical reaction: ^3^TSQ + ^3^O_2_ → [TSQ–O_2_] that lead to ROS. Rewardingly, this process is also endergonic with triplet state COT, but strongly exergonic with triplet state benzene (see Fig. S9†). COT, with an *E*_T_1__ of 106 kJ mol^−1^, which is within the energy range 100–120 kJ mol^−1^, has the lowest *k*_ISC_ (2 × 10^9^ s^−1^). Indeed, within this group there are TSQ cores with estimated *k*_ISC_ which are 10^6^ times higher than that of COT (see Table S9 and Fig. S10†).

An important energetic consideration for the TSQ candidates is that the *E*_T_1_–S_1__ gap is sufficiently large to mitigate quenching of the S_1_ state of the fluorophore with the S_1_ state of the TSQ candidate. For example, the *E*_S_1__ of cyanine fluorophores Cy3, C5, and Cy7 are 210.5, 179.1, and 154.8 kJ mol^−1^, respectively.^20^ The *E*_S_1__ of the TSQ candidates presented here is typically large (∼200–350 kJ mol^−1^, Table S9†), which will prevent destructive interactions between the S_1_ states of the fluorophore and TSQ. Additionally, by providing a range of TSQ candidates with different *E*_T_1__ and *E*_S_1__ values, an informed decision can be made such that TSQ agents can be chosen to match the energetics of a desired fluorophore.

With regard to the number of heteroatoms, there is no clear change in the properties of the 7- or 8-MR compounds when increasing the number of heteroatoms in the central 8π-electron ring. This suggests that one can incorporate multiple heteroatoms in the TSQs and enhance their solubility in water without compromising the efficiency of the TSQ, which is ideal from an applications perspective. The properties of TSQ candidate, compound 9 (Fig. 9A), do not change extensively when computations are run with explicit water molecules coordinated to the two heteroatoms (see Fig. S13†). Additionally, we modified 14 and 28 with methoxy groups, modeling for solubilizing alkyl ethers, and these compounds bear resemblance to the methoxy substituted dibenz[*b*,*f*]oxepines found naturally in plants.^61^ Importantly, these substituents have little impact on the computed T_1_ energy of the TSQ, which is ideal if polar substitutions are required (see Fig. S14†). We note in this context that such perturbations may not be essential, as COT, with its poor solubility in aqueous media, has proven to be an effective TSQ.^76–78^ This is important because *E*_T_1__ increases when an 8π-electron cycle is fused with gradually more 6π-electron cycles.^51^

## Conclusions

Taken together, this study unveils a portfolio of potential TSQ candidates exhibiting a range of triplet energies compatible with fluorophores spanning the visible spectrum. The selection process employed to identify these candidates was supported by an understanding of factors governing TSQ function, including SOC, Baird-aromaticity, and structural dynamics during the transition from S_0_ to T_1_ states. At the same time, our analysis considered the necessary compromise between Baird-aromatic character and ISC from T_1_ to S_0_ to ensure robust TSQ performance in elevated illumination intensity and the need for multi-turnover TET processes in continuous illumination settings. While no universal correlation was revealed among the parameters examined, we find that fused 7-MR compounds appear to emerge as particularly promising TSQ candidates. These compounds display the crucial attributes necessary for elevating the performance of fluorophores, *i.e.*, low inversion barriers in S_0_, reasonably high T_1_/S_0_ SOC, *E*_T_1__ within the desired range, and notably, their Baird-aromatic character in T_1_ suggests the potential for significant photostability.

Computational studies of the kinds employed here are likely to prove crucial for progress on the frontier of employing self-healing strategies for increasingly diverse applications as the chemical space in the search for new TSQs is experimentally intractable.^9,79,80^ We speculate that the compounds identified in the present analysis may represent tractable core structures that exhibit beneficial TSQ properties in experimental settings. They may also serve as important stepping stones towards development of more robust TSQs. Furthermore, our findings could function as a foundation for machine learning models aimed at identifying TSQ candidates with even greater suitability than those explored herein, by leveraging the features and parameters examined in this study to predict and optimize new candidates. These candidate compounds, as well as derivatives covalently bound to fluorophores must now be examined experimentally for their performance in “self-healing” contexts to reveal their potential to practically advance imaging applications across diverse life science applications.

## Data availability

The data supporting the findings of this article are provided in the ESI.†

## Author contributions

Conceptualization by H. O., O. E. B., S. C. B. and A. K. P.; investigation by O. E. B., M. A. J. and J. R. S.; writing – original draft by O. E. B., A. K. P., M. A. J. and M. I. M.; writing – review & editing by all authors; supervision by H. O. and S. C. B.

## Conflicts of interest

There are no conflicts of interest to declare.

## Supplementary Material

SC-016-D5SC01131K-s001

SC-016-D5SC01131K-s002
