# Supplementary material for: Search for improved triplet-state quenchers for fluorescence imaging: a computational framework incorporating excited-state Baird-aromaticity
Source: Chem Sci. 2025 Mar 26;16(18):7989–8001. doi: 10.1039/d5sc01131k (PMC11974263; doi:10.1039/d5sc01131k)
Supplement: SC-016-D5SC01131K-s001 [file SC-016-D5SC01131K-s001.pdf]

Supplementary Information (SI) for Chemical Science.
This journal is © The Royal Society of Chemistry 2025

```
1 (X = NH X' = NH)
S0
E = -265.444720925 A.U.
G = -265.371904 A.U.
C        1.182646    0.665021    0.071955
C        1.182646   -0.665021    0.071955
N        0.000000   -1.402919   -0.229703
C       -1.182646   -0.665021    0.071955
C       -1.182646    0.665021    0.071955
N       -0.000000    1.402919   -0.229703
H        2.076992    1.238478    0.268078
H        2.076992   -1.238478    0.268078
H        0.000000   -2.315068    0.208310
H       -2.076992   -1.238478    0.268078
H       -2.076992    1.238478    0.268078
H       -0.000000    2.315068    0.208310

T1
E = -265.371555415 A.U.
G = -265.306048 A.U.
C        1.204681    0.706968    0.016037
C        1.204681   -0.706902   -0.016085
N        0.000021   -1.360411    0.000032
C       -1.204681   -0.706968    0.016037
C       -1.204681    0.706902   -0.016085
N       -0.000021    1.360411    0.000032
H        2.101283    1.302497    0.013465
H        2.101196   -1.302571   -0.013430
H        0.000042   -2.368915    0.000025
H       -2.101283   -1.302497    0.013465
H       -2.101196    1.302571   -0.013430
H       -0.000042    2.368915    0.000025
```

```
1 (X = NH X' = O)
S0
E = -285.304296609 A.U.
G = -285.244991 A.U.
C       -0.052767    0.643631    1.174290
C       -0.052767   -0.683560    1.156609
C       -0.052767   -0.683560   -1.156609
C       -0.052767    0.643631   -1.174290
N        0.171759    1.405314   -0.000000
H       -0.209240    1.187028    2.094768
H       -0.193294   -1.297989    2.032352
H       -0.193294   -1.297989   -2.032352
H       -0.209240    1.187028   -2.094768
H       -0.323803    2.287396   -0.000000
O        0.149120   -1.427939    0.000000

T1
E = -285.218386055 A.U.
G = -285.162870 A.U.
C        0.756088    1.142610   -0.190493
C       -0.647336    1.195131    0.217061
C       -0.763775   -1.113404   -0.078358
C        0.588340   -1.208975    0.078778
N        1.349868   -0.080239    0.035758
```

H	1.385268	2.017129	-0.157972
H	-1.225451	2.103031	0.128787
H	-1.428466	-1.960258	-0.128759
H	1.089292	-2.160635	0.158768
H	2.356438	-0.165400	0.045841
O	-1.403258	0.079454	-0.057362

---

1 (X = NH X′ = S)
S0
E = -608.290589528 A.U.
G = -608.235002 A.U.

C	0.205456	0.958418	1.199242
C	0.205456	-0.365222	1.334306
C	0.205456	-0.365222	-1.334306
C	0.205456	0.958418	-1.199242
N	-0.158672	1.591096	0.000000
H	0.540020	1.610511	1.997078
H	0.573736	-0.858641	2.221547
H	0.573736	-0.858641	-2.221547
H	0.540020	1.610511	-1.997078
H	-0.021591	2.590185	0.000000
S	-0.376634	-1.396871	-0.000000

T1
E = -608.207466375 A.U.
G = -608.158895 A.U.

C	-0.038355	1.015001	1.213566
C	-0.038355	-0.390691	1.342125
C	-0.038355	-0.390691	-1.342125
C	-0.038355	1.015001	-1.213566
N	-0.035971	1.624173	-0.000000
H	-0.029579	1.663964	2.074555
H	-0.014669	-0.868695	2.308298
H	-0.014669	-0.868695	-2.308298
H	-0.029579	1.663964	-2.074555
H	-0.026794	2.634540	-0.000000
S	0.080475	-1.442875	0.000000

---

1 (X = O X′ = O)
S0
E = -305.161949958 A.U.
G = -305.115039 A.U.

C	-1.147668	0.662265	0.000005
C	-1.147668	-0.662265	0.000005
C	1.147668	-0.662265	0.000005
C	1.147668	0.662265	0.000005
H	-2.054851	1.246001	0.000050
H	-2.054851	-1.246001	0.000050
H	2.054851	-1.246001	0.000050
H	2.054851	1.246001	0.000050
O	0.000000	1.433612	-0.000020
O	-0.000000	-1.433612	-0.000020

T1
E = -305.063554234 A.U.
G = -305.020227 A.U.

C	0.664964	1.136878	-0.089974
C	-0.664958	1.136885	0.089914
C	-0.695329	-1.130961	-0.249026
C	0.695323	-1.130953	0.249076
H	1.254199	2.035153	-0.180268
H	-1.254190	2.035168	0.180154
H	-1.299945	-2.022807	-0.289821
H	1.299940	-2.022796	0.289933
O	1.400877	-0.005993	-0.073881
O	-1.400877	-0.005984	0.073889

---

1 (X = O X′ = S)
S0
E = -628.143908676 A.U.
G = -628.101447 A.U.

C	0.164600	0.988221	1.170604
C	0.164600	-0.327004	1.321698
C	0.164600	-0.327004	-1.321698
C	0.164600	0.988221	-1.170604
H	0.443435	1.676897	1.957175
H	0.466564	-0.783170	2.253340
H	0.466564	-0.783170	-2.253340
H	0.443435	1.676897	-1.957175
S	-0.279483	-1.427457	-0.000000
O	-0.162332	1.639656	0.000000

T1
E = -628.050027705 A.U.
G = -628.012134 A.U.

C	0.951042	-1.185598	-0.263819
C	-0.404588	-1.278302	0.276050
C	-0.274938	1.320846	-0.037157
C	1.049055	1.124455	0.102087
H	1.581780	-2.048669	-0.408560
H	-0.867967	-2.217891	0.533272
H	-0.675284	2.323268	-0.075635
H	1.742306	1.945303	0.221636
S	-1.446942	0.038480	-0.067475
O	1.680852	-0.063263	0.043240

---

1 (X = S X′ = S)
S0
E = -951.123452599 A.U.
G = -951.084446 A.U.

C	-1.328684	0.716273	0.293813
C	-0.249886	1.488638	0.293707
C	1.328684	-0.716273	0.293813
C	0.249886	-1.488638	0.293707
H	-2.269644	1.052844	0.711513
H	-0.265290	2.487920	0.711305
H	2.269644	-1.052844	0.711513
H	0.265290	-2.487920	0.711305
S	-1.328684	-0.951178	-0.309246
S	1.328684	0.951178	-0.309246

T1

E = -951.032215860 A.U.
G = -950.999698 A.U.

C	1.363874	0.702367	0.000158
C	1.363874	-0.702367	0.000158
C	-1.363874	-0.702367	0.000158
C	-1.363874	0.702367	0.000158
H	2.297241	1.246650	0.000082
H	2.297241	-1.246650	0.000082
H	-2.297241	-1.246650	0.000082
H	-2.297241	1.246650	0.000082
S	0.000000	1.709552	-0.000129
S	-0.000000	-1.709552	-0.000129

---

2 (X = NH X' = NH X'' = NH)
S0
E = -397.005898311 A.U.
G = -396.906648 A.U.

C	2.396692	0.029473	0.009650
N	1.562468	-1.062913	-0.005333
C	0.265966	-0.637630	0.007007
C	0.261215	0.732269	0.002212
C	1.616823	1.163817	-0.002174
N	-0.866312	-1.476918	-0.098231
C	-2.060585	-0.692318	0.003802
C	-2.089822	0.642322	0.011596
N	-0.948565	1.478942	-0.099009
H	3.466064	-0.091759	0.039056
H	1.858584	-2.026202	-0.016842
H	1.973241	2.182011	-0.000339
H	-0.862991	-2.220960	0.594009
H	-2.980190	-1.260391	0.020237
H	-3.040429	1.158336	0.040085
H	-0.989155	2.257591	0.549246

T1
E = -396.917020388 A.U.
G = -396.823331 A.U.

C	-2.394230	0.029123	-0.020686
N	-1.564194	-1.071328	-0.087235
C	-0.264284	-0.647099	-0.020078
C	-0.265788	0.740216	0.070400
C	-1.627857	1.164590	0.070812
N	0.874846	-1.398639	0.004999
C	2.116794	-0.751037	0.041589
C	2.093094	0.685464	-0.279657
N	0.940596	1.402807	0.088299
H	-3.463910	-0.091174	-0.048891
H	-1.869296	-2.030399	-0.128628
H	-1.993372	2.177700	0.121915
H	0.820726	-2.377572	0.246858
H	2.837506	-1.193461	0.727215
H	3.014147	1.245701	-0.199966
H	0.949087	2.411788	0.064788

---

2 (X = NH X' = NH X'' = O)
S0

E = -416.865216036 A.U.
G = -416.778577 A.U.

C	2.376064	0.027575	0.016606
C	0.255062	-0.621585	0.003537
C	0.243055	0.744060	-0.019624
C	1.602127	1.165613	-0.015181
C	-2.002779	-0.741673	0.022007
C	-2.083301	0.588569	0.014314
N	-0.980669	1.470351	-0.105209
H	3.444667	-0.098608	0.040890
H	1.964713	2.181290	-0.025934
H	-2.866908	-1.386185	0.047465
H	-3.060585	1.049357	0.059096
H	-1.036552	2.233782	0.560255
O	-0.813381	-1.470586	-0.030201
N	1.537983	-1.064347	0.019205
H	1.819148	-2.032334	0.031911

T1
E = -416.770512665 A.U.
G = -416.688153 A.U.

C	-2.367753	0.030071	0.004148
C	-0.249472	-0.634249	-0.060545
C	-0.241689	0.747577	0.046552
C	-1.606007	1.168656	0.083459
C	2.006072	-0.756455	0.268050
C	2.097969	0.619705	-0.254869
N	0.968313	1.394791	0.035645
H	-3.437576	-0.090993	-0.011526
H	-1.973828	2.179690	0.147219
H	2.849744	-1.426458	0.186525
H	3.047724	1.130300	-0.191822
H	0.998149	2.400069	-0.055466
O	0.814114	-1.436621	-0.021132
N	-1.539802	-1.068924	-0.078875
H	-1.831430	-2.032535	-0.124030

---

2 (X = NH X' = NH X'' = S)
S0
E = -739.849790753 A.U.
G = -739.767296 A.U.

C	2.554358	-0.146030	0.197050
C	0.383619	-0.565222	-0.097201
C	0.552276	0.791802	-0.192093
C	1.933059	1.068167	-0.000872
C	-2.130498	-0.001610	0.376952
C	-1.749670	1.267825	0.215256
N	-0.539025	1.673483	-0.351482
H	3.592587	-0.385493	0.355096
H	2.407656	2.036825	-0.006541
H	-3.065713	-0.254779	0.853158
H	-2.399290	2.069525	0.546117
H	-0.314980	2.645721	-0.199698
S	-1.170185	-1.393389	-0.214028
N	1.604896	-1.129218	0.156228
H	1.782735	-2.117019	0.248545

T1

E = -739.758371018 A.U.

G = -739.680721 A.U.

C	2.568514	-0.130695	0.052416
C	0.376906	-0.562877	-0.035251
C	0.532936	0.818614	0.009219
C	1.934691	1.085988	0.071182
C	-2.147805	-0.049675	0.393168
C	-1.848864	1.299626	-0.052747
N	-0.519169	1.697388	-0.062090
H	3.618433	-0.370061	0.086019
H	2.405085	2.055510	0.110433
H	-3.179866	-0.351299	0.511643
H	-2.518081	1.881074	-0.680233
H	-0.307321	2.660040	-0.281842
S	-1.131929	-1.408802	-0.114992
N	1.629351	-1.124163	-0.015761
H	1.823083	-2.112892	0.010883

2 (X = NH X' = O X'' = NH)

S0

E = -416.865490745 A.U.

G = -416.778696 A.U.

C	-2.375154	-0.028491	-0.016801
C	-0.248036	0.650806	0.013285
C	-0.252004	-0.716764	0.031166
C	-1.596014	-1.163952	0.009303
C	2.056309	0.637849	-0.031926
C	2.032861	-0.694351	-0.013905
H	-3.445146	0.089035	-0.018159
H	-1.943527	-2.184218	0.019121
H	3.007625	1.149528	-0.065994
H	2.935792	-1.285374	-0.049815
O	0.899442	-1.482464	0.021577
N	0.896891	1.473208	-0.061724
H	0.929451	2.170183	0.677791
N	-1.545739	1.066900	-0.028646
H	-1.845573	2.029222	-0.049697

T1

E = -416.769846866 A.U.

G = -416.687789 A.U.

C	-2.369516	-0.025995	-0.019908
C	-0.244009	0.653898	-0.025723
C	-0.251263	-0.724878	0.081600
C	-1.601586	-1.161012	0.085385
C	2.109784	0.696266	0.068359
C	2.013027	-0.722136	-0.308935
H	-3.439062	0.093205	-0.048885
H	-1.958528	-2.175874	0.146764
H	2.827489	1.067846	0.796597
H	2.896573	-1.343582	-0.298845
O	0.894634	-1.452939	0.090814
N	0.906132	1.395697	-0.004431
H	0.873777	2.366017	0.275710
N	-1.541542	1.073807	-0.100184
H	-1.848070	2.032507	-0.150215

2 (X = NH X' = O X'' = O)

S0

E = -436.722268061 A.U.

G = -436.648307 A.U.

C	-2.355331	0.027305	0.000034
C	-0.238073	-0.633804	-0.000028
C	-0.233935	0.729410	-0.000068
C	-1.581741	1.166694	-0.000111
C	2.000983	-0.689200	0.000136
C	2.030186	0.641090	0.000079
H	-3.424400	-0.095308	0.000079
H	-1.935564	2.184637	-0.000285
H	2.900315	-1.283597	0.000241
H	2.967144	1.176876	0.000133
O	0.933019	1.474842	-0.000071
O	0.844955	-1.464458	0.000063
N	-1.522195	-1.068451	-0.000103
H	-1.808452	-2.035489	0.000356

T1

E = -436.618214417 A.U.

G = -436.548411 A.U.

C	-2.344847	0.027305	0.002764
C	-0.230684	-0.638430	-0.060422
C	-0.227736	0.731067	0.059091
C	-1.579072	1.165160	0.095987
C	1.989470	-0.709104	0.281917
C	2.018028	0.664825	-0.280322
H	-3.414479	-0.092310	-0.014768
H	-1.938248	2.178240	0.167095
H	2.874153	-1.324193	0.320767
H	2.938265	1.225493	-0.330173
O	0.928861	1.447813	0.030916
O	0.849831	-1.435586	-0.024776
N	-1.518337	-1.070842	-0.092268
H	-1.811827	-2.034094	-0.140254

2 (X = NH X' = O X'' = S)

S0

E = -759.705109649 A.U.

G = -759.636624 A.U.

C	-2.556149	-0.157456	0.048584
C	-0.358159	-0.553536	-0.025371
C	-0.542369	0.803430	-0.044014
C	-1.932204	1.068286	0.004173
C	2.155243	0.051795	0.088169
C	1.726420	1.309305	0.049852
H	-3.601156	-0.413790	0.087132
H	-2.402899	2.037849	0.004549
H	3.212804	-0.135423	0.204631
H	2.421795	2.133949	0.129840
O	0.443529	1.763892	-0.085106
S	1.175103	-1.426359	-0.048531
N	-1.594842	-1.131342	0.034836
H	-1.773214	-2.123543	0.058997

T1
E = -759.606962102 A.U.
G = -759.542047 A.U.

C	-2.544035	-0.181019	0.057834
C	-0.346471	-0.557622	-0.074550
C	-0.539934	0.804500	0.014983
C	-1.933582	1.050420	0.104824
C	2.102345	0.028545	0.412277
C	1.736055	1.334602	-0.134761
H	-3.587731	-0.445077	0.088950
H	-2.415176	2.011978	0.173688
H	3.117114	-0.177114	0.719914
H	2.283310	1.832837	-0.929143
O	0.431195	1.756088	-0.062428
S	1.196451	-1.373424	-0.087529
N	-1.583167	-1.147463	-0.053609
H	-1.754393	-2.140863	-0.061899

---

2 (X = NH X' = S X'' = NH)
S0
E = -739.851150761 A.U.
G = -739.768933 A.U.

C	-2.560318	-0.224539	0.181986
C	-0.557067	0.696939	-0.175461
C	-0.369236	-0.656398	-0.065214
C	-1.644564	-1.246315	0.173380
C	1.700048	1.314259	0.205958
C	2.150092	0.066497	0.339267
H	-3.630790	-0.233347	0.297132
H	-1.858095	-2.295614	0.300001
H	2.308709	2.152377	0.521243
H	3.119159	-0.128306	0.774779
S	1.246592	-1.376672	-0.200707
N	0.447156	1.660113	-0.329736
H	0.165614	2.612292	-0.144030
N	-1.883634	0.960171	-0.021303
H	-2.308456	1.874692	-0.040037

T1
E = -739.761122483 A.U.
G = -739.683445 A.U.

C	2.569419	-0.210794	0.057831
C	0.537718	0.728161	-0.001690
C	0.367701	-0.652698	-0.042307
C	1.669093	-1.239013	-0.010660
C	-1.793621	1.346250	-0.039673
C	-2.152014	0.004222	0.395959
H	3.645017	-0.220784	0.109022
H	1.901290	-2.291674	-0.021702
H	-2.427816	1.955630	-0.677166
H	-3.197445	-0.239479	0.529398
S	-1.206188	-1.385779	-0.116229
N	-0.440431	1.671783	-0.074288
H	-0.179505	2.618822	-0.312721
N	1.878668	0.984440	0.070194
H	2.300028	1.899626	0.104731

2 (X = NH X' = S X'' = O)
S0
E = -759.708016428 A.U.
G = -759.639550 A.U.

C	2.558829	-0.229228	0.000024
C	0.542341	0.703019	-0.000012
C	0.347598	-0.649342	-0.000010
C	1.640584	-1.249666	0.000012
C	-1.671839	1.355397	0.000017
C	-2.161303	0.120677	0.000023
H	3.635060	-0.247319	0.000038
H	1.859816	-2.305274	0.000007
H	-2.314549	2.223808	0.000047
H	-3.235163	-0.002799	0.000060
S	-1.256601	-1.404683	-0.000026
O	-0.345937	1.733977	-0.000025
N	1.873246	0.963157	0.000013
H	2.277971	1.887457	0.000048

T1
E = -759.610685382 A.U.
G = -759.545936 A.U.

C	-2.547263	-0.241142	0.019081
C	-0.537500	0.711059	0.049253
C	-0.337703	-0.648870	-0.063210
C	-1.632615	-1.254140	-0.091915
C	1.649011	1.329572	-0.312667
C	2.164560	0.077981	0.238238
H	-3.623064	-0.267525	0.052731
H	-1.850667	-2.307553	-0.158589
H	2.276349	2.193554	-0.468740
H	3.046046	-0.000984	0.859042
S	1.260262	-1.358741	-0.026806
O	0.353152	1.712476	0.025484
N	-1.871749	0.957173	0.108098
H	-2.286771	1.875592	0.151211

2 (X = NH X' = S X'' = S)
S0
E = -1082.68784577 A.U.
G = -1082.621883 A.U.

C	-2.678695	0.060089	0.298352
C	-0.611555	-0.631357	-0.176806
C	-0.591950	0.741337	-0.162382
C	-1.905967	1.187759	0.152076
C	1.956202	-0.713220	0.561996
C	2.006304	0.614432	0.549291
H	-3.730197	-0.049710	0.505914
H	-2.237954	2.209023	0.246793
H	2.658784	-1.296245	1.144481
H	2.759492	1.142673	1.121635
S	0.780958	-1.686823	-0.358192
S	0.896882	1.668537	-0.354605
N	-1.880050	-1.038430	0.122365
H	-2.181232	-1.998405	0.194221

T1

E = -1082.59403335 A.U.

G = -1082.535151 A.U.

C	-2.708811	0.067260	-0.000612
C	-0.586783	-0.639511	-0.040167
C	-0.565577	0.740367	0.054843
C	-1.920796	1.184439	0.093689
C	2.050815	-0.709843	0.302507
C	2.097793	0.610579	-0.298328
H	-3.780787	-0.037422	-0.017358
H	-2.260162	2.205930	0.156856
H	2.891063	-1.171038	0.802736
H	2.977429	1.007786	-0.785942
S	0.735359	-1.783707	-0.034446
S	0.855997	1.764273	0.029449
N	-1.896328	-1.032170	-0.089368
H	-2.214780	-1.988869	-0.122366

---

2 (X = o X' = NH X'' = NH)

S0

E = -416.859012540 A.U.

G = -416.772069 A.U.

C	2.358155	-0.032504	0.002505
C	0.266210	-0.620073	0.016073
C	0.268532	0.732135	0.008267
C	1.648090	1.123138	-0.009194
N	-0.840820	-1.470238	-0.095154
C	-2.041596	-0.687485	-0.003556
C	-2.072732	0.646580	0.007188
N	-0.929678	1.487579	-0.089508
H	3.410106	-0.256054	0.028898
H	2.049662	2.123730	-0.012744
H	-0.828085	-2.222885	0.587874
H	-2.958669	-1.258873	-0.001178
H	-3.023482	1.161929	0.026823
H	-0.970036	2.262743	0.562978
O	1.519254	-1.112840	-0.003465

T1

E = -416.771160987 A.U.

G = -416.689850 A.U.

C	-2.357664	-0.034668	-0.024661
C	-0.265413	-0.627144	-0.020907
C	-0.274821	0.746579	0.068112
C	-1.658651	1.123007	0.063986
N	0.849192	-1.390369	0.012935
C	2.091632	-0.748978	0.037272
C	2.078000	0.691444	-0.260464
N	0.922427	1.415153	0.085332
H	-3.409307	-0.257618	-0.061255
H	-2.073724	2.116450	0.116153
H	0.774755	-2.383644	0.183292
H	2.843152	-1.225989	0.658596
H	3.000526	1.246833	-0.176565
H	0.934228	2.424249	0.070507
O	-1.518682	-1.124401	-0.082328

---

2 (X = O X' = NH X'' = O)

S0

E = -436.717211049 A.U.

G = -436.642794 A.U.

C	2.338139	-0.033815	0.005996
C	0.256620	-0.606691	0.015629
C	0.251058	0.741926	-0.006687
C	1.633299	1.124649	-0.017609
C	-1.985454	-0.735253	0.011718
C	-2.066090	0.594063	0.001418
N	-0.960406	1.478343	-0.096463
H	3.388144	-0.265088	0.022948
H	2.039668	2.122951	-0.031571
H	-2.845740	-1.384278	0.020399
H	-3.043952	1.054535	0.025802
H	-1.019491	2.237810	0.573328
O	-0.787583	-1.464528	-0.015820
O	1.492430	-1.113423	0.016013

T1

E = -436.622659802 A.U.

G = -436.552677 A.U.

C	-2.330670	-0.030898	0.007793
C	-0.251234	-0.617531	-0.078160
C	-0.249481	0.746970	0.040752
C	-1.634403	1.127215	0.089205
C	1.983451	-0.754380	0.272599
C	2.078989	0.625677	-0.238830
N	0.951965	1.403895	0.032587
H	-3.383067	-0.253090	-0.010713
H	-2.046505	2.119800	0.165965
H	2.826521	-1.425512	0.211337
H	3.030876	1.132778	-0.219694
H	0.982128	2.409982	-0.050060
O	0.790443	-1.431322	-0.025092
O	-1.497145	-1.117870	-0.085545

---

2 (X = O X' = NH X'' = S)

S0

E = -759.699231459 A.U.

G = -759.629347 A.U.

C	2.506597	-0.208746	0.190543
C	0.379349	-0.544603	-0.094388
C	0.558157	0.794567	-0.176426
C	1.960684	1.020926	0.015161
C	-2.118423	0.010199	0.345253
C	-1.736433	1.278859	0.190588
N	-0.507920	1.692926	-0.338443
H	3.515199	-0.550351	0.347359
H	2.480878	1.965218	0.015676
H	-3.069004	-0.237642	0.792771
H	-2.398551	2.078750	0.498705
H	-0.279807	2.661661	-0.171923
S	-1.150871	-1.404004	-0.191009
O	1.552634	-1.176408	0.139783

T1
E = -759.608837196 A.U.
G = -759.543632 A.U.

C	2.519522	-0.192404	0.053293
C	0.372590	-0.544702	-0.048365
C	0.538849	0.818675	0.003638
C	1.959312	1.037760	0.075074
C	-2.129967	-0.042870	0.382395
C	-1.821968	1.304452	-0.065360
N	-0.496227	1.708763	-0.054330
H	3.541413	-0.530100	0.080876
H	2.474315	1.983340	0.125996
H	-3.163532	-0.338247	0.499075
H	-2.502032	1.913497	-0.649619
H	-0.280147	2.675994	-0.249327
S	-1.115200	-1.410384	-0.105964
O	1.577093	-1.173144	-0.016915

---

2 (X = O X' = O X'' = NH)
S0
E = -436.716565988 A.U.
G = -436.642191 A.U.

C	2.337226	-0.034098	-0.017209
C	0.248117	-0.634072	0.015907
C	0.259014	0.715869	0.028009
C	1.627720	1.122404	0.002029
C	-2.037391	-0.631254	-0.031998
C	-2.016030	0.700160	-0.008280
H	3.389870	-0.254877	-0.016739
H	2.020950	2.125487	0.008005
H	-2.986621	-1.145759	-0.066503
H	-2.917399	1.292981	-0.036483
O	-0.877808	1.491372	0.024569
N	-0.873554	-1.465792	-0.069565
H	-0.895935	-2.182375	0.651558
O	1.501818	-1.117494	-0.022523

T1
E = -436.623342335 A.U.
G = -436.554262 A.U.

C	2.303798	0.049390	-0.173601
C	0.280257	-0.688746	0.452465
C	0.236329	0.767155	0.168513
C	1.497404	1.179594	-0.192552
C	-1.978913	-0.700752	-0.210267
C	-2.048386	0.631290	-0.014947
H	3.327127	-0.105607	-0.467592
H	1.812256	2.173745	-0.467599
H	-2.870140	-1.283229	-0.383187
H	-2.966802	1.194061	-0.016447
O	-0.940887	1.424854	0.142966
N	-0.773104	-1.370820	-0.132765
H	-0.741021	-2.374473	-0.246842
O	1.579308	-1.104398	0.148703

---

2 (X = O X' = O X'' = O)
S0
E = -456.572279060 A.U.
G = -456.510617 A.U.

C	-2.317697	-0.035615	0.000235
C	-0.239243	-0.618976	-0.000214
C	-0.241931	0.727152	-0.000064
C	-1.613826	1.124647	-0.000179
C	1.983181	-0.681545	0.000193
C	2.012533	0.647949	0.000278
H	-3.368130	-0.264581	0.000405
H	-2.012747	2.125365	-0.000200
H	2.880018	-1.278838	0.000352
H	2.947723	1.186004	0.000510
O	0.910639	1.482895	0.000062
O	0.820812	-1.458201	-0.000172
O	-1.474572	-1.118397	-0.000211

T1
E = -456.474925894 A.U.
G = -456.417899 A.U.

C	-2.266304	0.050685	-0.192787
C	-0.261579	-0.680391	0.469462
C	-0.214927	0.784723	0.183245
C	-1.460034	1.180971	-0.235951
C	1.901023	-0.747698	-0.237984
C	2.035174	0.561137	-0.007172
H	-3.275734	-0.124082	-0.521166
H	-1.768936	2.160348	-0.564566
H	2.741538	-1.392821	-0.435852
H	2.989019	1.062637	0.005266
O	0.974869	1.414203	0.178360
O	0.692715	-1.405441	-0.194487
O	-1.553335	-1.084092	0.221558

---

2 (X = O X' = O X'' = S)
S0
E = -779.553314745 A.U.
G = -779.496982 A.U.

C	-2.506679	-0.222993	0.000103
C	-0.352374	-0.533542	-0.000071
C	-0.548495	0.803870	-0.000070
C	-1.960883	1.017483	0.000027
C	2.136109	0.062077	0.000143
C	1.707205	1.319437	0.000087
H	-3.520163	-0.584107	0.000164
H	-2.478279	1.962522	0.000054
H	3.200751	-0.120932	0.000339
H	2.405682	2.144579	0.000225
O	0.412504	1.777964	-0.000135
S	1.158309	-1.429621	-0.000087
O	-1.536285	-1.178729	0.000048

T1
E = -779.455474329 A.U.
G = -779.402922 A.U.

C	-2.496650	-0.240030	0.014080

C	-0.342094	-0.537331	-0.058834
C	-0.547032	0.804181	0.059792
C	-1.962952	1.000454	0.115757
C	2.137212	0.030298	0.236879
C	1.668334	1.304255	-0.314863
H	-3.509006	-0.604647	-0.004011
H	-2.491951	1.934749	0.203659
H	2.981526	-0.078369	0.903740
H	2.342772	2.120710	-0.522200
O	0.409826	1.759333	0.038070
S	1.178926	-1.381040	-0.027115
O	-1.525709	-1.190179	-0.096097

---

2 (X = O X' = S X'' = NH)
S0
E = -759.703527213 A.U.
G = -759.633794 A.U.

C	-2.528727	-0.165469	0.178105
C	-0.555362	0.672996	-0.179048
C	-0.371048	-0.663024	-0.068264
C	-1.673868	-1.214770	0.180071
C	1.675287	1.321876	0.198688
C	2.132992	0.077355	0.327674
H	-3.594987	-0.071839	0.283838
H	-1.931110	-2.252292	0.318216
H	2.278573	2.165086	0.508663
H	3.106908	-0.109260	0.755743
S	1.241750	-1.385543	-0.191556
N	0.409669	1.657063	-0.322411
H	0.092218	2.596859	-0.128728
O	-1.845364	1.008364	-0.029913

T1
E = -759.614836423 A.U.
G = -759.549328 A.U.

C	-2.538914	-0.148888	0.057290
C	-0.534922	0.705660	0.001645
C	-0.370812	-0.660655	-0.035113
C	-1.699214	-1.205929	-0.003577
C	1.765237	1.349116	-0.043178
C	2.137255	0.007774	0.380410
H	-3.609396	-0.055680	0.106734
H	-1.978323	-2.246839	-0.015747
H	2.405530	1.998761	-0.628679
H	3.184757	-0.227435	0.509295
S	1.198086	-1.393242	-0.115558
N	0.411386	1.661682	-0.073979
H	0.117227	2.610328	-0.267816
O	-1.840081	1.037312	0.064768

---

2 (X = O X' = S X'' = O)
S0
E = -779.558789948 A.U.
G = -779.503284 A.U.

C	2.526663	-0.169504	0.000394
C	0.541813	0.688742	-0.000398

C	0.351121	-0.646606	-0.000172
C	1.669736	-1.216640	0.000381
C	-1.652247	1.353656	0.000452
C	-2.143833	0.121051	0.000712
H	3.598617	-0.085212	0.000632
H	1.929429	-2.262412	0.000677
H	-2.288472	2.225972	0.001161
H	-3.217867	0.000653	0.001695
S	-1.245618	-1.411111	-0.000397
O	-0.318273	1.725594	-0.000714
O	1.836857	1.013728	-0.000040

T1
E = -779.462045689 A.U.
G = -779.409772 A.U.

C	2.517050	-0.182969	0.026512
C	0.537215	0.695590	0.049216
C	0.341203	-0.647713	-0.060502
C	1.661022	-1.222137	-0.085069
C	-1.629848	1.329546	-0.303124
C	-2.137732	0.079674	0.260114
H	3.589063	-0.108428	0.072341
H	1.919814	-2.265585	-0.157040
H	-2.251241	2.193963	-0.473674
H	-3.050225	-0.007472	0.831079
S	-1.252582	-1.363690	-0.038035
O	-0.324697	1.704642	0.018976
O	1.837251	1.007186	0.107645

---

2 (X = O X' = S X'' = S)
S0
E = -1102.53550104 A.U.
G = -1102.482222 A.U.

C	-2.638261	0.004860	0.292284
C	-0.606605	-0.612404	-0.172599
C	-0.590263	0.742085	-0.151377
C	-1.928720	1.151768	0.167709
C	1.949987	-0.713238	0.534238
C	2.005420	0.613114	0.517359
H	-3.675865	-0.203029	0.490322
H	-2.299739	2.158102	0.271965
H	2.666491	-1.294964	1.100511
H	2.776553	1.136473	1.069620
S	0.750719	-1.703848	-0.339065
S	0.880701	1.688743	-0.344609
O	-1.839939	-1.084000	0.110085

T1
E = -1102.44330500 A.U.
G = -1102.396192 A.U.

C	-2.665804	0.010826	0.011026
C	-0.579363	-0.617291	0.039644
C	-0.564066	0.743277	-0.057693
C	-1.944170	1.148418	-0.090931
C	2.032488	-0.715481	-0.285293
C	2.074681	0.606528	0.315580
H	-3.722075	-0.193177	0.044587

H	-2.325546	2.154008	-0.161180
H	2.864425	-1.163638	-0.810053
H	2.954741	1.002812	0.802723
S	0.707848	-1.788447	0.026637
S	0.848643	1.773654	-0.040793
O	-1.849751	-1.077623	0.094553

---

2 (X = S X' = NH X'' = NH)
S0
E = -739.838568821 A.U.
G = -739.756343 A.U.

C	2.229082	0.708434	0.047650
C	0.088951	-0.526832	-0.023802
C	-0.084678	0.824366	-0.034294
C	1.151924	1.537050	0.008343
N	-0.956328	-1.472746	-0.138412
C	-2.232116	-0.854740	0.051404
C	-2.418712	0.463949	0.053636
N	-1.375335	1.408220	-0.143764
H	3.274673	0.964722	0.105565
H	1.221162	2.616735	0.016322
H	-0.832607	-2.268537	0.478783
H	-3.065457	-1.536890	0.138493
H	-3.412582	0.878895	0.145099
H	-1.483257	2.230326	0.439062
S	1.763313	-0.959183	0.002143

T1
E = -739.766686008 A.U.
G = -739.688623 A.U.

C	-2.198619	0.772575	0.157030
C	-0.101479	-0.565536	-0.339986
C	0.099944	0.868218	-0.120970
C	-1.068986	1.576959	0.107269
N	0.927259	-1.392199	0.100600
C	2.207124	-0.906377	0.167687
C	2.444075	0.426370	0.006001
N	1.400370	1.313219	-0.123465
H	-3.209564	1.061678	0.393430
H	-1.102648	2.649770	0.248790
H	0.779175	-2.388925	0.167124
H	3.003663	-1.618448	0.312898
H	3.440069	0.838488	0.019839
H	1.588665	2.304196	-0.076956
S	-1.817820	-0.957947	-0.047954

---

2 (X = S X' = NH X'' = O)
S0
E = -759.695655221 A.U.
G = -759.626202 A.U.

C	2.207548	0.706084	0.045531
C	0.081768	-0.511113	-0.042676
C	-0.103687	0.835077	-0.054064
C	1.135553	1.541931	-0.004824
C	-2.164185	-0.899696	0.067426
C	-2.403021	0.408038	0.062991

N	-1.405588	1.392736	-0.142779
H	3.254511	0.960064	0.084854
H	1.211706	2.620822	-0.009180
H	-2.931869	-1.649782	0.168622
H	-3.415515	0.765531	0.183796
H	-1.530741	2.204276	0.451263
O	-0.897531	-1.466446	-0.090511
S	1.744214	-0.962526	0.024868

T1
E = -759.613527325 A.U.
G = -759.547740 A.U.

C	-2.167298	0.767530	0.203950
C	-0.082997	-0.563804	-0.353155
C	0.118271	0.881530	-0.128713
C	-1.048545	1.578212	0.151808
C	2.129832	-0.937800	0.205001
C	2.420409	0.360624	-0.023715
N	1.421148	1.294837	-0.172226
H	-3.166772	1.041808	0.500956
H	-1.084783	2.644829	0.331638
H	2.871128	-1.700011	0.379698
H	3.439414	0.713171	-0.047556
H	1.651009	2.278300	-0.135355
O	0.852940	-1.413886	0.185419
S	-1.793724	-0.953039	-0.102388

---

2 (X = S X' = NH X'' = S)
S0
E = -1082.68379654 A.U.
G = -1082.617841 A.U.

C	-2.458467	0.582771	0.130537
C	-0.224923	-0.431495	-0.126096
C	-0.195809	0.929545	-0.208761
C	-1.489232	1.518677	-0.057780
C	2.339931	-0.228948	0.433917
C	2.140074	1.080123	0.284711
N	1.017189	1.628245	-0.348447
H	-3.519284	0.737660	0.251769
H	-1.677052	2.583315	-0.096057
H	3.183114	-0.623556	0.980634
H	2.848191	1.796176	0.683696
H	0.934129	2.630131	-0.255680
S	1.225546	-1.429772	-0.282446
S	-1.822975	-1.021820	0.165921

T1
E = -1082.60139329 A.U.
G = -1082.539438 A.U.

C	2.423178	0.663896	0.247932
C	0.214218	-0.442327	-0.357334
C	0.153004	0.997754	-0.074688
C	1.385925	1.566344	0.248354
C	-2.407867	-0.355958	0.040757
C	-2.260329	0.984119	-0.137565
N	-1.061529	1.632276	-0.136612
H	3.454082	0.832849	0.513892

H	1.515158	2.615511	0.483080
H	-3.388004	-0.807569	0.058373
H	-3.128004	1.617886	-0.254806
H	-1.074633	2.643103	-0.111522
S	-1.084676	-1.451855	0.241814
S	1.897384	-0.973813	-0.212906

---

2 (X = S X' = O X'' = NH)

S0

E = -759.697605693 A.U.

G = -759.628036 A.U.

C	2.210460	0.707731	0.005604
C	0.075914	-0.540973	0.014955
C	-0.089409	0.809347	0.017477
C	1.133032	1.536978	0.014404
C	-2.235184	-0.803257	-0.015517
C	-2.371452	0.518820	-0.005872
H	3.256910	0.965806	0.026886
H	1.187038	2.616365	0.029258
H	-3.111585	-1.434983	-0.027269
H	-3.331801	1.010276	-0.026125
O	-1.322932	1.425021	0.000180
N	-0.979676	-1.474724	-0.065570
H	-0.915348	-2.213430	0.627598
S	1.750988	-0.962063	-0.022445

T1

E = -759.612437928 A.U.

G = -759.547049 A.U.

C	-2.142559	0.793241	0.234510
C	-0.096657	-0.588818	-0.368094
C	0.128108	0.850461	-0.145402
C	-0.993953	1.579131	0.162736
C	2.174065	-0.875182	0.227143
C	2.398999	0.433409	-0.019243
H	-3.122878	1.092465	0.569063
H	-0.983967	2.643197	0.355607
H	2.983569	-1.556268	0.436801
H	3.376106	0.886591	-0.035932
O	1.391361	1.337590	-0.198479
N	0.901296	-1.388108	0.157628
H	0.749820	-2.374576	0.315925
S	-1.828164	-0.926801	-0.106683

---

2 (X = S X' = O X'' = O)

S0

E = -779.552324196 A.U.

G = -779.495378 A.U.

C	-2.188655	0.707365	0.000515
C	-0.069348	-0.523948	-0.000157
C	0.109578	0.820653	-0.000166
C	-1.115442	1.542867	-0.000074
C	2.168677	-0.850894	0.000583
C	2.360161	0.462142	0.000540
H	-3.235982	0.962272	0.000972
H	-1.176007	2.621726	0.000060

H	2.982456	-1.557877	0.001022
H	3.348178	0.894826	0.000973
O	1.355398	1.411444	-0.000038
O	0.922471	-1.466159	-0.000028
S	-1.733214	-0.964522	-0.000622

T1
E = -779.458083911 A.U.
G = -779.404900 A.U.

C	-2.101902	0.793007	0.277450
C	-0.083553	-0.589372	-0.370903
C	0.153334	0.863220	-0.159716
C	-0.952432	1.582462	0.203935
C	2.090154	-0.911273	0.264289
C	2.377033	0.357142	-0.039455
H	-3.056813	1.076883	0.691110
H	-0.937726	2.637402	0.440343
H	2.841842	-1.645969	0.502175
H	3.382747	0.741860	-0.084950
O	1.422661	1.314466	-0.258360
O	0.812986	-1.413391	0.248898
S	-1.813190	-0.911869	-0.157911

---

2 (X = S X' = O X'' = S)
S0
E = -1102.53747421 A.U.
G = -1102.484778 A.U.

C	-2.451428	0.583131	0.100594
C	-0.207767	-0.431549	-0.096311
C	-0.190183	0.927515	-0.149502
C	-1.478182	1.522840	-0.031310
C	2.357772	-0.169434	0.310441
C	2.107554	1.128156	0.204110
H	-3.516210	0.729758	0.190406
H	-1.646707	2.590016	-0.056172
H	3.298242	-0.512154	0.717037
H	2.824658	1.882057	0.499569
O	0.945601	1.692003	-0.271331
S	-1.815516	-1.023201	0.117355
S	1.231055	-1.451153	-0.192875

T1
E = -1102.44423846 A.U.
G = -1102.395309 A.U.

C	2.356319	0.690859	0.329116
C	0.209008	-0.454814	-0.401170
C	0.112566	0.988849	-0.095286
C	1.288508	1.574419	0.321093
C	-2.364545	-0.341441	0.087762
C	-2.225318	0.967361	-0.167568
H	3.352597	0.861727	0.704458
H	1.363877	2.611204	0.619675
H	-3.351124	-0.776165	0.149050
H	-3.072567	1.618803	-0.324668
O	-1.059805	1.653922	-0.214797
S	1.911717	-0.924727	-0.274188
S	-1.041315	-1.456420	0.282074

---

2 (X = S X' = S X'' = NH)
S0
E = -1082.68269952 A.U.
G = -1082.616984 A.U.

C	-2.333317	-0.871050	-0.224264
C	-0.371948	0.575702	0.216396
C	-0.043328	-0.740327	0.088874
C	-1.172346	-1.574166	-0.175822
C	1.798267	1.429756	-0.264795
C	2.373193	0.240189	-0.425681
H	-3.339139	-1.236553	-0.357396
H	-1.113224	-2.647110	-0.296030
H	2.256932	2.333220	-0.646912
H	3.299583	0.122849	-0.967934
S	1.653424	-1.248199	0.250337
N	0.559040	1.603493	0.376514
H	0.191376	2.542813	0.346969
S	-2.072920	0.829431	-0.000496

T1
E = -1082.59754099 A.U.
G = -1082.535874 A.U.

C	2.286577	-0.920676	0.277058
C	0.378736	0.624414	-0.353131
C	-0.002083	-0.766186	-0.059340
C	1.076968	-1.579712	0.275246
C	-1.796282	1.467426	0.263660
C	-2.441781	0.285727	0.079754
H	3.251917	-1.316503	0.550708
H	0.983762	-2.629977	0.520430
H	-2.351376	2.366227	0.492751
H	-3.519523	0.232355	0.119019
S	-1.656391	-1.240716	-0.185606
N	-0.449788	1.623970	0.131217
H	-0.069911	2.553124	0.255330
S	2.146443	0.788280	-0.174159

---

2 (X = S X' = S X'' = O)
S0
E = -1102.53571942 A.U.
G = -1102.482907 A.U.

C	-2.319018	0.874451	0.173698
C	-0.364084	-0.570084	-0.170629
C	-0.021956	0.739453	-0.081306
C	-1.159393	1.580711	0.123273
C	1.738401	-1.469485	0.200664
C	2.362044	-0.306668	0.319022
H	-3.324518	1.247050	0.288582
H	-1.104820	2.656686	0.212397
H	2.177524	-2.411129	0.498713
H	3.355773	-0.267312	0.741729
S	1.673511	1.255854	-0.172855
O	0.464374	-1.641760	-0.314208
S	-2.063194	-0.829697	0.009350

T1
E = -1102.44231204 A.U.
G = -1102.393169 A.U.

C	-2.255477	0.911006	0.314897
C	-0.360391	-0.618623	-0.365355
C	0.025374	0.779015	-0.047995
C	-1.046496	1.574152	0.337376
C	1.705551	-1.482733	0.304467
C	2.389725	-0.355562	0.068809
H	-3.210699	1.285684	0.648869
H	-0.953288	2.609511	0.637914
H	2.192446	-2.411270	0.565034
H	3.469832	-0.366000	0.098145
S	1.681708	1.212825	-0.224513
O	0.358424	-1.628582	0.217785
S	-2.126421	-0.771124	-0.235826

---

2 (X = S X' = S X'' = S)
S0
E = -1425.51864445 A.U.
G = -1425.469508 A.U.

C	-2.521009	0.798859	0.237090
C	-0.455506	-0.487168	-0.185663
C	-0.245392	0.859764	-0.163502
C	-1.436907	1.606404	0.090513
C	2.071703	-0.933531	0.584552
C	2.302266	0.373864	0.593853
H	-3.548252	1.084027	0.404827
H	-1.472489	2.685406	0.145255
H	2.651973	-1.617321	1.191556
H	3.084723	0.801050	1.209143
S	0.801963	-1.699663	-0.398554
S	-2.111296	-0.874722	0.128421
S	1.371403	1.557991	-0.348107

T1
E = -1425.42452526 A.U.
G = -1425.381992 A.U.

C	-2.532297	0.828312	0.044451
C	-0.438802	-0.495835	-0.033621
C	-0.213183	0.854905	0.050034
C	-1.429265	1.612066	0.111792
C	2.177816	-0.951549	0.313218
C	2.417827	0.342536	-0.296793
H	-3.568211	1.129696	0.047779
H	-1.456766	2.691117	0.180781
H	2.931509	-1.516112	0.844544
H	3.322980	0.591983	-0.833128
S	0.719377	-1.811502	-0.022480
S	-2.139250	-0.850342	-0.074829
S	1.349742	1.659388	0.011406

---

3 (X = S X' = S X'' = S)
S0
E = -528.568190331 A.U.
G = -528.442011 A.U.

C	-0.020805	-0.110110	3.282945
N	-1.089529	-0.061530	2.422988
C	-0.630439	0.049083	1.141342
C	0.743594	0.045203	1.170255
C	1.133461	-0.062782	2.536575
N	-1.477514	0.070711	-0.000000
C	-0.630439	0.049083	-1.141342
C	0.743594	0.045203	-1.170255
N	1.559610	0.046623	0.000000
N	-1.089529	-0.061530	-2.422988
C	-0.020805	-0.110110	-3.282945
C	1.133461	-0.062782	-2.536575
H	-0.170490	-0.170643	4.347668
H	-2.060999	-0.111004	2.687075
H	2.141953	-0.092476	2.918665
H	-2.061720	0.905502	-0.000000
H	2.209791	0.826101	0.000000
H	-2.060999	-0.111004	-2.687075
H	-0.170490	-0.170643	-4.347668
H	2.141953	-0.092476	-2.918665

T1
E = -528.467454605 A.U.
G = -528.347122 A.U.

C	-3.285883	-0.040066	-0.075276
N	-2.419059	-1.108334	-0.146553
C	-1.135638	-0.641853	-0.074720
C	-1.185772	0.739334	0.065763
C	-2.559094	1.117165	0.061842
N	0.032202	-1.354446	-0.063750
C	1.170588	-0.671204	0.422701
C	1.177160	0.781069	0.116688
N	-0.012277	1.474720	0.167869
N	2.469981	-1.151066	0.048987
C	3.275339	0.019666	-0.186776
C	2.465677	1.156863	-0.219041
H	-4.349805	-0.194048	-0.136129
H	-2.688237	-2.075412	-0.233574
H	-2.957701	2.115811	0.139745
H	0.028912	-2.363909	-0.111330
H	-0.025417	2.454044	-0.077851
H	2.876611	-1.792525	0.719915
H	4.291134	-0.089044	-0.535339
H	2.794313	2.153124	-0.478382

---

3 (X = NH X' = NH X'' = O)
S0
E = -548.425364955 A.U.
G = -548.311754 A.U.

C	-0.072967	-0.031143	3.255803
C	-0.609239	0.027446	1.105675
C	0.760408	0.000581	1.159175
C	1.102252	-0.038851	2.542463
C	-0.609239	0.027446	-1.105675
C	0.760408	0.000581	-1.159175
N	1.595578	-0.037503	0.000000
C	-0.072967	-0.031143	-3.255803

C	1.102252	-0.038851	-2.542463
H	-0.255551	-0.042233	4.316570
H	2.098019	-0.062346	2.956235
H	2.246938	0.742019	0.000000
H	-0.255551	-0.042233	-4.316570
H	2.098019	-0.062346	-2.956235
O	-1.423806	0.031229	-0.000000
N	-1.119539	-0.000399	-2.363299
H	-2.101185	-0.005484	-2.592654
N	-1.119539	-0.000399	2.363299
H	-2.101185	-0.005484	2.592654

T1
E = -548.324101597 A.U.
G = -548.215570 A.U.

C	3.248828	-0.086374	-0.069003
C	1.091612	-0.618097	-0.093923
C	1.170785	0.750913	0.068054
C	2.554984	1.090068	0.080002
C	-1.124356	-0.656656	0.443659
C	-1.169148	0.797354	0.112086
N	0.005621	1.503923	0.172923
C	-3.233855	-0.034395	-0.194189
C	-2.470841	1.122688	-0.249168
H	4.308241	-0.268822	-0.127795
H	2.981507	2.075357	0.175289
H	0.020737	2.480514	-0.085444
H	-4.247125	-0.193964	-0.527382
H	-2.831207	2.097226	-0.543829
O	-0.024047	-1.369431	-0.098521
N	-2.394027	-1.157462	0.110523
H	-2.781323	-1.863971	0.722296
N	2.352993	-1.126623	-0.161194
H	2.581388	-2.102755	-0.265843

3 (X = NH X' = NH X'' = S)
S0
E = -871.408433899 A.U.
G = -871.298912 A.U.

C	0.315848	0.385926	3.297470
C	-0.525299	-0.052633	1.277745
C	0.840186	-0.191060	1.185246
C	1.377794	0.092675	2.471424
C	-0.525299	-0.052633	-1.277745
C	0.840186	-0.191060	-1.185246
N	1.524454	-0.541432	0.000000
C	0.315848	0.385926	-3.297470
C	1.377794	0.092675	-2.471424
H	0.289413	0.651676	4.340946
H	2.420657	0.085670	2.747685
H	2.486284	-0.228025	0.000000
H	0.289413	0.651676	-4.340946
H	2.420657	0.085670	-2.747685
S	-1.717001	-0.338970	-0.000000
N	-0.834236	0.301256	-2.565590
H	-1.764314	0.465194	-2.917479
N	-0.834236	0.301256	2.565590

H	-1.764314	0.465194	2.917479

T1
E = -871.305846274 A.U.
G = -871.199951 A.U.

C	-0.335750	-0.000258	-3.349104
C	0.502123	0.000085	-1.263630
C	-0.902461	0.000150	-1.189572
C	-1.424712	-0.000155	-2.516774
C	0.502123	0.000085	1.263630
C	-0.902461	0.000150	1.189572
N	-1.575079	0.000293	0.000000
C	-0.335750	-0.000258	3.349104
C	-1.424712	-0.000155	2.516774
H	-0.282152	-0.000358	-4.425603
H	-2.463085	-0.000151	-2.809387
H	-2.586028	0.001116	0.000000
H	-0.282152	-0.000358	4.425603
H	-2.463085	-0.000151	2.809387
S	1.890720	0.000198	-0.000000
N	0.804836	-0.000164	2.585383
H	1.756209	-0.000441	2.931706
N	0.804836	-0.000164	-2.585383
H	1.756209	-0.000441	-2.931706

---

3 (X = NH X' = O X'' = NH)
S0
E = -548.427316021 A.U.
G = -548.313567 A.U.

C	3.250085	0.031730	-0.121487
C	1.128966	-0.648903	0.043430
C	1.132459	0.724113	0.065580
C	2.475177	1.166790	-0.043118
C	-1.128966	-0.648902	0.043426
C	-1.132458	0.724114	0.065573
C	-3.250086	0.031731	-0.121467
C	-2.475172	1.166793	-0.043170
H	4.317576	-0.084570	-0.198929
H	2.822115	2.187218	-0.056741
H	-4.317579	-0.084567	-0.198890
H	-2.822108	2.187221	-0.056830
O	0.000000	1.510832	0.136802
N	-0.000001	-1.512007	0.078272
H	-0.000001	-2.070751	0.931027
N	-2.424007	-1.062295	-0.080684
H	-2.718731	-2.024910	-0.132382
N	2.424004	-1.062296	-0.080708
H	2.718727	-2.024910	-0.132426

T1
E = -548.312590122 A.U.
G = -548.205165 A.U.

C	3.231503	-0.043735	0.124877
C	1.080583	-0.645433	0.098577
C	1.145997	0.719600	-0.095309
C	2.509103	1.108127	-0.078132
C	-1.153352	-0.661063	-0.553492

C	-1.123101	0.796137	-0.183772
C	-3.164131	0.046833	0.343244
C	-2.330662	1.164735	0.364537
H	4.292833	-0.195914	0.220935
H	2.902366	2.105255	-0.188272
H	-4.153249	-0.073561	0.753321
H	-2.591265	2.138820	0.749847
O	0.034113	1.511229	-0.233939
N	-0.096554	-1.367942	0.060405
H	-0.063920	-2.378052	0.009608
N	-2.517286	-1.035175	-0.312648
H	-2.689326	-1.971266	0.032322
N	2.360579	-1.105464	0.223607
H	2.626833	-2.066249	0.371034

3 (X = NH X' = O X'' = O)
S0
E = -568.281725883 A.U.
G = -568.180911 A.U.

C	3.229779	-0.021551	0.000107
C	1.095871	-0.626711	-0.000154
C	1.123734	0.742550	-0.000015
C	2.486807	1.136493	-0.000291
C	-1.095871	-0.626713	-0.000192
C	-1.123735	0.742551	0.000045
C	-3.229778	-0.021551	0.000095
C	-2.486807	1.136496	0.000216
H	4.295333	-0.172410	0.000267
H	2.866368	2.145159	-0.000418
H	-4.295331	-0.172409	-0.000022
H	-2.866368	2.145162	0.000324
O	0.000000	-1.456101	-0.000310
O	0.000000	1.548364	0.000034
N	-2.369341	-1.094022	0.000184
H	-2.629526	-2.068351	0.000234
N	2.369340	-1.094020	0.000201
H	2.629521	-2.068350	0.000278

T1
E = -568.172858465 A.U.
G = -568.076673 A.U.

C	3.182808	0.010978	-0.227351
C	1.112502	-0.669326	0.464665
C	1.118222	0.793152	0.116822
C	2.373121	1.152387	-0.296063
C	-1.064099	-0.626053	-0.100072
C	-1.133973	0.732117	0.081486
C	-3.212710	-0.063775	-0.080964
C	-2.502573	1.101907	0.090630
H	4.187405	-0.119601	-0.597210
H	2.686981	2.125069	-0.642231
H	-4.273888	-0.230639	-0.148772
H	-2.908179	2.094071	0.198842
O	0.052631	-1.394614	-0.097761
O	-0.017692	1.529332	0.182770
N	-2.330691	-1.113372	-0.187531
H	-2.572723	-2.084076	-0.312216

N	2.406206	-1.124376	0.150558
H	2.832508	-1.786663	0.785407

---

3 (X = NH X' = O X'' = S)

S0

E = -891.265584258 A.U.

G = -891.169581 A.U.

C	3.323287	-0.342769	0.141834
C	1.267967	0.523520	-0.018597
C	1.152884	-0.844769	-0.069979
C	2.452513	-1.401307	0.033645
C	-1.267968	0.523520	-0.018613
C	-1.152883	-0.844770	-0.069978
C	-3.323288	-0.342769	0.141822
C	-2.452511	-1.401308	0.033667
H	4.395340	-0.320796	0.240121
H	2.700732	-2.450175	0.029232
H	-4.395343	-0.320796	0.240078
H	-2.700730	-2.450176	0.029274
O	0.000000	-1.580303	-0.191601
S	-0.000000	1.753001	-0.124326
N	-2.598506	0.816472	0.112861
H	-2.985972	1.744827	0.180243
N	2.598506	0.816472	0.112858
H	2.985974	1.744827	0.180229

T1

E = -891.170833590 A.U.

G = -891.078517 A.U.

C	3.280531	-0.654510	0.073690
C	1.371290	0.545049	-0.042575
C	1.058647	-0.848562	0.025638
C	2.264100	-1.577903	0.095697
C	-1.452571	0.564213	0.423714
C	-1.296920	-0.758187	0.041139
C	-3.467312	-0.313613	-0.113752
C	-2.558485	-1.331059	-0.270230
H	4.348073	-0.795833	0.117172
H	2.354827	-2.649642	0.152737
H	-4.539486	-0.309025	-0.227569
H	-2.761167	-2.347103	-0.569114
O	-0.124671	-1.456936	-0.028405
S	0.414580	1.934903	-0.184696
N	-2.785289	0.817161	0.276521
H	-3.224898	1.697158	0.495806
N	2.745608	0.595802	-0.013158
H	3.268834	1.458180	-0.030120

---

3 (X = NH X' = S X'' = NH)

S0

E = -871.415198677 A.U.

G = -871.305595 A.U.

C	3.314251	-0.349730	-0.326161
C	1.165965	-0.746049	0.147026
C	1.318972	0.616070	0.047319
C	2.686896	0.870731	-0.259597

C	-1.165960	-0.746054	0.146999
C	-1.318970	0.616065	0.047286
C	-3.314281	-0.349729	-0.326031
C	-2.686895	0.870723	-0.259629
H	4.342914	-0.602948	-0.520069
H	3.147664	1.834583	-0.406468
H	-4.342962	-0.602947	-0.519840
H	-3.147666	1.834574	-0.406497
S	-0.000003	1.783674	0.259478
N	0.000003	-1.511264	0.376114
H	-0.000007	-1.932948	1.302676
N	-2.378587	-1.324514	-0.093361
H	-2.542117	-2.319639	-0.115742
N	2.378612	-1.324507	-0.093241
H	2.542154	-2.319631	-0.115558

T1
E = -871.307430826 A.U.
G = -871.202602 A.U.

C	3.337545	-0.383056	-0.157558
C	1.135136	-0.762141	-0.050304
C	1.330721	0.610310	0.040970
C	2.737129	0.840437	-0.019118
C	-1.198278	-0.749108	0.501204
C	-1.303448	0.670213	0.051272
C	-3.301308	-0.303366	-0.297555
C	-2.600473	0.881357	-0.412621
H	4.376393	-0.648158	-0.258404
H	3.235500	1.795691	0.015720
H	-4.316561	-0.517186	-0.594680
H	-2.996975	1.804274	-0.810198
S	0.025564	1.771395	0.184897
N	-0.057052	-1.424137	0.032920
H	-0.087939	-2.431817	-0.052917
N	-2.462221	-1.347158	0.165026
H	-2.870008	-1.946992	0.872821
N	2.362006	-1.354435	-0.172427
H	2.529297	-2.345902	-0.247066

---

3 (X = NH X' = S X'' = O)
S0
E = -891.271466991 A.U.
G = -891.175401 A.U.

C	3.324590	-0.384583	0.000031
C	1.132192	-0.737665	-0.000016
C	1.305385	0.620933	-0.000002
C	2.715708	0.844299	0.000017
C	-1.132193	-0.737665	-0.000014
C	-1.305385	0.620933	-0.000002
C	-3.324590	-0.384582	0.000023
C	-2.715707	0.844299	0.000031
H	4.365890	-0.657409	0.000048
H	3.211172	1.801912	0.000031
H	-4.365890	-0.657409	0.000032
H	-3.211172	1.801912	0.000049
S	0.000000	1.823486	-0.000018
O	-0.000000	-1.501789	-0.000038

N	-2.343611	-1.348004	-0.000002
H	-2.483882	-2.347119	0.000011
N	2.343611	-1.348004	0.000006
H	2.483882	-2.347118	-0.000018

T1
E = -891.161551355 A.U.
G = -891.069275 A.U.

C	-3.268450	-0.466276	0.183553
C	-1.063045	-0.741971	0.064167
C	-1.304841	0.609081	-0.047157
C	-2.721199	0.780230	0.023801
C	1.144549	-0.694773	-0.599168
C	1.295354	0.713032	-0.095625
C	3.188933	-0.395509	0.381372
C	2.533644	0.809817	0.543364
H	-4.293967	-0.774317	0.295570
H	-3.259357	1.713231	-0.020020
H	4.138057	-0.716108	0.780086
H	2.925245	1.668057	1.067586
S	-0.030843	1.816693	-0.200889
O	0.097429	-1.428808	-0.004293
N	2.421533	-1.259658	-0.420785
H	2.525311	-2.261858	-0.361437
N	-2.250111	-1.391224	0.202878
H	-2.350874	-2.391239	0.286283

3 (X = NH X' = S X'' = S)
S0
E = -1214.25277071 A.U.
G = -1214.158989 A.U.

C	-3.234000	-0.032419	0.801528
C	-1.308337	-0.639851	-0.146271
C	-1.335637	0.735958	-0.121987
C	-2.559208	1.124986	0.490336
C	1.308328	-0.639851	-0.146249
C	1.335630	0.735958	-0.121967
C	3.234007	-0.032419	0.801515
C	2.559212	1.124986	0.490332
H	-4.197377	-0.184782	1.259134
H	-2.897569	2.132113	0.673873
H	4.197384	-0.184784	1.259122
H	2.897576	2.132113	0.673863
S	0.000000	-1.659529	-0.749677
S	0.000000	1.743291	-0.706766
N	-2.458186	-1.097258	0.429778
H	-2.692551	-2.068663	0.568401
N	2.458190	-1.097259	0.429771
H	2.692538	-2.068662	0.568432

T1
E = -1214.14986277 A.U.
G = -1214.061402 A.U.

C	-3.471138	-0.241496	0.168723
C	-1.420317	0.673430	-0.078489
C	-1.285783	-0.735796	0.032660
C	-2.592452	-1.287215	0.188865

C	1.441610	0.617763	0.520470
C	1.462601	-0.704542	0.042956
C	3.564026	0.045704	-0.034198
C	2.806936	-1.064600	-0.286373
H	-4.544322	-0.226878	0.263949
H	-2.835962	-2.331245	0.302297
H	4.626021	0.206358	-0.129578
H	3.160386	-2.015092	-0.653985
S	-0.289148	1.932045	-0.312394
S	0.098490	-1.746576	-0.114877
N	-2.763889	0.920843	0.007475
H	-3.159340	1.849026	-0.014213
N	2.725813	1.045411	0.427635
H	3.037372	1.967066	0.694410

3 (X = O X' = NH X'' = NH)

S0

E = -568.273857745 A.U.

G = -568.171994 A.U.

C	0.078213	0.095261	3.223789
C	0.611490	-0.050465	1.124092
C	-0.744577	-0.044469	1.159502
C	-1.095210	0.059283	2.547489
N	1.465453	-0.031611	-0.000000
C	0.611490	-0.050465	-1.124092
C	-0.744577	-0.044469	-1.159502
N	-1.568580	-0.026824	0.000000
C	0.078213	0.095261	-3.223789
C	-1.095210	0.059283	-2.547489
H	0.329669	0.141762	4.268888
H	-2.085292	0.088512	2.972985
H	2.116042	-0.816298	-0.000000
H	-2.249729	-0.779637	0.000000
H	0.329669	0.141762	-4.268888
H	-2.085292	0.088512	-2.972985
O	1.135489	0.051820	-2.359968
O	1.135489	0.051820	2.359968

T1

E = -568.181132552 A.U.

G = -568.085611 A.U.

C	-3.218654	-0.108064	-0.090849
C	-1.106649	-0.615021	-0.087860
C	-1.171693	0.749718	0.046466
C	-2.568533	1.072924	0.038495
N	0.038916	-1.348546	-0.041592
C	1.153107	-0.656075	0.476931
C	1.171538	0.798161	0.156179
N	-0.014864	1.497927	0.175253
C	3.202150	-0.050162	-0.218549
C	2.463039	1.118134	-0.233510
H	-4.259062	-0.371196	-0.164204
H	-3.019768	2.048956	0.109776
H	0.001613	-2.359660	-0.027817
H	-0.024593	2.477976	-0.068829
H	4.207653	-0.273848	-0.528834
H	2.837144	2.090796	-0.514306

O	2.404489	-1.156050	0.120198
O	-2.336637	-1.158498	-0.153351

---

3 (X = O X' = NH X'' = O)
S0
E = -588.128711771 A.U.
G = -588.039352 A.U.

C	-0.130595	-0.044962	3.193040
C	-0.591380	0.038384	1.089842
C	0.760663	0.011452	1.148659
C	1.062562	-0.045491	2.551563
C	-0.591380	0.038384	-1.089842
C	0.760663	0.011452	-1.148659
N	1.602352	-0.031143	-0.000000
C	-0.130595	-0.044962	-3.193040
C	1.062562	-0.045491	-2.551563
H	-0.418339	-0.064705	4.229018
H	2.038279	-0.075044	3.008568
H	2.274058	0.730963	-0.000000
H	-0.418339	-0.064705	-4.229018
H	2.038279	-0.075044	-3.008568
O	-1.415838	0.043721	0.000000
O	-1.163669	-0.005989	-2.292757
O	-1.163669	-0.005989	2.292757

T1
E = -588.008121543 A.U.
G = -587.921925 A.U.

C	-0.099134	-0.218986	3.171604
C	-0.643034	0.273789	1.113908
C	0.782829	0.155538	1.163567
C	1.090000	-0.104515	2.526015
C	-0.643034	0.273789	-1.113908
C	0.782829	0.155538	-1.163567
N	1.485928	0.120547	-0.000000
C	-0.099134	-0.218986	-3.171604
C	1.090000	-0.104515	-2.526015
H	-0.346475	-0.388766	4.204932
H	2.065131	-0.205207	2.974747
H	2.491518	0.006246	-0.000000
H	-0.346475	-0.388766	-4.204932
H	2.065131	-0.205207	-2.974747
O	-1.364443	0.027356	0.000000
O	-1.186420	-0.071931	-2.319020
O	-1.186420	-0.071931	2.319020

---

3 (X = O X' = NH X'' = S)
S0
E = -911.108737755 A.U.
G = -911.024485 A.U.

C	0.231560	0.149076	3.299694
C	-0.490067	-0.013751	1.255018
C	0.864607	-0.075863	1.175144
C	1.336783	0.034205	2.525822
C	-0.490067	-0.013751	-1.255018
C	0.864607	-0.075863	-1.175144

N	1.615360	-0.254975	-0.000000
C	0.231560	0.149076	-3.299694
C	1.336783	0.034205	-2.525822
H	0.080089	0.257701	4.359620
H	2.362940	0.028346	2.856381
H	2.494060	0.247770	-0.000000
H	0.080089	0.257701	-4.359620
H	2.362940	0.028346	-2.856381
S	-1.730886	-0.126326	0.000000
O	-0.894253	0.116385	-2.538961
O	-0.894253	0.116385	2.538961

T1
E = -911.026485179 A.U.
G = -910.947190 A.U.

C	-3.532618	-0.258233	0.000255
C	-1.524430	0.648200	-0.000697
C	-1.367876	-0.718514	-0.000017
C	-2.681269	-1.306707	0.000552
C	1.420365	0.515674	0.000019
C	1.067606	-0.846796	-0.000024
N	-0.169306	-1.396002	0.000089
C	3.279516	-0.679737	-0.000169
C	2.292158	-1.595812	-0.000140
H	-4.607450	-0.186769	0.000465
H	-2.937618	-2.354108	0.001116
H	-0.213419	-2.407033	0.000234
H	4.353080	-0.766338	-0.000258
H	2.393676	-2.668797	-0.000201
S	0.560327	1.971355	0.000230
O	2.786904	0.584661	-0.000082
O	-2.848038	0.923455	-0.000459

---

3 (X = O X' = O X'' = NH)
S0
E = -588.128845373 A.U.
G = -588.039644 A.U.

C	3.194616	-0.025682	-0.098110
C	1.111549	-0.629963	0.037304
C	1.121647	0.725466	0.056260
C	2.488270	1.130036	-0.038021
C	-1.111549	-0.629962	0.037283
C	-1.121646	0.725467	0.056250
C	-3.194619	-0.025683	-0.098055
C	-2.488268	1.130036	-0.038062
H	4.246056	-0.244959	-0.157160
H	2.879304	2.133791	-0.049943
H	-4.246059	-0.244961	-0.157111
H	-2.879302	2.133792	-0.049963
O	-0.000001	1.523574	0.113252
N	0.000001	-1.501025	0.033207
H	-0.000008	-2.121812	0.842745
O	-2.363170	-1.108473	-0.066018
O	2.363171	-1.108473	-0.065997

T1
E = -588.027202201 A.U.

```
G = -587.944173 A.U.
C       -3.149837   -0.001061   -0.257667
C       -1.144484   -0.668805    0.510716
C       -1.123024    0.792158    0.169860
C       -2.370203    1.147199   -0.281688
C        1.080682   -0.625848   -0.091796
C        1.135221    0.728723    0.065628
C        3.183455   -0.082141   -0.107538
C        2.516285    1.086944    0.052148
H       -4.147077   -0.197075   -0.610854
H       -2.692524    2.120190   -0.617215
H        4.226431   -0.329120   -0.195532
H        2.944910    2.071283    0.139534
O        0.027531    1.516333    0.181127
N       -0.063643   -1.370520   -0.038921
H       -0.041025   -2.381558   -0.078289
O        2.315805   -1.143685   -0.186499
O       -2.420059   -1.116783    0.164976
```

```
3 (X = O X' = O X'' = O)
S0
E = -607.981146980 A.U.
G = -607.904668 A.U.
C        3.169703   -0.079149   -0.000052
C        1.079312   -0.608880    0.000036
C        1.113143    0.742880   -0.000047
C        2.497535    1.098269    0.000090
C       -1.079317   -0.608881    0.000012
C       -1.113133    0.742878   -0.000049
C       -3.169706   -0.079144   -0.000099
C       -2.497531    1.098270    0.000162
H        4.213809   -0.335788   -0.000016
H        2.920989    2.088778    0.000065
H       -4.213814   -0.335775   -0.000179
H       -2.920979    2.088782    0.000242
O        0.000002   -1.448321    0.000003
O        0.000002    1.557837   -0.000123
O       -2.299796   -1.138723    0.000019
O        2.299787   -1.138725    0.000047

T1
E = -607.880823997 A.U.
G = -607.809394 A.U.
C       -3.092413   -0.059260    0.283890
C       -1.097963   -0.645052   -0.535956
C       -1.112718    0.820013   -0.178337
C       -2.347619    1.109499    0.345710
C        1.028868   -0.605000    0.102693
C        1.120558    0.734295   -0.078582
C        3.138621   -0.140370    0.116155
C        2.514016    1.048901   -0.065941
H       -4.066606   -0.313816    0.662312
H       -2.687137    2.049344    0.750823
H        4.172269   -0.421122    0.211928
H        2.975032    2.016765   -0.169436
O       -0.076687   -1.375962    0.074436
O        0.027032    1.551196   -0.205691
```

O	2.235750	-1.167722	0.210808
O	-2.348801	-1.121178	-0.253730

---

3 (X = O X' = O X'' = S)
S0
E = -930.962801897 A.U.
G = -930.890933 A.U.

C	3.275742	0.275927	0.000697
C	1.240380	-0.498637	-0.000155
C	1.139553	0.854209	-0.000379
C	2.473298	1.366485	0.000574
C	-1.240377	-0.498631	-0.000294
C	-1.139560	0.854213	-0.000498
C	-3.275743	0.275916	0.000572
C	-2.473308	1.366481	0.000332
H	4.343605	0.144443	0.001088
H	2.771011	2.401853	0.000790
H	-4.343607	0.144434	0.000968
H	-2.771023	2.401849	0.000531
O	-0.000000	1.611991	-0.001191
S	0.000008	-1.755116	-0.000484
O	-2.537571	-0.867655	0.000351
O	2.537570	-0.867649	0.000749

T1
E = -930.861351376 A.U.
G = -930.794226 A.U.

C	-3.271998	0.185986	0.046284
C	-1.216077	-0.521541	-0.120698
C	-1.162600	0.833430	0.026317
C	-2.507519	1.300344	0.134041
C	1.239918	-0.483152	0.575408
C	1.124857	0.922019	0.097597
C	3.219682	0.263758	-0.168512
C	2.360901	1.318011	-0.381477
H	-4.335970	0.024535	0.062910
H	-2.839454	2.318111	0.253684
H	4.250788	0.098805	-0.427549
H	2.613184	2.261800	-0.837758
O	-0.041847	1.599616	0.043146
S	0.089589	-1.658325	-0.218283
O	2.568440	-0.805322	0.453174
O	-2.507214	-0.929693	-0.097885

---

3 (X = O X' = S X'' = NH)
S0
E = -911.118278055 A.U.
G = -911.033830 A.U.

C	3.291358	-0.389950	-0.123255
C	1.151382	-0.738390	0.055187
C	1.305951	0.608606	0.022946
C	2.719485	0.835333	-0.102682
C	-1.151381	-0.738390	0.055189
C	-1.305952	0.608607	0.022947
C	-3.291358	-0.389951	-0.123256
C	-2.719486	0.835332	-0.102683

H	4.309241	-0.733079	-0.182874
H	3.225792	1.785153	-0.158348
H	-4.309242	-0.733079	-0.182868
H	-3.225793	1.785152	-0.158356
S	0.000000	1.805357	0.103415
N	0.000000	-1.532299	0.088950
H	0.000001	-2.218491	0.840763
O	-2.337958	-1.364528	-0.041373
O	2.337957	-1.364528	-0.041374

T1
E = -911.020352537 A.U.
G = -910.940419 A.U.

C	3.261329	-0.448256	0.167080
C	1.107531	-0.737427	0.053750
C	1.312820	0.616575	-0.019518
C	2.737555	0.791604	0.045971
C	-1.181437	-0.735993	-0.547987
C	-1.301430	0.686917	-0.105301
C	-3.219584	-0.374615	0.315616
C	-2.590959	0.839507	0.408215
H	4.265129	-0.820188	0.273522
H	3.283005	1.720684	0.023394
H	-4.195258	-0.703283	0.631362
H	-3.027095	1.737313	0.818204
S	0.025677	1.788928	-0.169863
N	-0.061313	-1.418901	-0.053174
H	-0.047059	-2.431822	-0.048939
O	-2.400106	-1.351716	-0.230151
O	2.273194	-1.401176	0.165841

---

3 (X = O X' = S X'' = O)
S0
E = -930.972672671 A.U.
G = -930.900665 A.U.

C	-3.255970	-0.440390	0.000156
C	-1.115722	-0.723037	-0.000116
C	-1.291206	0.617596	-0.000013
C	-2.718047	0.800237	0.000255
C	1.115723	-0.723036	-0.000112
C	1.291207	0.617599	0.000020
C	3.255969	-0.440391	0.000128
C	2.718049	0.800240	0.000244
H	-4.264134	-0.814729	0.000198
H	-3.253110	1.735661	0.000437
H	4.264133	-0.814728	0.000207
H	3.253113	1.735663	0.000440
S	-0.000002	1.832752	-0.000107
O	0.000002	-1.496995	-0.000283
O	2.269471	-1.390178	-0.000065
O	-2.269471	-1.390177	-0.000019

T1
E = -930.873019900 A.U.
G = -930.805133 A.U.

C	3.215413	-0.495049	0.157072
C	1.065224	-0.724463	0.064199

C	1.293032	0.612035	-0.037537
C	2.723539	0.756105	0.019935
C	-1.128596	-0.717203	-0.550639
C	-1.290054	0.700705	-0.087112
C	-3.169374	-0.432227	0.299196
C	-2.573581	0.794219	0.447423
H	4.209804	-0.892086	0.259201
H	3.290176	1.671467	-0.023137
H	-4.124441	-0.814765	0.615982
H	-3.030287	1.657049	0.906123
S	0.022409	1.815481	-0.160125
O	-0.068373	-1.431956	0.003859
O	-2.324650	-1.353752	-0.316913
O	2.203346	-1.418553	0.179131

---

3 (X = O X' = S X'' = S)

S0

E = -1253.94777444 A.U.

G = -1253.879473 A.U.

C	-3.233376	-0.086685	0.686848
C	-1.291208	-0.620350	-0.129074
C	-1.325077	0.736199	-0.107299
C	-2.605065	1.087076	0.439728
C	1.291190	-0.620339	-0.129075
C	1.325092	0.736210	-0.107306
C	3.233374	-0.086704	0.686845
C	2.605082	1.087068	0.439720
H	-4.204923	-0.337633	1.076504
H	-2.990967	2.078166	0.613406
H	4.204930	-0.337647	1.076485
H	2.991002	2.078152	0.613398
S	0.000001	-1.707112	-0.630146
S	-0.000000	1.792730	-0.609576
O	2.434808	-1.140361	0.360849
O	-2.434825	-1.140361	0.360832

T1

E = -1253.84789852 A.U.

G = -1253.784488 A.U.

C	-3.340062	-0.093129	-0.168139
C	-1.283988	-0.581779	0.566860
C	-1.314003	0.805615	0.045906
C	-2.615900	1.032817	-0.428036
C	1.263773	-0.644549	-0.110837
C	1.346070	0.715177	0.015032
C	3.389106	-0.165919	0.011255
C	2.748336	1.019472	0.104253
H	-4.359075	-0.368298	-0.380664
H	-2.990918	1.926346	-0.901986
H	4.429463	-0.442194	0.010407
H	3.196934	1.993643	0.210131
S	-0.091316	-1.718179	-0.194683
S	0.037427	1.867453	0.066428
O	2.503739	-1.191636	-0.111414
O	-2.575509	-1.061378	0.473467

---

3 (X = S X' = NH X'' = NH)
S0
E = -1214.23213658 A.U.
G = -1214.139729 A.U.

C	0.439449	-0.087305	3.451166
C	-0.471278	0.073714	1.161107
C	0.892015	0.048922	1.181439
C	1.416465	-0.045914	2.508327
N	-1.288545	0.107380	-0.000000
C	-0.471278	0.073714	-1.161107
C	0.892015	0.048922	-1.181439
N	1.675567	0.025919	0.000000
C	0.439449	-0.087305	-3.451166
C	1.416465	-0.045914	-2.508327
H	0.543323	-0.132352	4.523568
H	2.474686	-0.070384	2.731239
H	-1.907146	0.914779	-0.000000
H	2.424655	0.709557	0.000000
H	0.543323	-0.132352	-4.523568
H	2.474686	-0.070384	-2.731239
S	-1.143203	-0.063280	-2.751507
S	-1.143203	-0.063280	2.751507

T1
E = -1214.15166067 A.U.
G = -1214.064015 A.U.

C	-3.426745	0.470715	0.264085
C	-1.170086	-0.493001	-0.396405
C	-1.188337	0.951337	-0.110453
C	-2.440252	1.445803	0.215750
N	-0.022670	-1.176470	0.011754
C	1.152671	-0.483638	0.053138
C	1.190945	0.896422	-0.082696
N	0.016176	1.615263	-0.154243
C	3.451239	0.421818	0.025967
C	2.525095	1.405771	-0.088107
H	-4.451076	0.579477	0.581372
H	-2.634349	2.489396	0.428406
H	-0.022444	-2.186099	0.060464
H	0.040855	2.615885	-0.014882
H	4.524628	0.516800	0.063635
H	2.767566	2.456663	-0.167698
S	2.747589	-1.167199	0.136143
S	-2.794270	-1.159991	-0.088740

---

3 (X = S X' = NH X'' = O)
S0
E = -1234.08545573 A.U.
G = -1234.005832 A.U.

C	0.389576	0.039528	3.426117
C	-0.442311	-0.018416	1.125623
C	0.918011	-0.029098	1.168290
C	1.396676	0.002523	2.514665
C	-0.442311	-0.018416	-1.125623
C	0.918011	-0.029098	-1.168290
N	1.721277	-0.111568	0.000000
C	0.389576	0.039528	-3.426117

C	1.396676	0.002523	-2.514665
H	0.459254	0.070292	4.501549
H	2.446668	-0.001863	2.773720
H	2.470116	0.573706	0.000000
H	0.459254	0.070292	-4.501549
H	2.446668	-0.001863	-2.773720
O	-1.233468	-0.058404	-0.000000
S	-1.175206	0.018850	2.684893
S	-1.175206	0.018850	-2.684893

T1
E = -1233.99868791 A.U.
G = -1233.923042 A.U.

C	3.382372	0.412438	0.317823
C	1.119532	-0.470546	-0.418521
C	1.177069	0.978217	-0.104103
C	2.434799	1.416698	0.288927
C	-1.108115	-0.448272	0.087486
C	-1.176692	0.912579	-0.097743
N	-0.012908	1.653387	-0.177079
C	-3.418188	0.363988	-0.004247
C	-2.528309	1.378604	-0.139917
H	4.392522	0.467796	0.690756
H	2.653289	2.440789	0.562784
H	-0.044293	2.652667	-0.025747
H	-4.494678	0.424497	0.016793
H	-2.805194	2.416895	-0.258457
O	0.031763	-1.171622	0.090300
S	2.719434	-1.174692	-0.171559
S	-2.666946	-1.191909	0.168609

---

3 (X = S X' = NH X'' = S)
S0
E = -1557.07640140 A.U.
G = -1556.999921 A.U.

C	0.724438	-0.465333	3.370586
C	-0.436485	0.182676	1.295769
C	0.921363	0.295126	1.192196
C	1.592683	-0.083396	2.395888
C	-0.436485	0.182676	-1.295769
C	0.921363	0.295126	-1.192196
N	1.550305	0.696693	0.000000
C	0.724438	-0.465333	-3.370586
C	1.592683	-0.083396	-2.395888
H	0.950568	-0.787195	4.375225
H	2.667525	-0.064366	2.517101
H	2.552838	0.566923	0.000000
H	0.950568	-0.787195	-4.375225
H	2.667525	-0.064366	-2.517101
S	-1.555003	0.636893	-0.000000
S	-0.918284	-0.408744	-2.847076
S	-0.918284	-0.408744	2.847076

T1
E = -1556.98890463 A.U.
G = -1556.916912 A.U.

C	3.480372	0.679971	0.260333

C	1.279011	-0.426922	-0.389183
C	1.201549	1.001197	-0.027765
C	2.427639	1.561251	0.335773
C	-1.300292	-0.430052	0.103407
C	-1.210251	0.943161	-0.053184
N	-0.019337	1.624933	-0.045545
C	-3.505955	0.661416	-0.139901
C	-2.496454	1.562133	-0.189023
H	4.507221	0.839253	0.547384
H	2.540612	2.591455	0.649463
H	-0.050281	2.629813	0.067601
H	-4.565818	0.849335	-0.213683
H	-2.637917	2.626825	-0.316209
S	0.047544	-1.499433	0.295007
S	-2.952158	-0.967640	0.054399
S	2.972602	-0.921935	-0.338061

---

3 (X = S X' = O X'' = NH)

S0

E = -1234.09056885 A.U.

G = -1234.010437 A.U.

C	3.401198	0.488945	-0.146200
C	1.149387	-0.498101	0.104118
C	1.143688	0.863913	0.091643
C	2.436373	1.441610	-0.053074
C	-1.149388	-0.498104	0.104119
C	-1.143688	0.863910	0.091641
C	-3.401207	0.488943	-0.146121
C	-2.436375	1.441607	-0.053064
H	4.467236	0.623404	-0.235902
H	2.616953	2.506931	-0.071320
H	-4.467251	0.623400	-0.235756
H	-2.616958	2.506929	-0.071285
O	-0.000002	1.628283	0.177462
N	0.000001	-1.326600	0.176954
H	0.000004	-1.902172	1.015538
S	-2.752565	-1.114216	-0.094342
S	2.752571	-1.114215	-0.094285

T1

E = -1233.99853487 A.U.

G = -1233.923335 A.U.

C	-3.411270	0.437701	0.020465
C	-1.126141	-0.497177	0.064333
C	-1.158403	0.867689	-0.119523
C	-2.473111	1.406141	-0.138514
C	1.161066	-0.509398	-0.449301
C	1.137566	0.936140	-0.125714
C	3.345957	0.505962	0.373192
C	2.328856	1.457936	0.308227
H	-4.482892	0.546236	0.067228
H	-2.688066	2.458580	-0.254779
H	4.329846	0.627045	0.797533
H	2.458973	2.492494	0.595225
O	-0.030805	1.628244	-0.209660
N	0.048645	-1.201317	0.014553
H	0.059394	-2.207999	0.111941

S	2.813933	-1.109068	-0.151275
S	-2.726336	-1.151123	0.192480

---

3 (X = S X' = O X'' = O)
S0
E = -1253.94124678 A.U.
G = -1253.874405 A.U.

C	-3.392018	0.437914	0.000582
C	-1.116054	-0.466815	-0.000160
C	-1.133793	0.892776	-0.000092
C	-2.454711	1.422388	0.000323
C	1.116054	-0.466815	-0.000384
C	1.133792	0.892776	-0.000307
C	3.392017	0.437910	0.000398
C	2.454713	1.422387	0.000074
H	-4.465462	0.536658	0.000934
H	-2.671969	2.480897	0.000404
H	4.465461	0.536655	0.000781
H	2.671969	2.480897	0.000217
O	-0.000002	-1.270352	-0.000639
O	0.000001	1.677917	-0.000434
S	2.696106	-1.147836	-0.000199
S	-2.696105	-1.147837	0.000427

T1
E = -1253.82824386 A.U.
G = -1253.760781 A.U.

C	-3.366424	0.434705	-0.162946
C	-1.097695	-0.474238	0.276570
C	-1.129695	0.920497	-0.024361
C	-2.427280	1.422526	-0.221740
C	1.097668	-0.474214	-0.276381
C	1.129716	0.920447	0.024513
C	3.366480	0.434648	0.162695
C	2.427371	1.422466	0.221665
H	-4.433471	0.531789	-0.286037
H	-2.649729	2.464160	-0.404079
H	4.433568	0.531726	0.285443
H	2.649880	2.464107	0.403887
O	-0.000042	-1.211380	-0.000039
O	0.000022	1.654278	0.000122
S	2.701862	-1.161757	-0.166542
S	-2.701921	-1.161741	0.166544

---

3 (X = S X' = O X'' = S)
S0
E = -1576.93049096 A.U.
G = -1576.867123 A.U.

C	3.370969	0.770311	-0.374058
C	1.286974	-0.446304	0.149073
C	1.159598	0.906656	0.247340
C	2.355118	1.616293	-0.058196
C	-1.286977	-0.446305	0.149072
C	-1.159595	0.906656	0.247338
C	-3.370966	0.770314	-0.374069
C	-2.355114	1.616294	-0.058201

H	4.388737	1.014576	-0.634655
H	2.431925	2.693812	-0.030685
H	-4.388730	1.014581	-0.634679
H	-2.431919	2.693813	-0.030702
O	0.000000	1.563970	0.585017
S	-2.883520	-0.886081	-0.340316
S	2.883516	-0.886083	-0.340326
S	-0.000000	-1.608588	0.498191

T1
E = -1576.83390502 A.U.
G = -1576.774690 A.U.

C	3.468976	0.664675	-0.147503
C	1.270291	-0.432911	0.121890
C	1.184364	0.922119	-0.080579
C	2.450681	1.556439	-0.235589
C	-1.263683	-0.430613	-0.435347
C	-1.144604	0.993395	-0.034811
C	-3.397193	0.704892	0.354563
C	-2.311163	1.563926	0.424668
H	4.528689	0.855705	-0.212293
H	2.566586	2.618930	-0.393786
H	-4.391039	0.862345	0.742213
H	-2.368486	2.575184	0.803958
O	0.039004	1.650239	-0.096409
S	-2.978183	-0.862331	-0.382283
S	2.923714	-0.955234	0.108331
S	-0.082643	-1.517786	0.275667

---

3 (X = S X' = S X'' = NH)
S0
E = -1557.07387786 A.U.
G = -1556.998238 A.U.

C	3.475558	0.103379	-0.425829
C	1.182258	-0.536076	0.250078
C	1.330341	0.807703	0.080176
C	2.652826	1.179238	-0.313551
C	-1.182266	-0.536067	0.250120
C	-1.330336	0.807714	0.080210
C	-3.475489	0.103386	-0.426077
C	-2.652791	1.179254	-0.313609
H	4.525307	0.089379	-0.673737
H	2.966501	2.199755	-0.484745
H	-4.525198	0.089387	-0.674158
H	-2.966436	2.199767	-0.484878
S	0.000008	1.958183	0.334812
N	-0.000004	-1.233999	0.558078
H	0.000012	-1.676521	1.471360
S	2.653655	-1.382700	-0.109667
S	-2.653710	-1.382668	-0.109488

T1
E = -1556.98429118 A.U.
G = -1556.912520 A.U.

C	-3.537581	0.092047	0.104873
C	-1.150400	-0.568205	0.032405
C	-1.337393	0.796940	-0.082250

C	-2.719587	1.161528	-0.045653
C	1.197466	-0.540129	-0.465144
C	1.313200	0.869943	-0.037447
C	3.456080	0.144612	0.470317
C	2.563671	1.196214	0.474254
H	-4.612632	0.077712	0.188221
H	-3.071368	2.181847	-0.112501
H	4.458171	0.132685	0.869788
H	2.819577	2.178483	0.850373
S	-0.027779	1.948089	-0.225504
N	0.046778	-1.213770	-0.062884
H	0.063731	-2.225129	-0.014293
S	-2.667459	-1.409019	0.194839
S	2.776635	-1.337002	-0.222431

3 (X = S X' = S X'' = O)

S0

E = -1576.92645724 A.U.

G = -1576.863138 A.U.

C	3.419736	0.036350	-0.458609
C	1.142785	-0.498760	0.270778
C	1.312212	0.834608	0.077461
C	2.641281	1.144317	-0.349688
C	-1.142776	-0.498776	0.270744
C	-1.312211	0.834594	0.077439
C	-3.419811	0.036348	-0.458325
C	-2.641301	1.144299	-0.349650
H	4.459319	-0.025689	-0.738793
H	2.988670	2.147716	-0.552176
H	-4.459459	-0.025684	-0.738267
H	-2.988716	2.147702	-0.552079
S	-0.000015	2.005311	0.332926
O	0.000007	-1.146252	0.658988
S	-2.561352	-1.417421	-0.078174
S	2.561406	-1.417385	-0.077970

T1

E = -1576.83040489 A.U.

G = -1576.771144 A.U.

C	3.498879	0.034648	0.073056
C	1.110522	-0.538993	0.059302
C	1.319982	0.804180	-0.094099
C	2.715286	1.128975	-0.087824
C	-1.143584	-0.517823	-0.465104
C	-1.298671	0.891866	-0.014656
C	-3.410015	0.081617	0.477800
C	-2.550342	1.159463	0.524531
H	4.574552	-0.010447	0.135749
H	3.096863	2.135734	-0.184303
H	-4.397817	0.016714	0.907360
H	-2.832193	2.113915	0.949375
S	0.024352	1.982453	-0.218920
O	-0.056673	-1.216556	0.024307
S	-2.700729	-1.349868	-0.294700
S	2.586354	-1.431777	0.211079

3 (X = S X' = S X'' = S)
S0
E = -528.568190331 A.U.
G = -528.442011 A.U.

C	-0.020805	-0.110110	3.282945
N	-1.089529	-0.061530	2.422988
C	-0.630439	0.049083	1.141342
C	0.743594	0.045203	1.170255
C	1.133461	-0.062782	2.536575
N	-1.477514	0.070711	-0.000000
C	-0.630439	0.049083	-1.141342
C	0.743594	0.045203	-1.170255
N	1.559610	0.046623	0.000000
N	-1.089529	-0.061530	-2.422988
C	-0.020805	-0.110110	-3.282945
C	1.133461	-0.062782	-2.536575
H	-0.170490	-0.170643	4.347668
H	-2.060999	-0.111004	2.687075
H	2.141953	-0.092476	2.918665
H	-2.061720	0.905502	-0.000000
H	2.209791	0.826101	0.000000
H	-2.060999	-0.111004	-2.687075
H	-0.170490	-0.170643	-4.347668
H	2.141953	-0.092476	-2.918665

T1
E = -528.467454605 A.U.
G = -528.347122 A.U.

C	-3.285883	-0.040066	-0.075276
N	-2.419059	-1.108334	-0.146553
C	-1.135638	-0.641853	-0.074720
C	-1.185772	0.739334	0.065763
C	-2.559094	1.117165	0.061842
N	0.032202	-1.354446	-0.063750
C	1.170588	-0.671204	0.422701
C	1.177160	0.781069	0.116688
N	-0.012277	1.474720	0.167869
N	2.469981	-1.151066	0.048987
C	3.275339	0.019666	-0.186776
C	2.465677	1.156863	-0.219041
H	-4.349805	-0.194048	-0.136129
H	-2.688237	-2.075412	-0.233574
H	-2.957701	2.115811	0.139745
H	0.028912	-2.363909	-0.111330
H	-0.025417	2.454044	-0.077851
H	2.876611	-1.792525	0.719915
H	4.291134	-0.089044	-0.535339
H	2.794313	2.153124	-0.478382

---

4 (X = NH X' = NH X'' = NH)
S0
E = -528.569100460 A.U.
G = -528.442130 A.U.

C	1.482801	2.929399	-0.115648
N	2.071384	1.689735	-0.052803
C	1.104012	0.731862	0.057268
C	-0.119011	1.359794	0.041221

C	0.119011	2.758312	-0.076749
N	1.368436	-0.658044	0.064484
C	0.119011	-1.359794	0.041221
C	-1.104012	-0.731862	0.057268
N	-1.368436	0.658044	0.064484
C	-0.119011	-2.758312	-0.076749
C	-1.482801	-2.929399	-0.115648
N	-2.071384	-1.689735	-0.052803
H	2.080190	3.823208	-0.177061
H	3.060977	1.504895	-0.102004
H	-0.624358	3.539206	-0.114283
H	1.921656	-0.924884	0.875034
H	-1.921656	0.924884	0.875034
H	0.624358	-3.539206	-0.114283
H	-2.080190	-3.823208	-0.177061
H	-3.060977	-1.504895	-0.102004

T1
E = -528.467414343 A.U.
G = -528.346841 A.U.

C	3.286115	-0.063446	-0.067458
N	2.479583	1.046363	0.071555
C	1.174008	0.647410	0.055015
C	1.142264	-0.733382	-0.085101
C	2.494979	-1.181147	-0.156097
N	0.050796	1.437906	0.158275
C	-1.159396	0.769510	0.116863
C	-1.180810	-0.683826	0.419785
N	-0.074144	-1.390518	-0.089681
C	-2.435486	1.179122	-0.217641
C	-3.272504	0.061362	-0.180850
N	-2.501899	-1.126985	0.059470
H	4.357578	0.039516	-0.091760
H	2.803496	1.996781	0.154066
H	2.835819	-2.198480	-0.261678
H	0.091230	2.405573	-0.131341
H	-0.101653	-2.399480	-0.129138
H	-2.740065	2.182932	-0.477077
H	-4.298544	-0.017815	-0.506215
H	-2.923237	-1.750003	0.738710

---

4 (X = NH X' = NH X'' = O)
S0
E = -548.427343140 A.U.
G = -548.313659 A.U.

C	3.256212	-0.024045	-0.080731
C	1.120889	-0.624164	0.052103
C	1.142377	0.747102	0.016126
C	2.511216	1.130720	-0.071996
C	-1.116394	-0.709856	0.046337
C	-1.144934	0.663250	0.029734
N	-0.044393	1.553504	0.024095
C	-2.445294	-1.193965	-0.028304
C	-3.253034	-0.080989	-0.081069
H	4.320752	-0.177541	-0.123429
H	2.895017	2.137801	-0.112848
H	-0.061502	2.157525	0.842941

H	-2.762510	-2.223971	-0.035834
H	-4.324973	0.005834	-0.128308
O	0.047927	-1.466380	0.090039
N	-2.455234	1.035842	-0.057109
H	-2.778900	1.989614	-0.099125
N	2.392978	-1.094625	-0.014159
H	2.649026	-2.069589	-0.026708

T1
E = -548.318346019 A.U.
G = -548.210049 A.U.

C	-3.249691	0.027069	-0.084811
C	-1.135266	-0.623223	0.076179
C	-1.120219	0.745940	-0.090921
C	-2.482646	1.160863	-0.187649
C	1.112293	-0.756390	0.119814
C	1.172369	0.696066	0.449461
N	0.092771	1.416396	-0.075394
C	2.347136	-1.205688	-0.262619
C	3.221463	-0.110429	-0.219652
H	-4.318380	-0.099180	-0.112070
H	-2.844729	2.168154	-0.314258
H	0.132278	2.422546	-0.157607
H	2.605304	-2.210022	-0.562717
H	4.244477	-0.060460	-0.558826
O	-0.067923	-1.448652	0.163378
N	2.509071	1.088710	0.089978
H	2.971135	1.680650	0.770159
N	-2.420476	-1.061410	0.080370
H	-2.708899	-2.023605	0.164810

---

4 (X = NH X' = O X'' = O)
S0
E = -568.282950119 A.U.
G = -568.181415 A.U.

C	-1.489938	2.866757	0.000346
C	-1.088822	0.679463	-0.000097
C	0.122703	1.319659	-0.000051
C	-0.122703	2.715331	0.000123
C	-0.122703	-1.319659	-0.000051
C	1.088822	-0.679463	-0.000097
C	0.122703	-2.715331	0.000123
C	1.489938	-2.866757	0.000346
H	-2.097685	3.754831	0.000518
H	0.613465	3.502323	0.000156
H	-0.613465	-3.502323	0.000156
H	2.097685	-3.754831	0.000518
O	-1.348576	-0.660827	-0.000207
O	1.348576	0.660827	-0.000207
N	2.068534	-1.618460	-0.000160
H	3.057252	-1.419481	0.000172
N	-2.068534	1.618460	-0.000160
H	-3.057252	1.419481	0.000172

T1
E = -568.172371628 A.U.
G = -568.076168 A.U.

C	-3.184136	0.057656	-0.222069
C	-1.125674	-0.684590	0.452870
C	-1.101464	0.779454	0.115725
C	-2.342227	1.179305	-0.289757
C	1.075074	-0.724868	-0.101302
C	1.119201	0.634323	0.077296
C	2.414916	-1.185812	-0.198490
C	3.214409	-0.073724	-0.085585
H	-4.195771	-0.038251	-0.583506
H	-2.630863	2.162555	-0.627974
H	2.742460	-2.203302	-0.333566
H	4.285923	0.023725	-0.111609
O	-0.100927	-1.435580	-0.105145
O	0.064301	1.483403	0.179132
N	2.415193	1.035189	0.087357
H	2.730732	1.988002	0.182255
N	-2.440500	-1.101123	0.141879
H	-2.882919	-1.744244	0.785721

4 (X = NH X' = S X'' = NH)
S0
E = -871.411681216 A.U.
G = -871.302643 A.U.

C	3.346917	-0.284196	-0.253127
C	1.188473	-0.759335	0.115333
C	1.305862	0.608255	0.032881
C	2.677015	0.913091	-0.207174
C	-1.169724	-0.850025	0.100334
C	-1.297223	0.519119	0.046933
C	-2.465823	-1.403718	-0.095117
C	-3.335120	-0.350800	-0.249832
H	4.391508	-0.502103	-0.397961
H	3.110613	1.893858	-0.320966
H	-2.715924	-2.452765	-0.115651
H	-4.399698	-0.331039	-0.411850
S	-0.040270	1.751126	0.214970
N	0.047885	-1.568742	0.263604
H	0.062760	-2.101568	1.128561
N	-2.614724	0.811602	-0.175562
H	-2.995364	1.738890	-0.283427
N	2.432409	-1.291096	-0.070721
H	2.629166	-2.279980	-0.100861

T1
E = -871.305166643 A.U.
G = -871.200135 A.U.

C	3.308369	-0.254978	-0.299131
C	1.215315	-0.756294	0.501922
C	1.288306	0.664853	0.049883
C	2.574419	0.911280	-0.420197
C	-1.130594	-0.849731	-0.058538
C	-1.309119	0.524204	0.035423
C	-2.424842	-1.438734	-0.187382
C	-3.331661	-0.410679	-0.158449
H	4.330023	-0.439173	-0.594613
H	2.944970	1.842671	-0.822943
H	-2.646209	-2.490100	-0.278118

H	-4.406386	-0.423496	-0.229490
S	-0.063535	1.741783	0.192432
N	2.502160	-1.316285	0.170202
H	2.927854	-1.903672	0.877728
N	-2.655731	0.772720	-0.014168
H	-3.082180	1.685861	0.001301
N	0.103237	-1.458550	0.021036
H	0.159658	-2.465348	-0.053440

---

4 (X = NH X' = S X'' = O)

S0

E = -891.269470672 A.U.

G = -891.173463 A.U.

C	3.332919	-0.329501	0.094957
C	1.151050	-0.747085	-0.048268
C	1.291327	0.615996	-0.006872
C	2.690429	0.881782	0.085184
C	-1.132840	-0.836204	-0.046685
C	-1.281028	0.529353	-0.014300
C	-2.414881	-1.432428	0.022777
C	-3.316909	-0.397233	0.094567
H	4.379786	-0.573491	0.151226
H	3.156376	1.852754	0.135174
H	-2.633363	-2.487761	0.020062
H	-4.391614	-0.404599	0.159664
S	-0.041123	1.787655	-0.080125
O	0.051609	-1.540284	-0.128348
N	2.380548	-1.318801	0.015027
H	2.548964	-2.313455	0.012569
N	-2.622970	0.781536	0.073894
H	-3.038496	1.699122	0.119463

T1

E = -891.158758684 A.U.

G = -891.066210 A.U.

C	3.273984	-0.305400	-0.265150
C	1.160738	-0.750472	0.486106
C	1.276025	0.673812	0.024701
C	2.572493	0.878313	-0.428358
C	-1.093848	-0.835894	-0.069259
C	-1.287513	0.526808	0.043452
C	-2.364982	-1.455307	-0.190554
C	-3.293287	-0.444416	-0.143949
H	4.287604	-0.532086	-0.557377
H	2.974245	1.787078	-0.850716
H	-2.557608	-2.510911	-0.289676
H	-4.368141	-0.476391	-0.201979
S	-0.056976	1.765701	0.180474
O	0.104328	-1.485875	-0.030191
N	2.430576	-1.324241	0.221672
H	2.810587	-1.982523	0.888869
N	-2.637705	0.748877	0.004724
H	-3.081460	1.653508	0.038111

---

4 (X = NH X' = S X'' = S)

S0

E = -1214.25350765 A.U.
G = -1214.159169 A.U.

C	1.516490	2.862420	0.794754
C	1.194031	0.861520	-0.145067
C	-0.021052	1.508069	-0.118117
C	0.178665	2.778792	0.487975
C	0.021052	-1.508069	-0.118117
C	-1.194031	-0.861520	-0.145067
C	-0.178665	-2.778792	0.487975
C	-1.516490	-2.862420	0.794754
H	2.086654	3.656616	1.247244
H	-0.568858	3.534533	0.667845
H	0.568858	-3.534533	0.667845
H	-2.086654	-3.656616	1.247244
S	1.516490	-0.773861	-0.723288
S	-1.516490	0.773861	-0.723288
N	2.117721	1.688869	0.424841
H	3.090194	1.461150	0.566357
N	-2.117721	-1.688869	0.424841
H	-3.090194	-1.461150	0.566357

T1
E = -1214.14414899 A.U.
G = -1214.055744 A.U.

C	3.539536	-0.002238	-0.059544
C	1.435762	-0.598748	0.561203
C	1.431230	0.713215	0.050801
C	2.756443	1.088191	-0.325146
C	-1.421319	-0.754060	-0.090085
C	-1.286063	0.651150	0.037142
C	-2.825222	-1.019723	-0.022258
C	-3.471940	0.171335	0.148599
H	4.601691	-0.146276	-0.177516
H	3.084257	2.033693	-0.727779
H	-3.278284	-1.996208	-0.074548
H	-4.520431	0.394324	0.259579
S	-0.199698	-1.936831	-0.307195
S	0.044371	1.733871	-0.103863
N	2.731173	-1.000653	0.450461
H	3.065591	-1.910511	0.729061
N	-2.540778	1.175270	0.186501
H	-2.750925	2.155286	0.305122

---

4 (X = O X' = NH X'' = NH)
S0
E = -568.274933998 A.U.
G = -568.172402 A.U.

C	-3.226553	0.033670	-0.095774
C	-1.140810	0.633970	0.057838
C	-1.140810	-0.723390	0.036081
C	-2.514174	-1.118598	-0.075371
N	-0.048506	1.515713	0.030953
C	1.140810	0.723390	0.036081
C	1.140810	-0.633970	0.057838
N	0.048506	-1.515713	0.030953
C	2.514174	1.118598	-0.075371
C	3.226553	-0.033670	-0.095774

H	-4.279030	0.253030	-0.136363
H	-2.907774	-2.121334	-0.115955
H	-0.072639	2.182472	0.798730
H	0.072639	-2.182472	0.798730
H	2.907774	2.121334	-0.115955
H	4.279030	-0.253030	-0.136363
O	2.394536	-1.116388	-0.037466
O	-2.394536	1.116388	-0.037466

T1
E = -568.181190094 A.U.
G = -568.085538 A.U.

C	3.203731	-0.006655	-0.209154
C	1.164636	-0.674430	0.467318
C	1.151983	0.781474	0.152904
C	2.432868	1.141738	-0.229591
N	0.088591	-1.399044	-0.073843
C	-1.121176	-0.731501	-0.086978
C	-1.153911	0.634290	0.042751
N	-0.060153	1.442699	0.160389
C	-2.494310	-1.142685	-0.154100
C	-3.224823	-0.005326	-0.076417
H	4.221386	-0.195643	-0.503150
H	2.781663	2.124885	-0.506030
H	0.105980	-2.409487	-0.047838
H	-0.122760	2.422926	-0.080176
H	-2.876632	-2.145189	-0.255483
H	-4.281146	0.195657	-0.101581
O	-2.415380	1.097827	0.053809
O	2.442687	-1.132847	0.127195

---

4 (X = O X' = NH X'' = O)
S0
E = -588.129985581 A.U.
G = -588.040696 A.U.

C	3.197280	-0.083511	-0.069611
C	1.106667	-0.614679	0.053675
C	1.130075	0.739431	0.017082
C	2.518584	1.088605	-0.068423
C	-1.103022	-0.703967	0.038886
C	-1.128182	0.651894	0.028535
N	-0.049019	1.550394	-0.000222
C	-2.454429	-1.154226	-0.032437
C	-3.197206	-0.020711	-0.070290
H	4.241308	-0.338818	-0.103731
H	2.942498	2.078547	-0.112274
H	-0.074898	2.199078	0.783829
H	-2.813060	-2.169916	-0.043214
H	-4.255765	0.166434	-0.105904
O	0.053470	-1.469115	0.077669
O	-2.397510	1.087301	-0.045759
O	2.329597	-1.143822	-0.007117

T1
E = -588.028568672 A.U.
G = -587.945281 A.U.

C	3.185894	-0.023858	-0.096962

C	1.117527	-0.614531	0.065665
C	1.095875	0.738004	-0.093054
C	2.475667	1.124796	-0.192566
C	-1.103878	-0.771444	0.166535
C	-1.157700	0.687777	0.501351
N	-0.111613	1.419029	-0.062703
C	-2.336796	-1.169247	-0.280136
C	-3.150261	-0.042438	-0.252197
H	4.237953	-0.245084	-0.123517
H	2.872540	2.118248	-0.320954
H	-0.137842	2.429494	-0.082459
H	-2.631876	-2.151901	-0.612268
H	-4.156025	0.118891	-0.599237
O	0.078605	-1.457396	0.175168
O	-2.457029	1.092585	0.167356
O	2.358246	-1.107340	0.065668

---

4 (X = O X' = O X'' = O)

S0

E = -607.982570065 A.U.

G = -607.906063 A.U.

C	3.172626	-0.032647	0.000348
C	1.095273	-0.633555	-0.000237
C	1.095355	0.719591	-0.000161
C	2.464158	1.123121	-0.000018
C	-1.095355	-0.719592	-0.000117
C	-1.095272	0.633554	-0.000093
C	-2.464158	-1.123121	0.000340
C	-3.172626	0.032647	0.000258
H	4.224206	-0.256359	0.000611
H	2.854955	2.126868	0.000040
H	-2.854955	-2.126868	0.000568
H	-4.224207	0.256358	0.000317
O	0.053077	-1.501887	-0.000332
O	2.335114	-1.118846	0.000030
O	-0.053076	1.501886	-0.000243
O	-2.335116	1.118846	0.000114

T1

E = -607.880732131 A.U.

G = -607.809230 A.U.

C	-3.093396	-0.015135	0.286604
C	-1.113199	-0.668644	-0.526546
C	-1.093693	0.799798	-0.183073
C	-2.313465	1.133065	0.343270
C	1.042620	-0.716540	0.102977
C	1.100477	0.624885	-0.075301
C	2.398604	-1.157354	0.218015
C	3.146689	-0.033038	0.110179
H	-4.078108	-0.231055	0.662302
H	-2.623644	2.084430	0.744951
H	2.754896	-2.163245	0.364386
H	4.204691	0.156135	0.140117
O	-0.131945	-1.425726	0.082797
O	-2.389382	-1.099392	-0.245819
O	0.079108	1.494860	-0.207841
O	2.354012	1.074196	-0.075200

4 (X = O X' = S X'' = NH)
S0
E = -911.114157264 A.U.
G = -911.030011 A.U.

C	3.309352	-0.332487	0.040074
C	1.171348	-0.753496	-0.024285
C	1.287352	0.599611	-0.000433
C	2.697416	0.872628	0.035490
C	-1.153287	-0.853603	-0.025965
C	-1.271391	0.498906	0.008363
C	-2.491454	-1.367358	-0.006340
C	-3.302719	-0.284381	0.039594
H	4.338029	-0.644679	0.075988
H	3.174023	1.838926	0.062397
H	-2.790111	-2.402991	-0.021444
H	-4.371246	-0.161772	0.074360
S	-0.049338	1.770259	-0.033174
N	0.055022	-1.578633	-0.120063
H	0.099071	-2.387396	0.491649
O	-2.572288	0.863097	0.040429
O	2.381637	-1.337438	-0.004267

T1
E = -911.015496644 A.U.
G = -910.935569 A.U.

C	-3.233654	-0.322634	0.305076
C	-1.197071	-0.748466	-0.540448
C	-1.283663	0.679577	-0.100427
C	-2.566755	0.872596	0.407719
C	1.107101	-0.850819	0.063061
C	1.278636	0.506537	-0.021271
C	2.427666	-1.405856	0.181637
C	3.272330	-0.350672	0.152819
H	-4.221609	-0.618446	0.615706
H	-2.977239	1.782196	0.818059
H	2.692196	-2.446972	0.269130
H	4.343665	-0.266363	0.211740
S	0.072105	1.750161	-0.167171
N	-0.117945	-1.469077	-0.026195
H	-0.166817	-2.479257	-0.012067
O	2.593374	0.824468	0.023803
O	-2.446598	-1.320939	-0.240486

4 (X = O X' = S X'' = O)
S0
E = -930.968643113 A.U.
G = -930.896708 A.U.

C	3.271044	-0.382546	0.000434
C	1.136288	-0.741740	-0.000228
C	1.273174	0.605494	-0.000070
C	2.691630	0.838822	0.000482
C	-1.116946	-0.837274	-0.000227
C	-1.255910	0.511801	-0.000062
C	-2.428974	-1.397504	0.000100
C	-3.266137	-0.332821	0.000384

H	4.291353	-0.722349	0.000649
H	3.193468	1.792475	0.000755
H	-2.692096	-2.442142	0.000139
H	-4.337671	-0.236273	0.000643
S	-0.047225	1.793239	-0.000343
O	0.057782	-1.553637	-0.000602
O	2.316498	-1.364278	0.000108
O	-2.564838	0.834300	0.000299

T1
E = -930.866656521 A.U.
G = -930.799221 A.U.

C	3.273309	-0.284044	0.047903
C	1.156409	-0.724523	0.021793
C	1.243506	0.627449	-0.110934
C	2.655653	0.909233	-0.086275
C	-1.099062	-0.906988	0.098098
C	-1.249091	0.500091	0.559412
C	-2.318324	-1.350464	-0.369607
C	-3.210248	-0.317894	-0.166893
H	4.304180	-0.584693	0.107483
H	3.126464	1.875375	-0.162073
H	-2.543805	-2.307724	-0.811536
H	-4.247678	-0.193567	-0.423269
S	-0.139613	1.688335	-0.217359
O	0.099704	-1.543357	0.046108
O	2.354407	-1.302612	0.120327
O	-2.593894	0.780981	0.434335

---

4 (X = O X' = S X'' = S)
S0
E = -1253.94838333 A.U.
G = -1253.879440 A.U.

C	-0.289213	3.219966	0.692244
C	0.492503	1.365393	-0.135780
C	-0.857487	1.224146	-0.100541
C	-1.372215	2.442838	0.454116
C	0.857487	-1.224146	-0.100541
C	-0.492503	-1.365393	-0.135780
C	1.372215	-2.442838	0.454116
C	0.289213	-3.219966	0.692244
H	-0.165224	4.216002	1.081020
H	-2.404166	2.693858	0.636486
H	2.404166	-2.693858	0.636486
H	0.165224	-4.216002	1.081020
S	1.733671	0.219827	-0.625724
S	-1.733671	-0.219827	-0.625724
O	0.857487	2.567667	0.354231
O	-0.857487	-2.567667	0.354231

T1
E = -1253.84736550 A.U.
G = -1253.783892 A.U.

C	-3.394373	-0.077460	0.019163
C	-1.325053	0.609313	0.004717
C	-1.274345	-0.752780	-0.111846
C	-2.644488	-1.193161	-0.104648

C	1.288395	0.803682	0.040790
C	1.293942	-0.589966	0.554763
C	2.580774	1.073253	-0.425814
C	3.340081	-0.032427	-0.164692
H	-4.454687	0.099688	0.069681
H	-2.997596	-2.208737	-0.176542
H	2.930842	1.979657	-0.894462
H	4.368923	-0.270377	-0.375330
S	-0.091794	1.832713	0.070155
S	0.150240	-1.750548	-0.196214
O	-2.604351	1.031982	0.095535
O	2.607825	-1.026681	0.469338

4 (X = S X' = NH X'' = NH)
S0
E = -1214.23240496 A.U.
G = -1214.139421 A.U.

C	1.326890	3.196532	-0.082222
C	1.121307	0.735543	0.070462
C	-0.087254	1.367012	0.051206
C	0.032231	2.787784	-0.039109
N	1.326890	-0.662060	0.061196
C	0.087254	-1.367012	0.051206
C	-1.121307	-0.735543	0.070462
N	-1.326890	0.662060	0.061196
C	-0.032231	-2.787784	-0.039109
C	-1.326890	-3.196532	-0.082222
H	1.713298	4.202502	-0.118926
H	-0.816619	3.458028	-0.055179
H	1.938989	-0.973493	0.809528
H	-1.938989	0.973493	0.809528
H	0.816619	-3.458028	-0.055179
H	-1.713298	-4.202502	-0.118926
S	-2.430562	-1.862267	-0.066613
S	2.430562	1.862267	-0.066613

T1
E = -1214.15150428 A.U.
G = -1214.063881 A.U.

C	-3.374034	-0.726933	0.130372
C	-1.250959	0.520221	-0.072992
C	-1.065744	-0.847465	0.064456
C	-2.302128	-1.555396	0.173092
N	-0.201432	1.398441	-0.133862
C	1.081281	0.898616	-0.103417
C	1.248406	-0.537289	-0.379181
N	0.208803	-1.364876	0.044725
C	2.258824	1.561170	0.200526
C	3.365747	0.725818	0.240518
H	-4.419615	-0.983765	0.187909
H	-2.372902	-2.629153	0.278922
H	-0.355381	2.390118	-0.010204
H	0.333376	-2.368040	0.064544
H	2.314383	2.623864	0.398829
H	4.373533	0.975979	0.529675
S	2.952487	-0.977519	-0.085770
S	-2.933321	0.947744	-0.060853

4 (X = S X' = NH X'' = O)

S0

E = -1234.08829058 A.U.

G = -1234.008479 A.U.

C	3.374509	0.645348	-0.085642
C	1.204587	-0.486773	0.061515
C	1.070907	0.867382	0.034725
C	2.337462	1.522240	-0.052607
C	-1.043034	-0.806905	0.062249
C	-1.225669	0.542979	0.061011
N	-0.199645	1.515677	0.051386
C	-2.249080	-1.556641	-0.013800
C	-3.336669	-0.744108	-0.073247
H	4.430354	0.856267	-0.139969
H	2.451645	2.597081	-0.084213
H	-0.279120	2.168580	0.826103
H	-2.284027	-2.636709	-0.009717
H	-4.378178	-1.020294	-0.109531
O	0.199954	-1.415326	0.112364
S	2.846714	-1.004432	-0.038258
S	-2.905519	0.932357	-0.068399

T1

E = -1233.99689596 A.U.

G = -1233.921612 A.U.

C	-3.341141	0.697556	0.180352
C	-1.217493	-0.487072	-0.112726
C	-1.038211	0.861393	0.082912
C	-2.285061	1.545227	0.243971
C	1.028253	-0.877268	-0.123720
C	1.240247	0.555557	-0.430956
N	0.237268	1.387771	0.044579
C	2.138309	-1.565243	0.288192
C	3.276487	-0.759873	0.352678
H	-4.389837	0.935662	0.259157
H	-2.372354	2.612011	0.394605
H	0.376352	2.386796	0.121280
H	2.129770	-2.612707	0.556818
H	4.238782	-1.020593	0.763617
O	-0.227389	-1.404472	-0.210428
S	-2.878859	-0.957260	-0.083835
S	2.964307	0.919669	-0.141686

4 (X = S X' = O X'' = O)

S0

E = -1253.94174256 A.U.

G = -1253.874123 A.U.

C	-1.795223	-2.888447	0.000025
C	-1.196422	-0.508488	-0.000017
C	-0.108629	-1.324394	-0.000014
C	-0.448297	-2.705095	0.000008
C	0.108629	1.324394	-0.000014
C	1.196422	0.508488	-0.000017
C	0.448297	2.705095	0.000008
C	1.795223	2.888447	0.000025

H	-2.343542	-3.816504	0.000045
H	0.283470	-3.500089	0.000004
H	-0.283470	3.500089	0.000004
H	2.343542	3.816504	0.000045
O	-1.196422	0.860975	-0.000036
O	1.196422	-0.860975	-0.000036
S	2.672184	1.394825	0.000014
S	-2.672184	-1.394825	0.000014

T1
E = -1253.82784768 A.U.
G = -1253.760182 A.U.

C	-0.568949	3.326338	0.193353
C	0.568949	1.169237	-0.244191
C	-0.823190	1.053794	0.040192
C	-1.457494	2.291499	0.236526
C	0.823190	-1.053794	0.040192
C	-0.568949	-1.169237	-0.244191
C	1.457494	-2.291499	0.236526
C	0.568949	-3.326338	0.193353
H	-0.773926	4.377532	0.318201
H	-2.519290	2.406999	0.400602
H	2.519290	-2.406999	0.400602
H	0.773926	-4.377532	0.318201
O	1.422363	0.148605	-0.022114
O	-1.422363	-0.148605	-0.022114
S	-1.094454	-2.828293	-0.118573
S	1.094454	2.828293	-0.118573

---

4 (X = S X' = S X'' = NH)
S0
E = -1557.07560349 A.U.
G = -1556.999405 A.U.

C	-3.404416	0.389004	-0.580245
C	-1.281753	-0.534164	0.308328
C	-1.237226	0.815864	0.110514
C	-2.457163	1.346389	-0.409769
C	1.064873	-0.879715	0.326259
C	1.354058	0.444736	0.162506
C	2.161930	-1.730210	-0.009656
C	3.251635	-1.022588	-0.412585
H	-4.422464	0.498404	-0.918521
H	-2.614739	2.394959	-0.621188
H	2.130705	-2.809398	0.054841
H	4.217494	-1.399593	-0.710863
S	0.218506	1.749563	0.538779
N	-0.208948	-1.312491	0.747663
H	-0.348314	-2.312956	0.713245
S	-2.817772	-1.187528	-0.155614
S	2.961036	0.677973	-0.428369

T1
E = -1556.98832576 A.U.
G = -1556.916309 A.U.

C	3.533487	-0.482346	-0.024698
C	1.294086	0.579359	-0.021735
C	1.241082	-0.798690	0.105046

C	2.540818	-1.395477	0.097240
C	-1.071577	0.966281	-0.010435
C	-1.331384	-0.437968	-0.372359
C	-2.215477	1.688528	0.331014
C	-3.374211	0.953841	0.240528
H	4.598068	-0.649845	-0.060721
H	2.708848	-2.460310	0.180346
H	-2.194631	2.727538	0.634898
H	-4.377247	1.255497	0.495468
S	-0.254430	-1.669145	0.279730
N	0.223386	1.422205	-0.001978
H	0.383383	2.412360	0.133949
S	2.933689	1.145986	-0.158642
S	-3.078200	-0.706955	-0.335944

4 (X = S X' = S X'' = O)
S0
E = -1576.92885727 A.U.
G = -1576.865468 A.U.

C	-3.423002	0.346834	-0.478557
C	-1.249124	-0.521711	0.263875
C	-1.232744	0.826542	0.096716
C	-2.497844	1.331200	-0.337645
C	1.027675	-0.863841	0.273039
C	1.339822	0.454626	0.135964
C	2.108080	-1.747161	-0.000150
C	3.234412	-1.062875	-0.333217
H	-4.457775	0.437826	-0.768245
H	-2.697069	2.377888	-0.519962
H	2.031247	-2.823117	0.062525
H	4.208831	-1.455890	-0.576989
S	0.222751	1.797128	0.413556
O	-0.219414	-1.334788	0.634509
S	-2.780638	-1.220628	-0.122762
S	2.984539	0.645995	-0.352891

T1
E = -1576.83182531 A.U.
G = -1576.772750 A.U.

C	-3.500502	0.464297	0.010508
C	-1.266103	-0.555216	-0.042608
C	-1.211876	0.800779	0.138484
C	-2.519849	1.386133	0.164314
C	1.014570	-0.952270	-0.019108
C	1.315754	0.442515	-0.420271
C	2.095798	-1.680527	0.419924
C	3.287397	-0.974068	0.337238
H	-4.566928	0.622064	-0.015703
H	-2.698509	2.445083	0.286825
H	2.021345	-2.692870	0.793070
H	4.256359	-1.268963	0.707301
S	0.292005	1.683844	0.266488
O	-0.253128	-1.441915	-0.063925
S	-2.884729	-1.146938	-0.182997
S	3.075324	0.640602	-0.382927

4 (X = S X' = S X'' = S)
S0
E = -1899.91451211 A.U.
G = -1899.854334 A.U.

C	3.305568	-0.725483	-0.779208
C	1.401025	0.475270	0.237675
C	1.229066	-0.879570	0.220917
C	2.324051	-1.575716	-0.373971
C	-1.229081	0.879640	0.220678
C	-1.401026	-0.475200	0.237761
C	-2.324051	1.575636	-0.374393
C	-3.305542	0.725304	-0.779486
H	4.252297	-0.968118	-1.236772
H	2.370552	-2.650562	-0.478473
H	-2.370559	2.650459	-0.479137
H	-4.252273	0.967823	-1.237108
S	0.238496	1.644005	0.866556
S	2.896604	0.927247	-0.498906
S	-2.896581	-0.927356	-0.498793
S	-0.238523	-1.643827	0.866871

T1
E = -1899.81495303 A.U.
G = -1899.759700 A.U.

C	-3.494457	0.657986	0.415044
C	-1.387545	-0.469803	-0.427135
C	-1.222669	0.918365	0.052727
C	-2.399913	1.475497	0.557408
C	1.224170	-0.862845	0.130314
C	1.423626	0.486236	-0.047160
C	2.455144	-1.594048	0.216301
C	3.540995	-0.795564	0.089000
H	-4.500708	0.839764	0.759687
H	-2.455002	2.460525	1.002701
H	2.502559	-2.665698	0.352255
H	4.582709	-1.075909	0.102320
S	-0.314408	-1.688388	0.219954
S	-3.119418	-0.859806	-0.422838
S	3.116184	0.864924	-0.137323
S	0.257287	1.779919	-0.168291

---

5 (X = NH X' = NH)
S0
E = -419.084911381 A.U.
G = -418.969271 A.U.

C	0.118132	0.031474	0.704339
C	0.118132	0.031474	-0.704339
N	0.275684	-1.204104	1.367975
C	-0.204289	-2.338513	0.664711
C	-0.204289	-2.338513	-0.664711
N	0.275684	-1.204104	-1.367975
C	-0.008575	1.232184	-1.388117
C	-0.105551	2.440738	-0.692692
C	-0.105551	2.440738	0.692692
C	-0.008575	1.232184	1.388117
H	0.007720	-1.176649	2.341853
H	-0.533488	-3.181302	1.253156

H	-0.533488	-3.181302	-1.253156
H	0.007720	-1.176649	-2.341853
H	-0.017607	1.221377	-2.472879
H	-0.184718	3.370006	-1.243114
H	-0.184718	3.370006	1.243114
H	-0.017607	1.221377	2.472879

T1
E = -418.998990122 A.U.
G = -418.889078 A.U.

C	-0.030174	-0.725352	0.000640
C	-0.030181	0.725369	-0.000109
N	1.187849	-1.363288	-0.000438
C	2.379903	-0.708954	-0.000894
C	2.379874	0.708956	0.000590
N	1.187837	1.363317	0.000548
C	-1.258502	1.407409	-0.000650
C	-2.449919	0.718815	-0.000461
C	-2.449917	-0.718849	0.000267
C	-1.258473	-1.407410	0.000671
H	1.192771	-2.374332	-0.001390
H	3.280383	-1.298388	-0.001292
H	3.280391	1.298310	0.001049
H	1.192746	2.374361	0.001198
H	-1.249121	2.493531	-0.001242
H	-3.386789	1.260889	-0.000872
H	-3.386774	-1.260938	0.000387
H	-1.249069	-2.493533	0.001072

---

5 (X = NH X' = O)
S0
E = -438.943615673 A.U.
G = -438.840756 A.U.

C	-0.016424	0.713794	-0.092439
C	-0.024647	-0.689268	-0.082375
N	1.215931	1.379675	-0.205868
C	2.342879	0.616017	0.150078
C	2.298006	-0.709752	0.149894
C	-1.213058	-1.388092	0.015352
C	-2.425911	-0.700455	0.083903
C	-2.428999	0.686858	0.075706
C	-1.227571	1.390541	-0.004808
H	1.216621	2.331564	0.132374
H	3.244913	1.151084	0.406031
H	3.129293	-1.353242	0.388904
H	-1.173325	-2.470643	0.025457
H	-3.354426	-1.253415	0.146614
H	-3.362636	1.232532	0.132637
H	-1.223623	2.474963	-0.009284
O	1.148250	-1.411052	-0.194190

T1
E = -438.847367049 A.U.
G = -438.748434 A.U.

C	0.004539	0.711674	0.037770
C	0.018867	-0.697232	-0.045201
N	-1.206977	1.360984	-0.022441

C	-2.361827	0.644258	-0.243524
C	-2.286898	-0.729772	0.295093
C	1.225435	-1.389043	-0.091336
C	2.425156	-0.697983	-0.049187
C	2.421736	0.700339	0.054635
C	1.229814	1.396770	0.096936
H	-1.206570	2.368283	-0.114564
H	-3.290952	1.187016	-0.297056
H	-3.151156	-1.376027	0.317338
H	1.192567	-2.470313	-0.149787
H	3.360991	-1.240498	-0.085551
H	3.357875	1.242994	0.096347
H	1.220282	2.479017	0.163280
O	-1.136891	-1.418928	-0.013005

---

5 (X = NH X' = S)
S0
E = -761.924502323 A.U.
G = -761.824911 A.U.

C	0.312107	0.790421	-0.167648
C	0.176208	-0.598912	-0.057420
N	-0.826732	1.596513	-0.336854
C	-2.021826	1.232665	0.293364
C	-2.405190	-0.034606	0.428099
C	1.294512	-1.397112	0.153452
C	2.564428	-0.829001	0.205521
C	2.704336	0.548536	0.076780
C	1.584870	1.356416	-0.087699
H	-0.631351	2.587004	-0.320180
H	-2.611067	2.048050	0.695084
H	-3.292539	-0.321770	0.971688
H	1.168349	-2.468809	0.255109
H	3.433083	-1.459669	0.345568
H	3.686475	1.003333	0.118164
H	1.691205	2.433136	-0.163279
S	-1.432107	-1.337957	-0.288179

T1
E = -761.830672832 A.U.
G = -761.736092 A.U.

C	0.297366	0.814425	-0.013503
C	0.168689	-0.592268	-0.038958
N	-0.791630	1.649537	-0.105295
C	-2.113451	1.283963	-0.023376
C	-2.407255	-0.071548	0.425945
C	1.312905	-1.395217	-0.012533
C	2.576575	-0.834803	0.040804
C	2.709271	0.561358	0.066285
C	1.593692	1.369720	0.044463
H	-0.590570	2.616752	-0.322532
H	-2.811464	1.889807	-0.590600
H	-3.417074	-0.360776	0.678176
H	1.192858	-2.473020	-0.027479
H	3.451572	-1.470984	0.070454
H	3.693131	1.012721	0.105689
H	1.694664	2.449720	0.063786
S	-1.406154	-1.376548	-0.135949

———————————————————————————————————

5 (X = O X' = O)

S0

E = -458.800934329 A.U.

G = -458.711215 A.U.

C	-0.000023	0.009166	0.698781
C	-0.000023	0.009166	-0.698781
C	0.000088	-2.306286	0.661818
C	0.000088	-2.306286	-0.661818
C	-0.000023	1.208808	-1.389756
C	-0.000023	2.416116	-0.694410
C	-0.000023	2.416116	0.694410
C	-0.000023	1.208808	1.389756
H	0.000169	-3.209999	1.250046
H	0.000169	-3.209999	-1.250046
H	-0.000023	1.174539	-2.472423
H	-0.000025	3.349308	-1.242939
H	-0.000025	3.349308	1.242939
H	-0.000023	1.174539	2.472423
O	-0.000030	-1.160084	1.427752
O	-0.000030	-1.160084	-1.427752

T1

E = -458.678965486 A.U.

G = -458.593393 A.U.

C	0.003109	-0.738916	-0.098678
C	0.012856	0.725004	-0.321378
C	2.307716	-0.618488	-0.041170
C	2.256792	0.698754	0.225966
C	-1.269737	1.408312	-0.198521
C	-2.393058	0.694322	0.028549
C	-2.374243	-0.750102	0.174027
C	-1.156546	-1.437758	0.110958
H	3.223805	-1.184687	-0.081810
H	3.128557	1.295355	0.440933
H	-1.289600	2.485151	-0.315673
H	-3.346179	1.206745	0.095023
H	-3.295673	-1.284713	0.354229
H	-1.106677	-2.515850	0.211199
O	1.194699	-1.379125	-0.185911
O	1.100855	1.393028	0.188109

———————————————————————————————————

5 (X = O X' = S)

S0

E = -781.778455489 A.U.

G = -781.692174 A.U.

C	0.308803	0.778114	-0.153268
C	0.158796	-0.604149	-0.063729
C	-1.955146	1.277655	0.258551
C	-2.387534	0.032180	0.382018
C	1.284582	-1.402679	0.118796
C	2.549305	-0.827434	0.178310
C	2.689457	0.553366	0.081530
C	1.566264	1.359081	-0.067866
H	-2.501906	2.138618	0.619540
H	-3.321076	-0.194363	0.876018

H	1.164945	-2.476495	0.202401
H	3.419969	-1.458373	0.304190
H	3.670880	1.007878	0.131559
H	1.642853	2.437321	-0.133506
S	-1.452742	-1.340932	-0.231606
O	-0.764868	1.625439	-0.337570

T1
E = -781.678637890 A.U.
G = -781.597160 A.U.

C	-0.306562	0.807519	0.037848
C	-0.140082	-0.579551	-0.058614
C	1.967197	1.253264	-0.357967
C	2.443991	0.013634	0.248696
C	-1.274493	-1.396910	-0.121923
C	-2.544695	-0.852403	-0.053188
C	-2.701001	0.529027	0.083767
C	-1.588849	1.350806	0.125096
H	2.621646	2.057366	-0.658107
H	3.231787	-0.054520	0.988243
H	-1.143419	-2.470152	-0.203832
H	-3.410672	-1.500564	-0.094555
H	-3.690755	0.963566	0.144998
H	-1.678110	2.427280	0.204966
S	1.448064	-1.364667	0.010149
O	0.720933	1.707424	0.004201

---

5 (X = S X' = S)
S0
E = -1104.75495078 A.U.
G = -1104.672463 A.U.

C	-0.122303	0.397756	0.697431
C	-0.122303	0.397756	-0.697431
C	0.597662	-2.230364	0.664179
C	0.597662	-2.230364	-0.664179
C	0.059669	1.593301	-1.392207
C	0.197633	2.787660	-0.696058
C	0.197633	2.787660	0.696058
C	0.059669	1.593301	1.392207
H	1.218871	-2.909717	1.234757
H	1.218871	-2.909717	-1.234757
H	0.082343	1.582559	-2.475370
H	0.318585	3.714917	-1.242014
H	0.318585	3.714917	1.242014
H	0.082343	1.582559	2.475370
S	-0.375985	-1.104867	-1.621663
S	-0.375985	-1.104867	1.621663

T1
E = -1104.65824831 A.U.
G = -1104.582549 A.U.

C	0.381068	-0.698303	-0.042052
C	0.381061	0.698297	0.041995
C	-2.342647	-0.652250	0.314999
C	-2.342788	0.652279	-0.314577
C	1.604434	1.379110	0.113305
C	2.804803	0.695339	0.062891

C	2.804811	-0.695333	-0.062801
C	1.604447	-1.379111	-0.113273
H	-3.142011	-1.023676	0.942403
H	-3.141970	1.023336	-0.942442
H	1.597748	2.461208	0.188039
H	3.737680	1.242754	0.110317
H	3.737694	-1.242744	-0.110164
H	1.597769	-2.461210	-0.187984
S	-1.054936	1.747382	0.007812
S	-1.054942	-1.747372	-0.008005

---

6 (X = NH X' = NH)
S0
E = -572.724443325 A.U.
G = -572.564469 A.U.

C	-3.575305	0.693153	-0.305709
C	-3.575305	-0.693153	-0.305709
C	-2.386712	-1.389964	-0.076147
C	-1.207434	-0.703174	0.175201
C	-1.207434	0.703174	0.175201
C	-2.386712	1.389964	-0.076147
N	0.000000	-1.356311	0.471288
C	1.207434	-0.703174	0.175201
C	1.207434	0.703174	0.175201
N	0.000000	1.356311	0.471288
C	2.386712	-1.389964	-0.076147
C	3.575305	-0.693153	-0.305709
C	3.575305	0.693153	-0.305709
C	2.386712	1.389964	-0.076147
H	-4.490458	1.242987	-0.486458
H	-4.490458	-1.242987	-0.486458
H	-2.375093	-2.474430	-0.079927
H	-2.375093	2.474430	-0.079927
H	0.000000	-2.354791	0.313625
H	0.000000	2.354791	0.313625
H	2.375093	-2.474430	-0.079927
H	4.490458	-1.242987	-0.486458
H	4.490458	1.242987	-0.486458
H	2.375093	2.474430	-0.079927

T1
E = -572.622448638 A.U.
G = -572.468653 A.U.

C	3.624112	0.720473	-0.000001
C	3.624112	-0.720473	-0.000001
C	2.438378	-1.410892	-0.000016
C	1.207746	-0.726112	-0.000065
C	1.207746	0.726112	-0.000065
C	2.438378	1.410892	-0.000016
N	0.000000	-1.364815	0.000093
C	-1.207746	-0.726112	-0.000065
C	-1.207746	0.726112	-0.000065
N	0.000000	1.364815	0.000093
C	-2.438378	-1.410892	-0.000016
C	-3.624112	-0.720473	-0.000001
C	-3.624112	0.720473	-0.000001
C	-2.438378	1.410892	-0.000016

H	4.562607	1.259242	-0.000023
H	4.562607	-1.259242	-0.000023
H	2.429199	-2.496420	-0.000032
H	2.429199	2.496420	-0.000032
H	0.000000	-2.377979	0.000440
H	0.000000	2.377979	0.000440
H	-2.429199	-2.496420	-0.000032
H	-4.562607	-1.259242	-0.000023
H	-4.562607	1.259242	-0.000023
H	-2.429199	2.496420	-0.000032

6 (X = NH X' = O)

S0

E = -592.581840473 A.U.

G = -592.435509 A.U.

C	-0.746589	-0.253554	3.542394
C	0.641485	-0.258891	3.577149
C	1.375612	-0.067277	2.408347
C	0.725320	0.145567	1.197064
C	-0.674634	0.149036	1.174667
C	-1.405940	-0.056559	2.328829
N	1.408083	0.377345	0.000000
C	0.725320	0.145567	-1.197064
C	-0.674634	0.149036	-1.174667
C	1.375612	-0.067277	-2.408347
C	0.641485	-0.258891	-3.577149
C	-0.746589	-0.253554	-3.542394
C	-1.405940	-0.056559	-2.328829
H	-1.320740	-0.403541	4.447580
H	1.163879	-0.414107	4.512870
H	2.459601	-0.073461	2.429104
H	-2.487106	-0.047991	2.263334
H	2.397652	0.173551	0.000000
H	2.459601	-0.073461	-2.429104
H	1.163879	-0.414107	-4.512870
H	-1.320740	-0.403541	-4.447580
H	-2.487106	-0.047991	-2.263334
O	-1.358568	0.395419	-0.000000

T1

E = -592.465370992 A.U.

G = -592.323246 A.U.

C	3.548256	-0.771384	-0.018162
C	3.601008	0.671221	-0.183673
C	2.416060	1.403892	-0.166797
C	1.205120	0.767576	0.045401
C	1.146685	-0.686867	0.327736
C	2.387922	-1.428950	0.207531
N	-0.001284	1.403574	0.083771
C	-1.193034	0.713970	0.080518
C	-1.165173	-0.688004	-0.059813
C	-2.434373	1.370680	0.125948
C	-3.603305	0.645830	0.026354
C	-3.565758	-0.750078	-0.124508
C	-2.351701	-1.410920	-0.163091
H	4.474357	-1.333052	-0.072330
H	4.549710	1.161281	-0.348424

H	2.425395	2.481167	-0.298839
H	2.355667	-2.504680	0.332407
H	-0.030709	2.412477	-0.007250
H	-2.452994	2.449218	0.232639
H	-4.555051	1.160475	0.060911
H	-4.486850	-1.312811	-0.203046
H	-2.288515	-2.486984	-0.267592
O	0.008467	-1.359238	-0.074941

---

6 (X = NH X' = S)
S0
E = -915.557787619 A.U.
G = -915.414402 A.U.

C	0.356653	-0.594291	3.639086
C	-1.015692	-0.400663	3.517282
C	-1.562901	0.017812	2.310379
C	-0.737374	0.283943	1.216888
C	0.644426	0.104109	1.348562
C	1.183152	-0.353293	2.546026
N	-1.272621	0.731956	-0.000000
C	-0.737374	0.283943	-1.216888
C	0.644426	0.104109	-1.348562
C	-1.562901	0.017812	-2.310379
C	-1.015692	-0.400663	-3.517282
C	0.356653	-0.594291	-3.639086
C	1.183152	-0.353293	-2.546026
H	0.783465	-0.932687	4.574562
H	-1.669391	-0.588993	4.360073
H	-2.634700	0.147928	2.208338
H	2.253863	-0.502380	2.622838
H	-2.281933	0.797580	-0.000000
H	-2.634700	0.147928	-2.208338
H	-1.669391	-0.588993	-4.360073
H	0.783465	-0.932687	-4.574562
H	2.253863	-0.502380	-2.622838
S	1.706540	0.571224	0.000000

T1
E = -915.446132300 A.U.
G = -915.308224 A.U.

C	3.745360	-0.432073	0.174314
C	3.639440	0.978930	-0.152640
C	2.385800	1.560049	-0.234989
C	1.231468	0.807433	-0.028857
C	1.318885	-0.635193	0.269523
C	2.645958	-1.201416	0.361689
N	-0.006254	1.393142	-0.007562
C	-1.225143	0.767573	0.044285
C	-1.358117	-0.636708	-0.064824
C	-2.395813	1.555047	0.142322
C	-3.637813	0.969807	0.134154
C	-3.767194	-0.429382	0.027004
C	-2.638410	-1.212904	-0.074400
H	4.729374	-0.873967	0.282440
H	4.531643	1.568147	-0.309873
H	2.284059	2.624123	-0.427935
H	2.742768	-2.255307	0.600608

H	-0.030241	2.403436	-0.085608
H	-2.292180	2.631666	0.221411
H	-4.521411	1.590904	0.210818
H	-4.746849	-0.889023	0.030398
H	-2.723441	-2.290740	-0.154580
S	0.025221	-1.675513	-0.243764

---

6 (X = O X' = O)

S0

E = -612.438259163 A.U.

G = -612.304890 A.U.

C	3.571942	0.695194	-0.000012
C	3.571942	-0.695194	-0.000012
C	2.366614	-1.390649	-0.000003
C	1.166987	-0.697122	0.000008
C	1.166987	0.697122	0.000008
C	2.366614	1.390649	-0.000003
C	-1.166987	-0.697122	0.000008
C	-1.166987	0.697122	0.000008
C	-2.366614	-1.390649	-0.000003
C	-3.571942	-0.695194	-0.000012
C	-3.571942	0.695194	-0.000012
C	-2.366614	1.390649	-0.000003
H	4.505339	1.243224	-0.000021
H	4.505339	-1.243224	-0.000021
H	2.331680	-2.473198	-0.000004
H	2.331680	2.473198	-0.000004
H	-2.331680	-2.473198	-0.000004
H	-4.505339	-1.243224	-0.000021
H	-4.505339	1.243224	-0.000021
H	-2.331680	2.473198	-0.000004
O	0.000000	-1.423816	0.000017
O	-0.000000	1.423816	0.000017

T1

E = -612.300905069 A.U.

G = -612.168914 A.U.

C	3.556857	0.035489	-0.721676
C	3.556857	-0.035489	0.721676
C	2.376491	-0.067075	1.416684
C	1.158024	-0.041369	0.721837
C	1.158024	0.041369	-0.721837
C	2.376491	0.067075	-1.416684
C	-1.158024	0.041369	0.721837
C	-1.158024	-0.041369	-0.721837
C	-2.376491	0.067075	1.416684
C	-3.556857	0.035489	0.721676
C	-3.556857	-0.035489	-0.721676
C	-2.376491	-0.067075	-1.416684
H	4.497642	0.062559	-1.254899
H	4.497642	-0.062559	1.254899
H	2.347828	-0.112206	2.498916
H	2.347828	0.112206	-2.498916
H	-2.347828	0.112206	2.498916
H	-4.497642	0.062559	1.254899
H	-4.497642	-0.062559	-1.254899
H	-2.347828	-0.112206	-2.498916

O	-0.000000	0.000000	1.399463
O	0.000000	-0.000000	-1.399463

---

6 (X = O X' = S)
S0
E = -935.412685789 A.U.
G = -935.282425 A.U.

C	0.282881	-0.601012	3.606868
C	-1.084174	-0.398176	3.441929
C	-1.583309	0.041089	2.220761
C	-0.706077	0.302931	1.178158
C	0.664098	0.107170	1.334828
C	1.155175	-0.360023	2.551030
C	-0.706077	0.302931	-1.178158
C	0.664098	0.107170	-1.334828
C	-1.583309	0.041089	-2.220761
C	-1.084174	-0.398176	-3.441929
C	0.282881	-0.601012	-3.606868
C	1.155175	-0.360023	-2.551030
H	0.672429	-0.952386	4.553875
H	-1.766559	-0.590636	4.260207
H	-2.642651	0.198520	2.060636
H	2.219852	-0.523998	2.667600
H	-2.642651	0.198520	-2.060636
H	-1.766559	-0.590636	-4.260207
H	0.672429	-0.952386	-4.553875
H	2.219852	-0.523998	-2.667600
S	1.760329	0.521256	-0.000000
O	-1.234315	0.786645	0.000000

T1
E = -935.291154253 A.U.
G = -935.166147 A.U.

C	3.728021	-0.431842	-0.065747
C	3.605549	0.945509	0.158575
C	2.357284	1.529900	0.232959
C	1.206296	0.749003	0.087882
C	1.318978	-0.631508	-0.118643
C	2.594624	-1.208489	-0.200925
C	-1.175037	0.810134	0.018100
C	-1.288764	-0.606847	0.421601
C	-2.266495	1.549572	-0.358774
C	-3.544429	0.977718	-0.331782
C	-3.710029	-0.375604	0.152931
C	-2.654555	-1.132441	0.523211
H	4.705974	-0.891664	-0.123653
H	4.490537	1.559241	0.269030
H	2.232699	2.593985	0.390257
H	2.675659	-2.277782	-0.358195
H	-2.118501	2.587736	-0.633805
H	-4.407218	1.553361	-0.634638
H	-4.712834	-0.778104	0.241866
H	-2.792024	-2.138221	0.904308
S	-0.084858	-1.669565	-0.242833
O	0.031848	1.431733	0.089229

---

6 (X = S X' = S)

S0

E = -1258.38683057 A.U.

G = -1258.259480 A.U.

C	3.564288	0.696037	-0.794670
C	3.564288	-0.696037	-0.794670
C	2.459474	-1.393153	-0.321027
C	1.364603	-0.698520	0.191474
C	1.364603	0.698520	0.191474
C	2.459474	1.393153	-0.321027
C	-1.364603	-0.698520	0.191474
C	-1.364603	0.698520	0.191474
C	-2.459474	-1.393153	-0.321027
C	-3.564288	-0.696037	-0.794670
C	-3.564288	0.696037	-0.794670
C	-2.459474	1.393153	-0.321027
H	4.418238	1.241481	-1.176476
H	4.418238	-1.241481	-1.176476
H	2.443246	-2.476245	-0.338323
H	2.443246	2.476245	-0.338323
H	-2.443246	-2.476245	-0.338323
H	-4.418238	-1.241481	-1.176476
H	-4.418238	1.241481	-1.176476
H	-2.443246	2.476245	-0.338323
S	0.000000	-1.607321	0.882517
S	0.000000	1.607321	0.882517

T1

E = -1258.23988140 A.U.

G = -1258.118870 A.U.

C	-0.025191	3.834157	0.690277
C	-0.025191	3.834157	-0.690277
C	0.023602	2.628936	-1.441877
C	0.020365	1.395407	-0.697058
C	0.020365	1.395407	0.697058
C	0.023602	2.628936	1.441877
C	0.000523	-1.406538	-0.701950
C	0.000523	-1.406538	0.701950
C	-0.003141	-2.634533	-1.391634
C	-0.006777	-3.824785	-0.701760
C	-0.006777	-3.824785	0.701760
C	-0.003141	-2.634533	1.391634
H	-0.062255	4.780170	1.219156
H	-0.062255	4.780170	-1.219156
H	0.031488	2.613709	-2.521736
H	0.031488	2.613709	2.521736
H	-0.002629	-2.628545	-2.475411
H	-0.009322	-4.760349	-1.245842
H	-0.009322	-4.760349	1.245842
H	-0.002629	-2.628545	2.475411
S	-0.000848	0.002447	-1.716343
S	-0.000848	0.002447	1.716343

---

7 (X = NH X' = NH X'' = NH)

S0

E = -550.641517153 A.U.

G = -550.499820 A.U.

C	-3.337930	-0.716350	0.115494
C	-3.353125	0.671851	0.125130
C	-2.157094	1.374903	0.020009
C	-0.932241	0.718120	-0.091225
C	-0.919753	-0.694667	-0.096880
C	-2.122968	-1.386251	0.002351
N	0.254166	1.470355	-0.231403
C	1.449430	0.732554	-0.037827
C	1.436021	-0.633437	-0.033215
N	0.293197	-1.430205	-0.226240
C	2.805630	1.147948	0.070546
C	3.566358	0.002650	0.143041
N	2.721063	-1.080197	0.062570
H	-4.258332	-1.280980	0.195257
H	-4.287096	1.212956	0.213060
H	-2.159409	2.459825	0.024323
H	-2.095698	-2.470816	-0.007003
H	0.223408	2.351500	0.265073
H	0.284333	-2.277139	0.331031
H	3.174239	2.161176	0.101939
H	4.628058	-0.133139	0.257386
H	3.005545	-2.046978	0.079901

T1
E = -550.543601176 A.U.
G = -550.407243 A.U.

C	3.352541	-0.737889	-0.120392
C	3.348099	0.700818	-0.066462
C	2.196863	1.405014	0.097492
C	0.927762	0.723443	0.271170
C	0.934910	-0.727290	0.063870
C	2.115565	-1.419047	-0.083548
N	-0.268805	1.417286	-0.022815
C	-1.450716	0.754900	-0.036637
C	-1.437367	-0.644720	0.067083
N	-0.297321	-1.375379	0.087824
C	-2.816776	1.156553	-0.125123
C	-3.564215	0.009400	-0.073923
N	-2.728690	-1.084735	0.041571
H	4.276913	-1.283560	-0.247333
H	4.288652	1.235505	-0.149810
H	2.210617	2.489722	0.142365
H	2.079878	-2.502164	-0.155533
H	-0.250288	2.425100	-0.090746
H	-0.329128	-2.383481	0.009729
H	-3.193432	2.162615	-0.208673
H	-4.632355	-0.125190	-0.102029
H	-3.027140	-2.045844	0.094779

---

7 (X = NH X' = NH X'' = O)
S0
E = -570.494359745 A.U.
G = -570.364728 A.U.

C	-3.325935	-0.717641	0.078886
C	-3.343900	0.670478	0.091516
C	-2.147975	1.376394	0.012031
C	-0.920899	0.721480	-0.070012

C	-0.906079	-0.690514	-0.077234
C	-2.108536	-1.385753	-0.010520
N	0.267331	1.487276	-0.178789
C	1.451424	0.734392	-0.015818
C	1.430513	-0.614587	-0.005776
N	0.312970	-1.430516	-0.173194
C	2.835123	1.105824	0.040970
C	3.526766	-0.060153	0.091516
H	-4.247028	-1.283591	0.136982
H	-4.280698	1.209282	0.159967
H	-2.152031	2.461092	0.015344
H	-2.078166	-2.469917	-0.023635
H	0.236841	2.340924	0.364397
H	0.317477	-2.258963	0.412510
H	3.251195	2.100256	0.057586
H	4.572105	-0.299522	0.173803
O	2.671898	-1.129549	0.044197

T1
E = -570.406222836 A.U.
G = -570.280887 A.U.

C	3.304398	-0.746419	-0.122928
C	3.343802	0.648785	-0.017991
C	2.167843	1.370914	0.078572
C	0.927724	0.721097	0.066116
C	0.887230	-0.692096	-0.038783
C	2.087429	-1.405757	-0.123935
N	-0.258830	1.439452	0.104633
C	-1.460381	0.792838	0.116091
C	-1.448296	-0.649363	0.456942
N	-0.343465	-1.333633	-0.037658
C	-2.751417	1.125765	-0.245565
C	-3.502284	-0.041478	-0.193735
H	4.222461	-1.315145	-0.197459
H	4.293330	1.168878	-0.012116
H	2.187300	2.452159	0.158941
H	2.045046	-2.487031	-0.195230
H	-0.222172	2.434450	-0.067587
H	-0.355234	-2.345753	-0.033978
H	-3.123513	2.095788	-0.536631
H	-4.511358	-0.259774	-0.496895
O	-2.707011	-1.153753	0.132928

---

7 (X = NH X' = NH X'' = S)
S0
E = -893.475596168 A.U.
G = -893.350340 A.U.

C	3.534087	-0.825820	-0.274339
C	3.623253	0.557750	-0.291844
C	2.481425	1.329487	-0.077172
C	1.252059	0.731894	0.173257
C	1.163841	-0.675022	0.190758
C	2.301480	-1.436154	-0.041365
N	0.101453	1.505285	0.446010
C	-1.127545	0.866545	0.158993
C	-1.196162	-0.491952	0.180658
N	-0.080168	-1.290104	0.485887

C	-2.382562	1.476745	-0.133840
C	-3.370708	0.559700	-0.319800
H	4.411261	-1.437666	-0.443104
H	4.572215	1.046029	-0.475116
H	2.540950	2.412386	-0.095641
H	2.219325	-2.517655	-0.030638
H	0.164130	2.457245	0.109513
H	-0.140355	-2.251187	0.175814
H	-2.535398	2.545936	-0.194493
H	-4.411522	0.726720	-0.545088
S	-2.790288	-1.065445	-0.151023

T1
E = -893.394306314 A.U.
G = -893.272956 A.U.

C	3.564388	-0.854928	0.138668
C	3.674410	0.535974	0.006615
C	2.537921	1.315203	-0.104375
C	1.267139	0.726501	-0.084265
C	1.156171	-0.679193	0.042789
C	2.318542	-1.453643	0.150389
N	0.116906	1.495312	-0.130405
C	-1.129346	0.930967	-0.093944
C	-1.200944	-0.513970	-0.379326
N	-0.101616	-1.251169	0.032154
C	-2.345144	1.506155	0.216591
C	-3.395228	0.591468	0.248367
H	4.453607	-1.466115	0.225382
H	4.649440	1.006104	-0.007153
H	2.611991	2.392527	-0.202360
H	2.222278	-2.529790	0.241858
H	0.208742	2.495806	-0.015162
H	-0.163802	-2.260216	0.067629
H	-2.475235	2.558514	0.432988
H	-4.406760	0.763367	0.579387
S	-2.868421	-1.081025	-0.092742

---

7 (X = NH X' = O X'' = NH)
S0
E = -570.501291318 A.U.
G = -570.371775 A.U.

C	3.332954	0.672162	-0.012889
C	3.318110	-0.717997	-0.001335
C	2.102541	-1.391729	0.003488
C	0.903069	-0.691735	0.002042
C	0.906572	0.715074	-0.017182
C	2.133914	1.374312	-0.029571
C	-1.408686	-0.712164	0.025912
C	-1.426530	0.652102	0.022857
N	-0.294481	1.480678	-0.042744
C	-2.744995	-1.182547	-0.007782
C	-3.541376	-0.059590	-0.025755
H	4.271178	1.212466	-0.015917
H	4.243622	-1.279671	0.005152
H	2.055705	-2.473999	0.019010
H	2.130676	2.458575	-0.052347
H	-0.285928	2.219758	0.653046

H	-3.075559	-2.208342	-0.009548
H	-4.613021	0.040893	-0.030529
O	-0.245552	-1.455174	0.020060
N	-2.729814	1.050297	-0.021809
H	-3.045629	2.007558	-0.036197

T1
E = -570.390632029 A.U.
G = -570.266735 A.U.

C	-3.311640	0.690095	-0.093861
C	-3.303254	-0.740170	-0.210301
C	-2.066504	-1.420814	-0.126760
C	-0.912035	-0.724613	0.095470
C	-0.905392	0.715597	0.353218
C	-2.176413	1.396610	0.152621
C	1.407052	-0.716256	0.099438
C	1.414639	0.679157	-0.055609
N	0.287202	1.396724	0.002494
C	2.743975	-1.169289	0.059842
C	3.519670	-0.046469	-0.113246
H	-4.252691	1.220822	-0.192803
H	-4.216063	-1.284214	-0.407066
H	-2.006755	-2.499514	-0.221266
H	-2.204080	2.477002	0.252618
H	0.287460	2.404390	-0.100655
H	3.091058	-2.185695	0.138088
H	4.589107	0.049746	-0.195338
O	0.273677	-1.431580	0.167491
N	2.715723	1.070188	-0.186538
H	3.041484	2.018623	-0.290058

---

7 (X = NH X' = O X'' = O)
S0
E = -590.352305411 A.U.
G = -590.235030 A.U.

C	-3.316747	-0.672336	-0.014683
C	-3.304612	0.717220	0.002042
C	-2.090578	1.394354	0.008708
C	-0.892407	0.695456	0.004029
C	-0.892778	-0.711384	-0.018502
C	-2.116917	-1.373665	-0.033421
C	1.408688	0.712787	0.022286
C	1.420665	-0.634664	0.026776
N	0.313828	-1.475508	-0.046172
C	2.770323	1.142736	-0.016989
C	3.498626	-0.001748	-0.025897
H	-4.254009	-1.214055	-0.019664
H	-4.231214	1.276826	0.010670
H	-2.045546	2.476505	0.026818
H	-2.111970	-2.457610	-0.059146
H	0.317956	-2.228066	0.635734
H	3.146465	2.152296	-0.025340
H	4.554582	-0.205594	-0.027617
O	0.259726	1.464645	0.020038
O	2.680445	-1.100181	-0.013081

T1

```
E = -590.241701060 A.U.
G = -590.128949 A.U.
C        -3.304906    0.698403   -0.191490
C        -3.276864   -0.732974   -0.056257
C        -2.122487   -1.414324    0.173982
C        -0.873940   -0.707589    0.344581
C        -0.908369    0.738947    0.076213
C        -2.101179    1.403039   -0.136849
C         1.398917   -0.739422   -0.076264
C         1.416510    0.635922    0.096108
N         0.316773    1.397726    0.137070
C         2.761242   -1.141037   -0.192073
C         3.481792    0.002668   -0.085820
H        -4.237918    1.215677   -0.363627
H        -4.208204   -1.284321   -0.130619
H        -2.116694   -2.493269    0.279273
H        -2.085511    2.483706   -0.242070
H         0.376966    2.408623    0.080540
H         3.147991   -2.136709   -0.329615
H         4.537422    0.208919   -0.104097
O         0.270737   -1.435526   -0.073408
O         2.672294    1.104462    0.090651
```

```
7 (X = NH X' = O X'' = S)
S0
E = -913.334027371 A.U.
G = -913.221647 A.U.
C        -3.576959   -0.774328    0.077833
C        -3.631397    0.613550    0.085090
C        -2.450601    1.347883    0.021540
C        -1.226805    0.703141   -0.051838
C        -1.160015   -0.699012   -0.066538
C        -2.347856   -1.421559   -0.001076
C         1.093945    0.835843   -0.034184
C         1.186357   -0.519184   -0.033068
N         0.082191   -1.371504   -0.170528
C         2.350400    1.498383    0.038121
C         3.380433    0.613054    0.087297
H        -4.486105   -1.359968    0.129483
H        -4.581874    1.128272    0.142668
H        -2.455133    2.431082    0.032628
H        -2.295978   -2.504552   -0.016645
H         0.127001   -2.223014    0.375962
H         2.461176    2.573258    0.058948
H         4.436757    0.812831    0.164835
O        -0.104700    1.509240   -0.119560
S         2.834463   -1.032371    0.032702

T1
E = -913.242427367 A.U.
G = -913.133799 A.U.
C        -3.532931   -0.831682    0.197283
C        -3.633013    0.548666   -0.001972
C        -2.485647    1.310265   -0.157483
C        -1.238278    0.699615   -0.119325
C        -1.125623   -0.690312    0.062819
C        -2.293785   -1.445905    0.224388
```

C	1.077410	0.912057	-0.116499
C	1.194407	-0.527362	-0.441945
N	0.131655	-1.272818	0.031791
C	2.223956	1.508240	0.325962
C	3.307127	0.621117	0.383451
H	-4.426702	-1.429049	0.324383
H	-4.603011	1.027903	-0.028409
H	-2.526936	2.383264	-0.298219
H	-2.208257	-2.517613	0.362767
H	0.201567	-2.280220	0.099210
H	2.284576	2.544664	0.628305
H	4.267179	0.796952	0.842448
O	-0.134419	1.510354	-0.208813
S	2.887726	-1.020451	-0.163911

7 (X = NH X' = S X'' = NH)
S0
E = -893.484133228 A.U.
G = -893.358076 A.U.

C	-3.300283	-0.977042	-0.309238
C	-3.400755	0.394939	-0.511280
C	-2.280074	1.197069	-0.322439
C	-1.069913	0.646522	0.088702
C	-0.964080	-0.741902	0.279577
C	-2.088079	-1.541755	0.067164
C	1.598896	0.626795	0.064381
C	1.425582	-0.718471	0.251028
N	0.249476	-1.325401	0.693001
C	2.900220	0.820689	-0.485167
C	3.472125	-0.422047	-0.589990
H	-4.161818	-1.616496	-0.457249
H	-4.338970	0.840475	-0.816456
H	-2.342874	2.267619	-0.480803
H	-2.003679	-2.614073	0.206476
H	0.248482	-2.336282	0.691359
H	3.361354	1.760111	-0.744233
H	4.451286	-0.724094	-0.920617
S	0.292537	1.741180	0.481436
N	2.556799	-1.358590	-0.153421
H	2.699869	-2.357002	-0.154936

T1
E = -893.381413952 A.U.
G = -893.260048 A.U.

C	-3.392086	0.947182	-0.195693
C	-3.480942	-0.453210	0.104768
C	-2.373300	-1.206666	0.342952
C	-1.044835	-0.622622	0.333954
C	-0.983598	0.805228	0.013606
C	-2.129090	1.544888	-0.222235
C	1.593365	-0.635037	-0.061075
C	1.425651	0.753975	0.078745
N	0.260153	1.430858	0.053247
C	2.995938	-0.883924	-0.085045
C	3.618250	0.326821	0.049981
H	-4.282461	1.530420	-0.383104
H	-4.458772	-0.920268	0.161661

H	-2.469661	-2.262439	0.577077
H	-2.032251	2.613661	-0.393257
H	0.285290	2.440585	-0.026727
H	3.476879	-1.843866	-0.174894
H	4.665708	0.572934	0.098412
S	0.256694	-1.696968	-0.230135
N	2.664588	1.320128	0.147585
H	2.858849	2.303754	0.257423

7 (X = NH X' = S X'' = O)
S0
E = -913.336289704 A.U.
G = -913.222527 A.U.

C	3.297132	-0.974648	0.287235
C	3.398377	0.398000	0.480289
C	2.274339	1.198303	0.304579
C	1.058877	0.644885	-0.085603
C	0.954001	-0.744565	-0.266959
C	2.080153	-1.542522	-0.068730
C	-1.603214	0.627519	-0.059263
C	-1.422482	-0.700016	-0.240887
N	-0.268350	-1.339300	-0.648580
C	-2.927090	0.771425	0.480568
C	-3.433339	-0.481615	0.553390
H	4.161498	-1.612345	0.424788
H	4.340391	0.846696	0.768331
H	2.339158	2.269424	0.457428
H	1.994001	-2.615236	-0.202033
H	-0.294466	-2.349578	-0.606212
H	-3.431687	1.684863	0.749733
H	-4.386074	-0.884038	0.848761
S	-0.304227	1.754557	-0.451880
O	-2.504657	-1.402273	0.127704

T1
E = -913.233121823 A.U.
G = -913.123951 A.U.

C	3.374469	-0.949775	-0.187737
C	3.465063	0.455020	0.113527
C	2.355814	1.209816	0.333341
C	1.031946	0.629191	0.310296
C	0.969957	-0.804700	0.004336
C	2.120044	-1.546580	-0.223592
C	-1.595842	0.637541	-0.055866
C	-1.416410	-0.732847	0.084233
N	-0.273890	-1.426453	0.057960
C	-3.016549	0.831212	-0.078294
C	-3.566193	-0.395879	0.055436
H	4.266684	-1.532094	-0.369314
H	4.442999	0.918936	0.181728
H	2.450276	2.267758	0.557987
H	2.024345	-2.614488	-0.398681
H	-0.329789	-2.437566	0.000506
H	-3.545759	1.765067	-0.171041
H	-4.581616	-0.747407	0.109070
S	-0.272217	1.708586	-0.230436
O	-2.598529	-1.371301	0.154615

____

7 (X = NH X' = S X'' = S)

S0

E = -1236.31616163 A.U.

G = -1236.206312 A.U.

```
C        -3.438743   -1.150142   -0.354969
C        -3.639023    0.204443   -0.596469
C        -2.596933    1.101648   -0.382557
C        -1.370153    0.654491    0.095312
C        -1.161911   -0.712988    0.323241
C        -2.202900   -1.609629    0.085254
C         1.294759    0.824227    0.109103
C         1.226459   -0.517870    0.331556
N         0.085823   -1.168202    0.795217
C         2.542065    1.237592   -0.450558
C         3.396254    0.196084   -0.623732
H        -4.240491   -1.858796   -0.522022
H        -4.596240    0.564264   -0.951535
H        -2.737571    2.159764   -0.569982
H        -2.038069   -2.668408    0.252331
H         0.156364   -2.170870    0.899007
H         2.788010    2.264366   -0.683209
H         4.414620    0.211018   -0.978228
S        -0.098265    1.836941    0.524920
S         2.682851   -1.317632   -0.164293
```

T1

E = -1236.22166140 A.U.

G = -1236.116452 A.U.

```
C         3.515841   -1.173004    0.246171
C         3.776418    0.194893    0.096646
C         2.724721    1.075676   -0.071244
C         1.400687    0.621793   -0.092892
C         1.137913   -0.757161    0.029339
C         2.219416   -1.640104    0.210062
C        -1.273839    0.869521   -0.028555
C        -1.240858   -0.542116   -0.471157
N        -0.140674   -1.273812   -0.058639
C        -2.483706    1.254785    0.522794
C        -3.439181    0.252660    0.513642
H         4.331383   -1.871614    0.384619
H         4.792658    0.566336    0.121186
H         2.915335    2.138480   -0.172239
H         2.012650   -2.700141    0.309348
H        -0.224489   -2.282271   -0.023049
H        -2.673306    2.238905    0.931457
H        -4.424722    0.286073    0.951719
S         0.134217    1.844920   -0.248544
S        -2.869796   -1.244966   -0.240294
```

____

7 (X = O X' = NH X'' = NH)

S0

E = -570.501172683 A.U.

G = -570.371753 A.U.

```
C        -3.293765   -0.762476    0.095159
C        -3.339791    0.626426    0.099970
```

C	-2.163951	1.362551	0.012853
C	-0.918658	0.739166	-0.074382
C	-0.890546	-0.668838	-0.067475
C	-2.061750	-1.403675	0.013949
C	1.440991	0.749949	-0.044180
C	1.393933	-0.611313	-0.038217
C	2.813089	1.114040	0.057982
C	3.533068	-0.057809	0.124653
H	-4.202503	-1.347036	0.158317
H	-4.288904	1.143419	0.167360
H	-2.194476	2.446655	0.005840
H	-1.983764	-2.484254	0.017183
H	3.218486	2.112943	0.083677
H	4.591719	-0.229473	0.214723
O	0.288696	-1.402976	-0.148127
N	0.255754	1.510730	-0.198176
H	0.229328	2.360605	0.351228
N	2.652121	-1.112860	0.058594
H	2.889699	-2.092259	0.081881

T1
E = -570.391595006 A.U.
G = -570.267445 A.U.

C	3.285205	-0.775084	-0.210757
C	3.324859	0.653782	-0.080068
C	2.204564	1.383064	0.165982
C	0.912559	0.733040	0.344586
C	0.899265	-0.706381	0.085682
C	2.033925	-1.431102	-0.133333
C	-1.427257	0.767210	-0.064230
C	-1.391566	-0.619537	0.107110
C	-2.802220	1.130204	-0.203734
C	-3.519540	-0.031461	-0.111555
H	4.186552	-1.337540	-0.408291
H	4.278722	1.163220	-0.166658
H	2.257516	2.461307	0.278216
H	1.951875	-2.508202	-0.227493
H	-3.201671	2.121370	-0.339400
H	-4.583008	-0.195368	-0.151996
O	-0.309373	-1.387179	0.155772
N	-0.254318	1.440194	-0.022335
H	-0.223862	2.443798	-0.147493
N	-2.658502	-1.098007	0.074662
H	-2.920166	-2.068873	0.152545

---

7 (X = O X' = NH X'' = O)
S0
E = -590.352936138 A.U.
G = -590.235752 A.U.

C	-3.280839	-0.762636	0.062225
C	-3.329614	0.625936	0.062678
C	-2.153866	1.364616	-0.001073
C	-0.907369	0.742744	-0.055508
C	-0.877940	-0.664325	-0.044909
C	-2.047067	-1.402878	0.009068
C	1.442303	0.748205	-0.019003
C	1.390206	-0.596591	-0.011704

C	2.838991	1.071703	0.034020
C	3.491155	-0.116969	0.073102
H	-4.189760	-1.348471	0.106308
H	-4.281040	1.140872	0.107179
H	-2.185512	2.448439	-0.013368
H	-1.967024	-2.483096	0.015597
H	3.288213	2.051362	0.048077
H	4.529007	-0.392560	0.129152
O	0.308566	-1.401763	-0.090951
N	0.269985	1.523827	-0.149449
H	0.245148	2.347410	0.439426
O	2.600849	-1.159433	0.036000

T1
E = -590.241470091 A.U.
G = -590.129724 A.U.

C	3.266973	-0.780004	-0.200212
C	3.313983	0.654386	-0.058501
C	2.193743	1.386071	0.167370
C	0.899700	0.743660	0.309435
C	0.882794	-0.704620	0.066007
C	2.021852	-1.431294	-0.136799
C	-1.430482	0.765663	-0.068585
C	-1.388490	-0.602640	0.118077
C	-2.823830	1.086285	-0.203241
C	-3.477487	-0.091919	-0.095333
H	4.170036	-1.344276	-0.383556
H	4.272354	1.157459	-0.121376
H	2.248117	2.463937	0.281346
H	1.940372	-2.508451	-0.230164
H	-3.267551	2.056521	-0.351606
H	-4.520232	-0.355324	-0.118847
O	-0.326810	-1.382142	0.150545
N	-0.265411	1.449625	-0.037383
H	-0.241589	2.453187	-0.167780
O	-2.610210	-1.145854	0.094998

---

7 (X = O X' = NH X'' = S)
S0
E = -913.332510957 A.U.
G = -913.219909 A.U.

C	3.497209	-0.876351	-0.214825
C	3.621847	0.506653	-0.230561
C	2.500606	1.313660	-0.059781
C	1.240158	0.755173	0.136452
C	1.130750	-0.644958	0.146790
C	2.240709	-1.449570	-0.031060
C	-1.119885	0.890922	0.130249
C	-1.162256	-0.464607	0.157159
C	-2.407212	1.459200	-0.110201
C	-3.368742	0.507877	-0.257610
H	4.364169	-1.510676	-0.346624
H	4.590961	0.967146	-0.376383
H	2.594296	2.393808	-0.070381
H	2.102423	-2.523828	-0.017852
H	-2.597700	2.522366	-0.162868
H	-4.423593	0.645235	-0.432786

O	-0.086394	-1.283638	0.367714
N	0.102129	1.558701	0.344227
H	0.162777	2.479643	-0.070104
S	-2.741012	-1.100218	-0.117124

T1
E = -913.243842631 A.U.
G = -913.134609 A.U.

C	-3.502040	-0.900200	0.154926
C	-3.648551	0.478208	-0.028763
C	-2.536858	1.291643	-0.155524
C	-1.249436	0.742741	-0.098017
C	-1.114541	-0.646369	0.076762
C	-2.234339	-1.458778	0.199627
C	1.121464	0.959514	-0.083011
C	1.149280	-0.490988	-0.399155
C	2.347437	1.476238	0.292713
C	3.360865	0.528489	0.301715
H	-4.373383	-1.534320	0.253612
H	-4.636901	0.918149	-0.070181
H	-2.643303	2.361726	-0.291536
H	-2.083210	-2.523412	0.328480
H	2.506337	2.509265	0.572839
H	4.362734	0.644487	0.683459
O	0.120968	-1.243552	0.113198
N	-0.113423	1.536054	-0.146158
H	-0.215077	2.534445	-0.019040
S	2.796833	-1.099831	-0.181734

---

7 (X = O X' = O X'' = NH)
S0
E = -590.359013784 A.U.
G = -590.242058 A.U.

C	-3.288145	-0.718028	0.000283
C	-3.304909	0.672091	0.000258
C	-2.108871	1.380240	0.000044
C	-0.890346	0.714889	-0.000169
C	-0.878613	-0.689538	-0.000161
C	-2.072694	-1.392951	0.000085
C	1.399690	0.730945	-0.000145
C	1.383807	-0.629153	-0.000127
C	2.754206	1.149577	0.000092
C	3.509691	-0.001267	0.000379
H	-4.213042	-1.280115	0.000464
H	-4.244380	1.209805	0.000412
H	-2.091248	2.463350	0.000032
H	-2.023484	-2.475035	0.000099
H	3.122528	2.162240	0.000092
H	4.576848	-0.139630	0.000726
O	0.247188	1.490932	-0.000404
O	0.289102	-1.439502	-0.000419
N	2.660971	-1.085209	0.000163
H	2.932774	-2.056418	0.000386

T1
E = -590.236895149 A.U.
G = -590.125000 A.U.

C	3.256428	-0.710335	-0.098561
C	3.262544	0.698659	-0.304317
C	2.054499	1.401425	-0.186338
C	0.901515	0.726280	0.123330
C	0.877508	-0.685133	0.453297
C	2.121012	-1.390685	0.242106
C	-1.391937	0.729208	0.134924
C	-1.355732	-0.667017	-0.083568
C	-2.741279	1.136066	0.095806
C	-3.470724	-0.005483	-0.145272
H	4.186640	-1.259921	-0.199745
H	4.174010	1.214998	-0.570338
H	2.006027	2.476633	-0.321142
H	2.142830	-2.460748	0.416179
H	-3.127312	2.134130	0.212567
H	-4.534982	-0.132343	-0.252260
O	-0.282191	1.452678	0.232600
O	-0.294679	-1.411881	-0.038567
N	-2.629238	-1.092349	-0.259936
H	-2.910592	-2.050599	-0.406404

7 (X = O X' = O X'' = O)
S0
E = -610.208964915 A.U.
G = -610.104211 A.U.

C	3.271939	-0.717704	0.000207
C	3.290822	0.672101	0.000190
C	2.096426	1.383445	0.000028
C	0.879292	0.718533	-0.000139
C	0.865539	-0.685446	-0.000125
C	2.056050	-1.392137	0.000059
C	-1.400145	0.729157	-0.000123
C	-1.379473	-0.614483	-0.000124
C	-2.777916	1.108729	0.000166
C	-3.465181	-0.060946	0.000294
H	4.196088	-1.280780	0.000342
H	4.231229	1.207941	0.000310
H	2.080452	2.466434	0.000019
H	2.004996	-2.473989	0.000071
H	-3.190083	2.104019	0.000234
H	-4.512423	-0.304165	0.000517
O	-0.262358	1.498531	-0.000319
O	-0.309532	-1.436470	-0.000311
O	-2.607408	-1.132931	0.000118

T1
E = -610.083886563 A.U.
G = -609.984106 A.U.

C	-3.222671	-0.739029	-0.284954
C	-3.254644	0.690217	-0.071444
C	-2.146004	1.395109	0.246747
C	-0.858472	0.721665	0.396824
C	-0.864000	-0.736456	0.111216
C	-2.004593	-1.426672	-0.184764
C	1.367794	0.737334	-0.103555
C	1.378136	-0.607260	0.126817
C	2.735214	1.121367	-0.266929

C	3.440315	-0.025769	-0.123127
H	-4.128846	-1.269835	-0.538251
H	-4.205185	1.204766	-0.156118
H	-2.181769	2.463762	0.421580
H	-1.946388	-2.500270	-0.321723
H	3.130137	2.105457	-0.454702
H	4.491295	-0.252075	-0.149418
O	0.230188	1.449059	-0.081224
O	0.332473	-1.414103	0.229108
O	2.614127	-1.101811	0.116821

---

7 (X = O X' = O X'' = S)
S0
E = -933.189417973 A.U.
G = -933.089668 A.U.

C	3.526165	-0.825051	0.000173
C	3.616605	0.561918	0.000172
C	2.459376	1.333686	0.000092
C	1.212606	0.727428	0.000007
C	1.124745	-0.669878	-0.000010
C	2.277305	-1.437899	0.000084
C	-1.084366	0.862392	-0.000031
C	-1.152065	-0.490532	0.000004
C	-2.364952	1.482426	0.000032
C	-3.364929	0.561169	0.000140
H	4.420859	-1.434042	0.000244
H	4.583478	1.048460	0.000235
H	2.496269	2.416223	0.000097
H	2.170468	-2.515742	0.000066
H	-2.512563	2.552846	0.000039
H	-4.429557	0.729671	0.000484
O	0.105407	1.550787	-0.000061
O	-0.086728	-1.343131	-0.000164
S	-2.773832	-1.068288	-0.000209

T1
E = -933.090981257 A.U.
G = -932.994579 A.U.

C	-3.472227	-0.879357	0.202025
C	-3.609306	0.490696	-0.027804
C	-2.486601	1.287670	-0.188668
C	-1.220662	0.717377	-0.124332
C	-1.083467	-0.657924	0.087637
C	-2.211096	-1.452861	0.251651
C	1.067419	0.942598	-0.105033
C	1.145845	-0.503950	-0.445678
C	2.222062	1.483871	0.378684
C	3.275548	0.559563	0.408741
H	-4.348423	-1.501019	0.332318
H	-4.593137	0.939441	-0.074094
H	-2.560869	2.355392	-0.352792
H	-2.070934	-2.514906	0.409599
H	2.313995	2.502558	0.729035
H	4.224621	0.680330	0.907203
O	-0.133545	1.553454	-0.212432
O	0.151071	-1.269390	0.100343
S	2.820590	-1.041274	-0.229869

---

7 (X = O X' = S X'' = NH)

S0

E = -913.340904162 A.U.

G = -913.227507 A.U.

C	-3.270381	-1.021713	0.263416
C	-3.405397	0.352147	0.433863
C	-2.302435	1.181581	0.272080
C	-1.058716	0.658257	-0.079690
C	-0.937928	-0.725434	-0.244320
C	-2.032127	-1.559649	-0.063810
C	1.589038	0.629933	-0.054800
C	1.379359	-0.706670	-0.224634
C	2.925416	0.782768	0.418924
C	3.463346	-0.478220	0.500147
H	-4.121996	-1.677384	0.393328
H	-4.363856	0.780601	0.697991
H	-2.400287	2.251953	0.411831
H	-1.891058	-2.625491	-0.194236
H	3.427318	1.707439	0.653203
H	4.447633	-0.806431	0.787332
O	0.246180	-1.346605	-0.613113
S	0.294807	1.785215	-0.380953
N	2.502072	-1.386936	0.117751
H	2.600334	-2.390745	0.099411

T1

E = -913.227612206 A.U.

G = -913.118954 A.U.

C	-3.315743	-0.981632	-0.278383
C	-3.454033	0.404658	0.064241
C	-2.379952	1.175691	0.376232
C	-1.028864	0.624939	0.398214
C	-0.942160	-0.790379	0.045155
C	-2.037999	-1.556681	-0.258334
C	1.564096	0.638658	-0.095917
C	1.385222	-0.731089	0.107906
C	2.974701	0.858401	-0.168932
C	3.574227	-0.356260	0.005198
H	-4.181366	-1.575637	-0.534527
H	-4.445480	0.843784	0.091620
H	-2.511551	2.219341	0.643635
H	-1.891065	-2.614874	-0.445944
H	3.470475	1.804700	-0.307499
H	4.616686	-0.623922	0.038036
O	0.274790	-1.447492	0.135008
S	0.234971	1.711462	-0.201960
N	2.599238	-1.322967	0.172302
H	2.752804	-2.313906	0.287574

---

7 (X = O X' = S X'' = O)

S0

E = -933.191531007 A.U.

G = -933.090543 A.U.

C	-3.270771	-1.016482	0.237528
C	-3.406322	0.359070	0.389826

C	-2.298877	1.185156	0.243629
C	-1.048616	0.656799	-0.075247
C	-0.929504	-0.728369	-0.221794
C	-2.026861	-1.559698	-0.057353
C	1.590065	0.622217	-0.048759
C	1.377659	-0.698532	-0.205723
C	2.952011	0.739159	0.395873
C	3.429456	-0.526315	0.449875
H	-4.126075	-1.669383	0.355300
H	-4.369314	0.792459	0.627678
H	-2.398675	2.256899	0.370115
H	-1.883500	-2.626812	-0.172768
H	3.492758	1.642637	0.624572
H	4.387577	-0.948408	0.696217
O	0.263937	-1.365547	-0.551649
S	0.307119	1.793173	-0.343365
O	2.457799	-1.426475	0.094849

T1
E = -933.076926847 A.U.
G = -932.980572 A.U.

C	-3.294616	-0.983718	-0.280011
C	-3.438413	0.403968	0.075666
C	-2.368310	1.176836	0.384673
C	-1.015036	0.631625	0.385209
C	-0.925951	-0.791841	0.040985
C	-2.021566	-1.559147	-0.263943
C	1.563060	0.631911	-0.099676
C	1.382494	-0.714463	0.110930
C	2.988768	0.813303	-0.165655
C	3.525070	-0.410776	0.018515
H	-4.160641	-1.575972	-0.538379
H	-4.432280	0.835732	0.113512
H	-2.500768	2.218164	0.659279
H	-1.875281	-2.616133	-0.457136
H	3.522596	1.737045	-0.314039
H	4.535542	-0.775984	0.068773
O	0.292294	-1.444323	0.145255
S	0.239913	1.716055	-0.213916
O	2.545111	-1.363916	0.186055

---

7 (X = O X' = S X'' = S)
S0
E = -1256.16970055 A.U.
G = -1256.072869 A.U.

C	3.384106	-1.205252	-0.322846
C	3.629194	0.146523	-0.540573
C	2.616137	1.078280	-0.343450
C	1.358237	0.669853	0.093476
C	1.126333	-0.689405	0.305004
C	2.124704	-1.626241	0.088769
C	-1.285216	0.838956	0.097339
C	-1.187524	-0.496331	0.312360
C	-2.563550	1.211823	-0.422037
C	-3.384619	0.139635	-0.573908
H	4.167266	-1.935844	-0.480561
H	4.605276	0.479318	-0.869860

H	2.798396	2.131632	-0.520687
H	1.899407	-2.671687	0.258651
H	-2.849248	2.228572	-0.651861
H	-4.407767	0.124876	-0.914663
O	-0.094349	-1.165891	0.765329
S	-2.621892	-1.351484	-0.132567
S	0.099058	1.886685	0.438289

T1
E = -1256.05276877 A.U.
G = -1255.961059 A.U.

C	-3.493102	-1.134034	-0.267703
C	-3.733329	0.238230	0.113160
C	-2.718151	1.083047	0.409971
C	-1.330475	0.635475	0.369012
C	-1.136441	-0.776037	0.008387
C	-2.183648	-1.615141	-0.288531
C	1.260823	0.820463	-0.101699
C	1.192830	-0.546753	0.075502
C	2.616087	1.280626	-0.173654
C	3.509543	0.272022	-0.047440
H	-4.319966	-1.784387	-0.514472
H	-4.755737	0.592805	0.178064
H	-2.917997	2.108625	0.701576
H	-1.965478	-2.654869	-0.505500
H	2.889553	2.318902	-0.298173
H	4.586225	0.334027	-0.042773
O	0.117741	-1.329388	0.073979
S	2.758768	-1.284778	0.157455
S	-0.156477	1.795566	-0.200741

---

7 (X = S X' = NH X'' = NH)
S0
E = -893.482456218 A.U.
G = -893.356108 A.U.

C	-3.378836	-0.446802	0.540862
C	-3.318098	0.925684	0.323366
C	-2.128526	1.521500	-0.074207
C	-0.979643	0.756068	-0.294934
C	-1.052971	-0.633677	-0.091110
C	-2.238730	-1.218115	0.341706
C	1.422202	0.817318	-0.256044
C	1.581720	-0.534903	-0.112058
C	2.608320	1.443302	0.214696
C	3.450263	0.430316	0.621543
H	-4.299675	-0.915269	0.863754
H	-4.195367	1.542193	0.477257
H	-2.077954	2.594785	-0.222627
H	-2.268159	-2.288953	0.507565
H	2.816748	2.501009	0.248266
H	4.451878	0.468007	1.016123
S	0.327985	-1.698979	-0.512840
N	0.210047	1.367401	-0.717747
H	0.176958	2.377241	-0.698264
N	2.811694	-0.765522	0.443998
H	3.201419	-1.672640	0.646706

T1
E = -893.379363601 A.U.
G = -893.257667 A.U.

C	-3.474142	-0.487765	0.096455
C	-3.411365	0.906942	-0.216011
C	-2.160918	1.530472	-0.229800
C	-1.001056	0.817134	0.028181
C	-1.039750	-0.606458	0.362112
C	-2.354056	-1.218060	0.360301
C	1.428858	0.836121	0.090383
C	1.571776	-0.554084	-0.056773
C	2.739577	1.396745	0.154280
C	3.614345	0.349538	0.046345
H	-4.442810	-0.974870	0.145925
H	-4.311133	1.469953	-0.419876
H	-2.083433	2.600007	-0.406092
H	-2.434469	-2.271781	0.608975
H	2.990593	2.439153	0.263344
H	4.691629	0.340228	0.060872
S	0.287508	-1.667055	-0.238918
N	0.225800	1.466486	0.074035
H	0.227438	2.476030	-0.002603
N	2.911843	-0.821766	-0.087603
H	3.318946	-1.742394	-0.145728

---

7 (X = S X' = NH X'' = O)
S0
E = -913.331609841 A.U.
G = -913.217716 A.U.

C	-3.377595	-0.453951	0.508516
C	-3.321592	0.919037	0.296688
C	-2.128781	1.522141	-0.079634
C	-0.973417	0.763839	-0.283329
C	-1.040827	-0.626603	-0.086042
C	-2.230363	-1.218285	0.325640
C	1.420342	0.824579	-0.236323
C	1.575215	-0.512772	-0.111693
C	2.642055	1.411396	0.233032
C	3.428743	0.366263	0.594349
H	-4.300640	-0.929328	0.814251
H	-4.204504	1.530354	0.437325
H	-2.081072	2.595947	-0.223725
H	-2.257355	-2.289650	0.487738
H	2.895114	2.458053	0.283839
H	4.432490	0.301016	0.977716
S	0.346002	-1.703552	-0.477487
N	0.220273	1.394062	-0.677642
H	0.187369	2.403243	-0.643321
O	2.785998	-0.818136	0.410279

T1
E = -913.227447343 A.U.
G = -913.118350 A.U.

C	-3.458299	-0.493466	0.095445
C	-3.396066	0.907626	-0.214525
C	-2.154656	1.533274	-0.229677
C	-0.988605	0.823662	0.027599

C	-1.026520	-0.607868	0.347012
C	-2.336696	-1.221046	0.347392
C	1.425943	0.839981	0.089687
C	1.559727	-0.531736	-0.063948
C	2.762981	1.362270	0.151730
C	3.575387	0.289556	0.035235
H	-4.426323	-0.980198	0.148557
H	-4.298242	1.467185	-0.417210
H	-2.081327	2.602346	-0.409401
H	-2.412034	-2.277647	0.584973
H	3.059357	2.391947	0.266275
H	4.646859	0.184036	0.032458
S	0.306123	-1.667473	-0.227271
N	0.233018	1.480222	0.092784
H	0.235602	2.491176	0.038088
O	2.870979	-0.871793	-0.096575

---

7 (X = S X' = NH X'' = S)
S0
E = -1236.31693443 A.U.
G = -1236.206966 A.U.

C	3.603303	-0.532093	-0.601139
C	3.590094	0.845906	-0.413478
C	2.433712	1.486855	0.013710
C	1.278723	0.755057	0.294943
C	1.303550	-0.637116	0.123259
C	2.451331	-1.268192	-0.342705
C	-1.125560	0.896696	0.311609
C	-1.334874	-0.444804	0.187273
C	-2.260492	1.669809	-0.084960
C	-3.293811	0.884568	-0.492540
H	4.498592	-1.032973	-0.946406
H	4.478574	1.432065	-0.613852
H	2.417658	2.564127	0.137341
H	2.443688	-2.342670	-0.484275
H	-2.295836	2.750543	-0.056615
H	-4.273072	1.192268	-0.824736
S	-0.102698	-1.622937	0.627671
N	0.112085	1.400231	0.740532
H	0.175211	2.409219	0.735284
S	-2.903881	-0.796707	-0.446814

T1
E = -1236.22557624 A.U.
G = -1236.120125 A.U.

C	3.738581	-0.614335	-0.034467
C	3.722659	0.781305	-0.162555
C	2.527692	1.467577	-0.168761
C	1.303182	0.784666	-0.037052
C	1.322359	-0.618824	0.096835
C	2.545423	-1.300177	0.088411
C	-1.127998	0.987562	-0.000392
C	-1.296886	-0.435644	-0.363102
C	-2.317095	1.627175	0.334778
C	-3.427978	0.816378	0.226029
H	4.675631	-1.155605	-0.039939
H	4.651602	1.328732	-0.262562

H	2.511696	2.546773	-0.272803
H	2.541623	-2.380463	0.179242
H	-2.369829	2.661353	0.650747
H	-4.446002	1.041231	0.500498
S	-0.145236	-1.563129	0.318179
N	0.131897	1.514178	0.003126
H	0.223671	2.515033	0.121724
S	-3.020470	-0.820020	-0.366749

7 (X = S X' = O X'' = NH)

S0

E = -913.338022090 A.U.

G = -913.224515 A.U.

C	3.384237	0.400905	0.476412
C	3.287944	-0.973336	0.283239
C	2.071755	-1.540629	-0.074956
C	0.953455	-0.738331	-0.267427
C	1.044316	0.645943	-0.082574
C	2.262595	1.202439	0.301619
C	-1.377869	-0.808465	-0.232715
C	-1.566535	0.538318	-0.096474
C	-2.558569	-1.474272	0.176832
C	-3.442134	-0.483262	0.544029
H	4.325193	0.850698	0.766453
H	4.154307	-1.607880	0.421540
H	1.963606	-2.608471	-0.220125
H	2.327562	2.273444	0.454666
H	-2.734773	-2.537357	0.191309
H	-4.458435	-0.545638	0.894729
S	-0.330106	1.741264	-0.422457
O	-0.201786	-1.380461	-0.658953
N	-2.827107	0.730993	0.394462
H	-3.246887	1.625847	0.593223

T1

E = -913.223523249 A.U.

G = -913.115010 A.U.

C	-3.448462	-0.443073	0.059204
C	-3.336048	0.941462	-0.292245
C	-2.072518	1.541934	-0.264763
C	-0.957546	0.804364	0.055887
C	-1.024417	-0.609133	0.416966
C	-2.360507	-1.189780	0.386665
C	1.391655	0.821461	0.121346
C	1.540133	-0.556216	-0.084315
C	2.679900	1.401115	0.179243
C	3.569483	0.367978	0.003222
H	-4.430779	-0.902776	0.082987
H	-4.212175	1.515533	-0.558396
H	-1.946333	2.601876	-0.458271
H	-2.473021	-2.233654	0.662097
H	2.912454	2.443437	0.318424
H	4.646729	0.376543	-0.013200
S	0.265198	-1.678813	-0.205232
O	0.234850	1.489103	0.150042
N	2.884024	-0.802667	-0.167131
H	3.302942	-1.714771	-0.267614

---

7 (X = S X' = O X'' = O)
S0
E = -933.185648022 A.U.
G = -933.084729 A.U.

C	3.393306	0.413792	0.411225
C	3.301625	-0.963231	0.239704
C	2.078521	-1.542698	-0.070334
C	0.948707	-0.751526	-0.234591
C	1.033172	0.635736	-0.072699
C	2.260543	1.203964	0.263063
C	-1.377459	-0.818355	-0.196952
C	-1.557567	0.514535	-0.090016
C	-2.608148	-1.438713	0.181427
C	-3.439833	-0.409944	0.479420
H	4.339391	0.875235	0.663358
H	4.176493	-1.590117	0.356468
H	1.972139	-2.612960	-0.196972
H	2.323219	2.277274	0.399603
H	-2.833398	-2.491753	0.213277
H	-4.468353	-0.360787	0.791926
S	-0.346552	1.745351	-0.362914
O	-0.212341	-1.425076	-0.569409
O	-2.807893	0.789593	0.334094

T1
E = -933.069792027 A.U.
G = -932.973830 A.U.

C	-3.431136	-0.443377	0.060182
C	-3.314140	0.944262	-0.301945
C	-2.056534	1.548697	-0.269557
C	-0.941072	0.814974	0.058632
C	-1.009215	-0.610451	0.407136
C	-2.347725	-1.188826	0.390463
C	1.391613	0.820270	0.125143
C	1.525504	-0.531883	-0.093925
C	2.711524	1.360578	0.181105
C	3.533128	0.301477	-0.011837
H	-4.415178	-0.898093	0.087701
H	-4.190301	1.514368	-0.575876
H	-1.933586	2.608226	-0.465965
H	-2.459448	-2.230900	0.671659
H	2.992504	2.388856	0.334623
H	4.604659	0.207288	-0.054555
S	0.272708	-1.678465	-0.202617
O	0.247616	1.501919	0.180478
O	2.835677	-0.855497	-0.183992

---

7 (X = S X' = O X'' = S)
S0
E = -1256.17136496 A.U.
G = -1256.074500 A.U.

C	3.607563	-0.474435	-0.553541
C	3.552643	0.904537	-0.378599
C	2.368349	1.509475	0.025737
C	1.247891	0.731840	0.282147

C	1.295817	-0.653372	0.116447
C	2.477623	-1.248918	-0.316299
C	-1.086075	0.874579	0.298772
C	-1.321744	-0.458421	0.170772
C	-2.195038	1.692197	-0.058969
C	-3.259791	0.938798	-0.443854
H	4.524245	-0.949700	-0.878536
H	4.427144	1.514519	-0.566648
H	2.292377	2.581606	0.157919
H	2.509245	-2.322997	-0.456096
H	-2.184784	2.771754	-0.013199
H	-4.239834	1.272008	-0.747222
S	-0.106335	-1.675787	0.537688
O	0.114291	1.391526	0.718124
S	-2.916549	-0.755280	-0.418744

T1
E = -1256.05423042 A.U.
G = -1255.962516 A.U.

C	-3.700618	-0.533711	0.087704
C	-3.631452	0.852402	-0.306338
C	-2.395190	1.501207	-0.301903
C	-1.252678	0.813970	0.032726
C	-1.272774	-0.609854	0.404102
C	-2.592307	-1.235677	0.421877
C	1.100055	0.903421	0.111611
C	1.298911	-0.450538	-0.084743
C	2.315060	1.641081	0.219431
C	3.399699	0.836691	0.099426
H	-4.669805	-1.017501	0.134921
H	-4.529763	1.386425	-0.581113
H	-2.311263	2.558849	-0.526022
H	-2.664223	-2.274003	0.727430
H	2.347957	2.710412	0.369799
H	4.440958	1.116904	0.136402
S	0.037621	-1.609757	-0.190465
O	-0.088205	1.527248	0.122627
S	2.992351	-0.828557	-0.143646

---

7 (X = S X' = S X'' = NH)
S0
E = -1236.31988127 A.U.
G = -1236.209519 A.U.

C	3.354148	-0.728810	0.672707
C	3.375974	0.661593	0.670179
C	2.256484	1.374358	0.256355
C	1.118175	0.704195	-0.189663
C	1.100312	-0.695750	-0.189404
C	2.212725	-1.405534	0.258111
C	-1.583702	0.737220	-0.121057
C	-1.577789	-0.636359	-0.156497
C	-2.730805	1.144789	0.614687
C	-3.388390	-0.006197	0.981470
H	4.217441	-1.289563	1.008508
H	4.257215	1.195070	1.004046
H	2.260984	2.457733	0.273536
H	2.182213	-2.488466	0.273569

H	-3.035874	2.155051	0.834514
H	-4.310626	-0.146643	1.520525
S	-0.256218	1.674307	-0.801510
S	-0.294313	-1.621771	-0.830486
N	-2.668286	-1.081528	0.533288
H	-2.907640	-2.050098	0.682890

T1
E = -1236.20482994 A.U.
G = -1236.101220 A.U.

C	3.523890	-0.695883	0.251765
C	3.533718	0.648375	-0.303003
C	2.358543	1.327365	-0.467552
C	1.118052	0.717318	-0.162139
C	1.098468	-0.689447	0.249161
C	2.346926	-1.339765	0.484372
C	-1.589577	0.728336	0.102283
C	-1.571509	-0.656672	-0.085844
C	-2.956859	1.128317	0.167584
C	-3.708579	-0.001280	-0.010901
H	4.463170	-1.186036	0.476683
H	4.474698	1.111645	-0.569967
H	2.364038	2.357725	-0.813141
H	2.342693	-2.364927	0.844869
H	-3.325572	2.131923	0.303652
H	-4.777504	-0.132615	-0.040926
S	-0.217947	1.769257	0.216858
S	-0.232525	-1.729862	-0.221027
N	-2.869575	-1.073546	-0.173586
H	-3.165363	-2.033193	-0.273723

---

7 (X = S X' = S X'' = O)
S0
E = -1256.16716484 A.U.
G = -1256.069543 A.U.

C	-3.354770	-0.728786	0.646859
C	-3.378734	0.661319	0.640634
C	-2.254681	1.374711	0.241126
C	-1.110393	0.704741	-0.189174
C	-1.090187	-0.694333	-0.184921
C	-2.206736	-1.404821	0.250321
C	1.581728	0.737110	-0.109334
C	1.572977	-0.617544	-0.151751
C	2.755130	1.106353	0.630020
C	3.359735	-0.062678	0.952066
H	-4.221406	-1.290474	0.971890
H	-4.264911	1.194660	0.960937
H	-2.260574	2.458005	0.257228
H	-2.174467	-2.487515	0.270335
H	3.096856	2.099957	0.868849
H	4.273176	-0.302062	1.469193
S	0.265431	1.690988	-0.776748
S	0.310135	-1.635256	-0.797360
O	2.637232	-1.127589	0.504028

T1
E = -1256.05287387 A.U.

G = -1255.961609 A.U.

C	3.507707	-0.695056	-0.258467
C	3.517919	0.651592	0.294658
C	2.348803	1.329086	0.475478
C	1.099418	0.716253	0.184853
C	1.087159	-0.696954	-0.216840
C	2.328882	-1.344208	-0.470592
C	-1.584323	0.731441	-0.105228
C	-1.559921	-0.631330	0.086847
C	-2.975065	1.092286	-0.162971
C	-3.661293	-0.054639	0.026674
H	4.445885	-1.182369	-0.491738
H	4.461699	1.115873	0.550400
H	2.357778	2.357326	0.826039
H	2.321793	-2.371919	-0.822685
H	-3.385274	2.078487	-0.305310
H	-4.711946	-0.282611	0.081578
S	-0.219531	1.774413	-0.235110
S	-0.255277	-1.735476	0.207701
O	-2.818589	-1.116076	0.184225

---

7 (X = S X' = S X'' = S)

S0

E = -1579.15080463 A.U.

G = -1579.057337 A.U.

C	-3.511315	-0.889393	-0.730975
C	-3.610132	0.497014	-0.784796
C	-2.553552	1.289107	-0.350184
C	-1.405618	0.698275	0.174770
C	-1.309130	-0.695245	0.230250
C	-2.355512	-1.485765	-0.240064
C	1.295344	0.879287	0.184691
C	1.366153	-0.481853	0.260307
C	2.423760	1.461286	-0.469198
C	3.325652	0.519865	-0.856959
H	-4.326112	-1.509629	-1.082768
H	-4.502933	0.965805	-1.179253
H	-2.616338	2.368910	-0.411363
H	-2.262785	-2.564734	-0.212731
H	2.550329	2.523383	-0.625069
H	4.277546	0.668584	-1.343140
S	-0.109594	1.755138	0.805651
S	2.800918	-1.083865	-0.492480
S	0.114076	-1.496760	0.958533

T1

E = -1579.04813924 A.U.

G = -1578.959796 A.U.

C	3.740877	-0.929462	-0.093485
C	3.868685	0.446718	0.115135
C	2.740424	1.237665	0.200396
C	1.456665	0.684393	0.068463
C	1.328374	-0.698814	-0.114741
C	2.484140	-1.491191	-0.198858
C	-1.281256	0.921843	-0.056012
C	-1.339878	-0.472792	0.431806
C	-2.494971	1.376488	-0.571649

C	-3.526534	0.481180	-0.408559
H	4.618847	-1.559151	-0.158710
H	4.848046	0.898508	0.208601
H	2.836979	2.306534	0.356165
H	2.375096	-2.561145	-0.335776
H	-2.625040	2.344629	-1.037805
H	-4.542920	0.576966	-0.758141
S	0.144140	1.867362	0.137390
S	-3.037608	-0.987597	0.463338
S	-0.192167	-1.588672	-0.257560

8 (X = NH X' = NH)

S0

E = -419.058201957 A.U.

G = -418.942744 A.U.

C	-1.690568	-1.273534	-0.305944
C	-2.334106	-0.108508	-0.454437
C	-1.940347	1.192128	0.075819
C	-0.669592	1.604488	0.285049
C	0.507701	0.782183	0.116406
C	0.502278	-0.601365	0.207964
N	-0.553069	-1.468024	0.563932
C	1.855064	1.164327	-0.181490
C	2.595835	0.013985	-0.277106
N	1.762933	-1.051579	-0.038706
H	-2.025431	-2.159467	-0.835955
H	-3.227973	-0.119111	-1.072733
H	-2.733562	1.924610	0.182487
H	-0.507592	2.645968	0.547980
H	-0.825369	-1.304494	1.530768
H	2.221091	2.168394	-0.331621
H	3.639769	-0.138007	-0.495745
H	2.032430	-2.022898	0.000673

T1

E = -419.009836787 A.U.

G = -418.898407 A.U.

C	1.863652	-1.274780	0.000306
C	2.495474	-0.014781	-0.000240
C	1.969648	1.262365	-0.000182
C	0.593240	1.656972	0.000229
C	-0.511537	0.792440	0.000219
C	-0.516504	-0.628395	0.000068
N	0.510384	-1.522793	-0.000239
C	-1.907322	1.167736	0.000117
C	-2.661633	0.035266	-0.000136
N	-1.821103	-1.058589	-0.000365
H	2.460114	-2.174154	0.000350
H	3.578075	-0.087505	-0.000532
H	2.692207	2.071425	-0.000493
H	0.377628	2.719288	0.000413
H	0.247687	-2.498993	0.001862
H	-2.279086	2.180505	0.000200
H	-3.730555	-0.101104	-0.000154
H	-2.121142	-2.020729	0.000290

8 (X = NH X' = O)
S0
E = -438.909967081 A.U.
G = -438.807470 A.U.

C	-1.684108	-1.268279	-0.298825
C	-2.316614	-0.098977	-0.453389
C	-1.918978	1.203195	0.072911
C	-0.652247	1.614171	0.291117
C	0.515897	0.775433	0.117839
C	0.500047	-0.587346	0.216682
N	-0.532217	-1.467180	0.549582
C	1.877666	1.124919	-0.196582
C	2.553417	-0.044260	-0.277776
H	-2.035281	-2.155698	-0.815155
H	-3.212441	-0.107761	-1.068131
H	-2.712894	1.934433	0.181621
H	-0.481916	2.650265	0.565346
H	-0.779291	-1.398902	1.533572
H	2.281210	2.112541	-0.353829
H	3.579170	-0.290543	-0.493790
O	1.729561	-1.098401	-0.028572

T1
E = -438.867307270 A.U.
G = -438.767544 A.U.

C	-1.841534	-1.275670	0.000033
C	-2.470653	-0.016831	-0.000292
C	-1.948454	1.265569	-0.000221
C	-0.576558	1.670110	0.000147
C	0.517744	0.798740	0.000079
C	0.510428	-0.605763	-0.000056
N	-0.488739	-1.516356	0.000370
C	1.931960	1.126321	0.000048
C	2.618423	-0.029660	-0.000015
H	-2.435892	-2.176060	0.000252
H	-3.552915	-0.089287	-0.000626
H	-2.675877	2.069738	-0.000476
H	-0.362304	2.731858	0.000370
H	-0.190523	-2.483608	0.000651
H	2.351894	2.119722	0.000049
H	3.668340	-0.267856	-0.000097
O	1.771288	-1.110864	-0.000131

---

8 (X = NH X' = S)
S0
E = -761.885502771 A.U.
G = -761.786879 A.U.

C	-1.762441	-1.377410	-0.324490
C	-2.538779	-0.305914	-0.525016
C	-2.323502	1.017796	0.044942
C	-1.119260	1.567133	0.315715
C	0.160304	0.898713	0.149062
C	0.308793	-0.464367	0.256067
C	1.404989	1.545235	-0.156345
C	2.434601	0.671118	-0.300134
H	-1.926977	-2.303030	-0.865623
H	-3.386757	-0.414519	-1.194916

H	-3.198744	1.652156	0.137740
H	-1.085504	2.611206	0.611917
H	1.507897	2.614927	-0.283214
H	3.462076	0.886970	-0.549372
S	1.942035	-0.964615	-0.038976
N	-0.684638	-1.396701	0.635766
H	-1.040323	-1.150790	1.557904

T1
E = -761.842416154 A.U.
G = -761.746600 A.U.

C	-1.988375	-1.381550	0.000019
C	-2.757816	-0.209672	-0.000080
C	-2.364647	1.118189	0.000037
C	-1.039090	1.638419	0.000190
C	0.156618	0.910624	0.000087
C	0.323930	-0.497909	-0.000073
C	1.455402	1.548680	0.000081
C	2.502678	0.702524	-0.000276
H	-2.480970	-2.342458	0.000072
H	-3.825588	-0.398878	-0.000217
H	-3.161901	1.852735	-0.000004
H	-0.934815	2.717734	0.000328
H	1.563920	2.625743	0.000141
H	3.555873	0.936469	-0.000537
S	2.011488	-0.973304	-0.000075
N	-0.623109	-1.480770	0.000186
H	-0.270760	-2.428933	0.000201

---

8 (X = O X' = NH)
S0
E = -438.920226664 A.U.
G = -438.817716 A.U.

C	1.656686	-1.293887	0.230452
C	2.322480	-0.153254	0.402854
C	1.956618	1.186638	-0.064010
C	0.699218	1.624297	-0.256557
C	-0.484241	0.796875	-0.106770
C	-0.491559	-0.578048	-0.206104
C	-1.838009	1.170015	0.182903
C	-2.583607	0.023637	0.253269
H	1.981990	-2.238072	0.649319
H	3.236530	-0.235788	0.982091
H	2.773113	1.891951	-0.171072
H	0.543087	2.668500	-0.509437
H	-2.202707	2.174146	0.334469
H	-3.631782	-0.132740	0.443635
O	0.510839	-1.432400	-0.554630
N	-1.743266	-1.044320	0.017703
H	-2.009604	-2.016192	-0.032106

T1
E = -438.856781825 A.U.
G = -438.758368 A.U.

C	-1.793815	-1.297919	0.000414
C	-2.454203	-0.054192	-0.000204
C	-1.979128	1.234355	-0.000413

C	-0.604021	1.664869	0.000001
C	0.494381	0.800346	0.000206
C	0.495940	-0.614652	-0.000104
C	1.896762	1.159440	0.000305
C	2.637271	0.017986	0.000128
H	-2.337621	-2.228858	0.000686
H	-3.532623	-0.175113	-0.000410
H	-2.726291	2.019803	-0.000924
H	-0.403423	2.728886	0.000161
H	2.278206	2.168261	0.000174
H	3.704524	-0.129865	0.000012
O	-0.458832	-1.544807	0.000093
N	1.783272	-1.064069	-0.000608
H	2.045856	-2.037564	0.001818

8 (X = O X' = S)

S0

E = -781.746636562 A.U.

G = -781.660824 A.U.

C	1.717298	-1.394624	0.259814
C	2.514449	-0.353965	0.488516
C	2.339019	0.999389	-0.038647
C	1.154577	1.577489	-0.302601
C	-0.134946	0.913846	-0.144403
C	-0.300321	-0.437998	-0.266816
C	-1.381886	1.553302	0.168272
C	-2.415456	0.682710	0.292964
H	1.853925	-2.373058	0.703302
H	3.361802	-0.530384	1.142245
H	3.238124	1.597984	-0.134857
H	1.133901	2.620436	-0.602003
H	-1.483574	2.622392	0.299516
H	-3.449181	0.895070	0.516072
S	-1.924875	-0.956955	0.027683
O	0.648326	-1.345257	-0.638723

T1

E = -781.684596981 A.U.

G = -781.602517 A.U.

C	-1.912923	-1.399969	0.000331
C	-2.711182	-0.250632	-0.000049
C	-2.373695	1.083879	-0.000370
C	-1.050659	1.641754	-0.000084
C	0.139690	0.919309	0.000042
C	0.310546	-0.482762	-0.000115
C	1.448135	1.547351	0.000162
C	2.483601	0.687901	0.000112
H	-2.346187	-2.387938	0.000612
H	-3.769194	-0.489901	-0.000090
H	-3.193659	1.791731	-0.000801
H	-0.963760	2.721693	0.000049
H	1.565554	2.623049	0.000226
H	3.538159	0.916566	0.000093
S	1.980786	-0.982291	-0.000029
O	-0.565571	-1.492440	0.000026

8 (X = O X' = O)

S0

E = -458.772117935 A.U.

G = -458.681969 A.U.

C	1.628904	-1.293889	0.237162
C	2.296178	-0.156775	0.416191
C	1.942370	1.182051	-0.065796
C	0.690818	1.628960	-0.264137
C	-0.488880	0.794675	-0.101883
C	-0.491038	-0.559923	-0.220502
C	-1.857054	1.131519	0.208967
C	-2.538501	-0.034244	0.257454
H	1.932002	-2.241837	0.662245
H	3.200357	-0.238929	1.010196
H	2.765418	1.877789	-0.183017
H	0.535187	2.667556	-0.535286
H	-2.260537	2.116954	0.378862
H	-3.566322	-0.291238	0.444592
O	-1.701473	-1.087947	-0.004117
O	0.488613	-1.417620	-0.568674

T1

E = -458.711064461 A.U.

G = -458.624639 A.U.

C	1.775511	1.292998	0.000208
C	2.433405	0.052674	0.000094
C	1.960740	-1.239368	-0.000075
C	0.586606	-1.669007	-0.000175
C	-0.499173	-0.796558	-0.000113
C	-0.495102	0.603943	0.000054
C	-1.916399	-1.120954	-0.000170
C	-2.595108	0.038036	-0.000042
H	2.316472	2.225124	0.000351
H	3.511495	0.173949	0.000158
H	2.708484	-2.023442	-0.000125
H	0.379952	-2.731187	-0.000299
H	-2.337195	-2.113589	-0.000269
H	-3.643752	0.281506	-0.000015
O	0.438769	1.538193	0.000187
O	-1.743561	1.113939	0.000002

---

8 (X = S X' = NH)

S0

E = -761.898951905 A.U.

G = -761.799986 A.U.

C	1.944348	-0.801029	0.606782
C	2.255085	0.498810	0.625887
C	1.671462	1.596415	-0.142825
C	0.373407	1.769793	-0.465346
C	-0.715950	0.849808	-0.193615
C	-0.613453	-0.530433	-0.137783
C	-2.073818	1.165889	0.123393
C	-2.726382	-0.015575	0.370375
H	2.417540	-1.502225	1.283574
H	3.035603	0.792840	1.324939
H	2.347652	2.419525	-0.351558
H	0.093326	2.719398	-0.913116

H	-2.503015	2.154011	0.184897
H	-3.749784	-0.216393	0.641049
N	-1.827993	-1.038804	0.215361
H	-2.037386	-2.022355	0.300127
S	0.781488	-1.517203	-0.543665

T1
E = -761.831495213 A.U.
G = -761.738867 A.U.

C	2.157745	-0.831822	0.054825
C	2.492594	0.542560	0.036286
C	1.728683	1.682028	-0.007044
C	0.306386	1.862503	-0.031491
C	-0.707005	0.897427	-0.009915
C	-0.626883	-0.520510	0.016005
C	-2.121979	1.193472	-0.003510
C	-2.808049	0.019320	0.021332
H	2.956774	-1.561131	0.078079
H	3.566337	0.703593	0.064024
H	2.294810	2.607650	-0.017505
H	-0.037161	2.890527	-0.058461
H	-2.547679	2.184580	-0.013268
H	-3.867379	-0.178997	0.038186
N	-1.908161	-1.018382	0.030797
H	-2.153224	-1.996141	0.062725
S	0.663481	-1.661955	-0.051768

---

8 (X = S X' = O)
S0
E = -781.747689601 A.U.
G = -781.661380 A.U.

C	1.919368	-0.795218	0.614337
C	2.230578	0.503641	0.629346
C	1.657842	1.596773	-0.155099
C	0.363757	1.774300	-0.482082
C	-0.715534	0.847267	-0.187271
C	-0.611096	-0.513274	-0.147896
C	-2.085997	1.130904	0.152795
C	-2.675683	-0.065507	0.377245
H	2.383682	-1.493536	1.299841
H	3.004033	0.801428	1.334097
H	2.342101	2.409011	-0.378791
H	0.080039	2.710458	-0.952771
H	-2.549707	2.101337	0.230040
H	-3.673628	-0.359823	0.654375
O	-1.786569	-1.081486	0.200818
S	0.762914	-1.524394	-0.537599

T1
E = -781.684353191 A.U.
G = -781.602682 A.U.

C	2.134529	-0.829288	-0.000008
C	2.468222	0.540215	0.000216
C	1.711004	1.689600	0.000151
C	0.290818	1.875244	-0.000248
C	-0.706150	0.901224	-0.000116
C	-0.616387	-0.501079	0.000111

C	-2.138269	1.153769	-0.000123
C	-2.757765	-0.037287	0.000062
H	2.936425	-1.555693	-0.000111
H	3.542742	0.697383	0.000467
H	2.282983	2.611002	0.000386
H	-0.057126	2.901485	-0.000570
H	-2.606356	2.125206	-0.000211
H	-3.794425	-0.329948	0.000133
O	-1.858484	-1.064963	0.000188
S	0.640477	-1.667758	-0.000117

8 (X = S X' = S)
S0
E = -1104.72745410 A.U.
G = -1104.645404 A.U.

C	2.044844	-0.968563	0.631483
C	2.501084	0.284146	0.711412
C	2.091082	1.442042	-0.080071
C	0.843585	1.758804	-0.474666
C	-0.368968	0.984309	-0.230304
C	-0.434617	-0.388517	-0.202619
C	-1.644612	1.575875	0.053026
C	-2.614547	0.658120	0.304748
H	2.388577	-1.741706	1.307928
H	3.264575	0.478645	1.461266
H	2.868412	2.178643	-0.257903
H	0.694736	2.726375	-0.945217
H	-1.811794	2.644645	0.075850
H	-3.653372	0.830334	0.541082
S	0.874910	-1.493305	-0.608802
S	-2.016050	-0.956334	0.204986

T1
E = -1104.66287191 A.U.
G = -1104.585718 A.U.

C	-2.311664	-0.997116	0.000342
C	-2.802974	0.317867	0.000250
C	-2.162991	1.537107	-0.000027
C	-0.770800	1.858924	-0.000160
C	0.352938	1.031192	-0.000081
C	0.442329	-0.384560	0.000059
C	1.688281	1.600868	-0.000076
C	2.684098	0.697264	0.000038
H	-3.018023	-1.817838	0.000497
H	-3.888041	0.359864	0.000436
H	-2.819889	2.399770	-0.000100
H	-0.546025	2.920020	-0.000290
H	1.852627	2.670581	-0.000154
H	3.748755	0.874843	0.000077
S	-0.730649	-1.643481	-0.000347
S	2.102855	-0.942550	0.000188

9 (X = NH X' = NH)
S0
E = -419.061302710 A.U.
G = -418.946101 A.U.

C	1.829261	-1.223278	0.186311
C	2.385693	-0.016859	0.375011
C	1.874072	1.292673	-0.051414
C	0.590813	1.622295	-0.262273
C	-0.512421	0.685524	-0.119428
C	-0.512813	-0.695338	-0.175940
N	0.620350	-1.501460	-0.468780
N	-1.812067	1.069398	0.127562
C	-2.615812	-0.020823	0.233040
C	-1.834481	-1.146654	0.054981
H	2.367645	-2.105395	0.518818
H	3.352629	-0.014192	0.864825
H	2.621790	2.069821	-0.174478
H	0.342597	2.642028	-0.541185
H	0.392179	-2.484898	-0.453705
H	-2.112056	2.024000	0.251933
H	-3.672419	0.078043	0.418528
H	-2.176227	-2.170216	0.062053

T1
E = -419.016572350 A.U.
G = -418.904469 A.U.

C	-1.908309	-1.240375	0.000064
C	-2.494625	0.034286	0.000030
C	-1.924789	1.295771	-0.000007
C	-0.531898	1.630982	0.000006
C	0.512584	0.714920	-0.000022
C	0.520112	-0.715657	-0.000009
N	-0.569496	-1.547477	-0.000030
N	1.854752	1.075931	-0.000090
C	2.659820	-0.030595	0.000023
C	1.871726	-1.153421	0.000003
H	-2.543742	-2.113667	0.000075
H	-3.579269	0.000550	0.000028
H	-2.612255	2.133712	-0.000046
H	-0.265417	2.682131	0.000021
H	-0.350618	-2.533790	0.000254
H	2.179425	2.029258	-0.000020
H	3.733352	0.063609	0.000019
H	2.214011	-2.176444	-0.000010

---

9 (X = NH X' = O)
S0
E = -438.912471671 A.U.
G = -438.809768 A.U.

C	-1.806795	1.233109	0.186917
C	-2.364478	0.028585	0.372322
C	-1.861291	-1.285404	-0.057983
C	-0.579297	-1.622811	-0.257160
C	0.505640	-0.670718	-0.115670
C	0.518537	0.690695	-0.182336
N	-0.594762	1.510287	-0.473518
C	2.572384	-0.043687	0.236902
C	1.865845	1.103127	0.059014
H	-2.335049	2.116141	0.531318
H	-3.326251	0.026972	0.871867
H	-2.614228	-2.055887	-0.187620

H	-0.312726	-2.637846	-0.530641
H	-0.359372	2.491855	-0.458346
H	3.613884	-0.240855	0.427082
H	2.250613	2.110436	0.073764
O	1.767899	-1.122523	0.141896

T1
E = -438.872417428 A.U.
G = -438.772435 A.U.

C	-1.890620	1.241899	0.000054
C	-2.473431	-0.030523	0.000055
C	-1.903775	-1.295340	0.000007
C	-0.511298	-1.626637	-0.000030
C	0.508534	-0.701201	-0.000010
C	0.524800	0.717639	-0.000009
N	-0.553890	1.556848	-0.000013
C	2.619403	-0.027725	-0.000000
C	1.892208	1.115898	-0.000051
H	-2.529340	2.112730	0.000069
H	-3.557644	0.002613	0.000092
H	-2.590213	-2.133263	-0.000002
H	-0.221801	-2.670350	-0.000059
H	-0.335286	2.543434	0.000101
H	3.680266	-0.212951	0.000018
H	2.276141	2.123642	-0.000077
O	1.820023	-1.128480	-0.000019

---

9 (X = NH X' = S)
S0
E = -761.885966698 A.U.
G = -761.787097 A.U.

C	2.066509	1.129998	-0.357122
C	2.552832	-0.103072	-0.551239
C	2.028209	-1.316419	0.057550
C	0.728509	-1.538127	0.362544
C	-0.317256	-0.554018	0.196574
C	-0.170353	0.815641	0.260771
C	-2.423218	0.689585	-0.245408
C	-1.376673	1.525577	-0.005986
H	2.434447	1.975875	-0.928516
H	3.384190	-0.222589	-1.239502
H	2.714388	-2.150818	0.156307
H	0.434761	-2.531297	0.686249
H	-3.449012	0.945260	-0.461670
H	-1.445872	2.604434	0.007905
N	1.066258	1.429995	0.636916
H	1.364952	1.025668	1.523230
S	-1.964563	-0.971968	-0.156783

T1
E = -761.850021643 A.U.
G = -761.754265 A.U.

C	2.303999	1.091738	0.000030
C	2.758938	-0.228884	0.000220
C	2.048387	-1.419223	0.000176
C	0.630562	-1.585433	-0.000086
C	-0.318816	-0.578570	-0.000057

C	-0.175517	0.841555	0.000017
C	-2.496904	0.701662	-0.000060
C	-1.419886	1.529115	0.000031
H	3.025363	1.895978	-0.000077
H	3.840234	-0.311119	0.000393
H	2.631172	-2.332528	0.000347
H	0.262272	-2.605621	-0.000271
H	-3.540128	0.975536	-0.000083
H	-1.497395	2.609198	0.000093
N	1.010515	1.535455	-0.000092
H	0.905428	2.540987	-0.000022
S	-2.042820	-0.977023	-0.000085

9 (X = O X' = NH)
S0
E = -438.919645459 A.U.
G = -438.817073 A.U.

C	1.707345	-1.262350	0.227284
C	2.324742	-0.094397	0.413782
C	1.904437	1.226425	-0.057209
C	0.630862	1.605731	-0.267096
C	-0.487720	0.700957	-0.126809
C	-0.496634	-0.679711	-0.203939
C	-2.578136	-0.025955	0.255950
C	-1.803432	-1.149644	0.044435
H	2.083968	-2.187731	0.648643
H	3.237135	-0.135774	0.999467
H	2.686720	1.969848	-0.161220
H	0.416794	2.637635	-0.526855
H	-3.630511	0.067958	0.466507
H	-2.138005	-2.174256	0.034824
O	0.587548	-1.455038	-0.565234
N	-1.782686	1.073301	0.149005
H	-2.086470	2.023171	0.299086

T1
E = -438.861396726 A.U.
G = -438.762784 A.U.

C	1.840887	-1.262997	-0.000090
C	2.456271	-0.003958	0.000160
C	1.935260	1.269508	0.000131
C	0.542379	1.637107	-0.000138
C	-0.494216	0.721553	-0.000087
C	-0.504381	-0.709283	0.000044
C	-2.635688	-0.039445	0.000017
C	-1.839822	-1.160546	0.000117
H	2.430287	-2.167103	-0.000221
H	3.538439	-0.085439	0.000347
H	2.646690	2.086315	0.000369
H	0.288059	2.690108	-0.000345
H	-3.709469	0.049806	0.000056
H	-2.163066	-2.188567	0.000202
O	0.524896	-1.576571	-0.000094
N	-1.837891	1.070536	-0.000066
H	-2.169016	2.022061	-0.000117

9 (X = O X' = O)

S0

E = -458.769072662 A.U.

G = -458.679076 A.U.

C	1.690865	-1.272018	0.220649
C	2.306405	-0.103481	0.403715
C	1.888911	1.223195	-0.057063
C	0.617028	1.609034	-0.257837
C	-0.482624	0.685684	-0.126289
C	-0.502458	-0.675394	-0.207185
C	-2.537612	0.037803	0.258863
C	-1.834914	-1.106681	0.054826
H	2.069862	-2.198240	0.635967
H	3.222165	-0.147854	0.983482
H	2.674458	1.963000	-0.159158
H	0.383381	2.638287	-0.505164
H	-3.575089	0.232235	0.471898
H	-2.211741	-2.116022	0.059536
O	0.559029	-1.470307	-0.561608
O	-1.738610	1.125276	0.158529

T1

E = -458.715243694 A.U.

G = -458.628850 A.U.

C	-1.823753	-1.265913	-0.000135
C	-2.434834	-0.010025	0.000022
C	-1.915763	1.267753	0.000099
C	-0.522581	1.632789	-0.000032
C	0.489957	0.709723	-0.000039
C	0.508741	-0.709967	0.000048
C	2.596106	0.018681	0.000114
C	1.861476	-1.122976	0.000026
H	-2.413718	-2.169390	-0.000281
H	-3.516679	-0.091503	0.000075
H	-2.626881	2.083894	0.000287
H	-0.245955	2.679040	-0.000132
H	3.657385	0.200590	0.000177
H	2.226488	-2.136787	0.000014
O	-0.506722	-1.584510	-0.000066
O	1.802129	1.123731	-0.000028

---

9 (X = O X' = S)

S0

E = -781.750109855 A.U.

G = -781.664345 A.U.

C	-2.062189	1.138011	0.278524
C	-2.555860	-0.081115	0.491034
C	-2.031172	-1.330089	-0.056894
C	-0.738242	-1.568043	-0.341160
C	0.305918	-0.571852	-0.184509
C	0.160565	0.790218	-0.249305
C	2.411159	0.692177	0.214349
C	1.354838	1.521708	-0.009528
H	-2.469703	2.031374	0.737279
H	-3.422083	-0.145658	1.140381
H	-2.736972	-2.146515	-0.162895
H	-0.440823	-2.560213	-0.663083

H	3.438426	0.957493	0.409763
H	1.410883	2.600686	-0.034285
S	1.958493	-0.969311	0.159651
O	-1.023215	1.403216	-0.604580

T1
E = -781.692865359 A.U.
G = -781.610803 A.U.

C	-2.241164	1.119503	-0.000052
C	-2.725251	-0.188266	0.000641
C	-2.061942	-1.395924	0.000244
C	-0.637352	-1.592010	-0.000388
C	0.304178	-0.587580	-0.000232
C	0.163586	0.831192	0.000046
C	2.473035	0.709884	0.000209
C	1.389685	1.532998	0.000263
H	-2.919241	1.959680	-0.000185
H	-3.809339	-0.223765	0.001185
H	-2.669597	-2.292046	0.000604
H	-0.279809	-2.615047	-0.000850
H	3.515180	0.987829	0.000323
H	1.437824	2.613093	0.000454
S	2.030730	-0.970157	-0.000185
O	-0.969418	1.564249	-0.000368

---

9 (X = S X' = NH)
S0
E = -761.900668364 A.U.
G = -761.801712 A.U.

C	2.003075	-0.740256	0.585520
C	2.251156	0.573248	0.604809
C	1.598366	1.643199	-0.146703
C	0.290713	1.746168	-0.456348
C	-0.719791	0.747476	-0.185936
C	-0.612984	-0.632890	-0.105156
C	-2.735464	-0.080351	0.356061
C	-1.882541	-1.156589	0.247509
H	2.538077	-1.411677	1.247069
H	3.033065	0.905174	1.284641
H	2.224376	2.502969	-0.362972
H	-0.057613	2.677049	-0.895023
H	-3.785541	-0.037188	0.594057
H	-2.140268	-2.195890	0.373829
N	-2.028467	1.054850	0.089267
H	-2.396016	1.992473	0.154865
S	0.851750	-1.526055	-0.526242

T1
E = -761.840314597 A.U.
G = -761.746223 A.U.

C	2.196054	-0.773020	0.000028
C	2.474230	0.608153	0.000043
C	1.662092	1.716891	0.000028
C	0.232557	1.831787	-0.000004
C	-0.714095	0.817119	-0.000010
C	-0.628938	-0.610093	0.000008
C	-2.808323	-0.061225	-0.000058

C	-1.949618	-1.131186	0.000019
H	3.027786	-1.465877	0.000036
H	3.540237	0.816960	0.000073
H	2.183161	2.667890	0.000046
H	-0.163098	2.841640	-0.000019
H	-3.885732	-0.035384	-0.000090
H	-2.228076	-2.172996	0.000042
N	-2.075012	1.090410	-0.000018
H	-2.459695	2.022147	0.000004
S	0.732922	-1.668612	-0.000018

---

9 (X = S X' = O)
S0
E = -781.749888006 A.U.
G = -781.663615 A.U.

C	1.985155	-0.741347	0.585173
C	2.233949	0.571352	0.597006
C	1.583299	1.642462	-0.155914
C	0.274759	1.747620	-0.456314
C	-0.711499	0.731107	-0.182911
C	-0.617073	-0.630257	-0.109956
C	-2.697135	-0.014134	0.353639
C	-1.912876	-1.116178	0.255427
H	2.516476	-1.409345	1.252644
H	3.014102	0.905196	1.277520
H	2.210700	2.500079	-0.375352
H	-0.097177	2.672477	-0.884802
H	-3.739550	0.128492	0.583669
H	-2.211884	-2.141863	0.396218
O	-1.986940	1.110074	0.095141
S	0.835711	-1.542461	-0.520496

T1
E = -781.694494118 A.U.
G = -781.612418 A.U.

C	-2.177108	-0.771566	-0.000069
C	-2.454485	0.604157	-0.000017
C	-1.643904	1.717851	0.000032
C	-0.214647	1.825838	0.000045
C	0.705096	0.803807	0.000027
C	0.630519	-0.611699	-0.000011
C	2.769427	0.000975	0.000105
C	1.971934	-1.093800	-0.000025
H	-3.010618	-1.462496	-0.000112
H	-3.520501	0.811473	-0.000022
H	-2.163888	2.668622	0.000062
H	0.207407	2.823598	0.000080
H	3.839859	0.120302	0.000162
H	2.292513	-2.123154	-0.000073
O	2.040722	1.146017	0.000066
S	-0.718221	-1.678741	-0.000072

---

9 (X = S X' = S)
S0
E = -1104.72871782 A.U.
G = -1104.646791 A.U.

C	2.296138	-0.562818	0.636224
C	2.390802	0.769120	0.676454
C	1.663868	1.744422	-0.130523
C	0.374227	1.686117	-0.513886
C	-0.543526	0.594346	-0.237436
C	-0.284800	-0.752450	-0.149680
C	-2.542413	-0.766110	0.350939
C	-1.426984	-1.531242	0.200696
H	2.860799	-1.184615	1.320614
H	3.086695	1.188946	1.399039
H	2.199021	2.664496	-0.343208
H	-0.048328	2.556251	-1.006657
H	-3.542589	-1.085239	0.600792
H	-1.408153	-2.606445	0.311043
S	-2.219009	0.905051	0.094776
S	1.299551	-1.443907	-0.549673

T1
E = -1104.66969128 A.U.
G = -1104.592073 A.U.

C	2.542317	-0.564392	-0.000269
C	2.665042	0.831312	0.000066
C	1.722276	1.834505	0.000306
C	0.291273	1.766555	-0.000082
C	-0.541049	0.663467	-0.000038
C	-0.296523	-0.738670	0.000141
C	-2.622978	-0.766067	0.000121
C	-1.488973	-1.514892	0.000255
H	3.446519	-1.160449	-0.000531
H	3.698623	1.164116	0.000177
H	2.118317	2.843441	0.000676
H	-0.208621	2.729395	-0.000317
H	-3.643154	-1.117250	0.000169
H	-1.491137	-2.597136	0.000398
S	-2.292193	0.933891	-0.000103
S	1.195390	-1.617206	-0.000120

---

10 (X = NH X' = NH)
S0
E = -419.032005076 A.U.
G = -418.917257 A.U.

C	-2.370427	-0.106584	-0.269763
C	-1.877937	1.147538	-0.388273
C	-0.596698	1.576174	0.090020
C	0.491714	0.744695	0.204906
C	0.476070	-0.690132	-0.075436
C	-0.599463	-1.512200	0.001342
N	-1.798905	-1.059045	0.639631
C	1.894550	1.112930	0.273712
C	2.617463	0.020803	-0.073096
N	1.791243	-1.046687	-0.369659
H	-3.257215	-0.425713	-0.806848
H	-2.462408	1.874112	-0.944894
H	-0.414424	2.644140	0.154890
H	-0.554793	-2.548251	-0.317588
H	-1.549370	-0.570222	1.498949
H	2.281952	2.100772	0.466033

H	3.687465	-0.091421	-0.162349
H	2.110792	-2.002638	-0.358475

T1
E = -419.019587760 A.U.
G = -418.907475 A.U.

C	2.484256	0.024236	-0.000004
C	1.902700	1.281724	-0.000042
C	0.556145	1.655735	-0.000029
C	-0.564399	0.773041	0.000016
C	-0.523263	-0.650459	-0.000022
C	0.542247	-1.553143	0.000088
N	1.876944	-1.211556	-0.000013
C	-1.944148	1.139049	0.000018
C	-2.693664	-0.009451	0.000030
N	-1.838453	-1.082175	-0.000089
H	3.563227	-0.046952	-0.000015
H	2.626224	2.089810	-0.000087
H	0.335192	2.717324	-0.000050
H	0.350934	-2.616658	0.000076
H	2.511370	-1.995261	0.000258
H	-2.329890	2.147304	-0.000003
H	-3.761407	-0.155352	0.000024
H	-2.124326	-2.048485	0.000186

---

10 (X = NH X' = O)
S0
E = -438.888764757 A.U.
G = -438.786102 A.U.

C	-2.359986	-0.091496	-0.279578
C	-1.867712	1.160429	-0.369288
C	-0.581146	1.582964	0.119387
C	0.497166	0.746945	0.205835
C	0.479987	-0.679481	-0.064979
C	-0.565715	-1.524550	0.006194
N	-1.788712	-1.074110	0.600041
C	1.914122	1.081170	0.256093
C	2.581275	-0.025932	-0.099208
H	-3.246577	-0.394240	-0.826155
H	-2.446324	1.898930	-0.915816
H	-0.400499	2.648062	0.217151
H	-0.480373	-2.563367	-0.291829
H	-1.573926	-0.623811	1.488670
H	2.342345	2.049146	0.460147
H	3.633890	-0.218205	-0.233205
O	1.763064	-1.097255	-0.343248

T1
E = -438.875039409 A.U.
G = -438.774937 A.U.

C	-2.463313	0.021462	-0.000122
C	-1.883879	1.284991	-0.000053
C	-0.543807	1.666602	0.000019
C	0.570922	0.775331	0.000004
C	0.520791	-0.631792	0.000008
C	-0.522534	-1.546238	0.000160
N	-1.853861	-1.211463	0.000016

C	1.965783	1.100176	-0.000029
C	2.649940	-0.069268	-0.000015
H	-3.541717	-0.051296	-0.000294
H	-2.611989	2.088575	-0.000109
H	-0.322655	2.727222	0.000085
H	-0.304988	-2.603695	0.000411
H	-2.486312	-1.997688	0.000102
H	2.394346	2.090376	-0.000042
H	3.699769	-0.309210	-0.000027
O	1.798395	-1.133953	-0.000010

---

10 (X = NH X' = S)
S0
E = -761.866402293 A.U.
G = -761.768113 A.U.

C	-2.611701	-0.299455	-0.283650
C	-2.241485	0.980838	-0.494692
C	-1.026977	1.567053	-0.002914
C	0.139612	0.874834	0.202033
C	0.283829	-0.562244	0.006506
C	-0.703358	-1.476426	0.126300
C	1.448339	1.498614	0.336753
C	2.468166	0.665262	0.082145
H	-3.447927	-0.750762	-0.806050
H	-2.873744	1.604774	-1.118410
H	-0.966293	2.650424	0.022876
H	-0.559627	-2.519582	-0.134254
H	1.574567	2.550884	0.554568
H	3.522073	0.902094	0.076358
S	1.982460	-0.975892	-0.305870
N	-1.961065	-1.111989	0.705736
H	-1.779508	-0.530489	1.523797

T1
E = -761.853448887 A.U.
G = -761.757452 A.U.

C	-2.752060	-0.166157	0.000042
C	-2.302617	1.147155	-0.000010
C	-1.005747	1.650855	0.000018
C	0.215165	0.903174	-0.000010
C	0.324828	-0.512407	0.000025
C	-0.649540	-1.505864	0.000088
C	1.500071	1.523654	-0.000016
C	2.546899	0.656039	-0.000048
H	-3.815717	-0.357212	0.000079
H	-3.102905	1.878722	0.000015
H	-0.896879	2.729773	0.000058
H	-0.355698	-2.546329	0.000124
H	1.625924	2.599327	-0.000035
H	3.602411	0.878581	-0.000059
S	2.017225	-0.996708	0.000010
N	-2.006943	-1.320142	-0.000139
H	-2.546128	-2.173231	0.000096

---

10 (X = O X' = NH)
S0

E = -438.892829422 A.U.
G = -438.791421 A.U.

C	-2.359853	-0.099429	-0.227090
C	-1.876836	1.143946	-0.367652
C	-0.583497	1.586884	0.091174
C	0.495207	0.757737	0.203977
C	0.467517	-0.684922	-0.058910
C	-0.609327	-1.487328	0.034497
C	1.906102	1.105190	0.273460
C	2.611852	0.005348	-0.075844
H	-3.260098	-0.462147	-0.710004
H	-2.472901	1.844345	-0.943592
H	-0.410132	2.655801	0.155746
H	-0.620399	-2.535421	-0.241071
H	2.307174	2.088538	0.460527
H	3.680330	-0.116875	-0.171851
O	-1.756210	-1.002160	0.646859
N	1.775900	-1.058066	-0.378931
H	2.087415	-2.015055	-0.313776

T1
E = -438.865175723 A.U.
G = -438.766467 A.U.

C	-2.459103	-0.029526	0.000026
C	-1.903967	1.234309	-0.000002
C	-0.577633	1.653896	-0.000050
C	0.558068	0.783758	-0.000027
C	0.507599	-0.633218	0.000020
C	-0.553172	-1.540874	0.000007
C	1.932280	1.146267	-0.000015
C	2.677801	-0.008995	0.000030
H	-3.532505	-0.151778	0.000057
H	-2.660925	2.011641	0.000023
H	-0.385346	2.720037	-0.000074
H	-0.384753	-2.607490	0.000015
H	2.322529	2.152447	-0.000095
H	3.744420	-0.161112	0.000007
O	-1.876675	-1.255250	-0.000024
N	1.817255	-1.073353	-0.000035
H	2.097963	-2.041976	0.000579

---

10 (X = O X' = O)
S0
E = -458.749552570 A.U.
G = -458.660273 A.U.

C	-2.351951	-0.081480	-0.233908
C	-1.864619	1.158913	-0.343061
C	-0.564052	1.598401	0.120947
C	0.505108	0.765314	0.199004
C	0.470257	-0.670588	-0.048844
C	-0.577348	-1.497618	0.043588
C	1.929740	1.072111	0.249722
C	2.576404	-0.047297	-0.101678
H	-3.260704	-0.419621	-0.717714
H	-2.459628	1.872852	-0.902861
H	-0.395276	2.664510	0.221765
H	-0.539901	-2.552526	-0.197984

H	2.376058	2.033674	0.444791
H	3.625334	-0.255271	-0.241439
O	-1.755066	-1.029241	0.598382
O	1.744176	-1.112027	-0.338530

T1
E = -458.717465170 A.U.
G = -458.630974 A.U.

C	-2.438787	-0.034419	0.000281
C	-1.887705	1.237014	0.000131
C	-0.568960	1.663849	-0.000105
C	0.564029	0.784916	-0.000077
C	0.506827	-0.615230	-0.000028
C	-0.531331	-1.535157	-0.000140
C	1.953497	1.108778	-0.000057
C	2.634085	-0.066184	0.000025
H	-3.511273	-0.160773	0.000554
H	-2.649831	2.008877	0.000263
H	-0.376137	2.728977	-0.000285
H	-0.337080	-2.596430	-0.000328
H	2.384993	2.097328	-0.000075
H	3.682487	-0.311595	0.000068
O	-1.850679	-1.255360	-0.000082
O	1.777792	-1.123113	0.000035

---

10 (X = O X' = S)
S0
E = -781.728604101 A.U.
G = -781.643608 A.U.

C	-2.607744	-0.286746	-0.239158
C	-2.246676	0.979622	-0.466400
C	-1.018180	1.583770	0.002563
C	0.140658	0.897749	0.188545
C	0.278055	-0.551087	0.013804
C	-0.697628	-1.454071	0.175894
C	1.458960	1.501325	0.339620
C	2.467486	0.655444	0.089330
H	-3.465896	-0.767955	-0.693370
H	-2.894217	1.577393	-1.098607
H	-0.976998	2.666860	0.050606
H	-0.576079	-2.518436	0.009096
H	1.598405	2.551574	0.558759
H	3.523968	0.880884	0.083987
S	1.971621	-0.984388	-0.313972
O	-1.925590	-1.074518	0.685986

T1
E = -781.696316636 A.U.
G = -781.614039 A.U.

C	2.718767	-0.219118	-0.000434
C	2.299272	1.098115	-0.000375
C	1.026506	1.646071	0.000149
C	-0.209266	0.912083	0.000178
C	-0.310549	-0.497387	0.000097
C	0.655476	-1.496288	0.000187
C	-1.489056	1.530299	0.000133
C	-2.532603	0.653715	-0.000000

H	3.771382	-0.460944	-0.000837
H	3.130477	1.794787	-0.000698
H	0.945745	2.726501	0.000426
H	0.381180	-2.541352	0.000430
H	-1.619702	2.604971	0.000166
H	-3.589386	0.869777	0.000003
S	-1.996610	-0.990372	-0.000052
O	1.996846	-1.364091	0.000217

10 (X = S X' = NH)
S0
E = -761.874109404 A.U.
G = -761.776357 A.U.

C	2.255436	0.524054	0.485072
C	1.448319	1.594480	0.448940
C	0.146711	1.715328	-0.168632
C	-0.804493	0.739991	-0.243888
C	-0.671286	-0.668704	0.172833
C	0.433636	-1.441131	0.205965
C	-2.232237	0.948640	-0.433981
C	-2.866416	-0.157830	0.012032
H	3.132547	0.495892	1.120450
H	1.768884	2.467068	1.015178
H	-0.188337	2.727129	-0.376847
H	0.403108	-2.443871	0.615983
H	-2.695623	1.868560	-0.752871
H	-3.924884	-0.362139	0.071631
N	-1.963138	-1.098128	0.480609
H	-2.193152	-2.078342	0.545212
S	1.948838	-0.907273	-0.529565

T1
E = -761.846127745 A.U.
G = -761.752144 A.U.

C	2.313900	0.673200	0.000204
C	1.442620	1.749496	-0.000078
C	0.055997	1.823182	-0.000194
C	-0.912711	0.770671	-0.000011
C	-0.715041	-0.639056	-0.000064
C	0.419667	-1.453632	-0.000267
C	-2.321414	0.983946	0.000072
C	-2.942357	-0.239885	0.000159
H	3.378281	0.875627	0.000424
H	1.953936	2.707886	-0.000137
H	-0.364253	2.823281	-0.000383
H	0.292080	-2.529109	-0.000494
H	-2.811399	1.945525	0.000031
H	-3.987519	-0.502149	0.000227
N	-1.975799	-1.205877	-0.000067
H	-2.151768	-2.199029	0.000663
S	2.092329	-1.043027	0.000076

10 (X = S X' = O)
S0
E = -781.730669719 A.U.
G = -781.645268 A.U.

C	2.231211	0.549695	0.481601
C	1.425461	1.616612	0.417984
C	0.120147	1.725700	-0.203297
C	-0.817688	0.742753	-0.242696
C	-0.667543	-0.656300	0.157952
C	0.414881	-1.444494	0.193325
C	-2.257683	0.906176	-0.405758
C	-2.825771	-0.217522	0.048275
H	3.108490	0.541829	1.116829
H	1.741017	2.499821	0.969868
H	-0.217279	2.729590	-0.440160
H	0.344643	-2.458923	0.566232
H	-2.768200	1.797418	-0.732645
H	-3.859256	-0.503621	0.162999
S	1.953660	-0.915595	-0.496268
O	-1.918258	-1.161539	0.451606

T1
E = -781.698863981 A.U.
G = -781.616928 A.U.

C	-2.292566	0.670957	0.000253
C	-1.422240	1.754708	0.000133
C	-0.040341	1.833221	-0.000091
C	0.917677	0.768771	-0.000120
C	0.708851	-0.622399	0.000054
C	-0.405455	-1.447999	0.000154
C	2.338488	0.938681	-0.000196
C	2.888803	-0.299067	-0.000059
H	-3.356821	0.873348	0.000416
H	-1.938313	2.710116	0.000246
H	0.384052	2.830633	-0.000206
H	-0.249577	-2.518815	0.000226
H	2.869741	1.877475	-0.000323
H	3.905307	-0.654825	-0.000055
S	-2.073573	-1.040842	-0.000111
O	1.925436	-1.255712	0.000089

---

10 (X = S X' = S)
S0
E = -1104.70691911 A.U.
G = -1104.625785 A.U.

C	2.536763	0.329453	0.549057
C	1.839000	1.469967	0.597183
C	0.583363	1.769031	-0.060404
C	-0.459797	0.914488	-0.241627
C	-0.480532	-0.518550	0.083535
C	0.530513	-1.399109	0.045689
C	-1.814545	1.369077	-0.539939
C	-2.762803	0.478492	-0.222087
H	3.396101	0.161455	1.186810
H	2.219340	2.255029	1.247138
H	0.379587	2.820208	-0.242066
H	0.387649	-2.432537	0.340857
H	-2.024310	2.366588	-0.902419
H	-3.832614	0.608993	-0.296944
S	-2.152433	-1.036871	0.425090
S	2.130088	-0.979181	-0.587704

T1
E = -1104.67463529 A.U.
G = -1104.596955 A.U.

C	2.647570	0.492510	-0.000173
C	1.895684	1.656879	-0.000160
C	0.528186	1.877812	-0.000060
C	-0.569525	0.951353	0.000131
C	-0.527524	-0.464146	-0.000010
C	0.518853	-1.382611	0.000236
C	-1.914343	1.430816	0.000174
C	-2.862138	0.456096	0.000128
H	3.727518	0.576380	-0.000281
H	2.504731	2.555904	-0.000295
H	0.225833	2.919502	-0.000123
H	0.283511	-2.440410	0.000400
H	-2.153166	2.486762	0.000298
H	-3.935284	0.566965	0.000124
S	-2.159570	-1.120826	-0.000019
S	2.224961	-1.177759	-0.000089

---

11 (X = NH X' = NH)
S0
E = -419.066391059 A.U.
G = -418.951249 A.U.

C	-1.887224	1.226888	-0.028865
C	-0.625100	1.591362	-0.267218
C	0.557528	0.742468	-0.086028
C	0.552515	-0.641719	-0.099161
C	-0.564654	-1.565204	-0.272599
C	-1.840724	-1.254601	-0.019049
N	-2.333589	-0.026470	0.453507
C	1.909838	1.155429	0.083771
C	2.681188	0.019067	0.173006
N	1.846587	-1.058915	0.056918
H	-2.689592	1.939567	-0.185700
H	-0.464766	2.603148	-0.622222
H	-0.350271	-2.568184	-0.622376
H	-2.606094	-2.011155	-0.154653
H	-3.332659	-0.045609	0.585753
H	2.270311	2.172067	0.128510
H	3.742446	-0.110610	0.305218
H	2.139440	-2.023671	0.089361

T1
E = -419.012446726 A.U.
G = -418.900515 A.U.

C	1.917373	1.237075	0.000028
C	0.570528	1.598643	0.000002
C	-0.552846	0.763935	-0.000008
C	-0.549946	-0.688251	0.000003
C	0.514204	-1.575135	-0.000023
C	1.884220	-1.264509	-0.000018
N	2.453818	-0.018444	-0.000003
C	-1.932959	1.163348	0.000010
C	-2.700693	0.035770	0.000009
N	-1.886358	-1.074565	-0.000013

H	2.667744	2.014982	0.000075
H	0.386424	2.667316	0.000003
H	0.282822	-2.634722	-0.000033
H	2.607069	-2.066246	-0.000124
H	3.463714	-0.031803	0.000062
H	-2.298147	2.178842	0.000012
H	-3.773711	-0.074011	0.000018
H	-2.207420	-2.028546	0.000087

---

11 (X = NH X' = O)

S0

E = -438.918070055 A.U.

G = -438.815318 A.U.

C	-1.869465	1.226250	-0.035020
C	-0.613367	1.597209	-0.289727
C	0.560917	0.741345	-0.090508
C	0.546806	-0.622309	-0.112570
C	-0.549422	-1.559455	-0.292817
C	-1.820840	-1.248886	-0.016108
N	-2.290482	-0.022257	0.484907
C	1.933333	1.116050	0.104342
C	2.633471	-0.041786	0.186035
H	-2.681338	1.924013	-0.209241
H	-0.453300	2.595307	-0.679037
H	-0.318777	-2.551398	-0.658961
H	-2.593265	-1.997866	-0.153322
H	-3.284028	-0.041383	0.655349
H	2.334922	2.115137	0.167767
H	3.676525	-0.269603	0.325422
O	1.803004	-1.108615	0.054489

T1

E = -438.871390334 A.U.

G = -438.771145 A.U.

C	1.898381	1.239986	-0.000089
C	0.557444	1.609451	-0.000014
C	-0.558497	0.767796	0.000017
C	-0.546913	-0.670716	-0.000006
C	0.493088	-1.571641	0.000096
C	1.861966	-1.264937	0.000080
N	2.432315	-0.018656	0.000001
C	-1.950589	1.126156	0.000093
C	-2.659243	-0.022759	-0.000110
H	2.652597	2.014057	-0.000196
H	0.373976	2.677435	0.000002
H	0.236779	-2.624123	0.000135
H	2.583160	-2.067818	0.000289
H	3.442362	-0.033153	-0.000131
H	-2.358090	2.125060	0.000200
H	-3.718312	-0.222377	-0.000208
O	-1.851563	-1.127313	-0.000063

---

11 (X = NH X' = S)

S0

E = -761.895904274 A.U.

G = -761.797470 A.U.

C	-2.266925	1.097708	-0.005493
C	-1.054365	1.573585	-0.296120
C	0.220164	0.858808	-0.118928
C	0.353234	-0.507784	-0.140560
C	-0.689078	-1.520054	-0.320045
C	-1.979379	-1.346554	-0.015349
C	1.474172	1.522596	0.059502
C	2.523752	0.663827	0.174931
H	-3.135603	1.733211	-0.139874
H	-0.999534	2.589484	-0.669671
H	-0.394348	-2.489205	-0.703398
H	-2.669178	-2.172913	-0.148982
H	1.577854	2.599629	0.091500
H	3.568741	0.892904	0.313774
N	-2.573056	-0.182927	0.498913
H	-3.559817	-0.300510	0.668542
S	2.008364	-0.976556	0.066756

T1
E = -761.849796635 A.U.
G = -761.753641 A.U.

C	2.310899	1.104057	-0.000192
C	1.012927	1.592358	-0.000059
C	-0.201842	0.891757	0.000015
C	-0.350396	-0.550280	0.000014
C	0.629626	-1.528169	0.000136
C	2.018588	-1.369968	0.000104
C	-1.478867	1.540908	0.000052
C	-2.537757	0.698789	-0.000041
H	3.131854	1.806814	-0.000370
H	0.940762	2.674438	-0.000069
H	0.303013	-2.562478	0.000252
H	2.645484	-2.249100	0.000332
H	-1.582450	2.618901	0.000073
H	-3.586557	0.953476	-0.000106
N	2.722883	-0.197842	-0.000078
H	3.726620	-0.316316	-0.000259
S	-2.066123	-0.988597	0.000033

---

11 (X = O X' = NH)
S0
E = -438.924026422 A.U.
G = -438.821346 A.U.

C	-1.903827	1.157649	-0.045987
C	-0.661328	1.556012	-0.315002
C	0.522575	0.743602	-0.071172
C	0.518926	-0.647187	-0.075304
C	-0.602204	-1.533445	-0.321568
C	-1.860149	-1.186384	-0.046936
C	1.878421	1.158059	0.073433
C	2.645259	0.021369	0.163142
H	-2.784058	1.743917	-0.282467
H	-0.537516	2.530013	-0.775609
H	-0.425057	-2.496171	-0.787568
H	-2.711784	-1.812997	-0.285783
H	2.241280	2.173592	0.108761
H	3.706934	-0.105509	0.296233

O	-2.192537	-0.024720	0.630102
N	1.815874	-1.061259	0.060599
H	2.113330	-2.024324	0.097787

T1
E = -438.853866544 A.U.
G = -438.755912 A.U.

C	1.924725	1.205309	-0.000026
C	0.590987	1.574021	0.000011
C	-0.544764	0.765696	-0.000003
C	-0.544194	-0.695010	-0.000027
C	0.528618	-1.555847	0.000015
C	1.894139	-1.232844	0.000058
C	-1.920497	1.168794	0.000034
C	-2.687012	0.040765	0.000021
H	2.701295	1.956240	-0.000050
H	0.435719	2.647607	0.000032
H	0.327793	-2.621766	0.000030
H	2.645024	-2.007379	0.000083
H	-2.285947	2.183616	0.000032
H	-3.760605	-0.064597	0.000033
O	2.481420	-0.018691	-0.000024
N	-1.879853	-1.077219	-0.000108
H	-2.207687	-2.028968	0.000282

---

11 (X = O X' = O)
S0
E = -458.774545499 A.U.
G = -458.684533 A.U.

C	-1.886900	1.158391	-0.049808
C	-0.647350	1.564145	-0.320345
C	0.527422	0.741786	-0.071053
C	0.514218	-0.628385	-0.078232
C	-0.586224	-1.530735	-0.324416
C	-1.843178	-1.179729	-0.047327
C	1.905530	1.117799	0.083333
C	2.602430	-0.040096	0.164656
H	-2.772069	1.734320	-0.292179
H	-0.518829	2.532442	-0.789450
H	-0.390620	-2.488885	-0.789632
H	-2.699625	-1.798782	-0.287470
H	2.310031	2.116213	0.128544
H	3.647091	-0.265947	0.295707
O	-2.160029	-0.019678	0.639693
O	1.773570	-1.111374	0.059511

T1
E = -458.711502003 A.U.
G = -458.625211 A.U.

C	1.906206	1.208895	-0.000035
C	0.577925	1.586424	-0.000041
C	-0.550759	0.770887	0.000005
C	-0.540916	-0.676329	0.000008
C	0.507100	-1.553076	0.000003
C	1.871952	-1.234021	-0.000022
C	-1.938229	1.131191	0.000027
C	-2.646098	-0.019025	0.000023

H	2.687779	1.954400	-0.000054
H	0.423810	2.659400	-0.000070
H	0.280028	-2.612536	-0.000006
H	2.621899	-2.009103	-0.000007
H	-2.347205	2.129039	0.000038
H	-3.705840	-0.215355	0.000040
O	2.458092	-0.019479	0.000009
O	-1.843537	-1.129960	0.000023

11 (X = O X' = S)
S0
E = -781.752477016 A.U.
G = -781.666791 A.U.

C	-2.278213	1.020496	-0.020284
C	-1.091089	1.534652	-0.332852
C	0.183882	0.857648	-0.105045
C	0.326055	-0.514433	-0.117613
C	-0.718694	-1.497472	-0.358489
C	-1.995709	-1.291310	-0.038021
C	1.437754	1.530171	0.049827
C	2.487321	0.674163	0.161179
H	-3.217195	1.519988	-0.228474
H	-1.074145	2.512650	-0.800606
H	-0.461627	-2.431647	-0.843749
H	-2.782637	-2.002717	-0.260357
H	1.537772	2.607186	0.076034
H	3.531197	0.908270	0.301380
O	-2.428666	-0.178968	0.662457
S	1.986757	-0.972842	0.063994

T1
E = -781.689644353 A.U.
G = -781.607437 A.U.

C	2.318425	1.065417	-0.000157
C	1.034429	1.564684	-0.000115
C	-0.191775	0.895291	-0.000019
C	-0.348724	-0.555175	-0.000039
C	0.641171	-1.510533	0.000021
C	2.026933	-1.343936	-0.000027
C	-1.462312	1.550228	0.000084
C	-2.521096	0.707346	0.000055
H	3.166349	1.734531	-0.000255
H	0.995185	2.648841	-0.000152
H	0.341318	-2.553290	0.000068
H	2.682498	-2.201200	0.000044
H	-1.564643	2.627808	0.000100
H	-3.568965	0.966834	0.000073
O	2.748349	-0.208784	-0.000068
S	-2.063802	-0.987074	0.000115

11 (X = S X' = NH)
S0
E = -761.903279161 A.U.
G = -761.804552 A.U.

C	1.609580	1.375277	0.303630
C	0.307520	1.616523	0.483302

C	-0.795656	0.745993	0.109306
C	-0.787001	-0.651033	0.113729
C	0.257052	-1.581761	0.494450
C	1.566798	-1.387343	0.311329
C	-2.137917	1.152849	-0.147404
C	-2.889945	0.015919	-0.306405
H	2.364250	2.050359	0.688325
H	0.038671	2.539547	0.991604
H	-0.056971	-2.486456	1.009238
H	2.293554	-2.089603	0.701204
H	-2.496418	2.168521	-0.210244
H	-3.934257	-0.113854	-0.537994
S	2.241862	-0.020601	-0.608174
N	-2.071287	-1.061678	-0.127369
H	-2.362202	-2.025692	-0.191386

T1
E = -761.835546440 A.U.
G = -761.741539 A.U.

C	1.592729	1.419005	0.000179
C	0.225508	1.654032	-0.000015
C	-0.852018	0.766197	-0.000067
C	-0.845340	-0.694571	0.000038
C	0.176026	-1.624326	-0.000040
C	1.565395	-1.429033	-0.000053
C	-2.233528	1.159298	-0.000156
C	-2.997694	0.031568	-0.000046
H	2.262803	2.269907	0.000309
H	-0.045078	2.706199	-0.000069
H	-0.131674	-2.666177	-0.000061
H	2.208378	-2.299354	-0.000161
H	-2.597541	2.174815	-0.000248
H	-4.070482	-0.080340	-0.000023
S	2.523102	-0.015475	0.000019
N	-2.182854	-1.074429	0.000096
H	-2.502522	-2.029464	0.000242

---

11 (X = S X' = O)
S0
E = -781.753336602 A.U.
G = -781.667289 A.U.

C	1.593937	1.376392	0.308785
C	0.293773	1.623113	0.487197
C	-0.800283	0.743194	0.107778
C	-0.782324	-0.631794	0.116546
C	0.240394	-1.579171	0.494503
C	1.550095	-1.383161	0.313068
C	-2.162737	1.113287	-0.163212
C	-2.846305	-0.044611	-0.308540
H	2.349544	2.047323	0.698705
H	0.018710	2.540476	1.000514
H	-0.094624	-2.479765	1.000494
H	2.276757	-2.085953	0.701935
H	-2.560490	2.112301	-0.241967
H	-3.873656	-0.272153	-0.537793
S	2.224948	-0.017395	-0.609075
O	-2.029338	-1.110928	-0.126680

T1
E = -781.692810076 A.U.
G = -781.610640 A.U.

C	1.574948	1.423446	-0.000326
C	0.212181	1.665136	-0.000010
C	-0.856265	0.768822	0.000127
C	-0.839511	-0.677309	-0.000005
C	0.157126	-1.620248	0.000094
C	1.545344	-1.429473	0.000087
C	-2.250268	1.121300	0.000231
C	-2.954687	-0.027788	0.000187
H	2.248685	2.271484	-0.000550
H	-0.059552	2.716287	0.000047
H	-0.177718	-2.652240	0.000182
H	2.185963	-2.301440	0.000269
H	-2.656100	2.120568	0.000321
H	-4.013341	-0.229972	0.000209
S	2.506719	-0.015734	-0.000171
O	-2.146081	-1.127031	-0.000005

---

11 (X = S X' = S)
S0
E = -1104.72941794 A.U.
G = -1104.647762 A.U.

C	1.993824	1.283386	0.277627
C	0.729614	1.630013	0.526235
C	-0.469936	0.874984	0.169980
C	-0.585968	-0.502939	0.193483
C	0.402200	-1.500926	0.575079
C	1.711170	-1.433553	0.317816
C	-1.720779	1.515984	-0.108521
C	-2.737608	0.638054	-0.305716
H	2.819146	1.891526	0.627816
H	0.562021	2.562172	1.060049
H	0.041975	-2.359083	1.135721
H	2.388937	-2.192096	0.690473
H	-1.837160	2.590361	-0.161444
H	-3.765721	0.850003	-0.555736
S	-2.219527	-0.993752	-0.126482
S	2.460508	-0.154554	-0.665567

T1
E = -1104.66816540 A.U.
G = -1104.590106 A.U.

C	1.984920	1.343694	-0.000136
C	0.645772	1.685976	-0.000017
C	-0.517028	0.906773	0.000050
C	-0.634178	-0.544072	-0.000006
C	0.319473	-1.542984	0.000109
C	1.715432	-1.474202	0.000059
C	-1.813579	1.522670	0.000114
C	-2.850522	0.656735	0.000062
H	2.717027	2.141786	-0.000222
H	0.470107	2.758080	0.000003
H	-0.059555	-2.561247	0.000215
H	2.271452	-2.402894	0.000095

H	-1.941547	2.597625	0.000171
H	-3.904522	0.889815	0.000064
S	-2.342563	-1.013030	-0.000020
S	2.801643	-0.158890	-0.000089

---

12 (X = NH X' = NH)
S0
E = -550.634350545 A.U.
G = -550.491362 A.U.

C	-0.590804	0.262692	1.179654
C	0.737319	0.073080	1.526050
C	1.901326	0.275741	0.673180
C	1.901326	0.275741	-0.673180
C	0.737319	0.073080	-1.526050
C	-0.590804	0.262692	-1.179654
N	-1.182094	0.775211	-0.000000
N	-1.367804	-0.095180	2.241351
C	-0.574127	-0.500723	3.282791
C	0.732188	-0.410391	2.873780
C	0.732188	-0.410391	-2.873780
C	-0.574127	-0.500723	-3.282791
N	-1.367804	-0.095180	-2.241351
H	2.853230	0.378594	1.185575
H	2.853230	0.378594	-1.185575
H	-1.196059	1.792182	-0.000000
H	-2.374707	-0.034305	2.246381
H	-1.004496	-0.817943	4.218033
H	1.600547	-0.671803	3.459358
H	1.600547	-0.671803	-3.459358
H	-1.004496	-0.817943	-4.218033
H	-2.374707	-0.034305	-2.246381

T1
E = -550.558086845 A.U.
G = -550.423925 A.U.

C	0.620421	0.000617	1.219201
C	-0.764737	-0.000056	1.559169
C	-1.882124	0.001365	0.722004
C	-1.882124	0.001365	-0.722004
C	-0.764737	-0.000056	-1.559169
C	0.620421	0.000617	-1.219201
N	1.238512	0.003541	0.000000
N	1.363756	0.001170	2.379743
C	0.507596	-0.002092	3.460325
C	-0.775229	-0.001861	3.010630
C	-0.775229	-0.001861	-3.010630
C	0.507596	-0.002092	-3.460325
N	1.363756	0.001170	-2.379743
H	-2.848375	0.001761	1.213962
H	-2.848375	0.001761	-1.213962
H	2.249698	0.011825	0.000000
H	2.369426	-0.010280	2.435261
H	0.901160	-0.003724	4.463736
H	-1.663705	-0.002097	3.622802
H	-1.663705	-0.002097	-3.622802
H	0.901160	-0.003724	-4.463736
H	2.369426	-0.010280	-2.435261

---

12 (X = NH X' = O)
S0
E = -590.336550881 A.U.
G = -590.219761 A.U.

C	-0.573414	0.229516	1.179225
C	0.736218	0.044925	1.512830
C	1.917589	0.247665	0.669665
C	1.917589	0.247665	-0.669665
C	0.736218	0.044925	-1.512830
C	-0.573414	0.229516	-1.179225
N	-1.173441	0.677309	0.000000
C	-0.600218	-0.404080	3.263890
C	0.697116	-0.372737	2.894541
C	0.697116	-0.372737	-2.894541
C	-0.600218	-0.404080	-3.263890
H	2.856607	0.393728	1.192730
H	2.856607	0.393728	-1.192730
H	-2.184500	0.613564	0.000000
H	-1.119949	-0.639213	4.175680
H	1.546453	-0.613063	3.514549
H	1.546453	-0.613063	-3.514549
H	-1.119949	-0.639213	-4.175680
O	-1.393446	-0.036319	-2.204493
O	-1.393446	-0.036319	2.204493

T1
E = -590.275139468 A.U.
G = -590.161742 A.U.

C	-0.605897	0.000006	1.198942
C	0.763634	-0.000011	1.540311
C	1.889306	0.000006	0.717941
C	1.889306	0.000006	-0.717941
C	0.763634	-0.000011	-1.540311
C	-0.605897	0.000006	-1.198942
N	-1.241467	0.000000	-0.000000
C	-0.557357	0.000009	3.379521
C	0.729488	-0.000006	2.995455
C	0.729488	-0.000006	-2.995455
C	-0.557357	0.000009	-3.379521
H	2.851880	-0.000006	1.214953
H	2.851880	-0.000006	-1.214953
H	-2.255304	-0.000311	-0.000000
H	-1.040740	0.000024	4.341652
H	1.590076	-0.000035	3.645601
H	1.590076	-0.000035	-3.645601
H	-1.040740	0.000024	-4.341652
O	-1.405433	0.000017	-2.298447
O	-1.405433	0.000017	2.298447

---

12 (X = NH X' = S)
S0
E = -1236.28769399 A.U.
G = -1236.179031 A.U.

C	-0.316356	0.420899	1.188620
C	0.972708	0.099835	1.526511

C	2.137188	0.350273	0.671187
C	2.137188	0.350273	-0.671187
C	0.972708	0.099835	-1.526511
C	-0.316356	0.420899	-1.188620
N	-0.701440	1.060991	0.000000
C	-0.177753	-0.664870	3.409431
C	1.035624	-0.517841	2.820154
C	1.035624	-0.517841	-2.820154
C	-0.177753	-0.664870	-3.409431
H	3.083439	0.487879	1.185560
H	3.083439	0.487879	-1.185560
H	-1.618545	1.483975	0.000000
H	-0.411937	-1.064011	4.383742
H	1.963688	-0.826872	3.283220
H	1.963688	-0.826872	-3.283220
H	-0.411937	-1.064011	-4.383742
S	-1.454959	-0.073889	-2.400976
S	-1.454959	-0.073889	2.400976

T1
E = -1236.22772088 A.U.
G = -1236.122576 A.U.

C	-0.399846	0.000168	1.231315
C	0.983575	0.000032	1.565312
C	2.086048	0.000065	0.709172
C	2.086048	0.000065	-0.709172
C	0.983575	0.000032	-1.565312
C	-0.399846	0.000168	-1.231315
N	-0.988783	0.000311	0.000000
C	-0.037402	-0.000095	3.688614
C	1.122491	-0.000115	3.006445
C	1.122491	-0.000115	-3.006445
C	-0.037402	-0.000095	-3.688614
H	3.055887	0.000055	1.193924
H	3.055887	0.000055	-1.193924
H	-2.001602	0.001230	0.000000
H	-0.186619	-0.000136	4.757228
H	2.092532	-0.000152	3.486772
H	2.092532	-0.000152	-3.486772
H	-0.186619	-0.000136	-4.757228
S	-1.439341	-0.000112	-2.645403
S	-1.439341	-0.000112	2.645403

---

12 (X = O X' = NH)
S0
E = -570.490478816 A.U.
G = -570.360705 A.U.

C	-0.572270	0.240440	1.149193
C	0.746971	0.060202	1.508350
C	1.931104	0.231293	0.671096
C	1.931104	0.231293	-0.671096
C	0.746971	0.060202	-1.508350
C	-0.572270	0.240440	-1.149193
C	-0.604199	-0.424813	3.271244
C	0.705683	-0.366711	2.877608
C	0.705683	-0.366711	-2.877608
C	-0.604199	-0.424813	-3.271244

H	2.876280	0.328198	1.195312
H	2.876280	0.328198	-1.195312
H	-1.056279	-0.692129	4.211089
H	1.563554	-0.607755	3.486425
H	1.563554	-0.607755	-3.486425
H	-1.056279	-0.692129	-4.211089
O	-1.144981	0.719468	-0.000000
N	-1.380176	-0.052985	2.196012
H	-2.386140	0.022248	2.179259
N	-1.380176	-0.052985	-2.196012
H	-2.386140	0.022248	-2.179259

T1
E = -570.408940198 A.U.
G = -570.285249 A.U.

C	0.602163	-0.000021	1.175989
C	-0.770501	0.000020	1.537979
C	-1.902639	-0.000024	0.728929
C	-1.902639	-0.000024	-0.728929
C	-0.770501	0.000020	-1.537979
C	0.602163	-0.000021	-1.175989
C	0.554505	0.000044	3.406334
C	-0.739700	0.000052	2.992342
C	-0.739700	0.000052	-2.992342
C	0.554505	0.000044	-3.406334
H	-2.863390	-0.000032	1.229198
H	-2.863390	-0.000032	-1.229198
H	0.975680	0.000061	4.398218
H	-1.612315	0.000021	3.626452
H	-1.612315	0.000021	-3.626452
H	0.975680	0.000061	-4.398218
O	1.250195	-0.000072	-0.000000
N	1.378568	-0.000097	2.300472
H	2.386301	0.000485	2.301234
N	1.378568	-0.000097	-2.300472
H	2.386301	0.000485	-2.301234

---

12 (X = O X' = O)
S0
E = -610.193300348 A.U.
G = -610.088267 A.U.

C	-0.556642	0.253529	1.134947
C	0.739809	0.052080	1.491746
C	1.935304	0.233534	0.669874
C	1.935304	0.233534	-0.669874
C	0.739809	0.052080	-1.491746
C	-0.556642	0.253529	-1.134947
C	-0.655341	-0.426526	3.186286
C	0.654823	-0.398753	2.861289
C	0.654823	-0.398753	-2.861289
C	-0.655341	-0.426526	-3.186286
H	2.873530	0.345739	1.201063
H	2.873530	0.345739	-1.201063
H	-1.202474	-0.682007	4.076467
H	1.481736	-0.664860	3.500262
H	1.481736	-0.664860	-3.500262
H	-1.202474	-0.682007	-4.076467

O	-1.137569	0.730867	-0.000000
O	-1.413779	-0.025691	-2.118106
O	-1.413779	-0.025691	2.118106

T1
E = -610.119708771 A.U.
G = -610.019308 A.U.

C	-0.595269	0.000110	1.158868
C	0.764826	-0.000013	1.519770
C	1.901694	-0.000139	0.723783
C	1.901694	-0.000139	-0.723783
C	0.764826	-0.000013	-1.519770
C	-0.595269	0.000110	-1.158868
C	-0.595686	0.000004	3.328407
C	0.699072	0.000196	2.976294
C	0.699072	0.000196	-2.976294
C	-0.595686	0.000004	-3.328407
H	2.858849	-0.000198	1.229112
H	2.858849	-0.000198	-1.229112
H	-1.101728	0.000003	4.278695
H	1.546286	0.000353	3.643223
H	1.546286	0.000353	-3.643223
H	-1.101728	0.000003	-4.278695
O	-1.252391	-0.000483	0.000000
O	-1.417707	0.000103	-2.227644
O	-1.417707	0.000103	2.227644

12 (X = O X' = S)
S0
E = -1256.14277491 A.U.
G = -1256.046342 A.U.

C	-0.289246	0.403977	1.140668
C	0.989414	0.090972	1.506732
C	2.171005	0.306199	0.671260
C	2.171005	0.306199	-0.671260
C	0.989414	0.090972	-1.506732
C	-0.289246	0.403977	-1.140668
C	-0.234416	-0.643568	3.355657
C	1.001688	-0.508437	2.811767
C	1.001688	-0.508437	-2.811767
C	-0.234416	-0.643568	-3.355657
H	3.116156	0.410573	1.193623
H	3.116156	0.410573	-1.193623
H	-0.504775	-1.053208	4.316139
H	1.909243	-0.827899	3.306530
H	1.909243	-0.827899	-3.306530
H	-0.504775	-1.053208	-4.316139
O	-0.681883	1.060948	-0.000000
S	-1.476485	-0.041757	-2.311796
S	-1.476485	-0.041757	2.311796

T1
E = -1256.06982387 A.U.
G = -1255.978105 A.U.

C	0.376315	-0.000149	1.193765
C	-0.997385	-0.000007	1.546875
C	-2.109809	-0.000154	0.716321

C	-2.109809	-0.000154	-0.716321
C	-0.997385	-0.000007	-1.546875
C	0.376315	-0.000149	-1.193765
C	0.086544	0.000209	3.639555
C	-1.094165	0.000192	2.996024
C	-1.094165	0.000192	-2.996024
C	0.086544	0.000209	-3.639555
H	-3.075497	-0.000185	1.207616
H	-3.075497	-0.000185	-1.207616
H	0.262536	0.000346	4.704226
H	-2.049997	0.000314	3.503321
H	-2.049997	0.000314	-3.503321
H	0.262536	0.000346	-4.704226
O	0.984693	-0.000508	-0.000000
S	1.459699	0.000063	-2.559414
S	1.459699	0.000063	2.559414

12 (X = S X' = NH)

S0

E = -893.470513686 A.U.

G = -893.343832 A.U.

C	-0.538988	0.184740	1.322518
C	0.824697	0.119650	1.555188
C	1.898316	0.566405	0.673385
C	1.898316	0.566405	-0.673385
C	0.824697	0.119650	-1.555188
C	-0.538988	0.184740	-1.322518
C	-0.272743	-0.863081	3.275693
C	0.979441	-0.547324	2.810402
C	0.979441	-0.547324	-2.810402
C	-0.272743	-0.863081	-3.275693
H	2.819901	0.853955	1.171494
H	2.819901	0.853955	-1.171494
H	-0.589179	-1.364342	4.175421
H	1.914859	-0.782301	3.294655
H	1.914859	-0.782301	-3.294655
H	-0.589179	-1.364342	-4.175421
N	-1.188517	-0.419799	2.360099
H	-2.190779	-0.491618	2.448942
N	-1.188517	-0.419799	-2.360099
H	-2.190779	-0.491618	-2.448942
S	-1.372440	0.995070	0.000000

T1

E = -893.381778372 A.U.

G = -893.261765 A.U.

C	-0.541962	-0.062804	1.370668
C	0.868888	0.016897	1.611360
C	1.924914	0.155849	0.724294
C	1.924914	0.155849	-0.724294
C	0.868888	0.016897	-1.611360
C	-0.541962	-0.062804	-1.370668
C	-0.237157	-0.223667	3.594072
C	1.001674	-0.101196	3.054375
C	1.001674	-0.101196	-3.054375
C	-0.237157	-0.223667	-3.594072
H	2.904969	0.229569	1.183525

H	2.904969	0.229569	-1.183525
H	-0.545612	-0.354346	4.618974
H	1.936887	-0.102029	3.592343
H	1.936887	-0.102029	-3.592343
H	-0.545612	-0.354346	-4.618974
N	-1.176625	-0.185178	2.587866
H	-2.164884	-0.339877	2.711699
N	-1.176625	-0.185178	-2.587866
H	-2.164884	-0.339877	-2.711699
S	-1.499141	0.394056	-0.000000

12 (X = S X' = O)

S0

E = -933.168382120 A.U.

G = -933.067023 A.U.

C	-0.521911	0.189699	1.303919
C	0.821997	0.116706	1.532949
C	1.902040	0.586231	0.672283
C	1.902040	0.586231	-0.672283
C	0.821997	0.116706	-1.532949
C	-0.521911	0.189699	-1.303919
C	-0.312321	-0.862902	3.195827
C	0.945507	-0.576353	2.790184
C	0.945507	-0.576353	-2.790184
C	-0.312321	-0.862902	-3.195827
H	2.810110	0.894658	1.180071
H	2.810110	0.894658	-1.180071
H	-0.718408	-1.361271	4.059510
H	1.860616	-0.830405	3.300956
H	1.860616	-0.830405	-3.300956
H	-0.718408	-1.361271	-4.059510
S	-1.398047	0.976186	0.000000
O	-1.222478	-0.404093	-2.294710
O	-1.222478	-0.404093	2.294710

T1

E = -933.090031389 A.U.

G = -932.994872 A.U.

C	-0.519681	-0.034703	1.352923
C	0.877113	0.003539	1.584535
C	1.957319	0.043731	0.717773
C	1.957319	0.043731	-0.717773
C	0.877113	0.003539	-1.584535
C	-0.519681	-0.034703	-1.352923
C	-0.275031	-0.057013	3.530511
C	0.971145	-0.021769	3.038810
C	0.971145	-0.021769	-3.038810
C	-0.275031	-0.057013	-3.530511
H	2.931975	0.071210	1.190716
H	2.931975	0.071210	-1.190716
H	-0.671391	-0.087463	4.531665
H	1.888099	-0.013841	3.606328
H	1.888099	-0.013841	-3.606328
H	-0.671391	-0.087463	-4.531665
S	-1.565898	0.116716	0.000000
O	-1.210835	-0.063293	-2.532556
O	-1.210835	-0.063293	2.532556

---

12 (X = S X' = S)
S0
E = -1579.12853673 A.U.
G = -1579.035604 A.U.

C	-0.335332	0.339846	1.326530
C	1.003645	0.131348	1.556720
C	2.098264	0.532056	0.672380
C	2.098264	0.532056	-0.672380
C	1.003645	0.131348	-1.556720
C	-0.335332	0.339846	-1.326530
C	0.057254	-0.911837	3.416020
C	1.213634	-0.586848	2.780235
C	1.213634	-0.586848	-2.780235
C	0.057254	-0.911837	-3.416020
H	3.034934	0.761970	1.171323
H	3.034934	0.761970	-1.171323
H	-0.067971	-1.443600	4.346721
H	2.194944	-0.851676	3.151189
H	2.194944	-0.851676	-3.151189
H	-0.067971	-1.443600	-4.346721
S	-1.014313	1.281093	0.000000
S	-1.329512	-0.358927	-2.554989
S	-1.329512	-0.358927	2.554989

T1
E = -1579.04982675 A.U.
G = -1578.961633 A.U.

C	0.370067	0.000103	-1.390959
C	-1.039002	0.024623	-1.623664
C	-2.081728	0.122181	-0.714179
C	-2.081728	0.122181	0.714179
C	-1.039002	0.024623	1.623664
C	0.370067	0.000103	1.390959
C	-0.187115	-0.175768	-3.808767
C	-1.292377	-0.081582	-3.050446
C	-1.292377	-0.081582	3.050446
C	-0.187115	-0.175768	3.808767
H	-3.067905	0.166870	-1.163718
H	-3.067905	0.166870	1.163718
H	-0.121290	-0.278589	-4.881388
H	-2.296484	-0.091376	-3.454115
H	-2.296484	-0.091376	3.454115
H	-0.121290	-0.278589	4.881388
S	1.286998	0.436359	0.000000
S	1.285664	-0.164070	2.872744
S	1.285664	-0.164070	-2.872744

---

13 (X = S)
S0
E = -630.326305682 A.U.
G = -630.255126 A.U.

C	0.229545	-1.647719	0.673850
C	0.229545	-1.647719	-0.673850
C	-0.356659	-0.634947	-1.542496
C	-0.356659	-0.634947	1.542496

C	-0.356659	0.687284	-1.348620
C	-0.356659	0.687284	1.348620
H	0.593994	-2.540368	1.173340
H	0.593994	-2.540368	-1.173340
H	-0.853245	-1.004688	-2.436585
H	-0.853245	-1.004688	2.436585
H	-0.872992	1.356245	-2.027003
H	-0.872992	1.356245	2.027003
S	0.504361	1.470138	0.000000

T1
E = -630.274479049 A.U.
G = -630.206588 A.U.

C	0.000006	-1.759184	0.715883
C	0.000006	-1.759184	-0.715883
C	0.000014	-0.705410	-1.611453
C	0.000014	-0.705410	1.611453
C	0.000014	0.676138	-1.416318
C	0.000014	0.676138	1.416318
H	0.000005	-2.745255	1.166995
H	0.000005	-2.745255	-1.166995
H	0.000024	-0.986188	-2.660693
H	0.000024	-0.986188	2.660693
H	0.000021	1.321013	-2.286507
H	0.000021	1.321013	2.286507
S	-0.000032	1.642646	-0.000000

---

13 (X = NH)
S0
E = -287.484190291 A.U.
G = -287.395646 A.U.

C	0.378641	1.357677	0.679287
C	0.378641	1.357677	-0.679287
C	0.378641	0.166272	-1.503233
C	0.378641	0.166272	1.503233
C	-0.212992	-0.986957	-1.147858
C	-0.212992	-0.986957	1.147858
H	0.520814	2.307532	1.184584
H	0.520814	2.307532	-1.184584
H	0.906622	0.210454	-2.450563
H	0.906622	0.210454	2.450563
H	-0.098490	-1.894395	-1.731502
H	-0.098490	-1.894395	1.731502
N	-1.076368	-1.066435	0.000000
H	-1.654807	-0.226038	0.000000

T1
E = -287.455148368 A.U.
G = -287.369154 A.U.

C	-0.000015	-1.487151	0.713550
C	-0.000015	-1.487151	-0.713550
C	-0.000013	-0.383344	-1.557283
C	-0.000013	-0.383344	1.557283
C	-0.000013	0.977409	-1.244498
C	-0.000013	0.977409	1.244498
H	-0.000019	-2.459350	1.193457
H	-0.000019	-2.459350	-1.193457

H	-0.000033	-0.583774	-2.623084
H	-0.000033	-0.583774	2.623084
H	-0.000026	1.703052	-2.045297
H	-0.000026	1.703052	2.045297
N	0.000083	1.548415	-0.000000
H	0.000075	2.558275	-0.000000

13 (X = O)

S0

E = -307.349873440 A.U.

G = -307.274716 A.U.

C	0.306389	-1.397950	0.673754
C	0.306389	-1.397950	-0.673754
C	-0.231285	-0.322464	-1.496857
C	-0.231285	-0.322464	1.496857
C	-0.231285	0.963818	-1.144409
C	-0.231285	0.963818	1.144409
H	0.643522	-2.290000	1.191091
H	0.643522	-2.290000	-1.191091
H	-0.675173	-0.578295	-2.452541
H	-0.675173	-0.578295	2.452541
H	-0.681441	1.751022	-1.739063
H	-0.681441	1.751022	1.739063
O	0.412546	1.414212	-0.000000

T1

E = -307.297297454 A.U.

G = -307.225084 A.U.

C	-0.000001	-1.477908	0.718840
C	-0.000001	-1.477908	-0.718840
C	0.000012	-0.363548	-1.532860
C	0.000012	-0.363548	1.532860
C	0.000012	0.991280	-1.210788
C	0.000012	0.991280	1.210788
H	-0.000005	-2.444336	1.207928
H	-0.000005	-2.444336	-1.207928
H	0.000032	-0.533367	-2.604337
H	0.000032	-0.533367	2.604337
H	0.000023	1.743753	-1.985244
H	0.000023	1.743753	1.985244
O	-0.000048	1.583753	-0.000000

14 (X = NH)

S0

E = -441.126107592 A.U.

G = -440.993976 A.U.

C	2.052472	-1.227108	0.241728
C	2.640106	-0.053704	0.502115
C	2.185634	1.233171	-0.017246
C	0.928372	1.562687	-0.345090
C	-0.281272	0.740130	-0.159088
C	-0.284054	-0.665228	-0.220667
N	0.895665	-1.390934	-0.541255
C	-1.495417	1.393236	0.071670
C	-2.686364	0.696346	0.245803
C	-2.673272	-0.690929	0.189133

C	-1.474928	-1.363843	-0.030407
H	2.493126	-2.150088	0.604803
H	3.551278	-0.069292	1.087469
H	2.949761	1.996919	-0.128699
H	0.743334	2.571383	-0.702685
H	0.686307	-2.368279	-0.692232
H	-1.497139	2.477596	0.105021
H	-3.611705	1.233040	0.413631
H	-3.589437	-1.255813	0.311986
H	-1.462837	-2.447471	-0.078220

T1
E = -441.073218496 A.U.
G = -440.944038 A.U.

C	2.171341	-1.238525	0.000024
C	2.809930	0.010309	0.000063
C	2.245674	1.266199	0.000025
C	0.855046	1.590429	-0.000046
C	-0.274163	0.754878	-0.000033
C	-0.291296	-0.686574	-0.000014
N	0.831450	-1.492114	-0.000095
C	-1.559134	1.393641	-0.000034
C	-2.741499	0.709092	0.000009
C	-2.729207	-0.705945	0.000054
C	-1.525413	-1.369146	0.000038
H	2.770426	-2.137269	0.000007
H	3.892593	-0.049818	0.000132
H	2.926027	2.109574	0.000057
H	0.628509	2.651675	-0.000088
H	0.610467	-2.479359	-0.000092
H	-1.566845	2.478351	-0.000063
H	-3.681949	1.246177	0.000012
H	-3.655413	-1.266132	0.000102
H	-1.511634	-2.454550	0.000066

---

14 (X = O)
S0
E = -460.984875767 A.U.
G = -460.865690 A.U.

C	1.905169	-1.271537	0.270493
C	2.555804	-0.143552	0.548852
C	2.207406	1.163227	-0.000260
C	0.967705	1.554502	-0.336586
C	-0.255439	0.760223	-0.177557
C	-0.269282	-0.637921	-0.264911
C	-1.473422	1.403700	0.079496
C	-2.645307	0.685488	0.273245
C	-2.625072	-0.705188	0.203961
C	-1.432035	-1.367594	-0.062043
H	2.166534	-2.234951	0.693214
H	3.403268	-0.208886	1.221512
H	3.011712	1.887193	-0.077472
H	0.821966	2.577460	-0.669736
H	-1.485884	2.486852	0.133246
H	-3.572842	1.208052	0.471373
H	-3.536049	-1.273704	0.345041
H	-1.389111	-2.446387	-0.147336

O	0.870906	-1.330464	-0.647247

T1
E = -460.916680127 A.U.
G = -460.801451 A.U.

C	-2.103219	-1.262070	0.000034
C	-2.774125	-0.030013	0.000308
C	-2.260518	1.237808	0.000059
C	-0.865864	1.593840	-0.000173
C	0.258264	0.758969	-0.000116
C	0.284265	-0.673370	-0.000059
C	1.549434	1.391625	-0.000079
C	2.725980	0.696899	0.000061
C	2.707659	-0.715616	0.000154
C	1.496415	-1.372557	0.000068
H	-2.654446	-2.190680	0.000017
H	-3.853196	-0.138965	0.000602
H	-2.963693	2.061319	0.000140
H	-0.651055	2.656539	-0.000290
H	1.562635	2.475927	-0.000144
H	3.669356	1.228700	0.000113
H	3.631159	-1.279970	0.000279
H	1.441969	-2.454436	0.000091
O	-0.786559	-1.513941	-0.000294

---

14 (X = S)
S0
E = -783.958839900 A.U.
G = -783.843517 A.U.

C	2.189709	-0.790872	0.641004
C	2.463783	0.511228	0.758209
C	1.903139	1.591937	-0.044192
C	0.642982	1.703493	-0.498445
C	-0.495071	0.808545	-0.245343
C	-0.408474	-0.591453	-0.171321
C	-1.750553	1.399017	-0.037885
C	-2.870693	0.636518	0.258658
C	-2.760902	-0.747737	0.354838
C	-1.530237	-1.357126	0.143513
H	2.650182	-1.524688	1.291529
H	3.190183	0.802403	1.512672
H	2.566257	2.434239	-0.217344
H	0.383404	2.626979	-1.008450
H	-1.834901	2.478090	-0.105529
H	-3.827039	1.120541	0.414090
H	-3.629896	-1.352802	0.582361
H	-1.434624	-2.434458	0.201492
S	1.102146	-1.445725	-0.601565

T1
E = -783.889142603 A.U.
G = -783.778789 A.U.

C	-2.457607	-0.805543	0.000410
C	-2.793578	0.561652	0.001594
C	-1.991554	1.671237	0.000898
C	-0.563087	1.794659	-0.000911
C	0.491944	0.866189	-0.000725

C	0.430393	-0.567351	0.000282
C	1.815685	1.428704	-0.001135
C	2.953898	0.674149	-0.000129
C	2.860515	-0.734836	0.001359
C	1.617887	-1.325653	0.001456
H	-3.255611	-1.537627	0.000117
H	-3.863738	0.744143	0.003043
H	-2.512917	2.621887	0.001785
H	-0.224918	2.825546	-0.002029
H	1.890598	2.510346	-0.002109
H	3.923766	1.156104	-0.000395
H	3.751693	-1.348896	0.002365
H	1.542901	-2.408046	0.002429
S	-0.964922	-1.621418	-0.001487

15 (X = NH)
S0
E = -441.081025684 A.U.
G = -440.951163 A.U.

C	2.812155	0.011851	-0.085269
C	2.166338	1.282614	-0.009632
C	0.859507	1.608492	0.065026
C	-0.345668	0.736101	0.027836
C	-0.315736	-0.686981	0.030890
C	0.890388	-1.524371	0.069348
N	2.161315	-1.162878	-0.015493
C	-1.576410	1.364054	-0.008456
C	-2.794625	0.653205	-0.037619
C	-2.763724	-0.718806	-0.031720
C	-1.519315	-1.376559	0.000837
H	3.884846	-0.065085	-0.139791
H	2.863058	2.115776	0.000442
H	0.627376	2.665947	0.132694
H	0.740586	-2.592661	0.118777
H	-1.601703	2.448709	-0.007983
H	-3.733733	1.192264	-0.060144
H	-3.677582	-1.299934	-0.049357
H	-1.498839	-2.461209	0.007137
H	2.789338	-1.961257	-0.020775

T1
E = -441.078858808 A.U.
G = -440.949309 A.U.

C	-2.565054	-1.110395	0.000000
C	-1.526897	-2.016125	0.000000
C	-0.147403	-1.796121	0.000000
C	0.587285	-0.564757	-0.000000
C	-0.000000	0.753027	-0.000000
C	-1.372912	1.074761	0.000000
N	-2.472248	0.257415	0.000000
C	1.995116	-0.657744	-0.000000
C	2.824190	0.446759	-0.000000
C	2.252874	1.732381	-0.000000
C	0.885206	1.870139	-0.000000
H	-3.585195	-1.467790	0.000000
H	-1.844653	-3.052480	0.000000
H	0.468081	-2.689550	0.000000

H	-1.636993	2.123656	0.000000
H	-3.355924	0.745036	0.000000
H	2.432629	-1.650933	-0.000000
H	3.899882	0.322029	-0.000000
H	2.884110	2.612884	-0.000000
H	0.449376	2.863701	-0.000000

---

15 (X = O)

S0

E = -460.986109739 A.U.

G = -460.865516 A.U.

C	2.335427	-0.285486	-0.285310
C	2.082858	1.170516	-0.275524
C	0.853354	1.651267	-0.050946
C	-0.323788	0.787070	0.037670
C	-0.190663	-0.604343	-0.132369
C	1.162722	-1.199000	-0.208320
C	-1.604851	1.333923	0.169393
C	-2.730781	0.523074	0.119768
C	-2.593844	-0.849094	-0.082895
C	-1.326415	-1.406262	-0.212080
H	3.242342	-0.641292	-0.760725
H	2.918859	1.837614	-0.448235
H	0.684968	2.722234	-0.005743
H	1.238597	-2.194471	-0.634873
H	-1.710665	2.405641	0.298666
H	-3.716188	0.960115	0.226841
H	-3.471302	-1.482273	-0.131375
H	-1.213337	-2.474792	-0.360469
O	2.005327	-0.982846	0.917448

T1

E = -460.923306101 A.U.

G = -460.807545 A.U.

C	-2.569313	-1.031613	-0.000000
C	-1.561481	-1.960281	-0.000000
C	-0.177855	-1.800589	-0.000000
C	0.582365	-0.579710	0.000000
C	-0.000000	0.732549	0.000000
C	-1.369349	1.073785	-0.000000
C	1.982195	-0.685052	0.000000
C	2.817081	0.421412	0.000000
C	2.252377	1.702747	0.000000
C	0.881292	1.847979	0.000000
H	-3.606952	-1.332211	-0.000000
H	-1.929752	-2.980249	-0.000000
H	0.408661	-2.712248	0.000000
H	-1.645427	2.118877	-0.000000
H	2.414758	-1.679988	0.000000
H	3.892094	0.292839	0.000000
H	2.885786	2.581413	0.000000
H	0.450015	2.843031	0.000000
O	-2.486631	0.317648	-0.000000

---

15 (X = S)

S0

E = -783.921616691 A.U.
G = -783.807260 A.U.

C	2.521405	0.418375	0.541414
C	1.720221	1.486573	0.661081
C	0.443522	1.685494	0.022288
C	-0.546076	0.756940	-0.172201
C	-0.440199	-0.669892	0.164776
C	0.696624	-1.407675	0.083296
C	-1.872272	1.241517	-0.527122
C	-2.978487	0.503606	-0.302233
C	-2.876755	-0.807563	0.300092
C	-1.669925	-1.353586	0.548802
H	3.400806	0.285349	1.160141
H	2.039555	2.278391	1.335328
H	0.157665	2.721344	-0.140408
H	0.700698	-2.430293	0.445578
H	-1.949663	2.251347	-0.914815
H	-3.957928	0.902943	-0.535764
H	-3.782259	-1.354932	0.531934
H	-1.583560	-2.354033	0.957443
S	2.186646	-0.838928	-0.672537

T1
E = -783.897084323 A.U.
G = -783.786078 A.U.

C	-2.641731	0.645529	0.000441
C	-1.765955	1.705133	0.000439
C	-0.374803	1.773736	-0.000021
C	0.669295	0.779188	-0.000169
C	0.514167	-0.649985	0.000159
C	-0.685632	-1.401511	0.000221
C	1.983198	1.294483	-0.000472
C	3.111621	0.497297	-0.000337
C	2.961367	-0.895686	0.000164
C	1.698020	-1.441544	0.000402
H	-3.706862	0.843045	0.000766
H	-2.261256	2.671669	0.000806
H	0.009337	2.788211	-0.000153
H	-0.573295	-2.479805	0.000348
H	2.100792	2.372627	-0.000784
H	4.096357	0.947314	-0.000578
H	3.829302	-1.543673	0.000352
H	1.586640	-2.520057	0.000757
S	-2.368644	-1.057448	-0.000404

---

16 (X = NH)
S0
E = -441.128331327 A.U.
G = -440.997205 A.U.

C	0.005201	-2.169861	1.234909
C	-0.114782	-0.872850	1.547371
C	-0.050252	0.342162	0.707607
C	-0.050252	0.342162	-0.707607
C	-0.114782	-0.872850	-1.547371
C	0.005201	-2.169861	-1.234909
C	0.003879	1.563909	1.373186
C	0.062324	2.782793	0.690970

C	0.062324	2.782793	-0.690970
C	0.003879	1.563909	-1.373186
H	-0.029617	-2.891702	2.042493
H	-0.240541	-0.676926	2.606146
H	-0.240541	-0.676926	-2.606146
H	-0.029617	-2.891702	-2.042493
H	-0.004171	1.563568	2.458102
H	0.102286	3.712060	1.246281
H	0.102286	3.712060	-1.246281
H	-0.004171	1.563568	-2.458102
N	0.182396	-2.770202	0.000000
H	0.190887	-3.776441	0.000000

T1
E = -441.069105187 A.U.
G = -440.939465 A.U.

C	-0.000096	-2.192720	1.234570
C	0.000300	-0.833991	1.543880
C	0.000151	0.311987	0.731821
C	0.000151	0.311987	-0.731821
C	0.000300	-0.833991	-1.543880
C	-0.000096	-2.192720	-1.234570
C	-0.000018	1.584231	1.392013
C	-0.000153	2.767807	0.716881
C	-0.000153	2.767807	-0.716881
C	-0.000018	1.584231	-1.392013
H	-0.000091	-2.912536	2.039994
H	0.000450	-0.643257	2.612312
H	0.000450	-0.643257	-2.612312
H	-0.000091	-2.912536	-2.039994
H	0.000006	1.583657	2.477487
H	-0.000270	3.706507	1.257472
H	-0.000270	3.706507	-1.257472
H	0.000006	1.583657	-2.477487
N	-0.000243	-2.763183	-0.000000
H	-0.000704	-3.774241	-0.000000

---

16 (X = O)
S0
E = -460.982654624 A.U.
G = -460.864834 A.U.

C	2.179473	-1.203414	0.000952
C	0.886620	-1.519659	-0.054068
C	-0.340415	-0.704907	-0.023245
C	-0.340415	0.704907	-0.023244
C	0.886619	1.519659	-0.054064
C	2.179473	1.203414	0.000957
C	-1.559002	-1.377763	0.001200
C	-2.774906	-0.692193	0.029533
C	-2.774906	0.692193	0.029533
C	-1.559002	1.377763	0.001200
H	2.923555	-1.989365	-0.019044
H	0.716717	-2.589013	-0.120027
H	0.716717	2.589013	-0.120023
H	2.923555	1.989366	-0.019038
H	-1.557137	-2.462406	-0.003327
H	-3.706262	-1.244746	0.048632

H	-3.706262	1.244746	0.048632
H	-1.557137	2.462406	-0.003326
O	2.818128	-0.000000	0.091875

T1
E = -460.907420890 A.U.
G = -460.791829 A.U.

C	-0.000286	-2.198367	1.202212
C	0.000030	-0.848425	1.521870
C	0.000045	0.304222	0.734650
C	0.000045	0.304222	-0.734650
C	0.000030	-0.848425	-1.521870
C	-0.000286	-2.198367	-1.202212
C	0.000033	1.576377	1.400625
C	0.000002	2.752259	0.721372
C	0.000002	2.752259	-0.721372
C	0.000033	1.576377	-1.400625
H	-0.000512	-2.945452	1.981810
H	-0.000106	-0.686692	2.595452
H	-0.000106	-0.686692	-2.595452
H	-0.000512	-2.945452	-1.981810
H	0.000042	1.575047	2.485648
H	-0.000039	3.693752	1.256494
H	-0.000039	3.693752	-1.256494
H	0.000042	1.575047	-2.485648
O	0.000419	-2.788264	-0.000000

16 (X = S)
S0
E = -783.956165975 A.U.
G = -783.841271 A.U.

C	1.863058	-1.359303	-0.261791
C	0.586323	-1.543169	-0.593397
C	-0.574328	-0.704981	-0.245664
C	-0.574328	0.704981	-0.245664
C	0.586323	1.543169	-0.593398
C	1.863058	1.359303	-0.261792
C	-1.776003	-1.379484	0.020329
C	-2.941463	-0.697120	0.325588
C	-2.941463	0.697120	0.325588
C	-1.776002	1.379484	0.020330
H	2.623135	-2.068323	-0.567819
H	0.353578	-2.441218	-1.160730
H	0.353578	2.441217	-1.160732
H	2.623136	2.068322	-0.567822
H	-1.779534	-2.463597	-0.013099
H	-3.849340	-1.245464	0.545152
H	-3.849339	1.245465	0.545154
H	-1.779533	2.463597	-0.013097
S	2.463329	0.000000	0.715764

T1
E = -783.883916223 A.U.
G = -783.772304 A.U.

C	1.867985	-1.397943	0.000086
C	0.490795	-1.591164	0.000251
C	-0.619884	-0.732971	0.000073

C	-0.619884	0.732971	0.000073
C	0.490795	1.591164	0.000252
C	1.867985	1.397943	0.000086
C	-1.899676	-1.387985	-0.000047
C	-3.081824	-0.716311	-0.000204
C	-3.081824	0.716311	-0.000205
C	-1.899676	1.387985	-0.000048
H	2.502941	-2.275049	0.000064
H	0.228790	-2.645739	0.000428
H	0.228790	2.645739	0.000429
H	2.502941	2.275049	0.000065
H	-1.901978	-2.472753	-0.000044
H	-4.019149	-1.258706	-0.000322
H	-4.019149	1.258706	-0.000323
H	-1.901978	2.472753	-0.000046
S	2.830627	0.000000	-0.000135

---

17 (X = NH)

S0

E = -594.752969441 A.U.

G = -594.576672 A.U.

C	-3.191157	-1.353335	-0.499992
C	-3.578220	-0.059604	-0.845974
C	-2.766181	1.016749	-0.519432
C	-1.545571	0.824380	0.142716
C	-1.188443	-0.480107	0.524489
C	-2.003408	-1.557578	0.191979
C	-0.672692	1.976580	0.394296
C	0.672703	1.976584	0.394302
C	1.545581	0.824386	0.142720
C	1.188439	-0.480110	0.524483
N	-0.000000	-0.714032	1.302362
C	2.766193	1.016745	-0.519422
C	3.578219	-0.059613	-0.845969
C	3.191147	-1.353344	-0.499995
C	2.003394	-1.557586	0.191963
H	-3.823572	-2.196590	-0.749850
H	-4.512023	0.107423	-1.368938
H	-3.061492	2.023385	-0.795219
H	-1.699071	-2.549325	0.504695
H	-1.175459	2.934879	0.486272
H	1.175462	2.934886	0.486280
H	0.000002	-0.046176	2.072557
H	3.061507	2.023384	-0.795196
H	4.512027	0.107384	-1.368934
H	3.823573	-2.196587	-0.749872
H	1.699027	-2.549323	0.504686

T1

E = -594.688934136 A.U.

G = -594.516546 A.U.

C	3.637911	-1.200349	0.000206
C	3.993906	0.174403	0.000108
C	3.020568	1.127640	-0.000031
C	1.614471	0.817529	-0.000090
C	1.279380	-0.586577	-0.000055
C	2.310729	-1.550852	0.000118

C	0.713075	1.877229	-0.000150
C	-0.713075	1.877229	-0.000050
C	-1.614471	0.817529	-0.000000
C	-1.279380	-0.586577	-0.000100
N	0.000000	-1.096228	-0.000303
C	-3.020568	1.127640	0.000151
C	-3.993905	0.174403	0.000176
C	-3.637911	-1.200349	0.000066
C	-2.310730	-1.550852	-0.000069
H	4.401408	-1.967163	0.000343
H	5.037802	0.463416	0.000152
H	3.290360	2.178080	-0.000085
H	2.032917	-2.600166	0.000199
H	1.165608	2.863302	-0.000213
H	-1.165608	2.863302	0.000010
H	0.000000	-2.109614	-0.000428
H	-3.290360	2.178080	0.000241
H	-5.037802	0.463416	0.000285
H	-4.401408	-1.967163	0.000086
H	-2.032917	-2.600166	-0.000133

---

17 (X = O)

S0

E = -614.620180764 A.U.

G = -614.457079 A.U.

C	-3.225219	-1.344455	-0.431825
C	-3.628418	-0.049774	-0.748725
C	-2.794722	1.023263	-0.464497
C	-1.539995	0.834013	0.128789
C	-1.169694	-0.475494	0.455908
C	-1.990877	-1.557071	0.171985
C	-0.670482	1.987035	0.384785
C	0.670483	1.987035	0.384784
C	1.539995	0.834014	0.128789
C	1.169694	-0.475493	0.455907
C	2.794724	1.023262	-0.464496
C	3.628418	-0.049775	-0.748724
C	3.225218	-1.344456	-0.431825
C	1.990876	-1.557072	0.171983
H	-3.872362	-2.186523	-0.644136
H	-4.592647	0.123021	-1.210255
H	-3.106861	2.032827	-0.708425
H	-1.659203	-2.549380	0.452211
H	-1.179670	2.938918	0.500209
H	1.179670	2.938918	0.500206
H	3.106863	2.032826	-0.708424
H	4.592648	0.123018	-1.210251
H	3.872362	-2.186523	-0.644138
H	1.659196	-2.549379	0.452208
O	0.000000	-0.715989	1.157972

T1

E = -614.534113240 A.U.

G = -614.376177 A.U.

C	3.577320	-1.235653	0.000192
C	3.964042	0.128243	0.000361
C	3.014163	1.104887	0.000243

C	1.598369	0.825081	-0.000052
C	1.247934	-0.568530	-0.000169
C	2.237838	-1.557925	-0.000075
C	0.720802	1.897839	-0.000272
C	-0.720802	1.897839	-0.000276
C	-1.598369	0.825081	-0.000055
C	-1.247934	-0.568530	-0.000168
C	-3.014163	1.104887	0.000238
C	-3.964042	0.128243	0.000359
C	-3.577320	-1.235653	0.000197
C	-2.237838	-1.557925	-0.000069
H	4.323838	-2.019040	0.000259
H	5.014142	0.393077	0.000597
H	3.305852	2.149160	0.000384
H	1.906288	-2.589089	-0.000210
H	1.180185	2.879805	-0.000298
H	-1.180185	2.879805	-0.000307
H	-3.305852	2.149160	0.000374
H	-5.014142	0.393077	0.000593
H	-4.323838	-2.019040	0.000267
H	-1.906288	-2.589089	-0.000201
O	0.000000	-1.094391	-0.000523

---

17 (X = S)
S0
E = -937.591420708 A.U.
G = -937.432033 A.U.

C	-3.425130	-1.020406	-0.854450
C	-3.666473	0.344905	-0.979892
C	-2.759584	1.257499	-0.461484
C	-1.589921	0.838284	0.188762
C	-1.371275	-0.542150	0.323231
C	-2.278117	-1.460069	-0.205477
C	-0.670860	1.873959	0.681240
C	0.670854	1.873959	0.681231
C	1.589916	0.838283	0.188755
C	1.371275	-0.542145	0.323233
C	2.759580	1.257502	-0.461495
C	3.666471	0.344911	-0.979893
C	3.425134	-1.020404	-0.854440
C	2.278126	-1.460067	-0.205469
H	-4.128562	-1.740755	-1.253581
H	-4.560811	0.697231	-1.478999
H	-2.947410	2.321004	-0.560320
H	-2.083652	-2.519654	-0.093087
H	-1.157203	2.796307	0.986170
H	1.157201	2.796307	0.986156
H	2.947397	2.321008	-0.560337
H	4.560809	0.697234	-1.479003
H	4.128571	-1.740750	-1.253570
H	2.083663	-2.519651	-0.093069
S	0.000001	-1.163290	1.281033

T1
E = -937.501070115 A.U.
G = -937.347035 A.U.

C	-3.847311	-0.958988	-0.261109

C	-4.092207	0.437376	-0.217015
C	-3.055369	1.306476	-0.066886
C	-1.680505	0.875872	0.047830
C	-1.467559	-0.549430	0.027097
C	-2.557781	-1.424316	-0.128900
C	-0.720287	1.871418	0.157830
C	0.720285	1.871418	0.157822
C	1.680504	0.875872	0.047824
C	1.467560	-0.549430	0.027102
C	3.055366	1.306477	-0.066900
C	4.092206	0.437377	-0.217021
C	3.847313	-0.958987	-0.261098
C	2.557784	-1.424316	-0.128885
H	-4.663058	-1.656394	-0.399918
H	-5.104560	0.811673	-0.307369
H	-3.241974	2.374178	-0.040261
H	-2.367748	-2.491730	-0.150858
H	-1.133286	2.873944	0.201492
H	1.133284	2.873945	0.201475
H	3.241969	2.374180	-0.040286
H	5.104559	0.811676	-0.307380
H	4.663061	-1.656393	-0.399900
H	2.367752	-2.491730	-0.150833
S	-0.000000	-1.407766	0.417981

18 (X = NH)

S0

E = -594.757530237 A.U.

G = -594.580940 A.U.

C	3.520633	-0.720125	-0.079089
C	2.721212	-1.770077	-0.515227
C	1.339973	-1.625375	-0.496443
C	0.718717	-0.454151	-0.045206
C	1.542222	0.617350	0.336564
C	2.931153	0.469273	0.326270
C	-0.769396	-0.430508	0.059440
C	-1.554772	0.677913	-0.312469
C	-1.001152	1.999010	-0.636782
C	0.100569	2.528055	-0.096052
N	0.995960	1.855998	0.749208
C	-1.417103	-1.601134	0.474750
C	-2.801469	-1.708142	0.483029
C	-3.574171	-0.632932	0.055928
C	-2.949555	0.542434	-0.334123
H	4.599838	-0.813906	-0.073356
H	3.165053	-2.693945	-0.864209
H	0.717316	-2.443859	-0.838246
H	3.549506	1.303894	0.638690
H	-1.610701	2.647879	-1.254613
H	0.344376	3.569567	-0.278041
H	1.713033	2.474523	1.102218
H	-0.819121	-2.444159	0.800776
H	-3.270713	-2.626625	0.813841
H	-4.654982	-0.705186	0.037804
H	-3.546495	1.390270	-0.652867

T1

E = -594.675977895 A.U.
G = -594.502774 A.U.

C	3.561120	-0.769825	-0.018753
C	2.707432	-1.873083	-0.244460
C	1.350642	-1.698352	-0.252133
C	0.696895	-0.440540	-0.031858
C	1.597603	0.676490	0.088715
C	2.998066	0.468619	0.128064
C	-0.740955	-0.426612	0.062949
C	-1.612008	0.740782	-0.124862
C	-1.207268	2.071686	-0.145919
C	0.053436	2.645654	0.045777
N	1.232248	2.001742	0.186769
C	-1.417450	-1.658438	0.334270
C	-2.774016	-1.805561	0.271496
C	-3.607579	-0.696512	-0.080797
C	-3.029346	0.519434	-0.251268
H	4.636281	-0.890312	0.006368
H	3.122603	-2.855598	-0.432797
H	0.737574	-2.558337	-0.479872
H	3.635175	1.336911	0.264474
H	-1.995199	2.804325	-0.284812
H	0.138883	3.721377	0.091897
H	2.020450	2.621901	0.324486
H	-0.835661	-2.517076	0.640020
H	-3.220165	-2.764612	0.505841
H	-4.679581	-0.820514	-0.167481
H	-3.645533	1.387282	-0.462832

---

18 (X = O)
S0
E = -614.615755372 A.U.
G = -614.452291 A.U.

C	3.519633	-0.644974	-0.098817
C	2.745367	-1.727586	-0.504919
C	1.359943	-1.643908	-0.461989
C	0.707625	-0.487831	-0.012983
C	1.512278	0.590260	0.368324
C	2.898175	0.521308	0.329240
C	-0.775181	-0.446068	0.062227
C	-1.522726	0.694290	-0.295025
C	-0.906831	1.983044	-0.642119
C	0.212719	2.455295	-0.104596
C	-1.462064	-1.609729	0.431964
C	-2.848063	-1.666737	0.427785
C	-3.583728	-0.552674	0.032156
C	-2.920448	0.609968	-0.328186
H	4.600804	-0.702515	-0.124295
H	3.218338	-2.635606	-0.857637
H	0.764266	-2.485117	-0.796292
H	3.467612	1.385545	0.648109
H	-1.439204	2.626883	-1.333160
H	0.618872	3.433629	-0.334302
H	-0.894676	-2.480750	0.738222
H	-3.351311	-2.577798	0.727629
H	-4.666059	-0.589039	0.010601
H	-3.486356	1.483205	-0.633826

O	0.943440	1.761703	0.842072

T1
E = -614.517467042 A.U.
G = -614.358729 A.U.

C	3.557707	-0.730664	-0.079049
C	2.718949	-1.847690	-0.252286
C	1.357691	-1.695794	-0.219762
C	0.696304	-0.442390	-0.009112
C	1.583456	0.667846	0.094204
C	2.978478	0.503085	0.084224
C	-0.742327	-0.433735	0.085040
C	-1.597965	0.746651	-0.147419
C	-1.168204	2.053845	-0.148936
C	0.099566	2.605652	0.093554
C	-1.426212	-1.648085	0.379526
C	-2.783877	-1.780682	0.293013
C	-3.602190	-0.671791	-0.123794
C	-3.020911	0.531193	-0.322421
H	4.634965	-0.832890	-0.096758
H	3.143317	-2.827687	-0.432186
H	0.747701	-2.567261	-0.409085
H	3.575984	1.398845	0.200381
H	-1.925591	2.814468	-0.310303
H	0.229410	3.674189	0.177374
H	-0.855279	-2.504641	0.711329
H	-3.248159	-2.726069	0.544693
H	-4.671077	-0.799120	-0.238933
H	-3.621808	1.395304	-0.584996
O	1.260969	1.978776	0.259724

---

18 (X = S)
S0
E = -937.588526538 A.U.
G = -937.429045 A.U.

C	-3.435919	-1.031761	0.211630
C	-2.569735	-2.054347	0.582731
C	-1.196659	-1.863135	0.503180
C	-0.649746	-0.656832	0.045162
C	-1.538703	0.369364	-0.305600
C	-2.917227	0.182439	-0.220650
C	0.831010	-0.547177	-0.059416
C	1.579892	0.523998	0.467932
C	0.995685	1.747659	1.042978
C	-0.029685	2.439419	0.547076
C	1.519756	-1.631564	-0.622572
C	2.904568	-1.675192	-0.660569
C	3.642679	-0.635270	-0.100538
C	2.979864	0.441107	0.465429
H	-4.508412	-1.171129	0.269329
H	-2.960685	-2.999220	0.939806
H	-0.527050	-2.658767	0.808464
H	-3.578047	0.994990	-0.496634
H	1.501905	2.155292	1.914345
H	-0.358602	3.364506	1.004694
H	0.947558	-2.449435	-1.045017
H	3.405732	-2.520376	-1.116321

H	4.725365	-0.664173	-0.106741
H	3.547411	1.253211	0.906698
S	-0.930616	1.940178	-0.903704

T1
E = -937.487697423 A.U.
G = -937.333812 A.U.

C	3.373805	-1.292191	-0.197420
C	2.401136	-2.280295	-0.401553
C	1.067255	-1.956725	-0.324891
C	0.590616	-0.645263	-0.036535
C	1.598717	0.346269	0.095194
C	2.964161	-0.000149	0.039158
C	-0.852727	-0.490286	0.111518
C	-1.663987	0.671400	-0.286859
C	-1.263496	1.975955	-0.461215
C	-0.035324	2.643512	-0.184167
C	-1.565489	-1.619483	0.585422
C	-2.933224	-1.732969	0.534393
C	-3.709905	-0.692225	-0.062716
C	-3.092230	0.446230	-0.454075
H	4.428863	-1.526845	-0.256423
H	2.693034	-3.295563	-0.640675
H	0.339363	-2.729003	-0.533548
H	3.703002	0.783129	0.165736
H	-2.039710	2.656704	-0.798462
H	-0.031322	3.725928	-0.192085
H	-1.002336	-2.434708	1.022029
H	-3.417955	-2.614631	0.934165
H	-4.781809	-0.803127	-0.169255
H	-3.673346	1.267041	-0.860163
S	1.406648	2.045524	0.474447

---

19 (X = NH)
S0
E = -303.510980116 A.U.
G = -303.434111 A.U.

C	0.160717	-1.484423	-0.277467
C	-0.874336	1.225018	-0.093546
C	0.428927	1.480780	-0.273177
C	1.479431	0.566045	0.146893
C	1.338536	-0.767237	0.227079
H	0.350477	-2.418625	-0.803741
H	-1.625893	1.955469	-0.376418
H	0.713033	2.435069	-0.698969
H	2.445945	1.001490	0.382629
H	2.184044	-1.381855	0.515338
N	-1.066125	-1.153388	-0.225703
N	-1.349420	0.040699	0.515128
H	-2.358440	0.076167	0.576499

T1
E = -303.473186069 A.U.
G = -303.398763 A.U.

C	1.535556	0.260448	-0.000000
C	-1.234512	-0.915530	0.000000
C	-1.545873	0.436160	0.000000

C	-0.664916	1.514188	0.000000
C	0.748329	1.405169	-0.000000
H	2.608892	0.419322	-0.000000
H	-2.031153	-1.646687	0.000000
H	-2.608252	0.650982	0.000000
H	-1.088190	2.510632	0.000000
H	1.307859	2.334259	-0.000000
N	1.256627	-1.056627	-0.000000
N	-0.000000	-1.508004	0.000000
H	-0.017054	-2.518709	0.000000

19 (X = O)

S0

E = -323.353026262 A.U.

G = -323.289663 A.U.

C	-1.473556	-0.024162	0.277112
C	1.240671	0.823200	0.093380
C	1.437116	-0.486566	0.287296
C	0.466853	-1.478947	-0.131116
C	-0.859402	-1.249838	-0.214537
H	-2.410170	-0.114428	0.824877
H	1.945086	1.590189	0.397878
H	2.362262	-0.812844	0.746486
H	0.831499	-2.481795	-0.327298
H	-1.531890	-2.061425	-0.464351
N	-1.028928	1.171254	0.246055
O	0.141951	1.272426	-0.596598

T1

E = -323.290505574 A.U.

G = -323.230836 A.U.

C	0.727453	1.345998	0.000000
C	-0.000000	-1.520102	-0.000000
C	-1.265521	-0.949157	-0.000000
C	-1.611383	0.384199	-0.000000
C	-0.651491	1.442994	-0.000000
H	1.275871	2.282925	0.000000
H	0.116082	-2.594834	-0.000000
H	-2.061981	-1.685237	-0.000000
H	-2.659693	0.649900	-0.000000
H	-1.034396	2.456696	0.000000
N	1.619373	0.342491	0.000000
O	1.229270	-0.966309	0.000000

19 (X = S)

S0

E = -646.355951276 A.U.

G = -646.296649 A.U.

C	0.457952	-1.537834	0.352726
C	-0.580804	1.393253	0.320263
C	0.753910	1.506632	0.316315
C	1.679098	0.552616	-0.272238
C	1.516574	-0.781488	-0.325266
H	0.806386	-2.435323	0.870728
H	-1.202688	2.148353	0.786533
H	1.187849	2.395566	0.764655

H	2.613621	0.962945	-0.642777
H	2.324122	-1.386615	-0.723705
N	-0.790025	-1.317462	0.474894
S	-1.447469	0.046140	-0.420655

T1
E = -646.308978104 A.U.
G = -646.252682 A.U.

C	0.134768	1.700981	0.000000
C	-0.000000	-1.553510	0.000000
C	-1.333753	-1.165310	0.000000
C	-1.880620	0.109824	0.000000
C	-1.212339	1.366639	0.000000
H	0.346960	2.767454	0.000000
H	0.247580	-2.609882	0.000000
H	-2.040363	-1.989564	0.000000
H	-2.962694	0.161994	0.000000
H	-1.868209	2.230888	0.000000
N	1.264517	0.979554	0.000000
S	1.448548	-0.635594	0.000000

---

20 (X = NH)
S0
E = -303.531822359 A.U.
G = -303.455395 A.U.

C	1.077104	1.094280	0.086039
C	-0.186037	1.496079	0.291463
C	-1.368216	0.743609	-0.157092
C	-0.550892	-1.409033	0.232902
C	0.771293	-1.293659	0.114869
H	1.903313	1.772733	0.276520
H	-0.348081	2.508678	0.637476
H	-2.200517	1.362790	-0.498555
H	-0.943834	-2.324871	0.658305
H	1.413279	-2.106073	0.436289
N	-1.547143	-0.516611	-0.233411
N	1.443469	-0.157159	-0.402863
H	2.442035	-0.284520	-0.465203

T1
E = -303.495750062 A.U.
G = -303.421181 A.U.

C	1.133563	1.104899	-0.000023
C	-0.181146	1.546928	-0.000013
C	-1.374851	0.826005	-0.000000
C	-0.638290	-1.425639	-0.000002
C	0.767521	-1.328772	-0.000044
H	1.949776	1.811761	-0.000015
H	-0.289964	2.625884	-0.000017
H	-2.284810	1.417484	0.000005
H	-0.983124	-2.457382	0.000017
H	1.333057	-2.250159	-0.000087
N	-1.604472	-0.522196	0.000022
N	1.533322	-0.209784	0.000047
H	2.532331	-0.364251	0.000101

---

20 (X = O)
S0
E = -323.387768858 A.U.
G = -323.324371 A.U.

C	-1.158959	-0.958411	-0.124441
C	0.054011	-1.485093	-0.286175
C	1.281553	-0.830579	0.175289
C	0.601655	1.353049	-0.259053
C	-0.722339	1.289515	-0.127837
H	-2.073289	-1.449843	-0.439390
H	0.135623	-2.470499	-0.728504
H	2.082250	-1.494660	0.500109
H	1.023018	2.237879	-0.722310
H	-1.387521	2.070766	-0.474771
N	1.542918	0.423192	0.202734
O	-1.364504	0.241640	0.522379

T1
E = -323.335168208 A.U.
G = -323.274509 A.U.

C	-1.532133	-0.320192	0.000000
C	-0.650462	-1.384815	0.000000
C	0.734610	-1.417719	0.000000
C	1.260395	0.877962	0.000000
C	-0.000000	1.516676	0.000000
H	-2.601944	-0.458043	0.000000
H	-1.146672	-2.349359	0.000000
H	1.199969	-2.396256	0.000000
H	2.068433	1.607701	0.000000
H	-0.037892	2.596679	0.000000
N	1.638965	-0.375696	0.000000
O	-1.228639	0.999709	0.000000

---

20 (X = S)
S0
E = -646.363957974 A.U.
G = -646.304623 A.U.

C	-0.660536	-1.336593	0.391283
C	0.663935	-1.489990	0.359708
C	1.599937	-0.601811	-0.343637
C	0.716373	1.461504	0.333187
C	-0.612169	1.335731	0.380071
H	-1.301259	-2.046876	0.901686
H	1.099338	-2.365951	0.832519
H	2.405183	-1.108439	-0.877378
H	1.170168	2.313779	0.831387
H	-1.217028	2.055498	0.916813
N	1.634958	0.676416	-0.366208
S	-1.490397	0.012752	-0.422828

T1
E = -646.311080869 A.U.
G = -646.255038 A.U.

C	-1.204567	-1.000105	0.000000
C	-0.012738	-1.707638	-0.000000
C	1.320809	-1.311050	-0.000000
C	1.343920	1.083311	-0.000000

C	0.000000	1.512441	0.000000
H	-2.142702	-1.539771	0.000000
H	-0.148800	-2.785427	0.000000
H	2.034219	-2.129093	-0.000000
H	2.036637	1.924535	-0.000000
H	-0.164822	2.583323	0.000000
N	1.934556	-0.088691	-0.000000
S	-1.490061	0.694094	0.000000

21 (X = NH)
S0
E = -457.162866115 A.U.
G = -457.042011 A.U.

C	-2.189226	1.111283	0.054579
C	0.244409	-0.703082	-0.170005
C	0.233938	0.713996	-0.174483
C	-0.982895	1.502730	-0.370901
H	-3.089271	1.686371	-0.127444
H	-0.878130	2.470220	-0.851553
C	-2.035895	-1.169389	-0.116355
H	-2.890583	-1.718430	-0.503740
N	-0.870243	-1.491136	-0.501081
C	1.461841	-1.380595	0.006685
H	1.444220	-2.463463	-0.021167
C	2.642399	-0.690973	0.199049
H	3.566989	-1.234114	0.352128
C	1.461484	1.388927	-0.041548
C	2.645298	0.707952	0.163326
H	3.571907	1.254837	0.286484
H	1.458739	2.472794	-0.081750
N	-2.338283	-0.113084	0.804312
H	-1.612306	-0.123763	1.522346

T1
E = -457.112865755 A.U.
G = -456.995249 A.U.

C	-2.205381	1.206433	-0.000105
C	0.275397	-0.729358	-0.000031
C	0.286548	0.739368	-0.000011
C	-0.843731	1.553220	-0.000086
H	-2.954231	1.984476	0.000036
H	-0.664263	2.622735	-0.000202
C	-2.070764	-1.263419	0.000037
H	-2.768574	-2.091589	0.000068
N	-0.791798	-1.552746	0.000003
C	1.539271	-1.395143	-0.000069
H	1.504590	-2.478307	-0.000121
C	2.729377	-0.729228	-0.000021
H	3.662947	-1.278324	-0.000052
C	1.570748	1.390046	0.000072
C	2.743660	0.704448	0.000071
H	3.687936	1.234858	0.000143
H	1.577307	2.475156	0.000128
N	-2.705450	-0.049121	0.000110
H	-3.715725	-0.114135	0.000067

21 (X = O)
S0
E = -477.034493379 A.U.
G = -476.927130 A.U.

C	2.163388	1.130288	-0.062704
C	-0.265088	-0.688861	0.162766
C	-0.270509	0.718485	0.161196
C	0.951635	1.512773	0.322883
H	3.049102	1.744369	0.039469
H	0.856256	2.507133	0.743979
C	2.035187	-1.191267	0.059866
H	2.868833	-1.854563	0.272387
N	0.871449	-1.486620	0.425931
C	-1.467142	-1.383305	-0.000861
H	-1.436183	-2.465578	0.033622
C	-2.662544	-0.707223	-0.189654
H	-3.581372	-1.263411	-0.328893
C	-1.495466	1.383184	0.022878
C	-2.678800	0.686368	-0.167605
H	-3.610716	1.224670	-0.288514
H	-1.503068	2.467232	0.053925
O	2.424129	-0.089520	-0.670009

T1
E = -476.953733928 A.U.
G = -476.850329 A.U.

C	2.208015	1.173346	0.000150
C	-0.268054	-0.729208	-0.000481
C	-0.283954	0.744266	-0.000296
C	0.851629	1.531863	-0.000435
H	2.981018	1.926989	0.000681
H	0.701684	2.606492	-0.000633
C	2.080727	-1.237079	-0.000002
H	2.812089	-2.033009	0.000884
N	0.819628	-1.520812	-0.000081
C	-1.522734	-1.406165	-0.000026
H	-1.486219	-2.488870	-0.000005
C	-2.710876	-0.739365	0.000079
H	-3.644084	-1.288595	0.000073
C	-1.572391	1.397199	0.000102
C	-2.734041	0.702290	0.000317
H	-3.684201	1.221466	0.000702
H	-1.581886	2.481820	0.000276
O	2.734285	-0.050436	0.000268

---

21 (X = S)
S0
E = -800.001637396 A.U.
G = -799.898404 A.U.

C	-1.880324	1.331568	-0.195261
C	0.534150	-0.685138	-0.282496
C	0.544832	0.722834	-0.270767
C	-0.621678	1.560079	-0.571212
H	-2.680217	2.014503	-0.453000
H	-0.422406	2.480609	-1.113820
C	-1.742149	-1.334035	-0.369532
H	-2.500227	-2.004335	-0.768094

N	-0.542915	-1.479672	-0.716717
C	1.711316	-1.388210	0.014113
H	1.680965	-2.469879	-0.036206
C	2.874187	-0.722282	0.351910
H	3.765748	-1.284622	0.600640
C	1.759288	1.375906	0.000848
C	2.903142	0.674448	0.333063
H	3.817767	1.205846	0.564777
H	1.778382	2.459823	-0.032555
S	-2.383512	-0.078454	0.761955

T1
E = -799.922210177 A.U.
G = -799.823128 A.U.

C	-1.889472	1.386406	0.000256
C	0.585090	-0.734293	0.000096
C	0.590625	0.738605	0.000027
C	-0.505079	1.599692	0.000255
H	-2.539848	2.251888	0.000530
H	-0.247277	2.654714	0.000341
C	-1.742069	-1.419236	0.000131
H	-2.361456	-2.311790	-0.000128
N	-0.449605	-1.588929	0.000182
C	1.857837	-1.393906	0.000111
H	1.824299	-2.476477	0.000202
C	3.044178	-0.728348	-0.000061
H	3.978365	-1.276071	-0.000096
C	1.881727	1.385064	-0.000116
C	3.055083	0.705380	-0.000198
H	3.997018	1.239332	-0.000342
H	1.889321	2.469529	-0.000183
S	-2.791294	-0.041550	-0.000288

---

22 (X = NH)
S0
E = -457.177479857 A.U.
G = -457.056657 A.U.

C	-2.197555	1.152027	0.035944
C	0.273888	-0.653494	0.221068
C	0.275810	0.747379	0.137153
C	-0.947222	1.538512	0.319451
H	-3.005990	1.859894	0.188116
H	-0.808782	2.557420	0.667088
N	-2.634435	-0.051530	-0.540157
C	-2.054078	-1.154222	-0.294087
H	-2.481694	-2.063561	-0.714551
N	-0.929611	-1.360298	0.511885
H	-0.752580	-2.347281	0.650329
C	1.458619	-1.366353	0.059477
H	1.439922	-2.448768	0.122815
C	2.661061	-0.698974	-0.152695
H	3.577214	-1.267905	-0.253469
C	1.493419	1.396232	-0.085143
C	2.680899	0.688435	-0.229886
H	3.612072	1.216159	-0.393494
H	1.499114	2.479587	-0.136620

T1
E = -457.115446252 A.U.
G = -456.998239 A.U.

C	-2.255582	1.195887	0.000660
C	0.282511	-0.685943	0.000180
C	0.275034	0.755316	-0.000014
C	-0.863784	1.559783	-0.000141
H	-2.950499	2.030598	0.001451
H	-0.682752	2.629710	-0.000520
N	-2.824721	0.005744	0.000120
C	-2.197887	-1.162042	-0.000689
H	-2.811041	-2.053445	-0.001513
N	-0.859050	-1.453559	-0.000049
H	-0.678596	-2.450187	-0.000494
C	1.509230	-1.379831	0.000456
H	1.489812	-2.464757	0.000983
C	2.711501	-0.715829	0.000143
H	3.637073	-1.277036	0.000758
C	1.560454	1.397366	-0.000366
C	2.733908	0.703343	-0.000230
H	3.680068	1.229756	-0.000687
H	1.570034	2.481766	-0.000465

---

22 (X = O)
S0
E = -477.036684458 A.U.
G = -476.928865 A.U.

C	2.226325	1.073007	-0.019539
C	-0.260304	-0.630557	-0.263514
C	-0.241535	0.764127	-0.154163
C	0.997242	1.523010	-0.316501
H	3.081254	1.728540	-0.135666
H	0.900805	2.553869	-0.640219
N	2.536607	-0.149044	0.593762
C	1.893197	-1.198981	0.337289
H	2.112776	-2.136373	0.839904
C	-1.423515	-1.365980	-0.090604
H	-1.381017	-2.443620	-0.187422
C	-2.617001	-0.700958	0.162750
H	-3.532768	-1.267151	0.278952
C	-1.459932	1.410752	0.093862
C	-2.634293	0.690059	0.253991
H	-3.564752	1.209926	0.444986
H	-1.469692	2.492566	0.165706
O	0.902006	-1.310166	-0.618000

T1
E = -476.961063726 A.U.
G = -476.858058 A.U.

C	2.266525	1.168950	0.000069
C	-0.272675	-0.672783	-0.000216
C	-0.259416	0.760186	-0.000012
C	0.872109	1.567790	0.000287
H	2.988267	1.979440	0.000593
H	0.702320	2.638553	0.000699
N	2.780092	-0.039662	-0.000682
C	2.138457	-1.187389	-0.000565

H	2.700268	-2.109557	-0.001066
C	-1.475752	-1.382815	-0.000219
H	-1.418622	-2.464239	0.000044
C	-2.688182	-0.726808	-0.000293
H	-3.609238	-1.295052	0.000133
C	-1.551890	1.394246	0.000040
C	-2.719201	0.688476	-0.000175
H	-3.668703	1.208744	-0.000316
H	-1.569055	2.478144	0.000260
O	0.819283	-1.477189	0.001368

22 (X = S)

S0

E = -800.003905022 A.U.

G = -799.900217 A.U.

C	1.938998	1.511291	0.043607
C	-0.393757	-0.587509	0.177842
C	-0.475399	0.813889	0.229543
C	0.677331	1.679758	0.477855
H	2.668846	2.288708	0.240995
H	0.462841	2.618221	0.979499
N	2.414581	0.522829	-0.814219
C	2.136401	-0.702735	-0.712850
H	2.514312	-1.405680	-1.451056
C	-1.519753	-1.357724	-0.117489
H	-1.426047	-2.435559	-0.166359
C	-2.749305	-0.746881	-0.317474
H	-3.622806	-1.351694	-0.527464
C	-1.733403	1.406221	0.033127
C	-2.856193	0.640964	-0.235553
H	-3.814743	1.122649	-0.384109
H	-1.812726	2.486335	0.086032
S	1.123671	-1.432901	0.590646

T1

E = -799.926940805 A.U.

G = -799.829201 A.U.

C	2.022304	1.605567	0.001408
C	-0.432958	-0.581406	0.000088
C	-0.488718	0.851043	0.000785
C	0.581787	1.750186	0.002005
H	2.546294	2.558023	0.003968
H	0.282877	2.793141	0.003419
N	2.812933	0.565970	-0.003008
C	2.472328	-0.719442	-0.006001
H	3.279343	-1.444521	-0.010491
C	-1.620350	-1.334715	-0.000980
H	-1.553417	-2.417116	-0.001158
C	-2.857046	-0.729638	-0.001944
H	-3.753252	-1.336394	-0.003054
C	-1.805990	1.431041	0.000019
C	-2.945610	0.681106	-0.001301
H	-3.914552	1.164255	-0.001991
H	-1.870322	2.513055	0.000522
S	0.983626	-1.594668	0.004086

23 (COT)
S0
E = -309.522918167 A.U.
G = -309.418471 A.U.

C	0.667404	1.547731	0.395115
C	-0.667404	1.547731	0.395115
C	-1.547731	0.667404	-0.395115
C	-1.547731	-0.667404	-0.395115
C	-0.667404	-1.547731	0.395115
C	0.667404	-1.547731	0.395115
C	1.547731	-0.667404	-0.395115
C	1.547731	0.667404	-0.395115
H	1.176133	2.316762	0.972147
H	-1.176133	2.316762	0.972147
H	-2.316762	1.176133	-0.972147
H	-2.316762	-1.176133	-0.972147
H	-1.176133	-2.316762	0.972147
H	1.176133	-2.316762	0.972147
H	2.316762	-1.176133	-0.972147
H	2.316762	1.176133	-0.972147

T1
E = -309.481524283 A.U.
G = -309.377999 A.U.

C	0.700321	1.690893	0.000000
C	-0.700321	1.690893	-0.000000
C	-1.690893	0.700321	0.000000
C	-1.690893	-0.700321	-0.000000
C	-0.700321	-1.690893	0.000000
C	0.700321	-1.690893	-0.000000
C	1.690893	-0.700321	0.000000
C	1.690893	0.700321	-0.000000
H	1.116203	2.694014	0.000000
H	-1.116203	2.694014	-0.000000
H	-2.694014	1.116203	0.000000
H	-2.694014	-1.116203	-0.000000
H	-1.116203	-2.694014	0.000000
H	1.116203	-2.694014	-0.000000
H	2.694014	-1.116203	0.000000
H	2.694014	1.116203	-0.000000

---

24 (X = NH)
S0
E = -441.099314450 A.U.
G = -440.968428 A.U.

C	2.284194	0.639723	0.644509
C	2.261336	-0.694845	0.640269
C	1.574517	-1.597607	-0.300313
C	0.276283	-1.606266	-0.616081
C	-0.731624	-0.642125	-0.172697
C	-0.738500	0.744871	-0.165723
C	0.335700	1.643519	-0.603759
C	1.633387	1.575319	-0.290589
N	-1.992552	-1.055860	0.173874
C	-2.796212	0.019374	0.422341
C	-2.054557	1.154612	0.203977
H	2.910814	1.119888	1.393847

H	2.869924	-1.205561	1.383930
H	2.188888	-2.401889	-0.697673
H	-0.090270	-2.410300	-1.250052
H	0.016688	2.473516	-1.230086
H	2.283204	2.355923	-0.679378
H	-2.274828	-2.020639	0.259611
H	-3.819539	-0.110793	0.733759
H	-2.404149	2.171425	0.297328

T1
E = -441.055760184 A.U.
G = -440.927123 A.U.

C	2.598759	0.685207	0.000273
C	2.577070	-0.735699	-0.000277
C	1.588670	-1.705204	-0.000311
C	0.182537	-1.695300	0.000245
C	-0.781534	-0.678220	0.000091
C	-0.787110	0.758607	-0.000084
C	0.239058	1.731057	-0.000158
C	1.638122	1.689728	0.000109
N	-2.114497	-1.056868	0.000032
C	-2.938682	0.027791	0.000229
C	-2.163982	1.154137	-0.000156
H	3.611726	1.076689	0.000627
H	3.577331	-1.158812	-0.000620
H	1.987330	-2.715286	-0.000670
H	-0.255476	-2.689564	0.000489
H	-0.159155	2.741771	-0.000329
H	2.075899	2.684088	0.000259
H	-2.424581	-2.016893	0.000292
H	-4.010285	-0.089641	0.000316
H	-2.518755	2.173093	-0.000357

---

24 (X = O)
S0
E = -460.950801269 A.U.
G = -460.832519 A.U.

C	2.255082	0.644445	0.650139
C	2.235789	-0.689764	0.644234
C	1.556649	-1.596705	-0.299244
C	0.260384	-1.604969	-0.620784
C	-0.725367	-0.623036	-0.182061
C	-0.740602	0.742107	-0.167705
C	0.322020	1.649781	-0.616158
C	1.615985	1.578659	-0.296007
C	-2.746326	-0.040053	0.429189
C	-2.072399	1.115889	0.228482
H	2.872131	1.126421	1.405689
H	2.838328	-1.200041	1.392678
H	2.172540	-2.403388	-0.688442
H	-0.124828	-2.406227	-1.244822
H	-0.002574	2.464886	-1.257385
H	2.273544	2.348580	-0.692356
H	-3.751207	-0.266956	0.743266
H	-2.457693	2.116329	0.345455
O	-1.948442	-1.104715	0.171926

T1
E = -460.909091867 A.U.
G = -460.792985 A.U.

C	2.577733	0.684081	-0.000001
C	2.558964	-0.730716	-0.000128
C	1.571212	-1.705822	-0.000037
C	0.166937	-1.690041	0.000123
C	-0.772147	-0.662960	0.000013
C	-0.790836	0.756836	0.000009
C	0.222728	1.741010	0.000033
C	1.616889	1.696782	0.000021
C	-2.893524	-0.032847	-0.000015
C	-2.184529	1.114794	-0.000013
H	3.590044	1.076553	0.000028
H	3.559606	-1.152116	-0.000257
H	1.969496	-2.715455	-0.000078
H	-0.300922	-2.668964	0.000241
H	-0.180714	2.748956	0.000056
H	2.059287	2.688550	0.000053
H	-3.950454	-0.242668	-0.000044
H	-2.579617	2.118280	-0.000021
O	-2.075913	-1.109979	0.000000

---

24 (X = S)
S0
E = -783.927345737 A.U.
G = -783.813463 A.U.

C	2.509124	0.466176	0.757100
C	2.350027	-0.858875	0.732567
C	1.673560	-1.655599	-0.304823
C	0.418349	-1.512996	-0.737856
C	-0.532068	-0.487090	-0.286640
C	-0.396463	0.877523	-0.253101
C	0.800140	1.630268	-0.675211
C	2.050293	1.441300	-0.248327
C	-2.618877	0.670776	0.418855
C	-1.606771	1.534825	0.146092
H	3.106759	0.886670	1.562865
H	2.827374	-1.440819	1.517672
H	2.245441	-2.493924	-0.695308
H	0.036144	-2.233893	-1.455323
H	0.612767	2.452583	-1.361135
H	2.814052	2.126892	-0.607958
H	-3.619569	0.893150	0.755960
H	-1.699603	2.610115	0.225321
S	-2.137953	-0.964914	0.172874

T1
E = -783.880574772 A.U.
G = -783.768882 A.U.

C	-2.941827	0.509508	-0.000123
C	-2.790474	-0.897007	0.000424
C	-1.708477	-1.762168	0.000286
C	-0.309495	-1.615125	-0.000315
C	0.572280	-0.525590	-0.000139
C	0.449417	0.896387	0.000053
C	-0.679197	1.753932	-0.000197

C	-2.067063	1.591561	-0.000230
C	2.792446	0.663982	0.000256
C	1.745635	1.521419	0.000288
H	-3.984228	0.812889	-0.000292
H	-3.744134	-1.416039	0.000872
H	-2.002970	-2.807046	0.000557
H	0.201730	-2.573856	-0.000709
H	-0.384929	2.799161	-0.000314
H	-2.586412	2.545324	-0.000457
H	3.845176	0.901761	0.000447
H	1.863460	2.597323	0.000493
S	2.275803	-0.980057	-0.000151

---

25 (X = N X' = N)
S0
E = -572.676645288 A.U.
G = -572.518102 A.U.

N	-2.729164	-1.055162	-0.443834
C	-3.464987	0.017251	-0.857915
C	-2.788419	1.151200	-0.484578
C	-1.581006	0.744293	0.161181
C	-1.572120	-0.643683	0.169942
C	-0.638595	1.686109	0.772679
C	0.697918	1.649558	0.777617
C	1.572116	0.643706	0.169852
C	1.581009	-0.744272	0.161263
C	0.638599	-1.686024	0.772859
C	-0.697915	-1.649471	0.777802
N	2.729159	1.055113	-0.443974
C	3.464982	-0.017347	-0.857932
C	2.788424	-1.151253	-0.484446
H	-2.984785	-2.021213	-0.582763
H	-4.399388	-0.114508	-1.378035
H	-3.108668	2.168744	-0.649404
H	-1.103026	2.552350	1.237618
H	1.218984	2.481931	1.244708
H	1.103030	-2.552226	1.237871
H	-1.218978	-2.481802	1.244969
H	2.984771	2.021148	-0.583030
H	4.399379	0.114350	-1.378075
H	3.108678	-2.168816	-0.649145

T1
E = -572.620793785 A.U.
G = -572.466046 A.U.

N	3.024435	1.062759	0.000096
C	3.845127	-0.029877	0.000082
C	3.076928	-1.154064	-0.000009
C	1.690665	-0.762531	-0.000053
C	1.684615	0.686080	0.000027
C	0.674602	-1.728295	-0.000200
C	-0.724019	-1.698331	-0.000101
C	-1.684615	-0.686080	-0.000041
C	-1.690665	0.762531	0.000011
C	-0.674602	1.728295	-0.000044
C	0.724019	1.698331	0.000057
N	-3.024435	-1.062759	-0.000011

C	-3.845127	0.029877	0.000076
C	-3.076928	1.154064	0.000085
H	3.337387	2.020668	0.000153
H	4.917379	0.085598	0.000137
H	3.433653	-2.172372	-0.000036
H	1.070631	-2.739825	-0.000315
H	-1.167404	-2.690940	-0.000141
H	-1.070631	2.739825	-0.000069
H	1.167404	2.690940	0.000107
H	-3.337387	-2.020668	-0.000036
H	-4.917379	-0.085598	0.000119
H	-3.433653	2.172372	0.000146

25 (X = N X' = O)

S0

E = -592.528584012 A.U.

G = -592.382606 A.U.

N	2.710690	1.056008	-0.442208
C	3.445617	-0.016775	-0.854991
C	2.769159	-1.150344	-0.480117
C	1.561641	-0.742544	0.163168
C	1.553149	0.646197	0.169736
C	0.619775	-1.687957	0.769926
C	-0.716361	-1.648783	0.778796
C	-1.565777	-0.625645	0.177617
C	-1.576673	0.740611	0.158771
C	-0.650938	1.689406	0.786221
C	0.683985	1.652080	0.785637
C	-3.403648	-0.036953	-0.869079
C	-2.787231	1.116028	-0.524585
H	2.968107	2.021743	-0.581274
H	4.379902	0.114064	-1.375421
H	3.089185	-2.168139	-0.643026
H	1.083872	-2.559788	1.224068
H	-1.256564	-2.478034	1.225609
H	-1.125596	2.540507	1.266457
H	1.207445	2.476996	1.262809
H	-4.316767	-0.262331	-1.393806
H	-3.132686	2.118493	-0.722145
O	-2.683490	-1.100937	-0.441801

T1

E = -592.478164361 A.U.

G = -592.335361 A.U.

N	-3.003908	1.061245	-0.000822
C	-3.827714	-0.027398	0.000234
C	-3.059240	-1.152802	0.000813
C	-1.675532	-0.761144	0.000370
C	-1.668729	0.684112	-0.000328
C	-0.660917	-1.732244	0.000103
C	0.738215	-1.694797	-0.000430
C	1.669792	-0.671913	-0.000309
C	1.686625	0.762825	0.000311
C	0.685211	1.738490	0.000253
C	-0.709910	1.705391	-0.000353
C	3.794783	-0.027498	0.000027
C	3.087892	1.116482	0.000576

H	-3.315522	2.020274	0.001178
H	-4.899421	0.091022	0.000179
H	-3.415467	-2.171149	0.001100
H	-1.055324	-2.743753	0.000320
H	1.211952	-2.671529	-0.000807
H	1.085888	2.747540	0.000540
H	-1.157912	2.695394	-0.000783
H	4.853270	-0.231166	-0.000087
H	3.484089	2.119729	0.001055
O	2.984118	-1.115262	-0.000567

25 (X = N X' = S)
S0
E = -915.504069422 A.U.
G = -915.362550 A.U.

N	2.950507	0.942032	-0.594886
C	3.577043	-0.188164	-1.035374
C	2.877507	-1.267082	-0.555121
C	1.764076	-0.764515	0.183275
C	1.836615	0.620246	0.137833
C	0.831215	-1.592854	0.950624
C	-0.495330	-1.460658	1.051567
C	-1.336146	-0.472739	0.359603
C	-1.263431	0.895428	0.289173
C	-0.251064	1.767231	0.913936
C	1.077453	1.668104	0.821172
C	-3.244620	0.534549	-0.890564
C	-2.371260	1.463942	-0.424849
H	3.246586	1.884726	-0.798926
H	4.459555	-0.131614	-1.650997
H	3.122003	-2.306539	-0.711281
H	1.281302	-2.434072	1.472188
H	-1.032436	-2.194273	1.646191
H	-0.652448	2.629140	1.440411
H	1.674568	2.455450	1.275058
H	-4.144309	0.686793	-1.466570
H	-2.492025	2.528803	-0.576348
S	-2.758043	-1.058348	-0.454573

T1
E = -915.449991306 A.U.
G = -915.311561 A.U.

N	-3.367708	0.974211	-0.000497
C	-4.136729	-0.154768	0.000067
C	-3.313856	-1.241569	0.000402
C	-1.953067	-0.779859	0.000203
C	-2.018275	0.661406	-0.000207
C	-0.882821	-1.687371	0.000097
C	0.512543	-1.580199	-0.000102
C	1.432185	-0.533705	-0.000100
C	1.368740	0.907437	0.000126
C	0.291464	1.807155	-0.000016
C	-1.100744	1.716481	-0.000070
C	3.705663	0.577253	0.000187
C	2.698518	1.473180	0.000273
H	-3.726311	1.916769	0.000477
H	-5.212949	-0.089116	-0.000017

H	-3.619444	-2.276127	0.000564
H	-1.219868	-2.719680	0.000201
H	0.987970	-2.557832	-0.000256
H	0.629016	2.839233	0.000013
H	-1.585729	2.688854	-0.000180
H	4.766964	0.774247	0.000266
H	2.861255	2.543454	0.000460
S	3.129459	-1.058245	-0.000200

25 (X = O X' = O)
S0
E = -612.379708200 A.U.
G = -612.246330 A.U.

C	3.378580	0.037740	-0.872389
C	2.764063	-1.115203	-0.523921
C	1.555772	-0.738940	0.161490
C	1.545600	0.627704	0.178574
C	0.631805	-1.690259	0.789143
C	-0.702496	-1.650232	0.791955
C	-1.545582	-0.627714	0.178548
C	-1.555774	0.738931	0.161521
C	-0.631797	1.690258	0.789138
C	0.702507	1.650228	0.791956
C	-3.378621	-0.037732	-0.872304
C	-2.764049	1.115204	-0.523918
H	4.289295	0.262581	-1.401369
H	3.108763	-2.117862	-0.721253
H	1.106839	-2.544425	1.262991
H	-1.245751	-2.469416	1.253177
H	-1.106822	2.544462	1.262924
H	1.245754	2.469447	1.253123
H	-4.289361	-0.262574	-1.401241
H	-3.108732	2.117867	-0.721262
O	2.660655	1.101977	-0.442872
O	-2.660659	-1.101976	-0.442859

T1
E = -612.333312571 A.U.
G = -612.202901 A.U.

C	-3.777876	-0.030628	-0.000095
C	-3.072040	1.114804	-0.000051
C	-1.672538	0.760504	0.000050
C	-1.654320	-0.669632	0.000012
C	-0.671495	1.741321	0.000270
C	0.723424	1.701266	-0.000084
C	1.654320	0.669632	0.000047
C	1.672538	-0.760504	0.000064
C	0.671495	-1.741321	0.000255
C	-0.723424	-1.701266	-0.000120
C	3.777876	0.030628	-0.000037
C	3.072040	-1.114804	-0.000020
H	-4.835300	-0.238763	-0.000203
H	-3.467850	2.118005	-0.000068
H	-1.070691	2.750373	0.000443
H	1.201880	2.675256	-0.000212
H	1.070691	-2.750373	0.000419
H	-1.201881	-2.675256	-0.000268

H	4.835300	0.238763	-0.000126
H	3.467850	-2.118006	-0.000045
O	-2.962623	-1.113473	-0.000136
O	2.962623	1.113473	-0.000075

---

25 (X = O X' = S)
S0
E = -935.355235711 A.U.
G = -935.226272 A.U.

C	-3.522613	-0.133348	-1.041187
C	-2.878353	-1.235687	-0.594950
C	-1.760093	-0.763053	0.177786
C	-1.829626	0.600408	0.140890
C	-0.842185	-1.598412	0.956389
C	0.482399	-1.462858	1.055889
C	1.320583	-0.474300	0.359859
C	1.246893	0.894464	0.290438
C	0.231595	1.771034	0.904943
C	-1.096093	1.667900	0.811483
C	3.230831	0.533872	-0.882586
C	2.356568	1.463611	-0.418973
H	-4.391161	0.018175	-1.659652
H	-3.147188	-2.262743	-0.784105
H	-1.303603	-2.426671	1.486863
H	1.020028	-2.188176	1.659904
H	0.630054	2.641145	1.419558
H	-1.712306	2.452890	1.239905
H	4.131255	0.685997	-1.457435
H	2.477417	2.528368	-0.570247
S	2.745241	-1.058360	-0.447002
O	-2.908473	0.987873	-0.592831

T1
E = -935.304696785 A.U.
G = -935.178631 A.U.

C	4.088291	-0.095504	-0.000027
C	3.326844	-1.205246	0.000030
C	1.948081	-0.780183	-0.000013
C	2.001641	0.647307	-0.000040
C	0.890945	-1.697558	-0.000034
C	-0.500586	-1.587166	0.000130
C	-1.419380	-0.531420	0.000017
C	-1.356719	0.904943	-0.000005
C	-0.279622	1.809267	-0.000132
C	1.113457	1.711128	-0.000088
C	-3.690820	0.572712	0.000185
C	-2.684938	1.471474	0.000042
H	5.154747	0.059914	-0.000030
H	3.673015	-2.226578	0.000093
H	1.232415	-2.727792	-0.000076
H	-0.980844	-2.561979	0.000217
H	-0.615010	2.841479	-0.000215
H	1.627899	2.666633	-0.000140
H	-4.752755	0.765792	0.000265
H	-2.848193	2.541419	0.000050
S	-3.108724	-1.056104	-0.000018
O	3.328142	1.027534	-0.000035

25 (X = S X' = S)
S0
E = -1258.33149995 A.U.
G = -1258.206966 A.U.

C	3.339018	0.667567	-1.020426
C	2.469382	1.540359	-0.449188
C	1.457305	0.894886	0.335578
C	1.600083	-0.469618	0.350458
C	0.462400	1.670399	1.097082
C	-0.860769	1.493642	1.101275
C	-1.600099	0.469652	0.350359
C	-1.457316	-0.894853	0.335616
C	-0.462438	-1.670290	1.097234
C	0.860730	-1.493534	1.101452
C	-3.338982	-0.667671	-1.020475
C	-2.469364	-1.540403	-0.449124
H	4.174154	0.879914	-1.669929
H	2.526986	2.613579	-0.575214
H	0.860215	2.507913	1.664105
H	-1.466666	2.194404	1.668791
H	-0.860274	-2.507744	1.664330
H	1.466610	-2.194236	1.669060
H	-4.174098	-0.880074	-1.669987
H	-2.526961	-2.613636	-0.575049
S	-2.970049	0.960624	-0.598697
S	2.970070	-0.960683	-0.598500

T1
E = -1258.27607409 A.U.
G = -1258.154396 A.U.

C	-3.981250	0.666858	0.000146
C	-2.941586	1.526642	0.000082
C	-1.638137	0.906214	-0.000006
C	-1.757917	-0.524668	-0.000131
C	-0.518881	1.754643	-0.000269
C	0.870064	1.601802	0.000064
C	1.757917	0.524668	-0.000036
C	1.638136	-0.906214	-0.000042
C	0.518880	-1.754643	-0.000412
C	-0.870064	-1.601802	-0.000102
C	3.981250	-0.666857	0.000195
C	2.941586	-1.526642	0.000025
H	-5.034792	0.901822	0.000227
H	-3.062811	2.602143	0.000177
H	-0.807100	2.801339	-0.000463
H	1.375984	2.563582	0.000181
H	0.807100	-2.801339	-0.000693
H	-1.375984	-2.563582	-0.000086
H	5.034791	-0.901822	0.000283
H	3.062811	-2.602143	0.000027
S	3.464505	0.984839	0.000193
S	-3.464505	-0.984839	0.000011

26 (X = N X' = N)
S0

E = -572.676144975 A.U.
G = -572.517625 A.U.

N	-2.719513	-1.085270	-0.423310
C	-3.470396	-0.030676	-0.851429
C	-2.807748	1.119216	-0.499098
C	-1.592621	0.742286	0.148546
C	-1.565117	-0.645056	0.178145
C	-0.668477	1.710668	0.752664
C	0.668481	1.710669	0.752662
C	1.592624	0.742286	0.148543
C	1.565113	-0.645057	0.178136
C	0.668484	-1.627022	0.786311
C	-0.668485	-1.627022	0.786315
C	2.807753	1.119214	-0.499099
C	3.470395	-0.030679	-0.851437
N	2.719507	-1.085272	-0.423324
H	-2.959721	-2.056831	-0.550692
H	-4.404891	-0.183435	-1.365724
H	-3.144855	2.129171	-0.676650
H	-1.156805	2.566238	1.213352
H	1.156810	2.566239	1.213347
H	1.164946	-2.474720	1.252166
H	-1.164944	-2.474720	1.252173
H	3.144864	2.129168	-0.676647
H	4.404888	-0.183440	-1.365734
H	2.959710	-2.056834	-0.550710

T1
E = -572.621111793 A.U.
G = -572.466291 A.U.

N	3.011164	-1.083195	-0.000338
C	3.846384	0.000060	-0.000248
C	3.090307	1.132743	-0.000017
C	1.701053	0.758292	0.000070
C	1.676741	-0.690516	0.000001
C	0.695415	1.742222	0.000175
C	-0.695415	1.742222	0.000174
C	-1.701053	0.758292	0.000070
C	-1.676741	-0.690516	0.000001
C	-0.703442	-1.684386	0.000400
C	0.703442	-1.684386	0.000400
C	-3.090307	1.132743	-0.000017
C	-3.846384	0.000060	-0.000248
N	-3.011164	-1.083195	-0.000338
H	3.312307	-2.044884	-0.000342
H	4.916983	-0.128969	-0.000442
H	3.458794	2.146908	0.000015
H	1.114524	2.745174	0.000264
H	-1.114524	2.745174	0.000264
H	-1.123522	-2.686352	0.000589
H	1.123522	-2.686352	0.000588
H	-3.458794	2.146908	0.000016
H	-4.916983	-0.128969	-0.000442
H	-3.312307	-2.044884	-0.000340

---

26 (X = N X' = O)
S0

E = -592.528201797 A.U.
G = -592.382233 A.U.

N	-2.702070	-1.082662	-0.419372
C	-3.450893	-0.027921	-0.848609
C	-2.787364	1.121766	-0.496812
C	-1.572918	0.743830	0.149389
C	-1.546650	-0.644306	0.179866
C	-0.653048	1.712880	0.759599
C	0.682584	1.715073	0.763182
C	1.589730	0.738534	0.147279
C	1.557632	-0.626779	0.184947
C	0.685183	-1.626741	0.788612
C	-0.651217	-1.630042	0.783918
C	2.809227	1.081599	-0.536725
C	3.408404	-0.086667	-0.861350
H	-2.943815	-2.054039	-0.546649
H	-4.385553	-0.179838	-1.362722
H	-3.123896	2.131923	-0.673483
H	-1.143985	2.562462	1.228016
H	1.180474	2.558256	1.234238
H	1.201554	-2.469753	1.237388
H	-1.147368	-2.482940	1.240104
H	3.172832	2.075129	-0.746576
H	4.320032	-0.334370	-1.378666
O	2.670023	-1.131944	-0.421979

T1
E = -592.478514370 A.U.
G = -592.335697 A.U.

N	2.993695	-1.080765	-0.000358
C	3.828369	-0.001525	-0.000039
C	3.069546	1.132793	0.000361
C	1.685110	0.757626	0.000227
C	1.661576	-0.688172	-0.000127
C	0.680273	1.750263	0.000181
C	-0.707661	1.754188	-0.000099
C	-1.698137	0.759084	-0.000136
C	-1.661466	-0.675629	0.000049
C	-0.716144	-1.679253	0.000120
C	0.690852	-1.687693	0.000209
C	-3.101847	1.093096	-0.000436
C	-3.795316	-0.060187	-0.000005
H	3.294303	-2.043277	-0.000174
H	4.898843	-0.130260	-0.000201
H	3.437575	2.147000	0.000561
H	1.104509	2.750589	0.000325
H	-1.131651	2.754366	-0.000267
H	-1.166698	-2.666267	0.000217
H	1.108943	-2.689790	0.000311
H	-3.511163	2.091113	-0.000732
H	-4.850688	-0.278851	0.000047
O	-2.968846	-1.137102	0.000073

---

26 (X = N X' = S)
S0
E = -915.503811861 A.U.
G = -915.362294 A.U.

N	-2.814580	-1.199113	-0.487555
C	-3.583323	-0.208318	-1.024802
C	-3.030875	0.994287	-0.658122
C	-1.861541	0.715986	0.110656
C	-1.752105	-0.664520	0.196777
C	-1.052549	1.732527	0.791797
C	0.277970	1.800299	0.887568
C	1.274786	0.901622	0.272871
C	1.330965	-0.465895	0.365971
C	0.469308	-1.430791	1.061803
C	-0.859711	-1.532081	0.961780
C	2.392773	1.440299	-0.448164
C	3.255447	0.491170	-0.894095
H	-2.983552	-2.187484	-0.599266
H	-4.450877	-0.438237	-1.621090
H	-3.411382	1.972961	-0.907279
H	-1.620720	2.545003	1.238613
H	0.701096	2.657629	1.404822
H	0.985779	-2.174859	1.661390
H	-1.340342	-2.356192	1.483286
H	2.528876	2.500525	-0.618130
H	4.158489	0.620775	-1.470436
S	2.747989	-1.087115	-0.431454

T1
E = -915.450289989 A.U.
G = -915.311761 A.U.

N	-3.247182	-1.169211	0.000436
C	-4.137315	-0.134709	-0.000133
C	-3.439867	1.038974	-0.000749
C	-2.039384	0.734473	-0.000455
C	-1.940079	-0.705419	0.000198
C	-1.076363	1.764076	-0.000526
C	0.309875	1.828061	0.000074
C	1.379987	0.909698	0.000309
C	1.424844	-0.532661	-0.000050
C	0.493671	-1.561057	0.000048
C	-0.910775	-1.642589	0.000234
C	2.714217	1.457393	0.000636
C	3.712593	0.549522	0.000425
H	-3.496705	-2.146322	0.000849
H	-5.199520	-0.319843	-0.000030
H	-3.860654	2.032302	-0.001226
H	-1.539565	2.746998	-0.000936
H	0.669315	2.853338	0.000249
H	0.947835	-2.548187	-0.000042
H	-1.270921	-2.667168	0.000466
H	2.890369	2.525562	0.000988
H	4.776121	0.733562	0.000529
S	3.116598	-1.078771	-0.000248

26 (X = O X' = O)
S0
E = -612.379459793 A.U.
G = -612.246094 A.U.

C	-3.381588	-0.084331	-0.866555
C	-2.783441	1.084206	-0.540155

C	-1.568036	0.740435	0.148682
C	-1.537803	-0.625344	0.188865
C	-0.667011	1.715621	0.777750
C	0.667012	1.715622	0.777750
C	1.568037	0.740435	0.148682
C	1.537802	-0.625345	0.188863
C	0.667869	-1.627889	0.793318
C	-0.667870	-1.627889	0.793319
C	2.783444	1.084205	-0.540151
C	3.381587	-0.084332	-0.866556
H	-4.290604	-0.331770	-1.388429
H	-3.145402	2.077746	-0.752205
H	-1.168619	2.548002	1.263379
H	1.168619	2.548003	1.263378
H	1.184600	-2.473610	1.236155
H	-1.184601	-2.473610	1.236157
H	3.145407	2.077745	-0.752198
H	4.290603	-0.331772	-1.388431
O	-2.647205	-1.129569	-0.421291
O	2.647203	-1.129570	-0.421293

T1
E = -612.333761369 A.U.
G = -612.203313 A.U.

C	0.061050	-0.000095	3.778324
C	-1.093478	0.000079	3.083317
C	-0.757164	0.000108	1.682953
C	0.673386	-0.000091	1.646968
C	-1.760542	-0.000067	0.692359
C	-1.760542	-0.000067	-0.692359
C	-0.757164	0.000108	-1.682953
C	0.673386	-0.000091	-1.646968
C	1.682897	0.000363	-0.702951
C	1.682897	0.000363	0.702951
C	-1.093478	0.000079	-3.083317
C	0.061050	-0.000095	-3.778324
H	0.280096	-0.000177	4.833612
H	-2.091770	0.000272	3.491426
H	-2.758239	-0.000194	1.121187
H	-2.758239	-0.000194	-1.121187
H	2.669698	0.000574	-1.152842
H	2.669698	0.000574	1.152842
H	-2.091770	0.000272	-3.491426
H	0.280096	-0.000177	-4.833612
O	1.132916	-0.000282	2.951333
O	1.132916	-0.000282	-2.951333

---

26 (X = O X' = S)
S0
E = -935.355004038 A.U.
G = -935.226065 A.U.

C	-3.519348	-0.263863	-1.027966
C	-3.029719	0.952642	-0.695445
C	-1.859942	0.711364	0.106930
C	-1.746140	-0.646382	0.199513
C	-1.068732	1.735906	0.796867
C	0.260415	1.801267	0.891584

C	1.255948	0.902441	0.273239
C	1.315922	-0.465548	0.366991
C	0.454210	-1.437058	1.054648
C	-0.874522	-1.535477	0.956040
C	2.373590	1.444180	-0.444225
C	3.239479	0.496389	-0.887460
H	-4.358508	-0.588391	-1.619900
H	-3.434858	1.911440	-0.977403
H	-1.647236	2.535276	1.251823
H	0.684616	2.652225	1.418025
H	0.970469	-2.189182	1.643998
H	-1.372612	-2.360315	1.456740
H	2.507636	2.504617	-0.613679
H	4.143337	0.628018	-1.462026
S	2.737035	-1.082013	-0.423979
O	-2.761545	-1.244581	-0.482277

T1
E = -935.305076680 A.U.
G = -935.178908 A.U.

C	4.083071	-0.193291	-0.000237
C	3.451572	0.997353	-0.000461
C	2.036199	0.733714	-0.000211
C	1.924380	-0.690839	0.000106
C	1.088770	1.773179	-0.000200
C	-0.295269	1.833625	0.000199
C	-1.366584	0.907788	0.000268
C	-1.413272	-0.530167	-0.000020
C	-0.483649	-1.564711	0.000313
C	0.921281	-1.638776	0.000409
C	-2.697424	1.456864	0.000372
C	-3.696765	0.546789	0.000074
H	5.125166	-0.468473	-0.000275
H	3.912538	1.972239	-0.000715
H	1.556509	2.753279	-0.000427
H	-0.658788	2.857064	0.000346
H	-0.936575	-2.551854	0.000422
H	1.312503	-2.650528	0.000670
H	-2.873808	2.524737	0.000637
H	-4.760250	0.730990	0.000003
S	-3.099610	-1.074478	-0.000306
O	3.200325	-1.220621	0.000072

---

26 (X = S X' = S)
S0
E = -1258.33134832 A.U.
G = -1258.206836 A.U.

C	3.399501	0.293063	-1.059297
C	2.641756	1.326397	-0.608985
C	1.556222	0.907298	0.229231
C	1.532132	-0.453209	0.405055
C	0.666995	1.885369	0.883789
C	-0.666995	1.885369	0.883788
C	-1.556222	0.907298	0.229230
C	-1.532130	-0.453209	0.405054
C	-0.667923	-1.283285	1.252522
C	0.667924	-1.283284	1.252524

C	-2.641756	1.326397	-0.608984
C	-3.399502	0.293063	-1.059296
H	4.255763	0.324184	-1.715263
H	2.831491	2.362127	-0.858551
H	1.172924	2.722727	1.357540
H	-1.172924	2.722727	1.357537
H	-1.174308	-1.990419	1.903291
H	1.174308	-1.990419	1.903293
H	-2.831492	2.362126	-0.858549
H	-4.255765	0.324183	-1.715259
S	-2.833657	-1.217026	-0.456307
S	2.833657	-1.217026	-0.456307

T1
E = -1258.27640041 A.U.
G = -1258.154769 A.U.

C	4.029007	0.423479	-0.000347
C	3.080464	1.386195	-0.000128
C	1.723658	0.904942	0.000104
C	1.694703	-0.531213	0.000122
C	0.693188	1.870198	0.000248
C	-0.693188	1.870198	0.000244
C	-1.723658	0.904942	0.000098
C	-1.694703	-0.531213	0.000124
C	-0.704232	-1.504687	0.000648
C	0.704232	-1.504687	0.000645
C	-3.080464	1.386195	-0.000138
C	-4.029007	0.423479	-0.000352
H	5.100910	0.550576	-0.000601
H	3.312508	2.443290	-0.000117
H	1.097263	2.878436	0.000300
H	-1.097263	2.878436	0.000292
H	-1.099331	-2.516560	0.000959
H	1.099331	-2.516560	0.000952
H	-3.312508	2.443290	-0.000133
H	-5.100910	0.550576	-0.000608
S	-3.347934	-1.165577	-0.000268
S	3.347934	-1.165577	-0.000273

---

27 (X = NH)
S0
E = -457.155245082 A.U.
G = -457.035364 A.U.

C	-2.256878	-0.645269	0.635490
C	-2.243729	0.689171	0.632111
C	-1.564626	1.601084	-0.306186
C	-0.265853	1.624080	-0.614967
C	0.733540	0.656044	-0.166356
C	0.751853	-0.722304	-0.162639
C	-0.299011	-1.646740	-0.598559
C	-1.597444	-1.583335	-0.292676
N	2.007413	1.023843	0.200650
C	2.719341	-0.107991	0.419457
N	2.001193	-1.182221	0.194989
H	-2.881986	-1.129125	1.383471
H	-2.857605	1.193621	1.375443
H	-2.187433	2.395702	-0.709281

H	0.102908	2.423602	-1.252036
H	0.048193	-2.476079	-1.208654
H	-2.241207	-2.370704	-0.677226
H	2.344002	1.970049	0.306255
H	3.749720	-0.086856	0.738508

T1
E = -457.111396408 A.U.
G = -456.993836 A.U.

C	-2.571039	-0.691079	0.000227
C	-2.557426	0.727744	-0.000192
C	-1.576268	1.709547	-0.000224
C	-0.173886	1.709917	0.000122
C	0.784460	0.685828	0.000072
C	0.800167	-0.742589	-0.000086
C	-0.208333	-1.727770	-0.000108
C	-1.605897	-1.693616	0.000154
N	2.128703	1.024032	0.000178
C	2.861662	-0.113397	-0.000023
N	2.118012	-1.183907	-0.000142
H	-3.581845	-1.087355	0.000491
H	-3.560436	1.143747	-0.000425
H	-1.984672	2.715359	-0.000454
H	0.264221	2.703323	0.000294
H	0.220054	-2.724501	-0.000236
H	-2.039136	-2.689548	0.000317
H	2.492995	1.965871	0.000118
H	3.941171	-0.095275	-0.000016

---

27 (X = O)
S0
E = -477.006494583 A.U.
G = -476.899391 A.U.

C	2.235868	0.650099	0.630519
C	2.225400	-0.684063	0.626470
C	1.545774	-1.604278	-0.304685
C	0.246374	-1.629387	-0.609751
C	-0.726680	-0.638821	-0.170979
C	-0.751519	0.719992	-0.160555
C	0.281524	1.659665	-0.600396
C	1.579382	1.591182	-0.298176
C	-2.676398	0.049843	0.422727
N	-2.024950	1.141234	0.219417
H	2.859265	1.134390	1.379191
H	2.840422	-1.186599	1.369743
H	2.166529	-2.404703	-0.698364
H	-0.144232	-2.430860	-1.229102
H	-0.074546	2.480733	-1.215284
H	2.226215	2.373133	-0.687758
H	-3.698410	-0.072202	0.744230
O	-1.969867	-1.070991	0.198797

T1
E = -476.965231661 A.U.
G = -476.860364 A.U.

C	2.547928	0.690647	0.000025
C	2.536046	-0.723678	-0.000113

C	1.557504	-1.712341	-0.000047
C	0.156088	-1.707845	0.000120
C	-0.773394	-0.672026	0.000003
C	-0.798604	0.740657	0.000007
C	0.191221	1.739914	0.000007
C	1.585438	1.702553	0.000011
C	-2.820034	0.055894	-0.000013
N	-2.139858	1.141709	-0.000005
H	3.558866	1.085962	0.000083
H	3.539815	-1.137140	-0.000227
H	1.966786	-2.717197	-0.000093
H	-0.311453	-2.686484	0.000249
H	-0.243184	2.733533	0.000001
H	2.024115	2.695567	0.000029
H	-3.893207	-0.056808	-0.000028
O	-2.094486	-1.074006	0.000002

27 (X = S)

S0

E = -799.976503226 A.U.

G = -799.873887 A.U.

C	2.491054	0.464877	0.748386
C	2.338521	-0.860901	0.720104
C	1.662560	-1.658244	-0.317290
C	0.404320	-1.522817	-0.741586
C	-0.542919	-0.501074	-0.278103
C	-0.413954	0.861617	-0.246081
C	0.768226	1.635470	-0.663638
C	2.020718	1.447613	-0.244837
C	-2.516773	0.742574	0.401121
N	-1.546869	1.555273	0.131180
H	3.092981	0.884742	1.551103
H	2.824998	-1.442567	1.499471
H	2.239730	-2.487219	-0.719166
H	0.019314	-2.234628	-1.465807
H	0.557315	2.466990	-1.329902
H	2.777624	2.144261	-0.597012
H	-3.497286	1.053863	0.735489
S	-2.153570	-0.932940	0.196194

T1

E = -799.928974972 A.U.

G = -799.828699 A.U.

C	-2.921819	0.511315	0.000046
C	-2.773536	-0.893791	0.000039
C	-1.693558	-1.765674	-0.000030
C	-0.296579	-1.627392	-0.000061
C	0.586255	-0.536476	-0.000020
C	0.463387	0.882283	-0.000024
C	-0.655818	1.746974	-0.000093
C	-2.042958	1.591017	-0.000017
C	2.686416	0.739949	0.000034
N	1.686168	1.547338	0.000008
H	-3.963043	0.817741	0.000103
H	-3.728310	-1.410380	0.000083
H	-1.994309	-2.808494	-0.000053
H	0.211054	-2.587089	-0.000107

H	-0.330679	2.781407	-0.000169
H	-2.557268	2.547172	-0.000027
H	3.720050	1.060757	0.000079
S	2.295537	-0.945107	0.000049

28 (X = NH)
S0
E = -457.131367838 A.U.
G = -457.011540 A.U.

C	2.257860	0.639209	0.640011
C	2.226370	-0.695331	0.645815
C	1.550990	-1.603654	-0.298099
C	0.259196	-1.615463	-0.632325
C	-0.740185	-0.641564	-0.184607
C	-0.739886	0.745939	-0.174994
C	0.322658	1.655048	-0.614751
C	1.618570	1.578935	-0.298380
N	-1.986693	-1.014226	0.193444
N	-2.794771	0.018162	0.461760
C	-2.052268	1.089260	0.227946
H	2.883358	1.118443	1.390476
H	2.826121	-1.203913	1.397403
H	2.172431	-2.406269	-0.687500
H	-0.107691	-2.409043	-1.276648
H	0.002396	2.483194	-1.241178
H	2.274039	2.356430	-0.682851
H	-2.330710	-1.958210	0.295082
H	-2.469521	2.077536	0.355097

T1
E = -457.083674295 A.U.
G = -456.966538 A.U.

C	-2.572981	0.693911	-0.000273
C	-2.548792	-0.734143	0.000219
C	-1.572480	-1.714275	0.000189
C	-0.169002	-1.711683	-0.000137
C	0.784835	-0.677252	-0.000073
C	0.782233	0.749217	0.000172
C	-0.220450	1.741810	0.000090
C	-1.622151	1.701018	-0.000327
N	2.115072	-1.015420	-0.000255
N	2.949615	0.030908	0.000094
C	2.169518	1.087177	0.000266
H	-3.588319	1.077871	-0.000559
H	-3.551728	-1.150553	0.000532
H	-1.980553	-2.720036	0.000417
H	0.275595	-2.702474	-0.000329
H	0.188669	2.747747	0.000239
H	-2.062503	2.693890	-0.000647
H	2.491934	-1.952169	0.000316
H	2.589718	2.082634	0.000404

28 (X = O)
S0
E = -476.964110481 A.U.
G = -476.857556 A.U.

C	2.232062	0.649629	0.644490
C	2.202953	-0.684445	0.652170
C	1.536181	-1.597252	-0.295023
C	0.246229	-1.612358	-0.635469
C	-0.735743	-0.626463	-0.190512
C	-0.738519	0.736145	-0.175763
C	0.308296	1.657224	-0.627914
C	1.601546	1.583840	-0.306610
N	-2.770421	-0.026301	0.473043
C	-2.065703	1.044690	0.248304
H	2.848240	1.132963	1.399432
H	2.795502	-1.192374	1.409442
H	2.159966	-2.401808	-0.675789
H	-0.137442	-2.405491	-1.268599
H	-0.021474	2.468745	-1.269454
H	2.260976	2.352483	-0.701079
H	-2.513796	2.017290	0.393550
O	-1.940354	-1.086719	0.189895

T1
E = -476.919230506 A.U.
G = -476.815084 A.U.

C	2.553880	0.700858	0.000082
C	2.535555	-0.719393	-0.000004
C	1.560893	-1.708479	-0.000080
C	0.160577	-1.706298	0.000043
C	-0.776325	-0.668491	-0.000011
C	-0.783819	0.738810	0.000026
C	0.200132	1.746778	-0.000099
C	1.597672	1.711012	-0.000081
N	-2.932173	-0.015793	0.000045
C	-2.186423	1.041117	0.000089
H	3.567032	1.089604	0.000197
H	3.539459	-1.132676	-0.000016
H	1.972283	-2.712326	-0.000152
H	-0.305708	-2.685813	0.000107
H	-0.218196	2.748114	-0.000183
H	2.036484	2.703986	-0.000146
H	-2.641110	2.021907	0.000135
O	-2.074737	-1.092215	-0.000005

---

28 (X = S)
S0
E = -799.967703465 A.U.
G = -799.865150 A.U.

C	2.490086	0.464682	0.750525
C	2.327922	-0.859933	0.729248
C	1.660016	-1.661334	-0.310819
C	0.407459	-1.525919	-0.751448
C	-0.538554	-0.499470	-0.294310
C	-0.398918	0.865424	-0.256037
C	0.783527	1.637673	-0.674998
C	2.034171	1.444619	-0.251730
N	-2.620661	0.665952	0.441926
C	-1.626834	1.467313	0.162044
H	3.085087	0.884389	1.558417
H	2.799646	-1.440531	1.518313

H	2.237269	-2.497991	-0.696483
H	0.024717	-2.241545	-1.472401
H	0.589709	2.462770	-1.354663
H	2.797666	2.132582	-0.606386
H	-1.770640	2.538189	0.260167
S	-2.140755	-0.906116	0.192795

T1
E = -799.920159933 A.U.
G = -799.819982 A.U.

C	2.914974	0.520159	-0.000104
C	2.767058	-0.890962	-0.000092
C	1.699110	-1.774544	0.000025
C	0.302078	-1.640831	0.000130
C	-0.571453	-0.541336	0.000022
C	-0.446788	0.872451	0.000041
C	0.654819	1.757619	0.000011
C	2.045091	1.602240	-0.000064
N	-2.796682	0.666910	0.000016
C	-1.765029	1.454749	0.000050
H	3.957729	0.820831	-0.000166
H	3.726292	-1.399519	-0.000170
H	2.009335	-2.814359	0.000054
H	-0.210559	-2.599015	0.000249
H	0.343924	2.797976	0.000042
H	2.563345	2.556276	-0.000095
H	-1.923677	2.528866	0.000069
S	-2.280548	-0.919793	-0.000013
