# Supplementary material for: Search for improved triplet-state quenchers for fluorescence imaging: a computational framework incorporating excited-state Baird-aromaticity
Source: Chem Sci. 2025 Mar 26;16(18):7989–8001. doi: 10.1039/d5sc01131k (PMC11974263; doi:10.1039/d5sc01131k)
Supplement: SC-016-D5SC01131K-s002 [file SC-016-D5SC01131K-s002.pdf]

Supplementary Information (SI) for Chemical Science.
This journal is © The Royal Society of Chemistry 2025

SUPPORTING INFORMATION

Search of Improved Triplet-State Quenchers for Fluorescence Imaging: A Computational Framework Incorporating Excited-State Baird-Aromaticity

Ouissam El Bakouri,[1,2] Matthew A. Johnson,[1] Joshua R. Smith,[1,3] Avik K. Pati,[4,5] Maxwell I. Martin,[4] Scott C. Blanchard,[4]* and Henrik Ottosson[1]*

[1] Department of Chemistry - Ångström, Uppsala University, Uppsala, Sweden.

[2] Institut de Química Computacional i Catàlisi (IQCC) and Departament de Química, Universitat de Girona, C/ Maria Aurèlia Capmany 6, 17003 Girona, Catalonia, Spain.

[3] Department of Chemistry and Biochemistry, Cal Poly Humboldt, Arcata, CA 95501, USA.

[4] Department of Structural Biology, St. Jude Children's Research Hospital, Memphis, USA.

[5] Department of Chemistry, Birla Institute of Technology and Science, Pilani, Rajasthan 333031, India.

Table of Contents

Computational methods 4

Table S1. DLPNO-(U)CCSDT/cc-pVTZ energies (kJ/mol) using cc-pVTZ as an auxiliary basis set at M062X, B3LYP, BLYP, and CAM-B3LYP optimized S_0 and T_1 state geometries.. 6

Commercially and synthetically available compounds 7

Table S2. MCI and HOMA values for the 214 compounds computed at the M062X/6-311G(d,p).. 8

Table S3. Absolute values of the various dihedral angles in the S_0 and T_1 states (in degrees), inversion barrier in the S_0 state (kJ/mol), and vertical excitation energies from the planar transition state in the S_0 state (kJ/mol). 13

Table S4. E_{T1} (kJ/mol), SOC values (cm^{-1}), reorganization energies (kJ/mol), k_{ISC} and Gibbs free reaction energies (kJ/mol) for singlet oxygen formation [$\Delta G_R(^1O_2)$][a]. 18

Table S5. MCI and HOMA of the 6π-electron rings of 8, 10 and 15, benzene, pyrrole, furan and thiophene in the S_0 state. 23

Table S6. MCI values for the 6π- and 8π-electron rings in a selection of twelve compounds. Normal text represents values in the S_0 state, while italicized text indicates values in the T_1 state. 24

Figure S1. Thiepin compounds that have low triplet energy and cannot have 6π-electron Hückel-aromatic rings in S_0.. 25

Figure S2: Schematic depiction for the reaction of methyl radical with various species. Reaction energies as a function of the T_1 state (anti)aromatic character of the (6π)8π-electron cycle as assessed through the MCI_{T1} values. For unsymmetric structures containing heteroatoms, the methyl group was added to the furthest carbon from the heteroatom. 25

Figure S3. Variations of the triplet energies as a function of aromaticity changes based on HOMA ($\Delta HOMA_{T1\text{-}S0}$) (A) and the spin-orbit coupling in dependence of aromaticity in T_1 based on $HOMA_{T1}$ (B) at different 6- (red), 7- (blue), and 8-MR (green) compounds. 26

Figure S4. Variations of the triplet energies as a function of aromaticity changes based on normalized MCI ($\Delta MCI^{1/n}_{T1\text{-}S0}$) and direct MCI ($\Delta MCI^{1/n}_{T1\text{-}S0}$) of (A and B) monocyclic compounds **1**, **13**, **19**, **20** and **23**, and (C and D) 8-MR classes **22-28**. Triplet energies, and MCI values are computed at M06-2X/6-311G(d,p) level. 27

Figure S5. Variations of the triplet energies as a function of aromaticity changes based on normalized MCI ($\Delta MCI^{1/n}_{T1\text{-}S0}$) and direct MCI ($\Delta MCI^{1/n}_{T1\text{-}S0}$) of (A and B) azepines, (C and D) oxepines and (E and F) thiepines. Triplet energies, and MCI values are computed at M06-2X/6-311G(d,p) level.. 28

Table S7. FLU and ΔFLU/FLU (electrons) values of 11 and 15- membered ring circuits in a selection of twelve compounds. Normal text represents values in the S_0 state, while italicized text indicates values in the T_1 state. 29

Table S8. HOMA values for 11 and 15-membered ring circuits in a selection of twelve compounds. Normal text represents values in the S_0 state, while italicized text indicates values in the T1 state. 30

Figure S6. Variations of the triplet energies as a function of aromaticity changes based on HOMA (left panels) and the spin-orbit coupling in dependence of aromaticity in T_1 based on HOMA (right panels) at different geometric parameters, i.e., dihedral angles (ϕ, color scale), for the (A) 6-, (B) 7-, and (C) 8-MR compounds. Legends next to each plot show color scales for Δ(ϕT1 – ϕS0) values, with scales from yellow (positive) to orange (negative) for left panels and from white (zero) to dark red (positive) for right panels. 31

Figure S7. (A) Comparison of reorganization energy (λ) and E_{T1}; grey reference line indicated where E_{T1} is equal to λ. (B) Structures of the ten 7MR compounds that lie above the reference line. 32

Figure S8. Triplet energy versus the natural logarithm of the ISC rate for the 7- (A) and 8-MR (B) compounds. 33

Figure S9: Gibbs free energies of reaction of triplet benzene and COT with triplet oxygen. 34

Figure S10: One of the most suitable 7-MR TSQ candidate compounds according to our computations (**11** (X = NH; X' = S)). 35

Figure S11: One of the most suitable 8-MR TSQ candidate compounds according to our computations (**26** (X = O)). 35

Figure S12: One of the most suitable 8-MR TSQ candidate compounds according to our computations (**27** (X = O)). 36

Figure S13: One of the most suitable 7-MR TSQ candidate compounds according to our computations (**9** (X = NH; X' = O)) with two water molecules coordinated. 37

Substitution effects 38

Figure S14: Effects of substitution to the E_{T1} with methoxy groups for compound **14** (X= NH) and **28** (X = NH). 38

Figure S15: The list of parent heterocyclic compounds with SOCs more than 0.9 cm^{-1}, are planar in the T_1 state, have triplet energies within the ideal span (80 - 200 kJ/mol), and cannot form singlet oxygen. 39

Table S9. E_{T1}, k_{ISC}, E_{S1} (vertical) and MCI(T_1) split into smaller tables with E_{T1} ranges of 20 kJ/mol. 40

Computational methods

All geometry optimizations were performed with Gaussian 16 at the M062X/6-311G(d,p) level with the polarizable continuum model (PCM) using water as a solvent.[1-3] The triplet energies, *i.e.*, free energy differences, are adiabatic and computed as the energy differences between the singlet ground state and the first triplet state at their optimal geometries. The reorganization energies (λ) and vertical excitations were also calculated at the M062X/6-311G(d,p) level. Spin-orbit coupling (SOC) elements were computed within the TD-M062X framework using PySOC.[4]

ISC rates have been computed through the Marcus theory model,[5] where they depend on the adiabatic singlet-triplet energy gap (ΔE), the reorganization energies (λ), and the SOC ($\langle S_0|H_{SO}|T_1\rangle$):

$$k_{ISC} = \frac{2\pi}{\hbar}\langle S_0|H_{SO}|T_1\rangle^2\sqrt{\frac{1}{4\pi k_B T}}\,e^{-\left(\frac{\lambda+\Delta E}{4\lambda k_B T}\right)}$$

where $\hbar$ is the reduced Planck constant ($1.05\cdot10^{-37}$ J·s), k_B is the Boltzmann constant ($8.31\cdot10^{-3}$ kJ·mol^{-1}·K^{-1}) and T is the temperature (298.15 K). For a few molecules, the domain-based local pair natural orbital (DLPNO) methodology was employed in the CCSD(T) calculations (DLPNO-(U)CCSD(T)/cc-pVTZ//(U)DFT/6-311G(d,p)).[6-8] In the DLPNO-(U)CCSDT computations, cc-pVTZ was chosen as an auxiliary basis set at different (U)DFT optimized geometries, i.e., M062X, B3LYP, CAM-B3LYP, and BLYP. The selection of the M06-2X functional was motivated by its consistent performance in predicting triplet energies, as validated by benchmarking against DLPNO-(U)CCSDT/cc-pVTZ results (see Table S1).

Aromaticity was evaluated in terms of geometric, and electronic for which we used the multicenter index (MCI)[9] and, the harmonic oscillator model of aromaticity (HOMA)[10,11]. MCI quantifies electron delocalization among multiple centers in a molecule. It is based on the molecular wavefunction and uses the electron density overlap integrals between atomic centers. Its expression is:

$$MCI(\mathcal{A}) = \frac{1}{2N}\sum_{P(\mathcal{A})}\sum_{i_1,i_2,\ldots i_N} n_{i_1}\ldots n_{i_N} S_{i_1 i_2}(A_1) S_{i_2 i_3}(A_2)\ldots S_{i_N i_1}(A_N)$$

where $S_{\mathrm{ij}}(A)$ is the overlap of natural orbitals i and j in the atom A defined in the framework of the quantum theory of atoms in molecules (QTAIM) and, $P(\mathcal{A})$ stands for

a permutation operator which interchanges the atomic labels A_1, A_2 ... A_N to generate up to the N! permutations of the elements in the string $\mathcal{A}$. MCI reflects the extent to which electrons are shared among three or more centers, highlighting delocalization and aromaticity. Here we use the normalized MCI ($MCI^{1/n}$), where n is the size of the member ring, used to consistently compare aromaticity across rings of different sizes by mitigating the size-dependent bias of the unnormalized MCI. Higher MCI values indicate stronger electron delocalization and often correlate with increased stability of the molecule. MCI calculations were carried out with the ESI-3D program[12] using the QTAIM atomic partition and the integration scheme as implemented in the AIMAll package.[13]

HOMA is a geometric-based indicator defined as:

$$HOMA = 1 - \frac{\alpha}{n}\sum_{i=1}^{n}\left(R_{opt} - R_i\right)^2$$

where n is the number of bonds considered, α is an empirical constant (for C–C, C–N, C–O, and C–S bonds α = 257.7, 93.5, 157.4, and 94.1, respectively), R_{opt} is an optimal bond value (1.388, 1.334, 1.265, and 1.677 Å for C–C, C–N, C–O, and C-S bonds, respectively) and R_i stands for a running bond length. This expression is optimized to give HOMA = 0 for a model nonaromatic system (bonds not equal to an optimal value R_{opt}), and HOMA = 1 for fully aromatic systems with all bonds equal to R_{opt}. Negative HOMA values usually indicate antiaromaticity.

The absolute mean of the average dihedral in the S_0 state (ϕ_{S0}) and T_1 state (ϕ_{T1}) was computed as the sum of every dihedral angle in absolute, i.e., for S_0:

$$\phi_{S0} = \frac{\sum_{i=1}^{n}|\phi_i|}{n}$$

where n stands for the number of dihedral angles, or equivalently, the total number of atoms or total number of bonds in the ring).

Table S1. DLPNO-(U)CCSDT/cc-pVTZ energies (kJ/mol) using cc-pVTZ as an auxiliary basis set at M062X, B3LYP, BLYP, and CAM-B3LYP optimized S_0 and T_1 state geometries.

Compound	State	M062X	B3LYP	BLYP	CAM-B3LYP
**6** (X = X’ = O)	S_0	0.0	0.0	5.4	1.3
	T_1	0.0	5.0	2.9	1.3
**7** (X = NH; X’ = O; X’’’ = S)	S_0	0.0	0.8	7.5	1.3
	T_1	0.0	1.7	11.3	0.8
**15** (X = S)	S_0	0.0	0.0	4.6	1.3
	T_1	0.0	0.0	5.4	0.4

Commercially and synthetically available compounds

The following compounds are commercially available according to SciFinder: 6, 14 (X = S), 17, 18.

Below is the list of compounds for which their syntheses have been reported:

1 (X = X’ = O), see reference 14

1 (X = O, X’ = S), see reference 15

1 (X = X’ = S), see reference 16

2 (X = X’ = X’’’ = S), see reference 17

2 (X = X’ = X’’’ = O), see reference 18

3 (X = S, X’ = NH, X’’’ = S), see reference 19

3 (X = X’ = X’’’ = S), see reference 20

4 (X = X’ = X’’’ = S), see reference 21

5 (X = X’ = S), 5 (X = X’ = O) and 5 (X = O, X’ = S), see reference 22

5 (X = X’ = NH), see reference 23

5 (X = NH, X’ = O), see reference 24

7 (X = NH, X’ = O, X’’ = O), see reference

7 (X = S, X’ = NH, X’’ = S) and 7 (X = NH, X’ = S, X’’ = S), see reference 25

12 (X = X’ = S), see reference 26

14 (X = S), see reference 27

14 (X = O), see reference 28

14 (X = NH), see reference 29

16 (X = S), see reference 30

17 (X = S), see reference 31

18 (X = O), see reference 32

18 (X = NH), see reference 33

18 (X = S), see reference 34

28 (X = NH), see reference 35

Table S2. MCI and HOMA values for the 214 compounds computed at the M062X/6-311G(d,p).

		MCI_{S0}	MCI_{T1}	$HOMA_{S0}$	$HOMA_{T1}$
**1**	X = NH X’ = NH	0.0046	0.0281	0.1811	0.8574
**1**	X = NH X’ = O	0.0041	0.0129	-0.3562	0.1711
**1**	X = NH X’ = S	0.0041	0.0296	0.2030	0.9013
**1**	X = O X’ = O	0.0039	0.0049	-0.8018	-0.4415
**1**	X = O X’ = S	0.0040	0.0087	-0.3437	0.1246
**1**	X = S X’ = S	0.0057	0.0390	0.0978	0.9546
**2**	X = NH X' = NH X" = NH	0.0029	0.0080	0.3722	0.5003
**2**	X = NH X' = NH X" = O	0.0027	0.0064	-0.1030	-0.0339
**2**	X = NH X' = NH X" = S	0.0025	0.0076	0.3858	0.5678
**2**	X = NH X' = O X" = NH	0.0026	0.0042	-0.1024	-0.0365
**2**	X = NH X' = O X" = O	0.0027	0.0027	-0.5630	-0.5244
**2**	X = NH X' = O X" = S	0.0025	0.0026	-0.0518	0.0641
**2**	X = NH X' = S X" = NH	0.0026	0.0077	0.3741	0.5781
**2**	X = NH X' = S X" = O	0.0025	0.0036	-0.0459	0.0790
**2**	X = NH X' = S X" = S	0.0031	0.0049	0.2883	0.5730
**2**	X = o X' = NH X" = NH	0.0032	0.0100	0.3538	0.5256
**2**	X = O X' = NH X" = O	0.0029	0.0071	-0.1261	-0.0104
**2**	X = O X' = NH X" = S	0.0027	0.0089	0.3576	0.5809
**2**	X = O X' = O X" = NH	0.0030	0.0042	-0.1269	-0.0214
**2**	X = O X' = O X" = O	0.0030	0.0022	-0.5817	-0.6918
**2**	X = O X' = O X" = S	0.0027	0.0034	-0.0748	0.0442
**2**	X = O X' = S X" = NH	0.0028	0.0093	0.3447	0.5974
**2**	X = O X' = S X" = O	0.0028	0.0042	-0.0696	0.0803
**2**	X = O X' = S X" = S	0.0034	0.0054	0.2630	0.5591
**2**	X = S X' = NH X" = NH	0.0034	0.0103	0.3557	0.5989
**2**	X = S X' = NH X" = O	0.0031	0.0051	-0.1409	-0.0075
**2**	X = S X' = NH X" = S	0.0029	0.0090	0.3688	0.5213
**2**	X = S X' = O X" = NH	0.0031	0.0053	-0.1262	0.0849
**2**	X = S X' = O X" = O	0.0031	0.0027	-0.5905	-0.5891
**2**	X = S X' = O X" = S	0.0028	0.0044	-0.1272	0.0098
**2**	X = S X' = S X" = NH	0.0030	0.0094	0.3430	0.5174
**2**	X = S X' = S X" = O	0.0030	0.0044	-0.1453	-0.1181
**2**	X = S X' = S X" = S	0.0037	0.0053	0.2529	0.5553
**3**	X = NH X' = NH X" = NH	0.0018	0.0039	0.4776	0.4082
**3**	X = NH X' = NH X" = O	0.0018	0.0021	0.0767	-0.3285
**3**	X = NH X' = NH X" = S	0.0015	0.0091	0.5192	-0.3232
**3**	X = NH X' = O X" = NH	0.0017	0.0016	0.0396	-0.2502
**3**	X = NH X' = O X" = O	0.0019	0.0014	-0.3843	-0.8234
**3**	X = NH X' = O X" = S	0.0016	0.0041	0.1048	-
**3**	X = NH X' = S X" = NH	0.0015	0.0035	0.4910	0.2963
**3**	X = NH X' = S X" = O	0.0017	0.0014	0.1362	-0.4707
**3**	X = NH X' = S X" = S	0.0017	0.0086	0.4607	-

**3**	X = O X' = NH X" = NH	0.0023	0.0042	0.4817	0.3785
**3**	X = O X' = NH X" = O	0.0021	0.0079	0.1008	0.4384
**3**	X = O X' = NH X" = S	0.0018	0.0080	0.5014	-
**3**	X = O X' = O X" = NH	0.0021	0.0024	0.0518	-0.1433
**3**	X = O X' = O X" = O	0.0022	0.0015	-0.3482	-0.8054
**3**	X = O X' = O X" = S	0.0019	0.0021	0.1071	-0.2751
**3**	X = O X' = S X" = NH	0.0021	0.0038	0.4915	0.2998
**3**	X = O X' = S X" = O	0.0021	0.0020	0.1470	-0.2786
**3**	X = O X' = S X" = S	0.0019	0.0033	0.4422	0.1587
**3**	X = S X' = NH X" = NH	0.0024	0.0059	0.4979	0.5582
**3**	X = S X' = NH X" = O	0.0023	0.0028	0.0546	-0.0588
**3**	X = S X' = NH X" = S	0.0021	0.0045	0.5192	0.4456
**3**	X = S X' = O X" = NH	0.0022	0.0030	0.0453	0.0586
**3**	X = S X' = O X" = O	0.0024	0.0065	-0.3892	0.1263
**3**	X = S X' = O X" = S	0.0020	0.0024	0.0438	-0.0036
**3**	X = S X' = S X" = NH	0.0021	0.0049	0.4637	0.4552
**3**	X = S X' = S X" = O	0.0022	0.0024	0.0343	-0.1297
**3**	X = S X' = S X" = S	0.0024	0.0038	0.4115	0.3555
**4**	X = NH X' = NH X" = NH	0.0018	0.0037	0.4747	0.4081
**4**	X = NH X' = NH X" = O	0.0017	0.0023	0.0531	-0.0478
**4**	X = NH X' = O X" = O	0.0018	0.0014	-0.3961	-0.7619
**4**	X = NH X' = S X" = NH	0.0015	0.0032	0.4994	0.2941
**4**	X = NH X' = S X" = O	0.0016	0.0016	0.1205	-0.3326
**4**	X = NH X' = S X" = S	0.0017	0.0082	0.4640	-
**4**	X = O X' = NH X" = NH	0.0022	0.0041	0.4855	0.3820
**4**	X = O X' = NH X" = O	0.0021	0.0024	0.0732	-0.0817
**4**	X = O X' = O X" = O	0.0022	0.0015	-0.3578	-0.7598
**4**	X = O X' = S X" = NH	0.0020	0.0035	0.5009	0.2908
**4**	X = O X' = S X" = O	0.0020	0.0022	0.1297	-0.1467
**4**	X = O X' = S X" = S	0.0019	0.0031	0.4485	0.2047
**4**	X = S X' = NH X" = NH	0.0024	0.0059	0.4920	0.5658
**4**	X = S X' = NH X" = O	0.0023	0.0030	0.0473	0.0807
**4**	X = S X' = O X" = O	0.0024	0.0066	-0.3949	0.1756
**4**	X = S X' = S X" = NH	0.0021	0.0047	0.4978	0.4800
**4**	X = S X' = S X" = O	0.0021	0.0025	0.0351	0.0311
**4**	X = S X' = S X" = S	0.0024	0.0038	0.4093	0.3730
**5**	X = NH X' = NH	0.0033	0.0133	0.4258	0.7195
**5**	X = NH X' = O	0.0028	0.0034	-0.1020	0.0017
**5**	X = NH X' = S	0.0028	0.0045	0.3667	0.5401
**5**	X = O X' = O	0.0026	0.0037	-0.5268	-0.3805
**5**	X = O X' = S	0.0026	0.0022	-0.1504	0.0139
**5**	X = S X' = S	0.0033	0.0035	0.2288	0.4523
**6**	X = NH X' = NH	0.0020	0.0079	0.6627	0.5809
**6**	X = NH X' = O	0.0017	0.0025	0.1509	-0.0057
**6**	X = NH X' = S	0.0017	0.0047	0.5141	0.4792
**6**	X = O X' = O	0.0017	0.0052	-0.2672	0.0828

**6**	X = O X' = S	0.0016	0.0022	0.0099	0.0423
**6**	X = S X' = S	0.0020	0.0107	0.3376	0.8019
**7**	X = NH X' = NH X" = NH	0.0019	0.0069	0.5395	0.5653
**7**	X = NH X' = NH X" = O	0.0021	0.0025	0.5073	0.4286
**7**	X = NH X' = NH X" = S	0.0023	0.0033	0.5568	0.5089
**7**	X = NH X' = O X" = NH	0.0017	0.0048	0.0647	0.1213
**7**	X = NH X' = O X" = O	0.0019	0.0059	0.0359	-0.0762
**7**	X = NH X' = O X" = S	0.0020	0.0019	0.0750	0.0272
**7**	X = NH X' = S X" = NH	0.0017	0.0071	0.4943	0.4890
**7**	X = NH X' = S X" = O	0.0019	0.0080	0.4642	0.5006
**7**	X = NH X' = S X" = S	0.0020	0.0029	0.4834	0.3766
**7**	X = O X' = NH X" = NH	0.0018	0.0045	0.0790	0.1482
**7**	X = O X' = NH X" = O	0.0019	0.0053	0.0533	0.1580
**7**	X = O X' = NH X" = S	0.0020	0.0017	0.0667	-0.0874
**7**	X = O X' = O X" = NH	0.0018	0.0044	-0.3665	-0.7962
**7**	X = O X' = O X" = O	0.0019	0.0027	-0.3934	-0.4677
**7**	X = O X' = O X" = S	0.0020	0.0012	-0.3582	-0.5617
**7**	X = O X' = S X" = NH	0.0016	0.0045	0.0397	0.1399
**7**	X = O X' = S X" = O	0.0018	0.0047	0.0249	0.1414
**7**	X = O X' = S X" = S	0.0019	0.0043	0.0013	0.1898
**7**	X = S X' = NH X" = NH	0.0017	0.0073	0.4920	0.4293
**7**	X = S X' = NH X" = O	0.0018	0.0082	0.4648	0.4451
**7**	X = S X' = NH X" = S	0.0019	0.0027	0.4996	0.3905
**7**	X = S X' = O X" = NH	0.0016	0.0049	0.0195	0.0979
**7**	X = S X' = O X" = O	0.0018	0.0047	0.0032	0.0903
**7**	X = S X' = O X" = S	0.0018	0.0041	0.0088	0.1484
**7**	X = S X' = S X" = NH	0.0020	0.0072	0.3726	0.5094
**7**	X = S X' = S X" = O	0.0021	0.0076	0.3493	0.5080
**7**	X = S X' = S X" = S	0.0023	0.0026	0.3581	0.2813
**8**	X = NH X' = NH	0.0026	0.0106	0.3130	0.8289
**8**	X = NH X' = O	0.0029	0.0128	0.2986	0.8800
**8**	X = NH X' = S	0.0028	0.0134	0.2731	0.8766
**8**	X = O X' = NH	0.0019	0.0061	-0.1546	0.5469
**8**	X = O X' = S	0.0021	0.0079	-0.2145	0.6031
**8**	X = O X' = O	0.0020	0.0076	-0.1837	0.6107
**8**	X = S X' = NH	0.0017	0.0102	0.2545	0.7967
**8**	X = S X' = O	0.0018	0.0132	0.2217	0.8567
**8**	X = S X' = S	0.0019	0.0134	0.1948	0.8496
**9**	X = NH X' = NH	0.0016	0.0113	0.2718	0.8118
**9**	X = NH X' = O	0.0017	0.0127	0.2554	0.8514
**9**	X = NH X' = S	0.0026	0.0129	0.2965	0.8455
**9**	X = O X' = NH	0.0019	0.0068	-0.1366	0.4969
**9**	X = O X' = O	0.0021	0.0075	-0.1409	0.5359
**9**	X = O X' = S	0.0020	0.0076	-0.1809	0.5329
**9**	X = S X' = NH	0.0017	0.0111	0.2622	0.7838
**9**	X = S X' = O	0.0019	0.0127	0.2526	0.8261

**9**	X = S X' = S	0.0018	0.0127	0.2103	0.8144
**10**	X = NH X' = NH	0.0043	0.0107	0.3652	0.8412
**10**	X = NH X' = O	0.0041	0.0128	0.3417	0.8845
**10**	X = NH X' = S	0.0038	0.0126	0.3565	0.8533
**10**	X = O X' = NH	0.0033	0.0065	-0.2115	0.5265
**10**	X = O X' = O	0.0030	0.0079	-0.2355	0.5750
**10**	X = O X' = S	0.0028	0.0076	-0.2504	0.5551
**10**	X = S X' = NH	0.0031	0.0102	0.1854	0.8179
**10**	X = S X' = O	0.0027	0.0128	0.1835	0.8612
**10**	X = S X' = S	0.0026	0.0122	0.1566	0.8322
**11**	X = NH X' = NH	0.0016	0.0122	0.2278	0.8007
**11**	X = NH X' = O	0.0016	0.0137	0.2342	0.8560
**11**	X = NH X' = S	0.0016	0.0136	0.1769	0.8201
**11**	X = O X' = NH	0.0017	0.0075	-0.2267	0.4889
**11**	X = O X' = O	0.0019	0.0084	-0.2045	0.5371
**11**	X = O X' = S	0.0018	0.0085	-0.2576	0.5131
**11**	X = S X' = NH	0.0018	0.0128	0.2063	0.7656
**11**	X = S X' = O	0.0020	0.0145	0.2164	0.8239
**11**	X = S X' = S	0.0018	0.0147	0.1389	0.7944
**12**	X = NH X' = NH	0.0017	0.0061	0.4055	0.7428
**12**	X = NH X' = O	0.0013	0.0088	0.3228	0.8583
**12**	X = NH X' = S	0.0016	0.0096	0.3185	0.8450
**12**	X = O X' = NH	0.0012	0.0030	0.0322	0.4743
**12**	X = O X' = O	0.0014	0.0048	0.0334	0.6392
**12**	X = O X' = S	0.0015	0.0052	-0.0560	0.6068
**12**	X = S X' = NH	0.0011	0.0041	0.3728	0.6279
**12**	X = S X' = O	0.0013	0.0085	0.3535	0.8141
**12**	X = S X' = S	0.0014	0.0077	0.3051	0.7652
**13**	X = S	0.0026	0.0213	0.0974	0.8856
**13**	X = NH	0.0037	0.0196	0.2635	0.9054
**13**	X = O	0.0029	0.0125	-0.3118	0.5933
**14**	X = NH	0.0016	0.0101	0.1704	0.7706
**14**	X = O	0.0016	0.0059	-0.2746	0.4642
**14**	X = S	0.0015	0.0097	0.0934	0.7262
**15**	X = NH	0.0008	0.0094	0.2270	0.7520
**15**	X = O	0.0000	0.0056	-1.0831	0.4631
**15**	X = S	0.0026	0.0089	0.2759	0.7136
**16**	X = NH	0.0014	0.0125	0.1299	0.7490
**16**	X = O	0.0013	0.0086	-0.2357	0.5050
**16**	X = S	0.0013	0.0145	-0.0634	0.7468
**17**	X = NH	0.0009	0.0065	0.1513	0.6688
**17**	X = O	0.0009	0.0032	-0.1970	0.3594
**17**	X = S	0.0008	0.0048	0.1021	0.5668
**18**	X = NH	0.0009	0.0063	0.0880	0.5236
**18**	X = O	0.0007	0.0036	-0.3842	0.2431
**18**	X = S	0.0007	0.0040	-0.0786	0.3817

**19**	X = NH	0.0016	0.0222	0.0136	0.9358
**19**	X = O	0.0021	0.0144	-0.0877	0.6624
**19**	X = S	0.0020	0.0227	-	-
**20**	X = NH	0.0016	0.0189	0.2724	0.9372
**20**	X = O	0.0026	0.0121	-0.1943	0.6563
**20**	X = S	0.0020	0.0208	0.1656	0.9291
**21**	X = NH	0.0009	0.0123	0.2514	0.7172
**21**	X = O	0.0011	0.0083	-0.1624	0.4140
**21**	X = S	0.0010	0.0136	0.1306	0.6721
**22**	X = NH	0.0014	0.0098	0.3794	0.7553
**22**	X = O	0.0010	0.0057	-0.0892	0.4194
**22**	X = S	0.0008	0.0087	0.2193	0.6881
**23**	COT	0.0010	0.0275	-0.3307	0.9580
**24**	X = NH	0.0008	0.0149	-0.1253	0.8453
**24**	X = O	0.0008	0.0179	-0.1411	0.9114
**24**	X = S	0.0008	0.0172	-0.2087	0.8789
**25**	X = N X' = N	0.0007	0.0103	0.0619	0.7396
**25**	X = N X' = O	0.0007	0.0114	0.0609	0.7995
**25**	X = N X' = S	0.0007	0.0113	-0.0146	0.7823
**25**	X = O X' = O	0.0007	0.0131	0.0482	0.8695
**25**	X = O X' = S	0.0007	0.0129	-0.0247	0.8434
**25**	X = S X' = S	0.0007	0.0127	-0.0952	0.8252
**26**	X = N X' = N	0.0007	0.0100	0.0589	0.7248
**26**	X = N X' = O	0.0007	0.0110	0.0570	0.7771
**26**	X = N X' = S	0.0007	0.0110	-0.0147	0.7608
**26**	X = O X' = O	0.0007	0.0125	0.0439	0.8368
**26**	X = O X' = S	0.0007	0.0123	-0.0261	0.8131
**26**	X = S X' = S	0.0007	0.0123	-0.0953	0.7982
**27**	X = NH	0.0007	0.0163	-0.1296	0.8839
**27**	X = O	0.0008	0.0189	-0.1345	0.9362
**27**	X = S	0.0008	0.0181	-0.2015	0.8975
**28**	X = NH	0.0007	0.0138	-0.1339	0.8563
**28**	X = O	0.0008	0.0173	-0.1496	0.9288
**28**	X = S	0.0008	0.0167	-0.1990	0.8973

Table S3. Absolute values of the various dihedral angles in the S_0 and T_1 states (in degrees), inversion barrier in the S_0 state (kJ/mol), and vertical excitation energies from the planar transition state in the S_0 state (kJ/mol).

		λ	ϕ_{S0}	ϕ_T	$\Delta G^{\ddagger}_{invers}$	$\Delta E_{S0=>T1vert}$ (TS)
**1**	X = NH X’ = NH	206.7	17.5	2.0	5.9	269.5
**1**	X = NH X’ = O	221.3	12.8	18.7	-	-
**1**	X = NH X’ = S	198.1	24.9	4.2	8.5	262.4
**1**	X = O X’ = O	220.6	0.0	23.8	-	-
**1**	X = O X’ = S	253.5	19.7	24.3	-	-
**1**	X = S X’ = S	223.7	26.1	0.0	19.4	283.8
**2**	X = NH X' = NH X" = NH	280.8	6.3	16.9	-	-
**2**	X = NH X' = NH X" = O	243.6	4.8	23.1	-	-
**2**	X = NH X' = NH X" = S	228.6	20.2	20.4	-	-
**2**	X = NH X' = O X" = NH	288.9	2.0	19.4	-	-
**2**	X = NH X' = O X" = O	240.4	0.0	24.9	-	-
**2**	X = NH X' = O X" = S	279.5	4.7	23.9	-	-
**2**	X = NH X' = S X" = NH	234.8	19.0	20.2	-	-
**2**	X = NH X' = S X" = O	257.6	0.0	24.5	-	-
**2**	X = NH X' = S X" = S	283.5	24.9	26.4	-	-
**2**	X = o X' = NH X" = NH	259.5	6.1	15.9	-	-
**2**	X = O X' = NH X" = O	238.7	4.2	22.4	-	-
**2**	X = O X' = NH X" = S	224.0	18.4	20.1	-	-
**2**	X = O X' = O X" = NH	237.0	2.5	19.4	-	-
**2**	X = O X' = O X" = O	244.6	0.0	22.1	-	-
**2**	X = O X' = O X" = S	269.6	0.0	25.0	-	-
**2**	X = O X' = S X" = NH	227.1	18.1	19.7	-	-
**2**	X = O X' = S X" = O	249.9	0.0	24.8	-	-
**2**	X = O X' = S X" = S	289.5	23.7	26.4	-	-
**2**	X = S X' = NH X" = NH	198.4	9.1	14.9	-	-
**2**	X = S X' = NH X" = O	195.0	7.8	18.6	-	-
**2**	X = S X' = NH X" = S	183.6	23.2	19.4	-	-
**2**	X = S X' = O X" = NH	197.6	2.9	17.7	-	-
**2**	X = S X' = O X" = O	205.8	0.0	20.8	-	-
**2**	X = S X' = O X" = S	188.4	16.4	22.1	-	-
**2**	X = S X' = S X" = NH	208.0	23.2	19.4	-	-
**2**	X = S X' = S X" = O	224.8	17.0	23.4	-	-
**2**	X = S X' = S X" = S	299.6	26.2	26.2	-	-
**3**	X = NH X' = NH X" = NH	314.7	0.9	18.3	-	-
**3**	X = NH X' = NH X" = O	348.3	1.1	20.3	-	-
**3**	X = NH X' = NH X" = S	299.6	17.8	0.0	5.7	310.9
**3**	X = NH X' = O X" = NH	310.8	3.2	22.3	-	-
**3**	X = NH X' = O X" = O	346.0	0.0	21.5	-	-
**3**	X = NH X' = O X" = S	316.8	6.5	17.0	-	-
**3**	X = NH X' = S X" = NH	331.2	12.1	22.8	-	-

**3**	X = NH X' = S X" = O	256.8	0.0	26.4	-	-
**3**	X = NH X' = S X" = S	410.1	25.6	24.4	-	-
**3**	X = O X' = NH X" = NH	283.4	1.2	18.6	-	-
**3**	X = O X' = NH X" = O	187.4	1.3	15.8	-	-
**3**	X = O X' = NH X" = S	385.7	7.9	0.0	245.6	28.9
**3**	X = O X' = O X" = NH	284.6	2.2	19.9	-	-
**3**	X = O X' = O X" = O	286.6	0.0	22.2	-	-
**3**	X = O X' = O X" = S	228.1	0.0	24.8	-	-
**3**	X = O X' = S X" = NH	257.4	3.8	22.2	-	-
**3**	X = O X' = S X" = O	267.2	0.0	24.3	-	-
**3**	X = O X' = S X" = S	263.5	21.5	27.1	-	-
**3**	X = S X' = NH X" = NH	222.3	1.2	16.2	-	-
**3**	X = S X' = NH X" = O	212.7	4.0	20.0	-	-
**3**	X = S X' = NH X" = S	193.8	23.5	21.8	-	-
**3**	X = S X' = O X" = NH	236.8	5.1	18.8	-	-
**3**	X = S X' = O X" = O	137.5	0.0	19.5	-	-
**3**	X = S X' = O X" = S	214.0	18.8	23.8	-	-
**3**	X = S X' = S X" = NH	240.3	16.2	21.6	-	-
**3**	X = S X' = S X" = O	250.2	18.0	24.6	-	-
**3**	X = S X' = S X" = S	262.8	27.7	26.5	-	-
**4**	X = NH X' = NH X" = NH	322.1	1.4	18.9	-	-
**4**	X = NH X' = NH X" = O	284.5	1.6	20.2	-	-
**4**	X = NH X' = O X" = O	297.3	0.0	21.5	-	-
**4**	X = NH X' = S X" = NH	248.4	9.4	23.6	-	-
**4**	X = NH X' = S X" = O	259.4	4.3	25.1	-	-
**4**	X = NH X' = S X" = S	374.8	25.8	26.3	-	-
**4**	X = O X' = NH X" = NH	276.2	1.8	19.3	-	-
**4**	X = O X' = NH X" = O	250.7	1.2	20.6	-	-
**4**	X = O X' = O X" = O	282.6	0.0	22.2	-	-
**4**	X = O X' = S X" = NH	227.9	4.2	23.0	-	-
**4**	X = O X' = S X" = O	205.8	0.0	24.4	-	-
**4**	X = O X' = S X" = S	262.2	22.5	27.0	-	-
**4**	X = S X' = NH X" = NH	214.4	1.3	16.7	-	-
**4**	X = S X' = NH X" = O	207.5	2.1	19.0	-	-
**4**	X = S X' = O X" = O	125.6	0.0	18.5	-	-
**4**	X = S X' = S X" = NH	176.5	23.4	21.2	-	-
**4**	X = S X' = S X" = O	192.8	18.4	23.2	-	-
**4**	X = S X' = S X" = S	260.9	27.4	26.5	-	-
**5**	X = NH X' = NH	150.4	19.5	0.1	3.6	280.0
**5**	X = NH X' = O	212.4	14.3	22.6	-	-
**5**	X = NH X' = S	234.7	25.0	20.9	-	-
**5**	X = O X' = O	179.0	0.0	18.3	-	-
**5**	X = O X' = S	322.1	21.7	26.1	-	-
**5**	X = S X' = S	398.2	27.8	26.6	-	-
**6**	X = NH X' = NH	110.2	18.5	0.0	1.2	296.5
**6**	X = NH X' = O	147.7	15.2	17.1	-	-

**6**	X = NH X' = S	155.2	26.3	17.7	-	-
**6**	X = O X' = O	77.2	0.0	5.2	0.3	398.9
**6**	X = O X' = S	203.1	25.2	23.2	-	-
**6**	X = S X' = S	144.0	29.8	0.5	28.1	342.7
**7**	X = NH X' = NH X'' = NH	141.1	10.0	12.1	-	-
**7**	X = NH X' = NH X'' = O	205.9	8.3	19.4	-	-
**7**	X = NH X' = NH X'' = S	217.5	17.4	16.7	-	-
**7**	X = NH X' = O X'' = NH	155.6	1.9	14.5	-	-
**7**	X = NH X' = O X'' = O	178.9	2.4	17.0	-	-
**7**	X = NH X' = O X'' = S	243.0	6.5	19.4	-	-
**7**	X = NH X' = S X'' = NH	151.3	23.5	19.3	-	-
**7**	X = NH X' = S X'' = O	150.1	22.0	18.8	-	-
**7**	X = NH X' = S X'' = S	220.9	25.6	23.0	-	-
**7**	X = O X' = NH X'' = NH	147.4	7.4	15.5	-	-
**7**	X = O X' = NH X'' = O	145.5	5.7	15.2	-	-
**7**	X = O X' = NH X'' = S	194.7	12.9	20.7	-	-
**7**	X = O X' = O X'' = NH	184.9	0.0	19.4	-	-
**7**	X = O X' = O X'' = O	178.9	0.0	19.2	-	-
**7**	X = O X' = O X'' = S	277.6	0.0	22.2	-	-
**7**	X = O X' = S X'' = NH	157.2	19.3	21.1	-	-
**7**	X = O X' = S X'' = O	156.9	17.3	21.1	-	-
**7**	X = O X' = S X'' = S	165.5	22.5	20.6	-	-
**7**	X = S X' = NH X'' = NH	157.0	24.0	19.9	-	-
**7**	X = S X' = NH X'' = O	155.7	22.4	19.1	-	-
**7**	X = S X' = NH X'' = S	210.7	25.5	21.9	-	-
**7**	X = S X' = O X'' = NH	162.5	20.9	21.4	-	-
**7**	X = S X' = O X'' = O	159.8	18.1	21.0	-	-
**7**	X = S X' = O X'' = S	169.3	22.6	21.3	-	-
**7**	X = S X' = S X'' = NH	222.1	28.4	24.6	-	-
**7**	X = S X' = S X'' = O	228.7	27.5	24.2	-	-
**7**	X = S X' = S X'' = S	315.6	28.9	27.0	-	-
**8**	X = NH X' = NH	257.1	27.1	0.0	-0.8	201.0
**8**	X = NH X' = O	271.8	25.9	0.0	-6.6	176.4
**8**	X = NH X' = S	282.3	29.3	0.0	-1.0	167.5
**8**	X = O X' = NH	198.0	25.6	0.0	10.0	222.2
**8**	X = O X' = S	229.2	28.4	0.0	18.4	211.4
**8**	X = O X' = O	216.2	25.8	0.0	11.7	217.2
**8**	X = S X' = NH	228.1	29.1	3.8	31.2	208.9
**8**	X = S X' = O	247.4	28.7	0.0	29.9	202.5
**8**	X = S X' = S	249.6	29.7	0.0	37.6	196.9
**9**	X = NH X' = NH	170.6	20.8	0.0	3.3	168.6
**9**	X = NH X' = O	182.2	20.8	0.0	17.5	271.1
**9**	X = NH X' = S	276.1	30.5	0.0	-10.6	160.0
**9**	X = O X' = NH	193.0	26.2	0.0	9.5	208.8
**9**	X = O X' = O	203.1	25.6	0.0	9.2	201.3
**9**	X = O X' = S	218.9	28.3	0.0	14.9	203.8

**9**	X = S X' = NH	214.4	28.7	0.0	25.5	195.4
**9**	X = S X' = O	227.1	28.5	0.0	24.3	187.4
**9**	X = S X' = S	238.4	29.4	0.0	31.3	189.5
**10**	X = NH X' = NH	275.6	33.7	0.0	45.9	29.3
**10**	X = NH X' = O	266.4	32.7	0.0	-2.9	90.8
**10**	X = NH X' = S	311.0	33.6	0.0	60.0	15.9
**10**	X = O X' = NH	219.5	33.6	0.0	28.7	111.2
**10**	X = O X' = O	213.5	32.0	0.0	18.6	129.2
**10**	X = O X' = S	219.2	32.2	0.0	22.5	125.1
**10**	X = S X' = NH	269.2	31.5	0.0	39.8	93.1
**10**	X = S X' = O	216.3	30.8	0.0	29.8	116.2
**10**	X = S X' = S	225.9	31.0	0.0	35.1	111.6
**11**	X = NH X' = NH	162.9	19.3	0.0	3.4	192.8
**11**	X = NH X' = O	178.5	20.9	0.0	4.2	175.2
**11**	X = NH X' = S	170.0	20.6	0.0	4.4	171.7
**11**	X = O X' = NH	205.5	29.1	0.0	11.6	231.7
**11**	X = O X' = O	218.1	29.8	0.0	13.8	214.7
**11**	X = O X' = S	211.7	29.1	0.0	12.3	214.0
**11**	X = S X' = NH	211.0	28.9	0.0	22.0	215.4
**11**	X = S X' = O	225.3	29.1	0.0	23.1	198.4
**11**	X = S X' = S	231.8	29.1	0.0	24.6	197.5
**12**	X = NH X' = NH	200.1	20.4	0.1	10.5	281.5
**12**	X = NH X' = O	163.9	17.7	0.0	5.7	230.3
**12**	X = NH X' = S	202.4	25.6	0.0	18.4	199.8
**12**	X = O X' = NH	152.4	19.8	0.0	6.7	268.1
**12**	X = O X' = O	171.6	19.3	0.0	6.9	251.0
**12**	X = O X' = S	201.0	26.6	0.0	19.9	231.5
**12**	X = S X' = NH	189.4	27.4	18.2	-	-
**12**	X = S X' = O	219.4	26.7	6.6	27.0	240.2
**12**	X = S X' = S	214.0	29.3	16.3	-	-
**13**	X = S	281.4	29.7	0.0	35.7	171.8
**13**	X = NH	321.9	32.7	0.0	-7.9	145.3
**13**	X = O	266.6	30.6	0.0	20.9	191.0
**14**	X = NH	192.5	26.0	0.0	9.0	186.8
**14**	X = O	220.4	29.9	0.0	19.9	226.4
**14**	X = S	242.1	30.5	0.1	36.6	211.4
**15**	X = NH	78.1	4.1	0.0	5.7	43.3
**15**	X = O	383.4	50.2	0.0	132.5	91.7
**15**	X = S	245.6	33.1	0.0	71.6	44.9
**16**	X = NH	112.9	7.9	0.0	4.1	207.1
**16**	X = O	123.2	3.8	0.1	6.9	257.5
**16**	X = S	237.8	27.8	0.0	20.4	231.6
**17**	X = NH	223.6	31.3	0.0	-4.9	226.7
**17**	X = O	189.0	30.4	0.0	26.3	262.3
**17**	X = S	200.0	31.1	14.6	-	-
**18**	X = NH	175.7	29.5	9.5	31.1	232.8

**18**	X = O	201.2	30.6	10.8	-	-
**18**	X = S	204.6	32.6	18.6	-	-
**19**	X = NH	254.3	30.8	0.0	23.6	138.0
**19**	X = O	265.3	34.9	0.0	41.4	192.0
**19**	X = S	244.0	30.8	0.0	27.5	169.0
**20**	X = NH	235.8	26.5	0.0	10.2	153.7
**20**	X = O	280.4	28.4	0.0	17.3	207.1
**20**	X = S	291.9	30.0	0.0	25.5	187.0
**21**	X = NH	331.4	33.6	0.0	-37.5	229.0
**21**	X = O	216.8	27.4	0.0	8.4	272.4
**21**	X = S	252.6	30.4	0.0	28.7	245.5
**22**	X = NH	208.0	26.0	0.0	8.4	220.1
**22**	X = O	226.2	31.1	0.1	20.7	251.0
**22**	X = S	246.0	31.7	0.3	41.0	232.7
**23**	COT	359.7	28.2	0.0	65.5	122.2
**24**	X = NH	282.5	27.4	0.0	36.1	147.3
**24**	X = O	297.1	27.6	0.0	40.5	140.3
**24**	X = S	315.9	28.2	0.0	49.7	144.8
**25**	X = N X' = N	204.0	23.1	0.0	19.5	188.2
**25**	X = N X' = O	217.4	23.3	0.0	20.5	174.8
**25**	X = N X' = S	237.9	26.5	0.0	29.5	174.4
**25**	X = O X' = O	232.4	23.6	0.0	22.4	164.2
**25**	X = O X' = S	252.0	26.5	0.0	31.2	165.9
**25**	X = S X' = S	273.0	27.7	0.0	40.8	169.1
**26**	X = N X' = N	201.1	22.6	0.0	19.2	186.4
**26**	X = N X' = O	213.8	22.7	0.0	20.1	173.4
**26**	X = N X' = S	234.0	26.2	0.0	29.5	173.3
**26**	X = O X' = O	229.0	23.3	0.0	22.1	162.9
**26**	X = O X' = S	248.5	26.2	0.0	31.2	164.9
**26**	X = S X' = S	270.1	27.2	0.0	41.1	168.3
**27**	X = NH	292.4	27.5	0.0	38.3	147.6
**27**	X = O	302.2	27.6	0.0	40.1	141.4
**27**	X = S	320.8	28.3	0.0	52.2	145.2
**28**	X = NH	297.3	27.5	0.0	34.7	163.3
**28**	X = O	307.2	27.7	0.0	40.3	151.1
**28**	X = S	320.1	28.4	0.0	47.4	150.0

Table S4. E_{T1} (kJ/mol), SOC values (cm^{-1}), reorganization energies (kJ/mol), k_{ISC} and Gibbs free reaction energies (kJ/mol) for singlet oxygen formation [$\Delta G_R(^1O_2)$][a].

		E_{T1}	SOC	λ	k_{ISC}	$\Delta G_R(^1O_2)$
**1**	X = NH X' = NH	172.9	1.5	206.7	$1.03\cdot10^{06}$	-14.5
**1**	X = NH X' = O	215.6	4.3	221.3	$2.10\cdot10^{-01}$	-57.2
**1**	X = NH X' = S	199.8	8.0	198.1	$4.62\cdot10^{02}$	-41.4
**1**	X = O X' = O	248.9	4.7	220.6	$2.56\cdot10^{-07}$	-90.5
**1**	X = O X' = S	234.5	26.1	253.5	-	-76.1
**1**	X = S X' = S	222.5	4.6	223.7	$1.51\cdot10^{-02}$	-64.1
**2**	X = NH X' = NH X" = NH	218.7	3.6	280.8	$9.23\cdot10^{-03}$	-60.3
**2**	X = NH X' = NH X" = O	237.4	3.8	243.6	$2.42\cdot10^{-05}$	-79.0
**2**	X = NH X' = NH X" = S	227.3	38.1	228.6	-	-68.9
**2**	X = NH X' = O X" = NH	238.7	8.7	288.9	$2.85\cdot10^{-05}$	-80.3
**2**	X = NH X' = O X" = O	262.3	4.9	240.4	$1.45\cdot10^{-09}$	-103.9
**2**	X = NH X' = O X" = S	248.3	37.0	279.5	-	-89.9
**2**	X = NH X' = S X" = NH	224.4	39.6	234.8	-	-66.0
**2**	X = NH X' = S X" = O	245.8	21.1	257.6	-	-87.4
**2**	X = NH X' = S X" = S	227.7	44.9	283.5	-	-69.3
**2**	X = o X' = NH X" = NH	215.9	3.1	259.5	$4.46\cdot10^{-02}$	-57.5
**2**	X = O X' = NH X" = O	236.6	3.9	238.7	$3.52\cdot10^{-05}$	-78.2
**2**	X = O X' = NH X" = S	225.0	38.3	224.0	-	-66.6
**2**	X = O X' = O X" = NH	230.9	5.7	237.0	$7.50\cdot10^{-04}$	-72.4
**2**	X = O X' = O X" = O	243.4	10.5	244.6	-	-85.0
**2**	X = O X' = O X" = S	247.0	19.5	269.6	-	-88.5
**2**	X = O X' = S X" = NH	221.8	39.2	227.1	-	-63.4
**2**	X = O X' = S X" = O	245.5	23.0	249.9	-	-87.1
**2**	X = O X' = S X" = S	225.9	43.6	289.5	-	-67.5
**2**	X = S X' = NH X" = NH	177.8	9.2	198.4	$3.51\cdot10^{06}$	-19.4
**2**	X = S X' = NH X" = O	206.0	11.6	195.0	-	-47.6
**2**	X = S X' = NH X" = S	205.8	38.9	183.6	-	-47.4
**2**	X = S X' = O X" = NH	212.6	11.0	197.6	-	-54.2
**2**	X = S X' = O X" = O	237.5	14.2	205.8	-	-79.1
**2**	X = S X' = O X" = S	234.9	43.2	188.4	-	-76.5
**2**	X = S X' = S X" = NH	213.0	18.2	208.0	-	-54.5
**2**	X = S X' = S X" = O	235.6	20.2	224.8	-	-77.2
**2**	X = S X' = S X" = S	229.8	42.4	299.6	-	-71.4
**3**	X = NH X' = NH X" = NH	249.1	3.7	314.7	$4.45\cdot10^{-08}$	-90.7
**3**	X = NH X' = NH X" = O	252.5	7.8	348.3	$1.31\cdot10^{-08}$	-94.1
**3**	X = NH X' = NH X" = S	259.8	88.0	299.6	-	-101.4
**3**	X = NH X' = O X" = NH	284.6	5.5	310.8	$1.88\cdot10^{-13}$	-126.2
**3**	X = NH X' = O X" = O	273.7	8.1	346.0	$8.79\cdot10^{-12}$	-115.3
**3**	X = NH X' = O X" = S	239.1	116.1	316.8	-	-80.7
**3**	X = NH X' = S X" = NH	270.4	10.8	331.2	-	-112.0
**3**	X = NH X' = S X" = O	278.6	11.6	256.8	-	-120.2
**3**	X = NH X' = S X" = S	256.2	86.6	410.1	-	-97.8

**3**	X = O X' = NH X" = NH	226.8	5.3	283.4	$1.02\cdot10^{-03}$	-68.4
**3**	X = O X' = NH X" = O	308.3	5.1	187.4	$7.00\cdot10^{-21}$	-149.9
**3**	X = O X' = NH X" = S	202.9	126.2	385.7	-	-44.5
**3**	X = O X' = O X" = NH	250.7	6.2	284.6	$1.90\cdot10^{-07}$	-92.2
**3**	X = O X' = O X" = O	250.1	10.8	286.6	-	-91.7
**3**	X = O X' = O X" = S	253.9	51.1	228.1	-	-95.5
**3**	X = O X' = S X" = NH	245.3	8.8	257.4	$4.95\cdot10^{-06}$	-86.8
**3**	X = O X' = S X" = O	250.8	11.3	267.2	-	-92.4
**3**	X = O X' = S X" = S	249.4	51.2	263.5	-	-91.0
**3**	X = S X' = NH X" = NH	198.8	7.2	222.3	$4.18\cdot10^{02}$	-40.4
**3**	X = S X' = NH X" = O	217.4	10.2	212.7	-	-59.0
**3**	X = S X' = NH X" = S	217.9	39.4	193.8	-	-59.5
**3**	X = S X' = O X" = NH	228.7	10.0	236.8	$5.54\cdot10^{-03}$	-70.3
**3**	X = S X' = O X" = O	298.3	4.4	137.5	$5.24\cdot10^{-24}$	-139.9
**3**	X = S X' = O X" = S	242.7	42.9	214.0	-	-84.3
**3**	X = S X' = S X" = NH	225.1	19.1	240.3	-	-66.6
**3**	X = S X' = S X" = O	241.5	19.9	250.2	-	-83.1
**3**	X = S X' = S X" = S	249.1	3.7	262.8	-	-90.7
**4**	X = NH X' = NH X" = NH	250.2	4.4	322.1	$3.19\cdot10^{-08}$	-91.8
**4**	X = NH X' = NH X" = O	272.0	4.5	284.5	$2.55\cdot10^{-11}$	-113.6
**4**	X = NH X' = O X" = O	276.3	8.9	297.3	$1.54\cdot10^{-11}$	-117.9
**4**	X = NH X' = S X" = NH	269.1	11.3	248.4	-	-110.7
**4**	X = NH X' = S X" = O	281.6	13.1	259.4	-	-123.2
**4**	X = NH X' = S X" = S	271.5	108.3	374.8	-	-113.1
**4**	X = O X' = NH X" = NH	228.1	6.1	276.2	$1.10\cdot10^{-03}$	-69.6
**4**	X = O X' = NH X" = O	250.5	6.8	250.7	$3.82\cdot10^{-07}$	-92.1
**4**	X = O X' = O X" = O	254.2	11.5	282.6	-	-95.8
**4**	X = O X' = S X" = NH	248.0	8.5	227.9	$1.47\cdot10^{-06}$	-89.5
**4**	X = O X' = S X" = O	256.0	51.9	205.8	-	-97.5
**4**	X = O X' = S X" = S	250.9	50.8	262.2	-	-92.4
**4**	X = S X' = NH X" = NH	198.3	9.0	214.4	$9.07\cdot10^{02}$	-39.9
**4**	X = S X' = NH X" = O	228.1	11.4	207.5	-	-69.7
**4**	X = S X' = O X" = O	299.2	2.6	125.6	$7.39\cdot10^{-27}$	-140.7
**4**	X = S X' = S X" = NH	218.2	40.9	176.5	-	-59.8
**4**	X = S X' = S X" = O	243.4	44.7	192.8	-	-85.0
**4**	X = S X' = S X" = S	248.5	49.4	260.9	-	-90.0
**5**	X = NH X' = NH	210.5	1.4	150.4	$1.89\cdot10^{-02}$	-52.1
**5**	X = NH X' = O	242.4	4.2	212.4	$2.71\cdot10^{-06}$	-84.0
**5**	X = NH X' = S	233.2	34.6	234.7	-	-74.8
**5**	X = O X' = O	309.3	5.5	179.0	$1.01\cdot10^{-21}$	-150.9
**5**	X = O X' = S	249.5	19.6	322.1	-	-91.0
**5**	X = S X' = S	236.1	42.9	398.2	-	-77.7
**6**	X = NH X' = NH	251.6	1.2	110.2	$1.34\cdot10^{-16}$	-93.2
**6**	X = NH X' = O	294.7	5.1	147.7	$1.89\cdot10^{-21}$	-136.3
**6**	X = NH X' = S	278.8	30.3	155.2	-	-120.4
**6**	X = O X' = O	357.0	1.2	77.2	$1.57\cdot10^{-71}$	-198.6

**6**	X = O X' = S	305.3	41.1	203.1	-	-146.9
**6**	X = S X' = S	369.2	3.3	144.0	$7.16 \cdot 10^{-44}$	-210.8
**7**	X = NH X' = NH X" = NH	243.1	2.2	141.1	$6.65 \cdot 10^{-10}$	-84.6
**7**	X = NH X' = NH X" = O	220.1	5.6	205.9	$5.55 \cdot 10^{-02}$	-61.7
**7**	X = NH X' = NH X" = S	203.2	9.6	217.5	$1.48 \cdot 10^{02}$	-44.8
**7**	X = NH X' = O X" = NH	275.8	3.1	155.6	$3.17 \cdot 10^{-16}$	-117.4
**7**	X = NH X' = O X" = O	278.5	6.7	178.9	$2.04 \cdot 10^{-14}$	-120.1
**7**	X = NH X' = O X" = S	230.6	12.6	243.0	-	-72.2
**7**	X = NH X' = S X" = NH	257.4	43.0	151.3	-	-99.0
**7**	X = NH X' = S X" = O	258.8	41.3	150.1	-	-100.4
**7**	X = NH X' = S X" = S	235.9	20.8	220.9	-	-77.5
**7**	X = O X' = NH X" = NH	273.9	3.3	147.4	$1.68 \cdot 10^{-16}$	-115.4
**7**	X = O X' = NH X" = O	278.4	2.9	145.5	$5.84 \cdot 10^{-18}$	-120.0
**7**	X = O X' = NH X" = S	224.0	12.0	194.7	-	-65.5
**7**	X = O X' = O X" = NH	307.3	9.2	184.9	$2.51 \cdot 10^{-20}$	-148.9
**7**	X = O X' = O X" = O	315.3	7.0	178.9	$5.84 \cdot 10^{-23}$	-156.9
**7**	X = O X' = O X" = S	249.7	14.8	277.6	-	-91.2
**7**	X = O X' = S X" = NH	285.0	50.7	157.2	-	-126.6
**7**	X = O X' = S X" = O	288.7	48.2	156.9	-	-130.3
**7**	X = O X' = S X" = S	293.6	41.2	165.5	-	-135.1
**7**	X = S X' = NH X" = NH	258.5	45.1	157.0	-	-100.0
**7**	X = S X' = NH X" = O	260.9	43.1	155.7	-	-102.5
**7**	X = S X' = NH X" = S	228.0	38.0	210.7	-	-69.6
**7**	X = S X' = O X" = NH	287.5	51.7	162.5	-	-129.1
**7**	X = S X' = O X" = O	291.2	48.0	159.8	-	-132.8
**7**	X = S X' = O X" = S	294.0	40.2	169.3	-	-135.6
**7**	X = S X' = S X" = NH	284.3	53.3	222.1	-	-125.9
**7**	X = S X' = S X" = O	283.4	51.2	228.7	-	-125.0
**7**	X = S X' = S X" = S	256.1	48.9	315.6	-	-97.7
**8**	X = NH X' = NH	116.4	1.1	257.1	$1.43 \cdot 10^{12}$	42.0
**8**	X = NH X' = O	104.8	1.2	271.8	$1.16 \cdot 10^{13}$	53.6
**8**	X = NH X' = S	105.8	1.3	282.3	$4.48 \cdot 10^{12}$	52.7
**8**	X = O X' = NH	155.8	1.1	198.0	$1.85 \cdot 10^{08}$	2.6
**8**	X = O X' = S	153.1	1.2	229.2	$1.22 \cdot 10^{08}$	5.3
**8**	X = O X' = O	150.5	1.1	216.2	$5.13 \cdot 10^{08}$	7.9
**8**	X = S X' = NH	160.5	3.3	228.1	$7.51 \cdot 10^{07}$	-2.1
**8**	X = S X' = O	154.1	2.0	247.4	$7.40 \cdot 10^{07}$	4.3
**8**	X = S X' = S	156.7	2.2	249.6	$3.41 \cdot 10^{07}$	1.7
**9**	X = NH X' = NH	109.3	1.1	170.6	$7.58 \cdot 10^{15}$	49.1
**9**	X = NH X' = O	98.0	1.1	182.2	$1.23 \cdot 10^{17}$	60.4
**9**	X = NH X' = S	86.2	1.1	276.1	$1.19 \cdot 10^{15}$	72.2
**9**	X = O X' = NH	142.5	1.1	193.0	$2.45 \cdot 10^{10}$	15.9
**9**	X = O X' = O	131.9	1.1	203.1	$5.13 \cdot 10^{11}$	26.5
**9**	X = O X' = S	140.6	1.1	218.9	$1.10 \cdot 10^{10}$	17.8
**9**	X = S X' = NH	145.7	1.9	214.4	$8.48 \cdot 10^{09}$	12.7
**9**	X = S X' = O	134.4	1.9	227.1	$1.58 \cdot 10^{11}$	24.0

**9**	X = S X' = S	143.7	2.0	238.4	$4.07 \cdot 10^{09}$	14.8
**10**	X = NH X' = NH	25.7	1.0	275.6	$2.55 \cdot 10^{21}$	132.7
**10**	X = NH X' = O	29.3	1.1	266.4	$3.03 \cdot 10^{21}$	129.1
**10**	X = NH X' = S	28.0	1.1	311.0	$4.85 \cdot 10^{19}$	130.4
**10**	X = O X' = NH	65.5	1.0	219.5	$4.57 \cdot 10^{19}$	92.9
**10**	X = O X' = O	76.9	1.0	213.5	$3.71 \cdot 10^{18}$	81.5
**10**	X = O X' = S	77.6	0.9	219.2	$1.56 \cdot 10^{18}$	80.8
**10**	X = S X' = NH	63.6	0.4	269.2	$1.08 \cdot 10^{17}$	94.8
**10**	X = S X' = O	74.4	0.4	216.3	$8.73 \cdot 10^{17}$	84.0
**10**	X = S X' = S	75.7	0.4	225.9	$2.01 \cdot 10^{17}$	82.7
**11**	X = NH X' = NH	133.2	1.2	162.9	$3.00 \cdot 10^{12}$	25.2
**11**	X = NH X' = O	116.0	1.1	178.5	$5.33 \cdot 10^{14}$	42.4
**11**	X = NH X' = S	115.1	1.2	170.0	$1.39 \cdot 10^{15}$	43.3
**11**	X = O X' = NH	171.8	1.2	205.5	$4.46 \cdot 10^{05}$	-13.4
**11**	X = O X' = O	155.7	1.1	218.1	$7.35 \cdot 10^{07}$	2.7
**11**	X = O X' = S	155.8	1.2	211.7	$1.14 \cdot 10^{08}$	2.6
**11**	X = S X' = NH	165.4	2.4	211.0	$1.57 \cdot 10^{07}$	-7.0
**11**	X = S X' = O	148.7	2.3	225.3	$2.23 \cdot 10^{09}$	9.7
**11**	X = S X' = S	151.4	2.3	231.8	$6.55 \cdot 10^{08}$	7.0
**12**	X = NH X' = NH	177.1	1.1	200.1	$6.09 \cdot 10^{04}$	-18.6
**12**	X = NH X' = O	152.3	1.1	163.9	$1.81 \cdot 10^{09}$	6.1
**12**	X = NH X' = S	148.2	1.4	202.4	$3.52 \cdot 10^{09}$	10.2
**12**	X = O X' = NH	198.1	1.0	152.4	$4.36 \cdot 10^{00}$	-39.7
**12**	X = O X' = O	181.1	1.1	171.6	$1.60 \cdot 10^{04}$	-22.6
**12**	X = O X' = S	179.2	1.3	201.0	$3.94 \cdot 10^{04}$	-20.7
**12**	X = S X' = NH	215.5	13.6	189.4	-	-57.1
**12**	X = S X' = O	189.4	5.7	219.4	$9.79 \cdot 10^{03}$	-31.0
**12**	X = S X' = S	194.2	6.3	214.0	$2.21 \cdot 10^{03}$	-35.8
**13**	X = S	127.4	2.1	281.4	$2.75 \cdot 10^{10}$	31.0
**13**	X = NH	69.6	1.2	321.9	$1.12 \cdot 10^{15}$	88.9
**13**	X = O	130.3	1.1	266.6	$1.09 \cdot 10^{10}$	28.1
**14**	X = NH	131.1	1.1	192.5	$1.37 \cdot 10^{12}$	27.3
**14**	X = O	168.7	1.1	220.4	$6.70 \cdot 10^{05}$	-10.2
**14**	X = S	169.9	2.0	242.1	$5.28 \cdot 10^{05}$	-11.5
**15**	X = NH	4.9	1.0	78.1	$1.74 \cdot 10^{32}$	153.5
**15**	X = O	152.2	1.0	383.4	$8.95 \cdot 10^{02}$	6.2
**15**	X = S	55.6	0.3	245.6	$4.62 \cdot 10^{18}$	102.8
**16**	X = NH	151.6	1.2	112.9	$9.47 \cdot 10^{08}$	6.8
**16**	X = O	191.7	1.1	123.2	$6.83 \cdot 10^{00}$	-33.3
**16**	X = S	181.1	2.3	237.8	$1.77 \cdot 10^{04}$	-22.7
**17**	X = NH	157.9	1.0	223.6	$2.32 \cdot 10^{07}$	0.6
**17**	X = O	212.4	1.0	189.0	$3.15 \cdot 10^{-02}$	-54.0
**17**	X = S	223.2	7.5	200.0	$2.48 \cdot 10^{-02}$	-64.8
**18**	X = NH	205.2	1.2	175.7	$7.83 \cdot 10^{-01}$	-46.8
**18**	X = O	245.6	1.3	201.2	$4.22 \cdot 10^{-08}$	-87.2
**18**	X = S	250.0	5.6	204.6	$1.28 \cdot 10^{-07}$	-91.6

**19**	X = NH	92.8	1.2	254.3	$1.68\cdot10^{15}$	65.6
**19**	X = O	154.5	1.1	265.3	$5.83\cdot10^{06}$	4.0
**19**	X = S	115.3	2.5	244.0	$2.77\cdot10^{13}$	43.0
**20**	X = NH	89.8	1.3	235.8	$2.23\cdot10^{16}$	68.6
**20**	X = O	130.9	1.2	280.4	$3.62\cdot10^{09}$	27.5
**20**	X = S	130.2	2.3	291.9	$5.74\cdot10^{09}$	28.2
**21**	X = NH	122.8	1.2	331.4	$4.80\cdot10^{08}$	35.6
**21**	X = O	201.6	1.2	216.8	$4.32\cdot10^{00}$	-43.2
**21**	X = S	197.6	2.4	252.6	$2.57\cdot10^{01}$	-39.2
**22**	X = NH	153.4	1.2	208.0	$3.43\cdot10^{08}$	5.0
**22**	X = O	185.9	1.2	226.2	$1.26\cdot10^{03}$	-27.5
**22**	X = S	186.5	2.0	246.0	$1.32\cdot10^{03}$	-28.0
**23**	COT	106.3	1.0	359.7	$1.86\cdot10^{09}$	52.2
**24**	X = NH	108.4	1.0	282.5	$1.13\cdot10^{12}$	50.0
**24**	X = O	103.8	0.9	297.1	$1.09\cdot10^{12}$	54.6
**24**	X = S	117.0	0.9	315.9	$5.10\cdot10^{09}$	41.4
**25**	X = N X' = N	136.7	0.9	204.0	$7.83\cdot10^{10}$	21.7
**25**	X = N X' = O	124.0	0.9	217.4	$1.96\cdot10^{12}$	34.4
**25**	X = N X' = S	133.9	1.0	237.9	$2.25\cdot10^{10}$	24.5
**25**	X = O X' = O	114.0	0.9	232.4	$1.37\cdot10^{13}$	44.4
**25**	X = O X' = S	125.1	0.9	252.0	$1.08\cdot10^{11}$	33.3
**25**	X = S X' = S	138.0	0.9	273.0	$4.41\cdot10^{08}$	20.4
**26**	X = N X' = N	134.8	0.9	201.1	$1.70\cdot10^{11}$	23.6
**26**	X = N X' = O	122.2	0.9	213.8	$4.37\cdot10^{12}$	36.2
**26**	X = N X' = S	132.7	0.9	234.0	$4.02\cdot10^{10}$	25.7
**26**	X = O X' = O	112.3	0.9	229.0	$2.84\cdot10^{13}$	46.1
**26**	X = O X' = S	123.8	0.9	248.5	$2.03\cdot10^{11}$	34.6
**26**	X = S X' = S	136.7	0.9	270.1	$7.64\cdot10^{08}$	21.7
**27**	X = NH	109.0	1.0	292.4	$4.12\cdot10^{11}$	49.4
**27**	X = O	102.5	1.0	302.2	$1.03\cdot10^{12}$	55.9
**27**	X = S	118.6	1.0	320.8	$2.36\cdot10^{09}$	39.8
**28**	X = NH	118.2	1.0	297.3	$2.15\cdot10^{10}$	40.3
**28**	X = O	111.5	1.0	307.2	$5.79\cdot10^{10}$	46.9
**28**	X = S	118.6	0.9	320.1	$2.32\cdot10^{09}$	39.8

[a] One of the many entities that could cause undesired side-reactions and harm a TSQ is the formation of singlet oxygen ($^1O_2$). Thus, we computed the Gibbs free reaction energy (ΔG_R) at the M062X/6-311G(d,p) level for the formation of $^1O_2$ calculated using the following reaction: $^3TSQ\ +\ ^3O_2\ =>\ TSQ\ +\ ^1O_2$.

Such a process should ideally be endergonic ($\Delta G_R > 0$) to avoid the formation of $^1O_2$ (Table S4). The rationale lies in the fact that an endergonic process indicates an unfavorable thermodynamic condition for the formation of $^1O_2$. By evaluating the ΔG_R, we gain insights into the energetic favorability of the process and, consequently, the likelihood of avoiding singlet oxygen formation.

Table S5. MCI and HOMA of the 6π-electron rings of **8**, **10** and **15**, benzene, pyrrole, furan and thiophene in the S_0 state.

Species	MCI_{S0}	$HOMA_{S0}$	E_{T1} (kJ/mol)
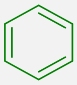	0.0420	0.997	–
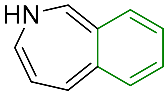 **15** (X = NH)	0.0525	0.895	4.9
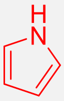	0.0420	0.868	–
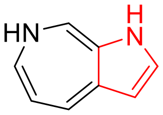 **10** (X = NH, X' = NH)	0.0095	0.339	25.7
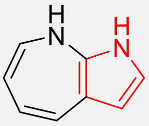 **8** (X = NH, X' = NH)	0.0348	0.844	116.4
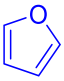	0.0255	0.285	–
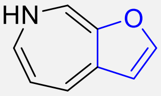 **10** (X = NH, X' = O)	0.0056	–0.318	29.3
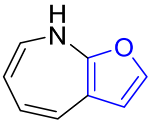 **8** (X = NH, X' = O)	0.0217	0.231	104.8
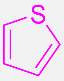	0.0399	0.776	–
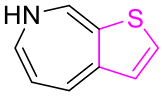 **10** (X = NH, X' = S)	0.0064	0.102	28.0
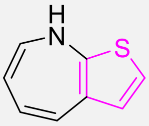 **8** (X = NH, X' = S)	0.0313	0.721	105.8

Table S6. MCI values for the 6π- and 8π-electron rings in a selection of twelve compounds. Normal text represents values in the S_0 state, while italicized text indicates values in the T_1 state.

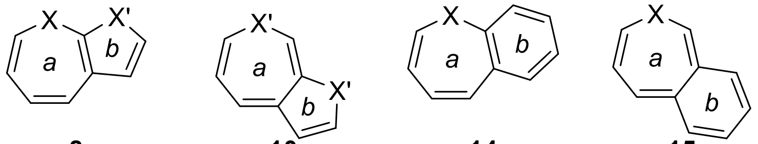

8 **10** **14** **15**

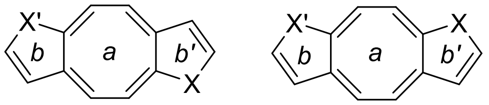

25 **26**

		a	b
8	X = NH X' = NH	0.0026, *0.0106*	0.0348, *0.0160*
8	X = NH X' = O	0.0029, *0.0128*	0.0217, *0.0082*
8	X = S X' = O	0.0018, *0.0132*	0.0213, *0.0094*
10	X = NH X' = NH	0.0043, *0.0107*	0.0095, *0.0206*
10	X = NH X' = O	0.0041, *0.0128*	0.0056, *0.0124*
10	X = S X' = O	0.0027, *0.0128*	0.0048, *0.0137*
14	X = S	0.0015, *0.0097*	0.0609, *0.0342*
15	X = S	0.0026, *0.0089*	0.0123, *0.0381*
25	X = N X' = O	0.0007, *0.0114*	0.0356, *0.0173*<br>(0.0215, *0.0090*)
25	X = O X' = O	0.0007, *0.0131*	0.0214, *0.0101*
26	X = N X' = O	0.0007, *0.0110*	0.0351, *0.0176*<br>(0.0203, *0.0093*)
26	X = O X' = O	0.0007, *0.0125*	0.0210, *0.0104*

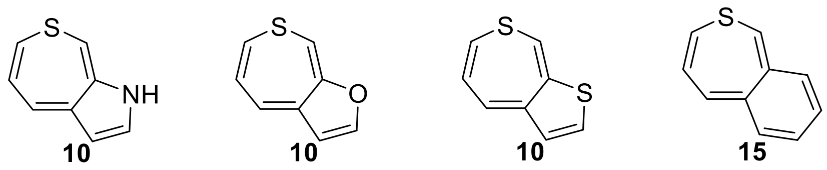

Figure S1. Thiepin compounds that have low triplet energy and cannot have 6π-electron Hückel-aromatic rings in S_0.

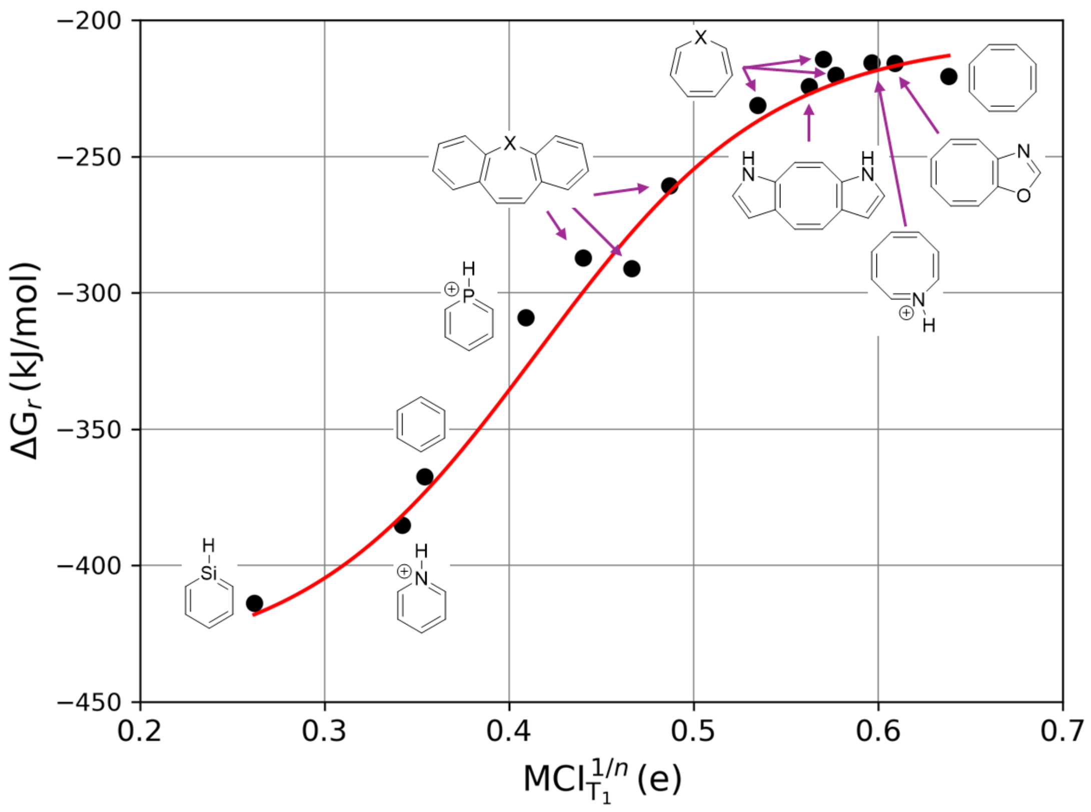

Figure S2: Schematic depiction for the reaction of methyl radical with various species. Reaction energies as a function of the T_1 state (anti)aromatic character of the (6π)8π-electron cycle as assessed through the MCI_{T1} values. For unsymmetric structures containing heteroatoms, the methyl group was added to the furthest carbon from the heteroatom.

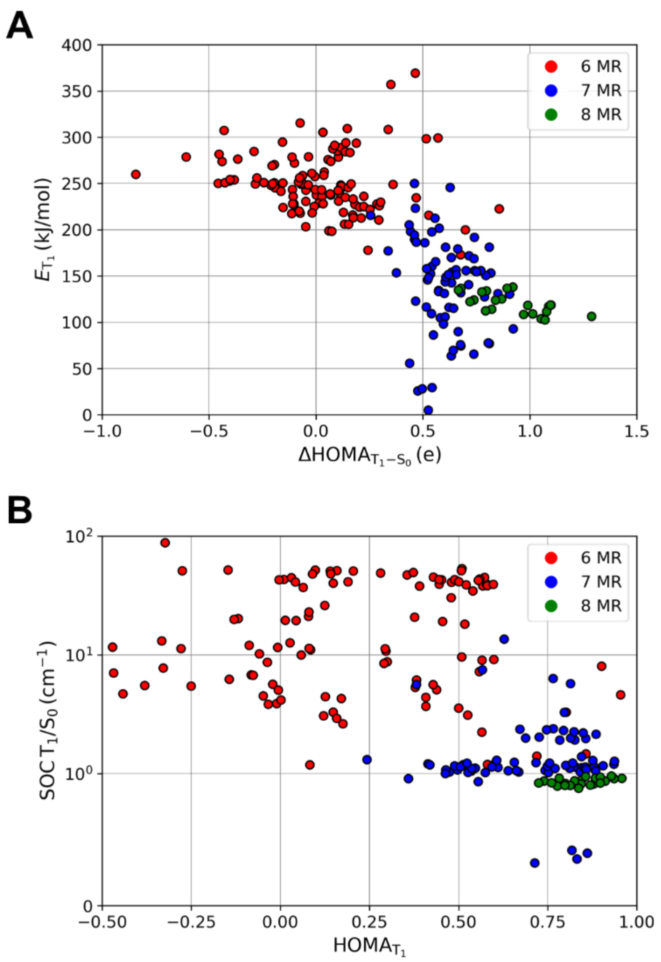

Figure S3. Variations of the triplet energies as a function of aromaticity changes based on HOMA (ΔHOMA$_{T1\text{-}S0}$) (A) and the spin-orbit coupling in dependence of aromaticity in T_1 based on HOMA$_{T1}$ (B) at different 6- (red), 7- (blue), and 8-MR (green) compounds.

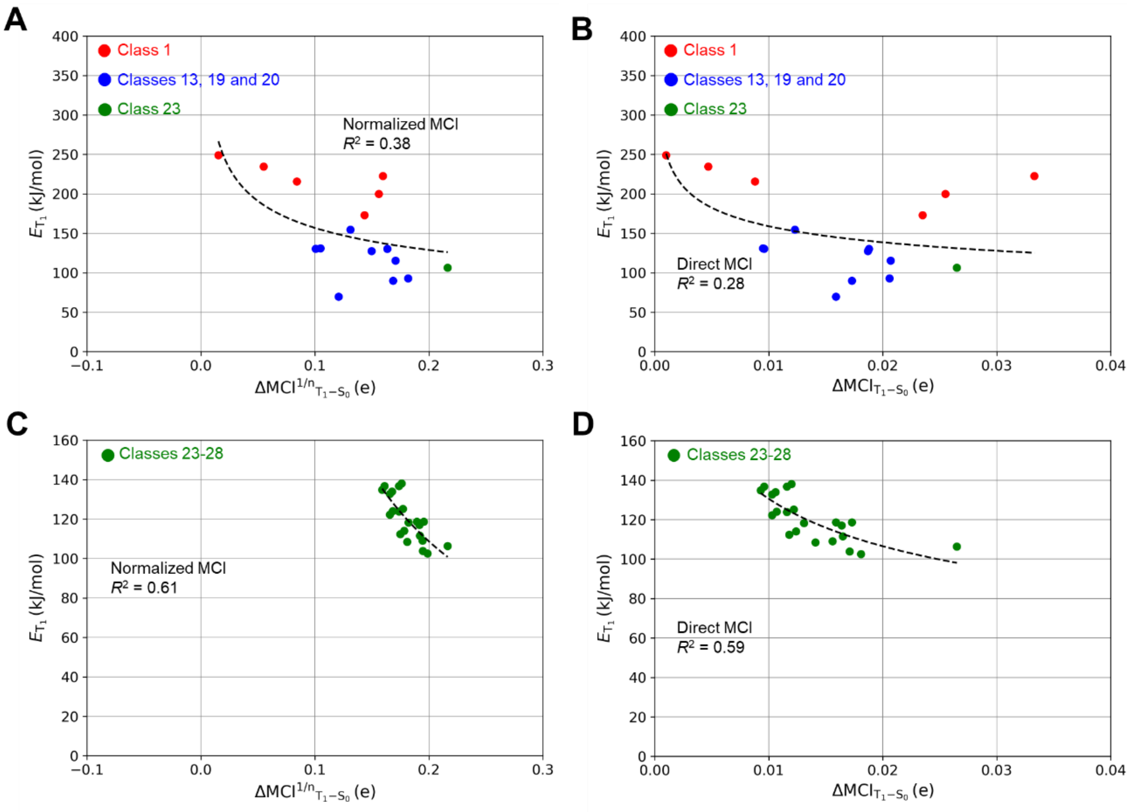

Figure S4. Variations of the triplet energies as a function of aromaticity changes based on normalized MCI ($\Delta MCI^{1/n}{}_{T1\text{-}S0}$) and direct MCI ($\Delta MCI^{1/n}{}_{T1\text{-}S0}$) of (A and B) monocyclic compounds **1**, **13**, **19**, **20** and **23**, and (C and D) 8-MR classes **22-28**. Triplet energies, and MCI values are computed at M06-2X/6-311G(d,p) level.

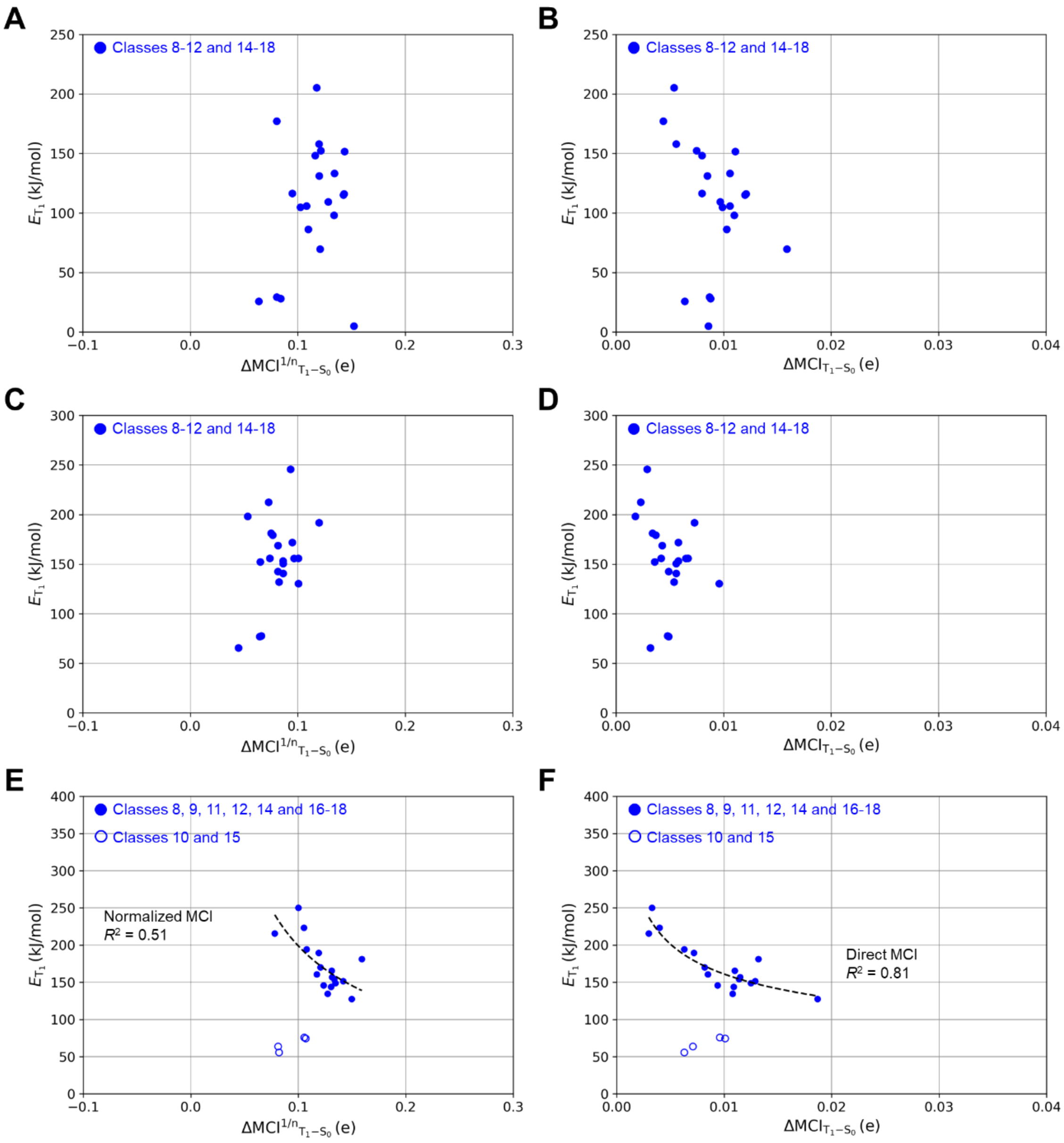

Figure S5. Variations of the triplet energies as a function of aromaticity changes based on normalized MCI ($\Delta MCI^{1/n}_{T1\text{-}S0}$) and direct MCI ($\Delta MCI^{1/n}_{T1\text{-}S0}$) of (A and B) azepines, (C and D) oxepines and (E and F) thiepines. Triplet energies, and MCI values are computed at M06-2X/6-311G(d,p) level.

Table S7. FLU and ΔFLU/FLU (electrons) values of 11 and 15- membered ring circuits in a selection of twelve compounds. Normal text represents values in the S_0 state, while italicized text indicates values in the T_1 state.

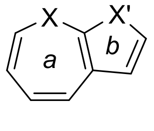

8

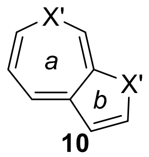

10

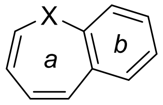

14

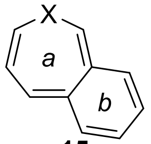

15

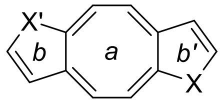

25

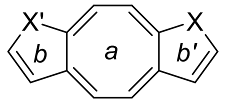

26

		FLU				*ΔFLU/FLU*		
		a	b<br>b'	a+b<br>(a+b')	b+a+b'	a	a+b	b+a+b'
8	X = NH X' = NH	0.0478, *0.0191*	0.0206, *0.0345*	0.0431, *0.0247*	-	0.130	0.2844	-
8	X = NH X' = O	0.0480, *0.0172*	0.0105, *0.0217*	0.0389, *0.0180*	-	0.190	0.2920	-
8	X = S X' = O	0.0388, *0.0063*	0.0087, *0.0167*	0.0314, *0.0102*	-	0.840	0.1444	-
10	X = NH X' = NH	0.0358, *0.0159*	0.0469, *0.0260*	0.0368, *0.0197*	-	0.338	0.6345	-
10	X = NH X' = O	0.0391, *0.0145*	0.0315, *0.0112*	0.0312, *0.0126*	-	0.384	0.5383	-
10	X = S X' = O	0.0370, *0.0059*	0.0351, *0.0104*	0.0292, *0.0066*	-	0.965	0.0243	-
14	X = S	0.0410, *0.0094*	0.0010, *0.0097*	0.0260, *0.0082*	-	2.053	1.3343	-
15	X = S	0.0302, *0.0079*	0.0376, *0.0064*	0.0307, 0.0051	-	0.775	0.5837	-
25	X = N X' = O	0.0464, *0.0102*	0.0173, *0.0308*<br>(0.0086, *0.0190*)	0.0415, *0.0154*<br>(0.0374, *0.0101*)	0.0355, *0.0142*	0.977	0.1358<br>0.2124	0.3982
25	X = O X' = O	0.0480, *0.0091*	0.0084, *0.0172*	0.0385, *0.0093*	0.0331, *0.0094*	1.027	0.1901	0.2694
26	X = N X' = O	0.0464, *0.0107*	0.0171, *0.0304*<br>(0.0085, *0.0185*)	0.0415, *0.0155*<br>(0.0373, *0.0103*)	0.0354, *0.0141*	0.895	0.1813<br>0.1799	0.4427
26	X = O X' = O	0.0479, *0.0098*	0.0083, *0.0164*	0.0384, *0.0094*	0.0329, *0.0092*	0.962	0.1509	0.3435

Table S8. HOMA values for 11 and 15-membered ring circuits in a selection of twelve compounds. Normal text represents values in the S_0 state, while italicized text indicates values in the T1 state.

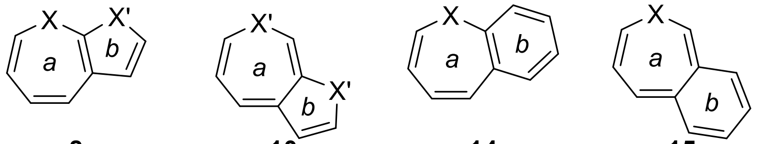

8 **10** **14** **15**

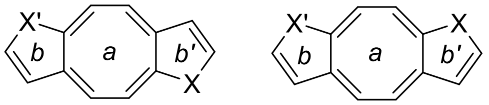

25 **26**

		HOMA			
		a	b	a+b<br>a+b'	b+a+b'
8	X = NH X' = NH	0.3130, *0.8289*	0.8442, *0.6682*	0.4413, *0.7699*	-
8	X = NH X' = O	0.2986, *0.8800*	0.2314, *0.0346*	0.1486, *0.4473*	-
8	X = S X' = O	0.2217, *0.8567*	0.2395, *0.0247*	0.1019, *0.4272*	-
10	X = NH X' = NH	0.3652, *0.8412*	0.3391, *0.7643*	0.5078, *0.8382*	-
10	X = NH X' = O	0.3417, *0.8845*	-0.3176, *0.1524*	0.0915, *0.5159*	-
10	X = S X' = O	0.1835, *0.8612*	-0.4399, *0.2016*	-0.0015, *0.5218*	-
14	X = S	0.0934, *0.7262*	0.9763, *0.7254*	0.4230, *0.7789*	-
15	X = S	0.2759, *0.7136*	0.0177, *0.8001*	0.3177, *0.8238*	-
25	X = N X' = O	0.0609, *0.7995*	0.8620, *0.5973*<br>(0.2165, *-0.1114*)	0.2543, *0.8248*<br>(-0.0173, *0.4518*)	0.1514, *0.5462*
25	X = O X' = O	0.0482, *0.8695*	0.2329, *-0.0299*	-0.0198, *0.5206*	-0.0587, *0.3213*
26	X = N X' = O	0.0570, *0.7771*	0.8672, *0.6146*<br>(0.2190, *-0.0957*)	0.2538, *0.8203*<br>(-0.0186, *0.4441*)	0.1523, *0.5494*
26	X = O X' = O	0.0439, *0.8368*	0.2359, *-0.0060*	-0.0214, *0.5107*	-0.0588, *0.3244*

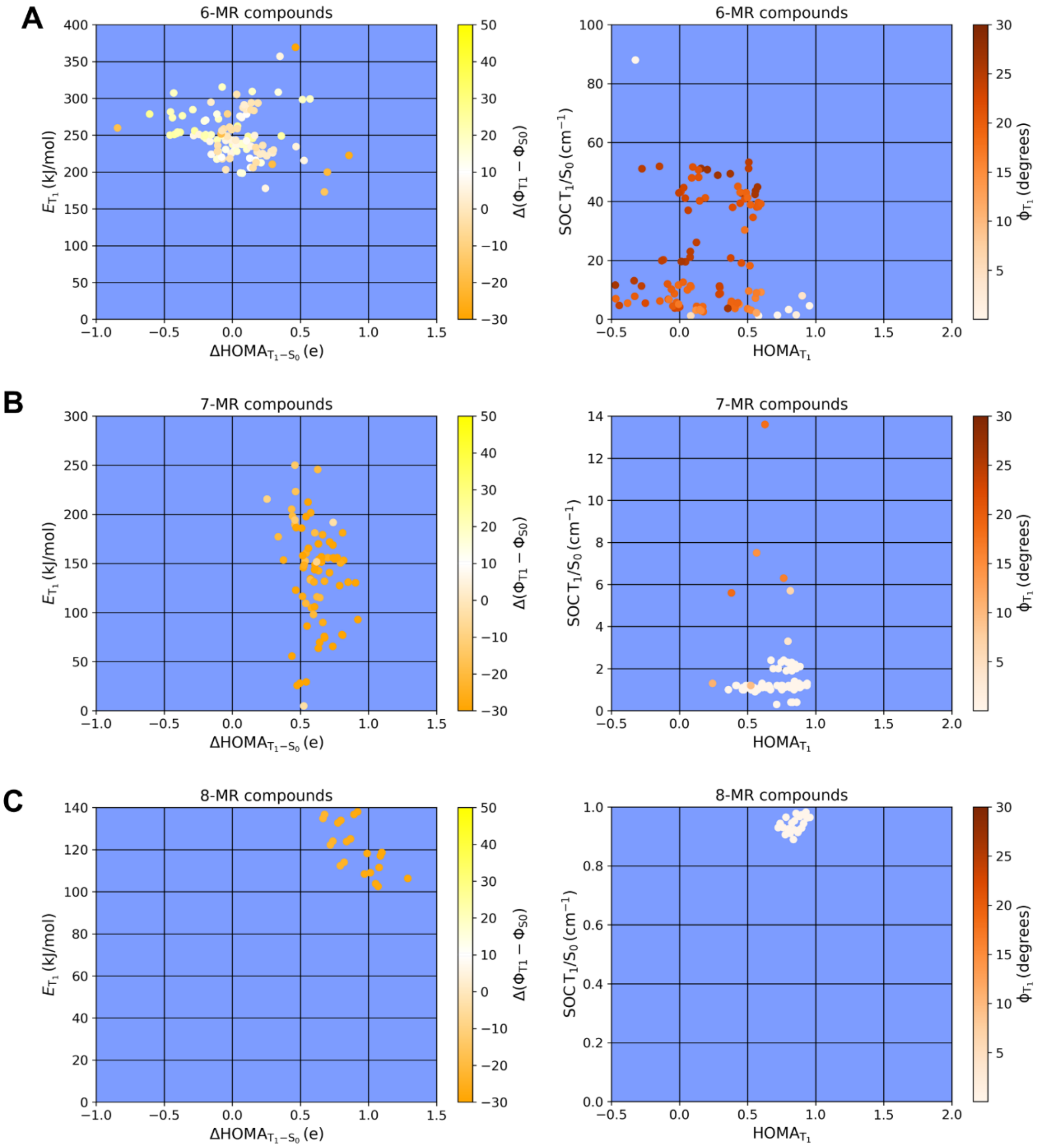

Figure S6. Variations of the triplet energies as a function of aromaticity changes based on HOMA (left panels) and the spin-orbit coupling in dependence of aromaticity in T_1 based on HOMA (right panels) at different geometric parameters, i.e., dihedral angles (ϕ, color scale), for the (A) 6-, (B) 7-, and (C) 8-MR compounds. Legends next to each plot show color scales for $\Delta(\phi T1 - \phi S0)$ values, with scales from yellow (positive) to orange (negative) for left panels and from white (zero) to dark red (positive) for right panels.

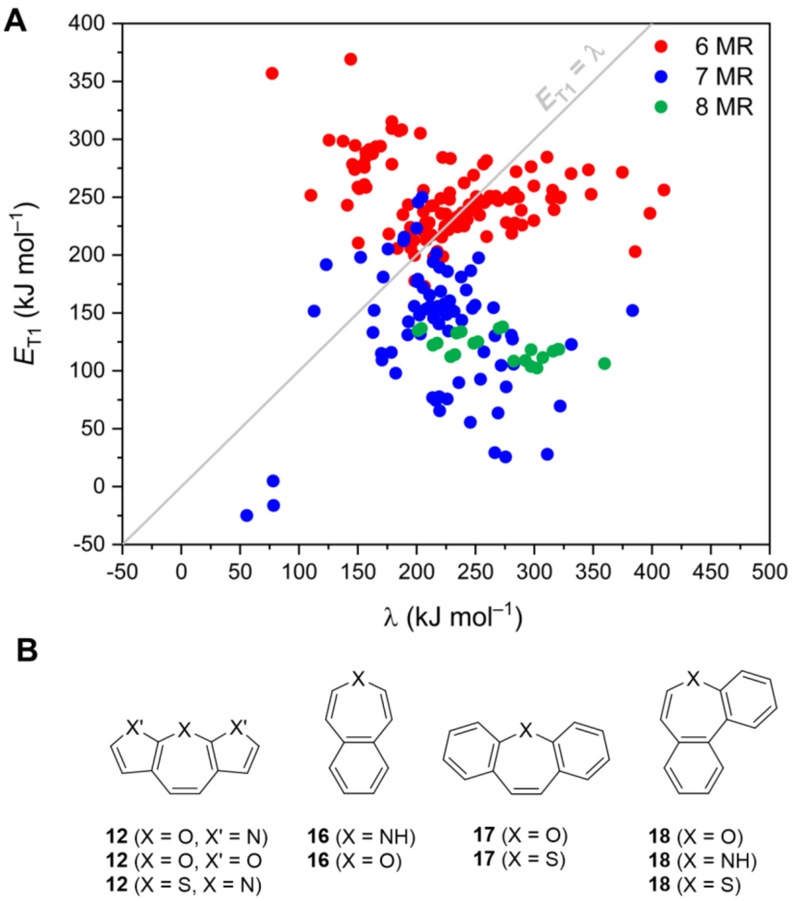

Figure S7. (A) Comparison of reorganization energy (λ) and E_{T1}; grey reference line indicated where E_{T1} is equal to λ. (B) Structures of the ten 7MR compounds that lie above the reference line.

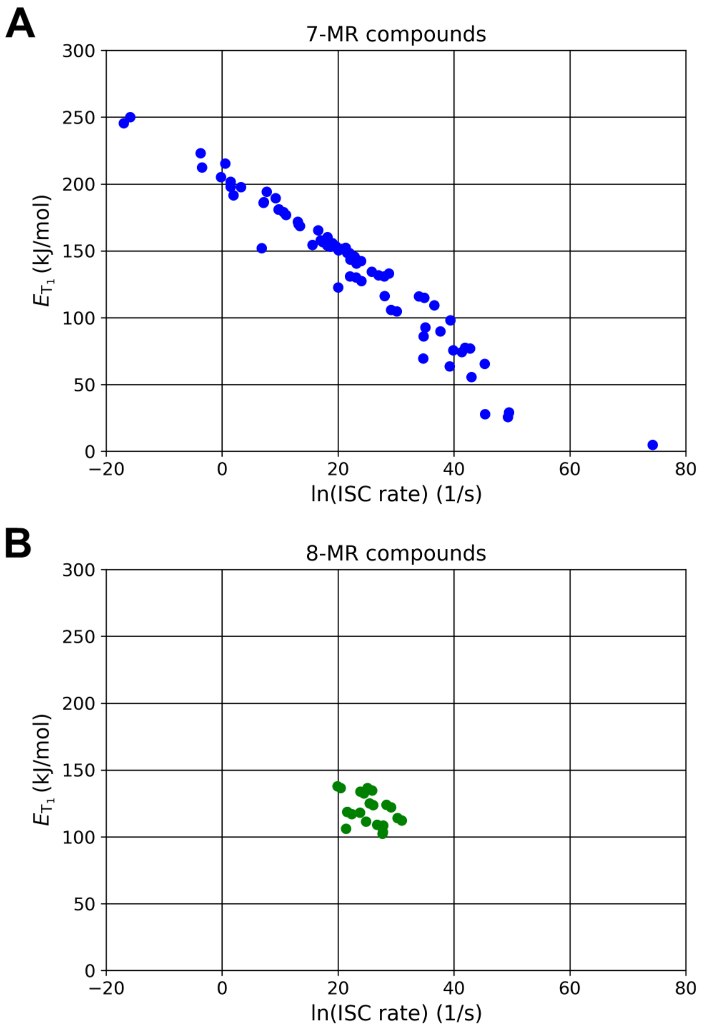

Figure S8. Triplet energy versus the natural logarithm of the ISC rate for the 7- (A) and 8-MR (B) compounds.

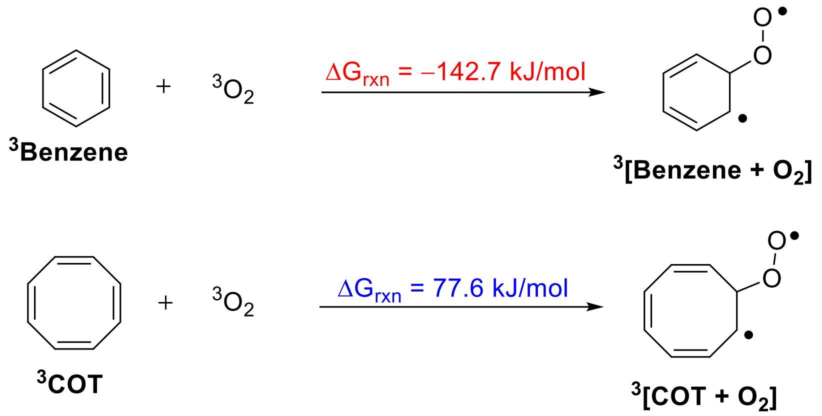

Figure S9: Gibbs free energies of reaction of triplet benzene and COT with triplet oxygen.

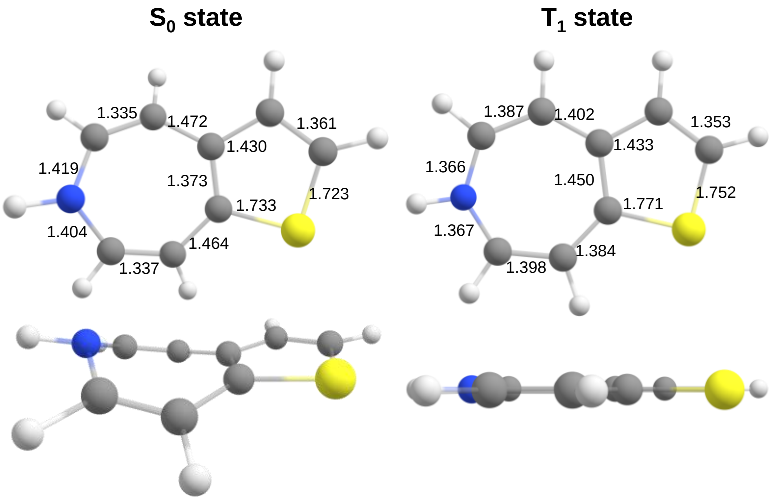

$\Delta G^{\ddagger}_{inv}$ = 4.4 kJ/mol MCI$^{(1/n)}_{T1}$ = 0.541 e

E_{T1} = 115.1 kJ/mol

$k_{ISC} = 1.39 \cdot 10^{15}\ s^{-1}$

Figure S10: One of the most suitable 7-MR TSQ candidate compounds according to our computations (**11** (X = NH; X' = S)).

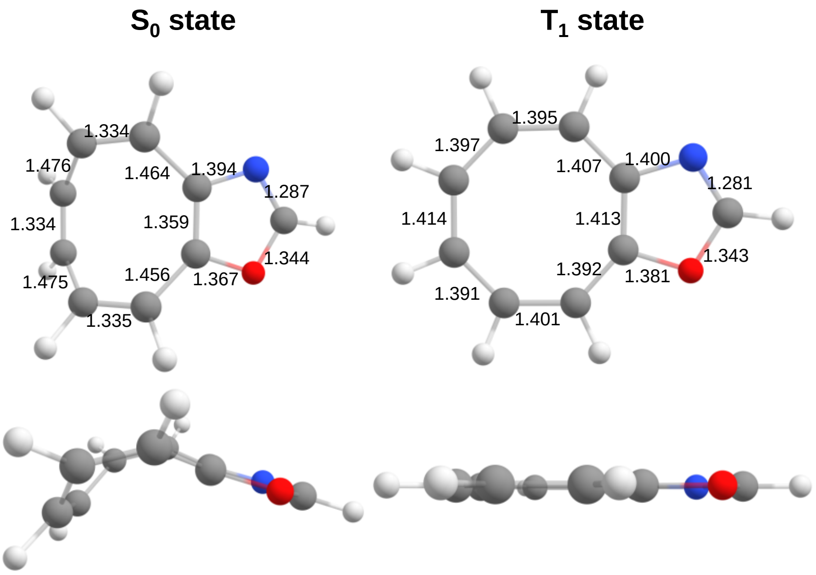

$\Delta G^{\ddagger}_{inv}$ = 40.1 kJ/mol MCI$^{(1/n)}_{T1}$ = 0.609 e

E_{T1} = 102.5 kJ/mol

$k_{ISC} = 1.03 \cdot 10^{12}\ s^{-1}$

Figure S11: One of the most suitable 8-MR TSQ candidate compounds according to our computations (**26** (X = O)).

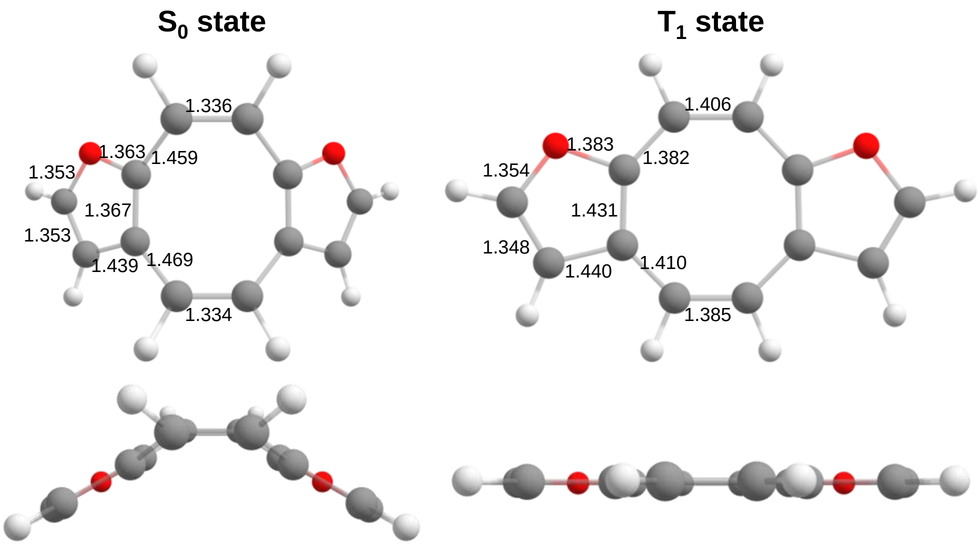

$\Delta G^{\ddagger}_{inv}$ = 22.1 kJ/mol $MCI^{(1/n)}_{T1}$ = 0.578 e

E_{T1} = 112.3 kJ/mol

$k_{ISC} = 2.84 \cdot 10^{13}\ s^{-1}$

Figure S12: One of the most suitable 8-MR TSQ candidate compounds according to our computations (**27** (X = O)).

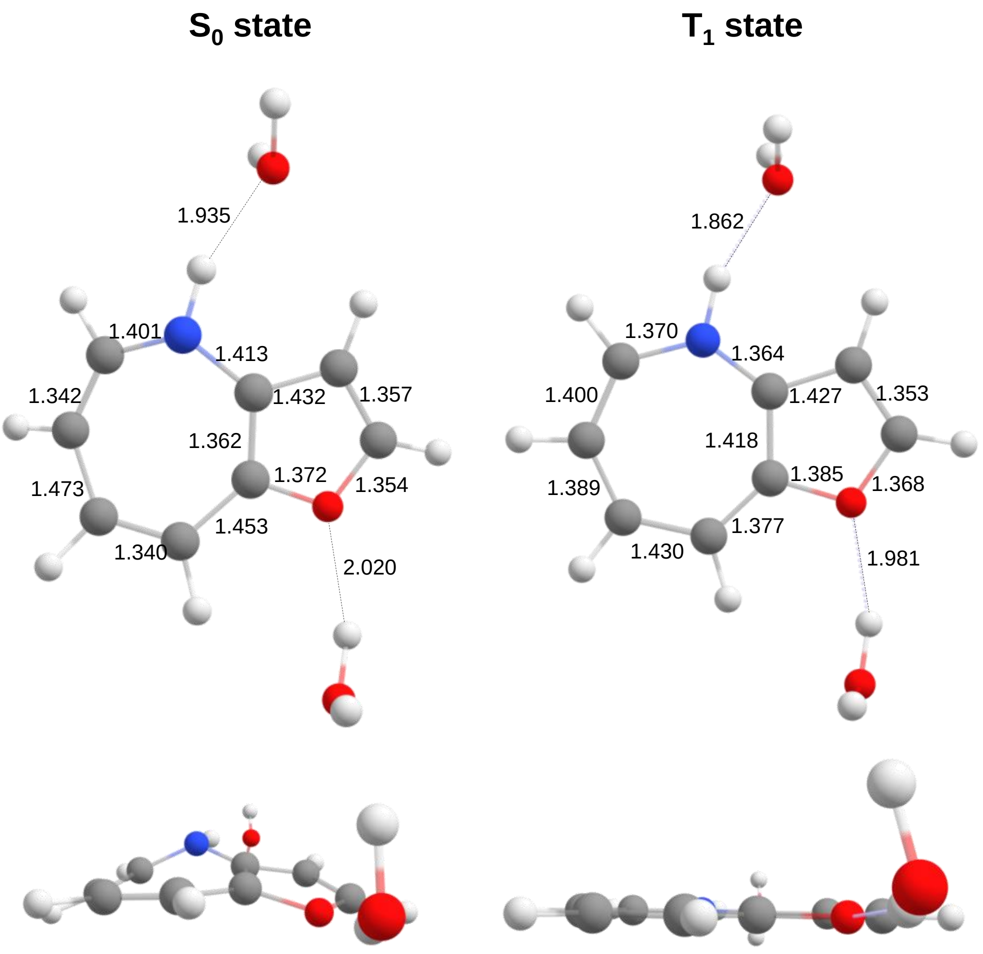

$\Delta G^{\ddagger}_{inv} = 5.4$ kJ/mol $\qquad$ $MCI^{(1/n)}{}_{T1} = 0.542$ e

$E_{T1} = 94.5$ kJ/mol

$k_{ISC} = 7.63 \cdot 10^{17}\ s^{-1}$

Figure S13: One of the most suitable 7-MR TSQ candidate compounds according to our computations (**9** (X = NH; X’ = O)) with two water molecules coordinated.

Substitution effects

To test for substitution effects, compounds **14** (X = NH) and **28** (X = NH) were chosen, as they both have reported synthetic procedures in the literature. The compounds were scanned with methoxy substituents (modelling for solubilizing alkyl ethers in aqueous media) at each available position (*a* – *h* for **14**; *a* – *g* for **28**), and the resulting T_1 energies computed at the same level of theory. Substitution with methoxy groups has negligible effects on the energies (and geometries) of the T_1 state, with the exception of positions *a* and *d* in compound **14**, and positions *b*, *f*, and *g*¸ in compound **28**. In general, the reason for the increased E_{T1} is due to the stabilization of S_0 rather than T_1.

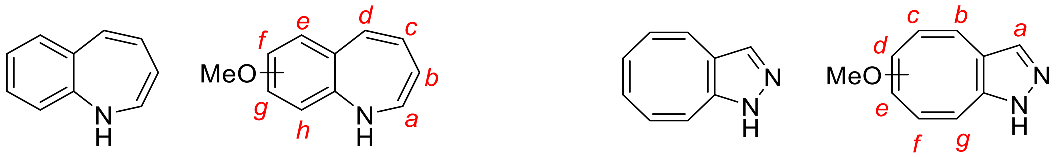

14 (X = NH) **14a–h** **28** (X = NH) **28a–g**

		E_{T1}
**14**	X = NH	131.1
**14**^{a}		168.9
**14b**		136.9
**14c**		142.3
**14d**		152.6
**14e**		137.8
**14f**		141.8
**14g**		144.9
**14h**		133.5

		E_{T1}
**28**	X = NH	118.2
**28a**		120.5
**28b**		139.9
**28c**		123.3
**28d**		124.3
**28e**		120.5
**28f**		138.4
**28g**		131.6

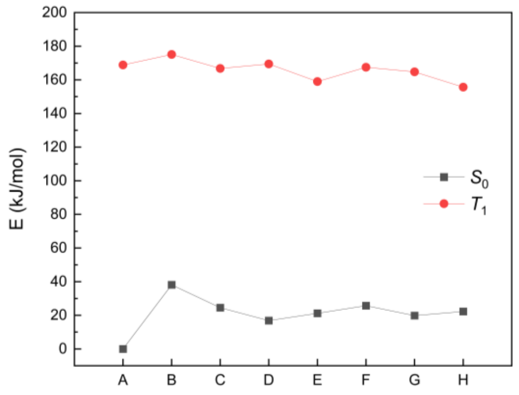

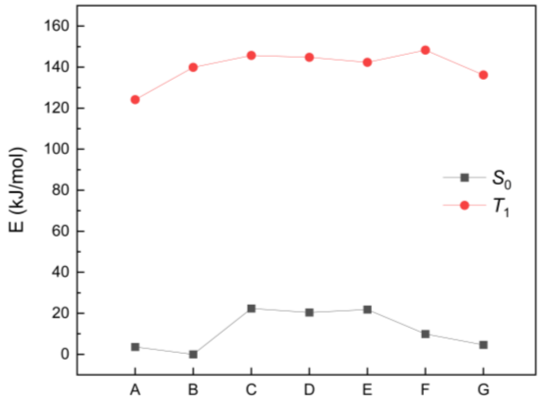

Figure S14: Effects of substitution to the E_{T1} with methoxy groups for compound **14** (X= NH) and **28** (X = NH).

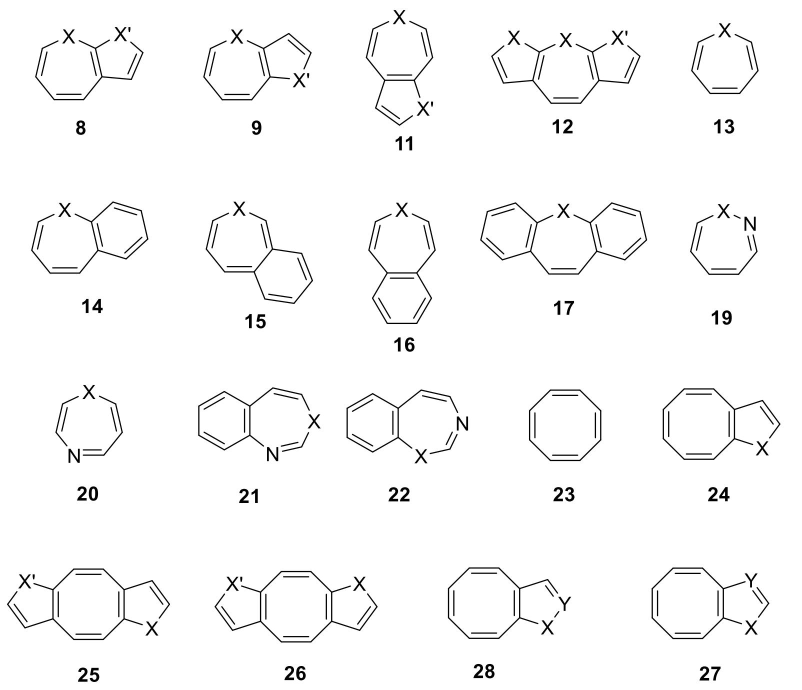

8 X = NH X' = NH	11 X = O X' = O	24 X = O
8 X = NH X' = O	11 X = O X' = S	24 X = S
8 X = NH X' = S	11 X = S X' = O	25 X = N X' = N
8 X = O X' = NH	11 X = S X' = S	25 X = N X' = O
8 X = O X' = S	12 X = NH X' = O	25 X = N X' = S
8 X = O X' = O	12 X = NH X' = S	25 X = O X' = O
8 X = S X' = O	13 X = S	25 X = O X' = S
8 X = S X' = S	13 X = O	25 X = S X' = S
9 X = NH X' = NH	**14 X = NH**	26 X = N X' = N
9 X = NH X' = O	15 X = O	26 X = N X' = O
9 X = NH X' = S	16 X = NH	26 X = N X' = S
9 X = O X' = NH	17 X = NH	26 X = O X' = S
9 X = O X' = O	19 X = NH	26 X = S X' = S
9 X = O X' = S	19 X = O	27 X = NH
9 X = S X' = NH	20 X = NH	27 X = O
9 X = S X' = O	20 X = O	27 X = S
9 X = S X' = S	21 X = NH	28 X = NH
11 X = NH X' = NH	22 X = NH	28 X = O
11 X = NH X' = O	23 COT	28 X = S
11 X = NH X' = S	24 X = NH	

Figure S15: The list of parent heterocyclic compounds with SOCs more than 0.9 cm^{-1}, are planar in the T_1 state, have triplet energies within the ideal span (80 - 200 kJ/mol), and cannot form singlet oxygen.

Table S9. E_{T1}, k_{ISC}, E_{S1} (vertical) and MCI(T_1) split into smaller tables with E_{T1} ranges of 20 kJ/mol.

		E_{T1}	k_{ISC}	E_{S1}	MCI_{T1}
	E_{T1}: 80.0 – 100.0 kJ/mol				
9	X = NH X' = O	98	$1.23 \cdot 10^{17}$	271.5	0.53564
20	X = NH	89.8	$2.23 \cdot 10^{16}$	280.9	0.56725
19	X = NH	92.8	$1.68 \cdot 10^{15}$	305.9	0.58055
9	X = NH X' = S	86.2	$1.19 \cdot 10^{15}$	418.1	0.53692
	E_{T1}: 100.1 – 120.0 kJ/mol				
9	X = NH X' = NH	109.3	$7.58 \cdot 10^{15}$	274.4	0.52733
11	X = NH X' = S	115.1	$1.39 \cdot 10^{15}$	276.7	0.54114
11	X = NH X' = O	116	$5.33 \cdot 10^{14}$	284.3	0.54151
8	X = NH X' = O	104.8	$1.16 \cdot 10^{13}$	422.6	0.53663
8	X = NH X' = S	105.8	$4.48 \cdot 10^{12}$	438.9	0.53999
8	X = NH X' = NH	116.4	$1.43 \cdot 10^{12}$	414.2	0.52207
24	X = NH	108.4	$1.13 \cdot 10^{12}$	337.9	0.59087
24	X = O	103.8	$1.09 \cdot 10^{12}$	346.3	0.60459
27	X = O	102.5	$1.03 \cdot 10^{12}$	349.2	0.6088
27	X = NH	109	$4.12 \cdot 10^{11}$	345.7	0.59758
28	X = NH	118.2	$2.15 \cdot 10^{10}$	366.5	0.58536
28	X = O	111.5	$5.79 \cdot 10^{10}$	365.1	0.6024
24	X = S	117	$5.10 \cdot 10^{09}$	369.1	0.60161
27	X = S	118.6	$2.36 \cdot 10^{09}$	372.1	0.60551
28	X = S	118.6	$2.32 \cdot 10^{09}$	375.3	0.59936
23	COT	106.3	$1.86 \cdot 10^{09}$	381.7	0.63814
	E_{T1}: 120.1 – 140.0 kJ/mol				
26	X = N X' = O	122.2	$4.37 \cdot 10^{12}$	308.3	0.56938
11	X = NH X' = NH	133.2	$3.00 \cdot 10^{12}$	287.2	0.53307
25	X = N X' = O	124	$1.96 \cdot 10^{12}$	311.8	0.57165
14	X = NH	131.1	$1.37 \cdot 10^{12}$	316.2	0.51904
9	X = O X' = O	131.9	$5.13 \cdot 10^{11}$	349.3	0.49752
26	X = O X' = S	123.8	$2.03 \cdot 10^{11}$	334.8	0.57723

9	X = S X' = O	134.4	$1.58 \cdot 10^{11}$	330.2	0.53617
26	X = N X' = N	134.8	$1.70 \cdot 10^{11}$	311	0.56249
25	X = O X' = S	125.1	$1.08 \cdot 10^{11}$	337.9	0.5803
25	X = N X' = N	136.7	$7.83 \cdot 10^{10}$	314.3	0.56433
26	X = N X' = S	132.7	$4.02 \cdot 10^{10}$	330.7	0.56882
13	X = S	127.4	$2.75 \cdot 10^{10}$	349.1	0.5769
25	X = N X' = S	133.9	$2.25 \cdot 10^{10}$	334	0.57082
13	X = O	130.3	$1.09 \cdot 10^{10}$	408.5	0.53442
20	X = O	130.9	$3.62 \cdot 10^{09}$	377.8	0.53213
26	X = S X' = S	136.7	$7.64 \cdot 10^{08}$	359.1	0.57683
21	X = NH	122.8	$4.80 \cdot 10^{08}$	455.6	0.53348
25	X = S X' = S	138	$4.41 \cdot 10^{08}$	361.6	0.57965

E_{T1}: 140.1 – 160.0 kJ/mol					
9	X = O X' = NH	142.5	$2.45 \cdot 10^{10}$	350	0.49009
9	X = O X' = S	140.6	$1.10 \cdot 10^{10}$	370.7	0.49835
9	X = S X' = NH	145.7	$8.48 \cdot 10^{09}$	331.6	0.52598
9	X = S X' = S	143.7	$4.07 \cdot 10^{09}$	342.6	0.53622
12	X = NH X' = S	148.2	$3.52 \cdot 10^{09}$	332.8	0.51459
11	X = S X' = O	148.7	$2.23 \cdot 10^{09}$	342.3	0.54626
12	X = NH X' = O	152.3	$1.81 \cdot 10^{09}$	312.1	0.50855
16	X = NH	151.6	$9.47 \cdot 10^{08}$	248.7	0.53461
11	X = S X' = S	151.4	$6.55 \cdot 10^{08}$	349.8	0.54718
8	X = O X' = O	150.5	$5.13 \cdot 10^{08}$	376.8	0.49819
22	X = NH	153.4	$3.43 \cdot 10^{08}$	346.5	0.5165
8	X = O X' = NH	155.8	$1.85 \cdot 10^{08}$	363.4	0.48276
8	X = O X' = S	153.1	$1.22 \cdot 10^{08}$	387.9	0.50071
11	X = O X' = S	155.8	$1.14 \cdot 10^{08}$	391	0.50567
8	X = S X' = O	154.1	$7.40 \cdot 10^{07}$	360.4	0.53911
11	X = O X' = O	155.7	$7.35 \cdot 10^{07}$	395.5	0.50485
8	X = S X' = S	156.7	$3.41 \cdot 10^{07}$	356.4	0.54031
17	X = NH	157.9	$2.32 \cdot 10^{07}$	434.3	0.48708
19	X = O	154.5	$5.83 \cdot 10^{06}$	390.1	0.54558
15	X = O	152.2	$8.95 \cdot 10^{02}$	462.7	0.47664

	E_{T1}: 160.1 – 180.0 kJ/mol				
8	X = S X' = NH	160.5	$7.51 \cdot 10^{07}$	-	0.51921
11	X = S X' = NH	165.4	$1.57 \cdot 10^{07}$	-	0.53656
2	X = S X' = NH X" = NH	177.8	$3.51 \cdot 10^{06}$	-	0.46628
1	X = NH X’ = NH	172.9	$1.03 \cdot 10^{06}$	-	0.55134
14	X = O	168.7	$6.70 \cdot 10^{05}$	-	0.48032
14	X = S	169.9	$5.28 \cdot 10^{05}$	-	0.51545
11	X = O X' = NH	171.8	$4.46 \cdot 10^{05}$	-	0.49691
12	X = NH X' = NH	177.1	$6.09 \cdot 10^{04}$	-	0.4826
12	X = O X' = S	179.2	$3.94 \cdot 10^{04}$	-	0.47118

	E_{T1}: 180.1 – 200.0 kJ/mol				
12	X = S X' = S	194.2	$2.21 \cdot 10^{03}$	-	0.49857
22	X = S	186.5	$1.32 \cdot 10^{03}$	-	0.50767
22	X = O	185.9	$1.26 \cdot 10^{03}$	-	0.47842
4	X = S X' = NH X" = NH	198.3	$9.07 \cdot 10^{02}$	-	0.42451
1	X = NH X’ = S	199.8	$4.62 \cdot 10^{02}$	-	0.55605
3	X = S X' = NH X" = NH	198.8	$4.18 \cdot 10^{02}$	-	0.42469
21	X = S	197.6	$2.57 \cdot 10^{01}$	-	0.54134
16	X = O	191.7	$6.83 \cdot 10^{00}$	-	0.50685
12	X = O X' = NH	198.1	$4.36 \cdot 10^{00}$	-	0.4356

References

1 M. J. Frisch, G. W. Trucks, H. B. Schlegel, G. E. Scuseria, M. Robb, J. R. Cheeseman, G. Scalmani, V. Barone, B. Mennucci, G. A. Petersson, H. Nakatsuji, M. Caricato, X. Li, H. P. Hratchian, A. F. Izmaylov, J. Bloino, G. Zheng, J. L. Sonnenberg, M. Hada, M. Ehara, K. Toyota, R. Fukuda, J. Hasegawa, M. Ishida, T. Nakajima, Y. Honda, O. Kitao, H. Nakai, T. Vreven, J. A. Montgomery, Jr., J. E. Peralta, F. Ogliaro, M. Bearpark, J. J. Heyd, E. Brothers, K. N. Kudin, V. N. Staroverov, R. Kobayashi, J. Normand, K. Raghavachari, A. Rendell, J. C. Burant, S. S. Iyengar, J. Tomasi, M. Cossi, N. Rega, J. M. Millam, M. Klene, J. E. Knox, J. B. Cross, V. Bakken, C. Adamo, J. Jaramillo, R. Gomperts, R. E. Stratmann, O. Yazyev, A. Austin, R. Cammi, C. Pomelli, J. W. Ochterski, R. L. Martin, K. Morokuma, V. G. Zakrzewski, G. A. Voth, P. Salvador, J. J. Dannenberg, S. Dapprich, A. D. Daniels, Ö. Farkas, J. Foresman, J. V. Ortiz, J. Cioslowski and D. J. Fox, *Gaussian 16*; Gaussian Inc.: Wallingford CT 2016.

2 Y. Zhao and D. G. Truhlar, *Theor. Chem. Acc.*, 2008, **120**, 215-241.

3 J. Tomasi, B. Mennucci and R. Cammi, *Chem. Rev.*, 2005, **105**, 2999-3093.

4 X. Gao, S. Bai, D. Fazzi, T. Niehaus, M. Barbatti and W. Thiel, *J. Chem. Theory Comput.*, 2017, **13**, 515-524.

5 C. M. Marian, *Wiley Interdiscip. Rev. Comput. Mol. Sci.*, 2012, **2**, 187-203.

6 C. Riplinger, B. Sandhoefer, A. Hansen and F. Neese, *J. Chem. Phys.*, 2013, **139**, 134101.

7 C. Riplinger and F. Neese, *J. Chem. Phys.*, 2013, **138**, 034106.

8 D. G. Liakos and F. Neese, *J. Chem. Theory Comput.*, 2015, **11**, 4054-4063.

9 P. Bultinck, M. Rafat, R. Ponec, B. Van Gheluwe, R. Carbó-Dorca and P. Popelier, *J. Phys. Chem. A*, 2006, **110**, 7642-7648.

10 Krygowski, T. M. Crystallographic Studies of Inter- and Intramolecular Interactions Reflected in Aromatic Character of π-Electron Systems. *J. Chem. Inf. Model.* **1993**, *33*, 70– 78.

11 T. M. Krygowski, *J. Chem. Inf. Model.*, 1993, **33**, 70-78.

12 Matito, E. *ESI-3D: Electron Sharing Indexes Program for 3D Molecular Space Partitioning*; Institute of Computational Chemistry and Catalysis: Girona, Catalonia, Spain, **2006**..

13 *AIMAll (Version 17.11.14 B)*; TK Gristmill Software: Overland Park, KS, US, **2018**. http://aim.tkgristmill.com..

14 R. K. Summerbell, R. R. Umhoefer, *J. Am. Chem. Soc.*, 1939, **61**, 3020-3022.

15 M. Schoufs, J. Meijer, L. Brandsma, *Recl. Trav. Chim. Pays-Bas*, 1980, **99**(1), 12-14.

16 A. Castello-Mico, J. Nafe, K. Higashida, K. Karaghiosoff, M. Gingras, P. Knochel, *Org. Lett.*, 2017, **19**(2), 360-363.

17 R. L. P. De Jong, L. Brandsma, *J. Chem. Soc., Chem. Commun.*, 1983, **19**, 1056-1057.

18 H. C. Srivastava, K. V. Ramalingam, A. S. Chaudhari, *Tetrahedron Lett.*, 1969, **31**, 2643-2646.
19 C. Dostert, T. J. J. Mueller, *Org. Chem. Front.*, 2015, **2**(5), 481-491.
20 H. Ito, D. Watanabe, T. Yamamoto, N. Tsushima, H. Muraoka, S. Ogawa, *Chem. Lett.*, 2013, **42**(6), 646-648.
21 H. Bock, B. Roth, *Phosphorus Sulfur*, 1983, **14**, 211.
22 G. L. Morgans, E. L. Ngidi, L. G. Madeley, S. D. Khanye, J. P. Michael, C. B. de Koning, W. A. L. van Otterlo, *Tetrahedron*, 2009, **65**(51), 10650-10659.
23 J. Hamer, R. E. Holliday, *J. Org. Chem.*, 1963, **28**(9), 2488.
24 C. O. Okafor, *Heterocycles*, 1977, **7**, 391-427.
25 C. J. Grol, H. Rollema, D. Dijkstra, B. H. C. Westerink, *J. Med. Chem.*, 1980, **23**, 322-324.
26 S. Yasuike, F. Nakashima, J. Kurita, T. Tsuchiya, *Heterocycles*, 1997, **45**(10), 1899-1902.
27 a) S. Yasuike, S.-I. Shiratori, J. Kurita, T. Tsuchiya, *Chem. Pharm. Bull.*, 1999, **47**, 1108-1114. b) H. Hofmann, H. Djafari, *Z. Naturforsch., B: Chem. Sci.*, 1989, **44**, 220. c) H. Hofmann, H. Fischer, M. De Vries, *Z. Naturforsch., B: Chem. Sci.*, 1990, **45**, 1573. d) K. Sindelar, M. Protiva, *Collect. Czech. Chem. Commun.*, 1968, **33**, 4315. e) V. J. Traynelis, Y. Yoshikawa, J. C. Sih, L. J. Miller, J. R. Livingston Jr., *J. Org. Chem.*, 1973, **38**, 3978. f) V. J. Traynelis, Y. Yoshikawa, S. M. Tarka, J. R. Livingston Jr., *J. Org. Chem.*, 1973, **38**, 3986.
28 H. Igeta, H. Arai, H. Hasegawa, T. Tsuchiya, *Chem. Pharm. Bull.*, 1975, **23**, 2791–2797.
29 H. Plieninger, D. Wild, *Chem. Ber.*, 1966, **99**(10), 3070–3075.
30 S. Yasuike, T. Kiharada, T. Tsuchiya, J. Kurita, *Chem. Pharm. Bull.*, 2003, **51**, 1283–1288.
31 a) J. O. Jilek, V. Seidlova, E. Svatek, M. Protiva, J. Pomykacek and Z. Sedivy, *Monatsh. Chem.*, 1965, **96**, 182-207. b) J. O. Jilek, E. Svatek, J. Metysova, J. Pomykacek and M. Protiva, *Collect. Czech. Chem. Commun.*, 1967, **32**, 3186. c) H. Seidl and K. Biemann, *J. Heterocycl. Chem.*, 1967, **4**, 209. d) M. Queisnerova, E. Svatek and J. Metysova, *Cesk. Farm.*, 1968, **17**, 248. e) K. Sindelar, J. Metysova, J. Metys and M. Protiva, *Naturwissenschaften*, 1969, **56**, 374. f) J. C. L. Fouche, *Bull. Soc. Chim. Fr.*, 1970, **4**, 1376. g) J. O. Jilek, K. Sindelar, J. Pomykacek, O. Horesovsky, K. Pelz, E. Svatek, B. Kakac, J. Holubek, J. Metysova and M. Protiva, *Collect. Czech. Chem. Commun.*, 1973, **38**, 115. h) R. Leardini and G. Placucci, *J. Heterocycl. Chem.*, 1976, **13**, 277. i) P. Negrini, L. Cappellini, A. De Pascale, A. Frigerio, D. Macciantelli, M. McCamish, G. Placucci, E. Rossi and G. Testoni, *Anal. Chem. Symp. Ser.*, 1980, **4**, 253. j) K. Sindelar, J. Holubek, M. Ryska, E. Svatek, J. Urban, J. Grimova, I. Cervena, M. Hrubantova and M. Protiva, *Collect. Czech. Chem. Commun.*, 1983, **48**, 1187. k) H. Shirani and T. Janosik, *J. Org. Chem.*, 2007, **72**, 8984-8986. l) M. Saito, T. Yamamoto, I. Osaka, E. Miyazaki, K. Takimiya, H. Kuwabara and M. Ikeda, *Tetrahedron Lett.*, 2010,

51, 5277-5280. m) T. Takeda and A. Tsubouchi, *Sci. Synth.*, 2010, **47a**, 247-325. n) T. H. Jepsen, M. Larsen, M. Joergensen and M. B. Nielsen, *Synlett*, 2012, **23**, 418-422. o) T. Stopka, L. Marzo, M. Zurro, S. Janich, E.-U. Wuerthwein, C. G. Daniliuc, J. Aleman and O. G. Mancheno, *Angew. Chem., Int. Ed.*, 2015, **54**, 5049-5053. p) A. Gini and O. G. Mancheno, *Synlett*, 2016, **27**, 526-539. q) R. Li, B. Ma, S. Li, C. Lu and P. An, *Chem. Sci.*, 2023, **14**, 8905-8913.

32 G. W. Griffin, S. K. Satra, N. E. Brightwell, K. Ishikawa, N. S. Bhacca, *Tetrahedron Lett.*, 1976, **16**, 1239–1242.

33 (a) B. A. Hess Jr., L. J. Schaad, C. W. Holyoke Jr., *Tetrahedron*, 1972, **28**, 3657. (b) M. J. S. Dewar, N. Trinajstic, *Tetrahedron*, 1970, **26**, 4269.

34 K. Nakasuji, K. Kawamura, T. Ishihara, I. Murata, *Angew. Chem.*, 1976, **88**(19), 650–651.

35 D. C. Sanders, A. Marczak, J. L. Melendez, H. Shechter, *J. Org. Chem.*, 1987, **52**(25), 5622–5624.
